# Mapping proteins to disease terminologies: from UniProt to MeSH

**DOI:** 10.1186/1471-2105-9-S5-S3

**Published:** 2008-04-29

**Authors:** Anaïs Mottaz, Yum L Yip, Patrick Ruch, Anne-Lise Veuthey

**Affiliations:** 1 Swiss-Prot Group, Swiss Institute of Bioinformatics, 1211 Genève 4, Switzerland; 2 Department of Structural Biology and Bioinformatics, University of Geneva, 1211 Genève 4, Switzerland; 3 Medical Informatics Service, Hôpitaux Universitaire de Genève, 1211 Genève 4, Switzerland

## Abstract

**Background:**

Although the UniProt KnowledgeBase is not a medical-oriented database, it contains information on more than 2,000 human proteins involved in pathologies. However, these annotations are not standardized, which impairs the interoperability between biological and clinical resources. In order to make these data easily accessible to clinical researchers, we have developed a procedure to link diseases described in the UniProtKB/Swiss-Prot entries to the MeSH disease terminology.

**Results:**

We mapped disease names extracted either from the UniProtKB/Swiss-Prot entry comment lines or from the corresponding OMIM entry to the MeSH. Different methods were assessed on a benchmark set of 200 disease names manually mapped to MeSH terms. The performance of the retained procedure in term of precision and recall was 86% and 64% respectively. Using the same procedure, more than 3,000 disease names in Swiss-Prot were mapped to MeSH with comparable efficiency.

**Conclusions:**

This study is a first attempt to link proteins in UniProtKB to the medical resources. The indexing we provided will help clinicians and researchers navigate from diseases to genes and from genes to diseases in an efficient way. The mapping is available at: .

## Background

Biomedical data available to researchers and clinicians have increased drastically over the last decade because of the exponential growth of knowledge in molecular biology. While this has led to the creation of numerous databases and information resources, the interoperability between the resources remains poor to date. One of the main problems lies in the fact that medical terminologies are scarcely used in molecular biology. For instance, while the UniProt Knowledgebase (UniProtKB) - the most comprehensive protein warehouse with extensive cross-references to other database resources [1] – contains more than 2,000 human proteins with manually curated information related to their involvement in pathologies, this information is not easily accessible for clinical researchers. This is due to the fact that UniProtKB does not use standard medical vocabularies to describe diseases associated to proteins and their variants.

In order to increase the interoperability between the biomolecular and clinical resources, one of the key solutions lies in the development or unification of common terminologies capable of acting as a metadata layer to provide the missing links between the various resources. In the medical/clinical domain, there have already been numerous and successful efforts to implement controlled vocabularies for pathologies. Terminologies such as MeSH - the controlled vocabulary thesaurus used for biomedical and health-related documents indexing [2], ICD-10 - the official disease classification provided by the World Health Organisation (WHO) for diagnostic information [3], and SNOMED-CT – the clinical terminology used for clinical information [4], have all served well in their respective domain of application. Most of these terminologies are collected and organised into concepts in the UMLS, a major repository of biomedical standard terminologies [5].

The recent integration of the Gene Ontology (GO) [6] into the UMLS, as well as the development of numerous biological ontologies under the Open Biological Ontologies initiative (OBO) [7], have opened new ways of linking biological and medical resources via terminologies. Therefore, terminology and ontology mapping has become an active field of research, the objective being identifying correspondence between concepts of different resources. The National Library of Medicine (NLM) made an important pioneer effort through the integration of more than 60 medical vocabularies in the UMLS Metathesaurus and the development of lexical tools for this purpose [8]. In parallel, many approaches have been developed which integrate lexically-based, as well as knowledge- and semantics-based methods to map, for instance, GO terms to UMLS concepts [9,10], representations of anatomy [11], genotypic and phenotypic data [12,13]. In the biological field, identical initiatives are emerging for linking OBO ontologies [14]. It was shown that the mapping could be improved by a combination of lexical alignments and hybrid mapping techniques which integrate structural properties of the ontologies. The most advanced tools for aligning and merging ontologies indeed take advantage of both the similarity between terms and the structural features of the resources.

In this study, we tested different automatic approaches to map the disease terms in UniProtKB to MeSH. The MeSH thesaurus is the NLM's controlled vocabulary for subject indexing in MEDLINE [2]. It is structured in a hierarchy of descriptors, with each descriptor including a set of concepts, and each concept itself containing a set of terms, which are synonyms and lexical variants. This rich vocabulary is included in the UMLS and, therefore, is linked to many other biomedical terminologies. The mapping procedures described below took advantage of the manual annotation in UniProtKB as well as the curated links of UniProtKB entries to OMIM, a comprehensive knowledge base of human genes and genetic diseases [15]. A benchmark set was created for the evaluation and refinement of term matching algorithms.

## Results

### Overview of the mapping procedure

**Figure 1:**
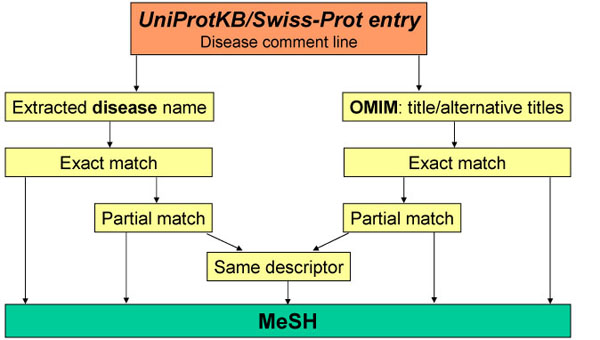
Procedure of the mapping of UniProtKB/Swiss-Prot disease comment lines to MeSH terms.

We mapped the disease names extracted from Swiss-Prot annotations to terms from the disease category of the MeSH terminology. The complete procedure is summarised in Fig. 1. It consisted of three successive steps:

(1) we extracted the disease names from the Swiss-Prot and OMIM entries;

(2) for each disease name, we looked for an exact match with a MeSH term where all words composing the name had an identical correspondent in a MeSH term and vice versa;

(3) when the previous step failed, we looked for partial matches by decomposing the name into its word components and calculate a similarity score with MeSH terms.

To define the whole procedure, a benchmark set was created for the evaluation and refinement of term matching algorithms. Different methods adapted from textual information retrieval techniques were tested. Namely, we evaluated the effect of linguistic pre-processing of the terms to get rid of word lexical variations (with/without normalisation). A method developed by Ha-Thuc and Srinivasan for gene name recognition was also tested [18].

The methods were assessed in term of *retrieval*, *recall* and *precision*, which measure the proportion of terms mapped among all terms, the proportion of terms correctly mapped among all terms, and the proportion of terms correctly mapped among mapped terms, respectively. A detailed description of the methodology is provided in the Methods section.

### The benchmark set

We constructed a benchmark set consisting of 200 randomly selected diseases manually mapped to one or several MeSH terms. The principal problem encountered in this manual mapping process was the lack of specificity of MeSH in the field of genetic diseases. This means that only a quarter of the disease names (52) were mapped to a term of similar meaning. For the other 148 ones, we mapped to a term with coarser granularity and, for 90 of them, we had to choose more than one parent term since the same term could belong to several branches in the MeSH hierarchy. For instance, the disease name *X-linked congenital idiopathic intestinal pseudoobstruction* (P21333) was associated to the MeSH term *Intestinal Pseudo-Obstruction*. However, this term is in no way linked to a branch indicating the genetic origin of the disease. Therefore, we mapped the disease to two other coarser terms belonging to other hierarchies: *Genetic Disease*, *X-Linked* and *Digestive System Abnormalities*.

The manually mapped terms were used to evaluate the performance of automatic procedures described below.

### Disease name extraction

**Figure 2:**
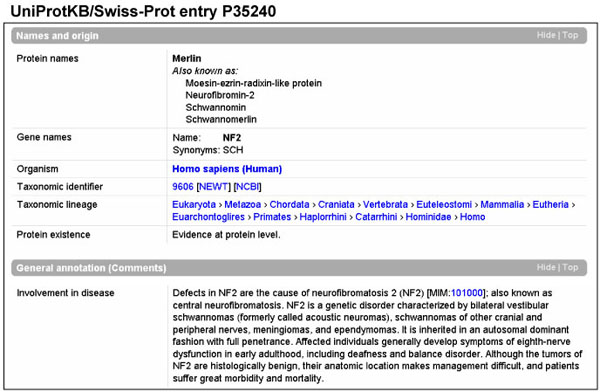
Disease comment lines in a UniProtKB/Swiss-Prot entry.

In Swiss-Prot, the manually annotated section of UniProtKB (release 54.1), 2,252 human protein entries contained information on the involvement of these proteins in a total of 3,408 diseases, mainly of genetic causes (Fig. 2). We extracted almost all disease names from the UniProtKB/Swiss-Prot free text comment lines with a set of regular expressions. The extraction failed in only 7 comment lines where a clear reference to a disease was not expressed, for instance:

“(CBL) can be converted to an oncogenic protein by deletions or mutations that disturb its ability to down-regulate RTKs.” (P22681)

By manually assessing the extraction results, we noticed that as the system was constructed to extract only a single disease name per line, it was unable to treat lines such as:

“KRT16 and KRT17 are coexpressed only in pathological situations such as metaplasias and carcinomas of the uterine cervix and in psoriasis vulgaris.” (P08779)

We did not investigate further these cases, as the structure of disease lines is scheduled for revision as part of Swiss-Prot annotation standardization efforts.

In parallel, we extracted disease names and synonyms from the 2,087 OMIM phenotypes (#) and genes with phenotypes (+) entries cited in the 2,601 Swiss-Prot disease lines. This corresponded to 82% of the total OMIM entries on phenotypes with a known molecular basis (v. August 2007).

### Establishing the mapping procedure using the benchmark set

The 200 disease names of the benchmark set and their associated OMIM terms were automatically mapped to the “Diseases” and “Psychiatry and Psychology” categories of the MeSH (v. August 2007). This subset of MeSH consists of 43,220 different terms. The automatic mapping procedure was done independently on disease names from Swiss-Prot and from OMIM. Different techniques were evaluated to maximize the number of exact and partial term matches.

#### Exact matches

Briefly, the step consisted of transforming all terms into *bag of words* either with or without word normalisation. The word normalisation step was performed using the *Norm* program of the NLM [16]. The effect of term pre-processing was found to be not significant on this dataset, the two procedures giving exactly the same results (Table 1, columns 1-3). All exact matches provided by Swiss-Prot disease names were correct. It was found that the coverage obtained using OMIM terms was better. This could be explained by the presence of synonyms for each disease, which increased matching opportunities. The presence of synonyms however also augmented the risk of possible incorrect mappings. Indeed, the only three false positive matches were caused by a difference of classification between MeSH and OMIM. For instance, two types of *epidermolysis bullosa*, which are distinct MeSH descriptors, are synonyms in OMIM. When we gathered the exact matches provided by Swiss-Prot and OMIM, the recall increased to 26%, with a precision of 96%. It should be noted that the overlap of disease mapping from the two resources did not necessarily mean that the matching terms were the same, but rather that they belonged to the same descriptor in the MeSH terminology.

#### Partial matches

The disease names not mapped by exact matches went through a partial matching procedure. For this, three separate procedures were tested in order to evaluate the effect of term pre-processing as well as the use of different scoring functions:

**Procedure 1:** Term pre-processing followed by calculation of a similarity score for matching terms based on an adaptation of the weighting schema ‘Term Frequency x Inverse Document Frequency’ (TFIDF) [17];

**Procedure 2:** No term pre-processing followed by calculation of the same similarity score as in procedure 1;

**Procedure 3:** Use of the program developed by Ha-Thuc and Srinivasan [18].

The weighting schema TFIDF is commonly used in information retrieval techniques. This scoring method allows evaluate the informative content of a word in a collection or documents. Ha-Thuc and Srinivasan's program uses a different adaptation of TFIDF which allows partial matches at the word level [19,20]. The method also takes advantage of synonymy resources to improve the similarity scoring by increasing the weights or words common to several synonyms.

**Figure 3:**
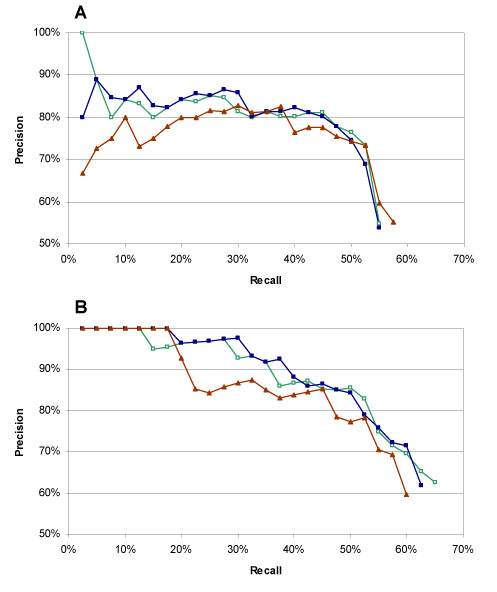
Recall –precision curves for partial matches of Swiss-Prot disease names (A) and OMIM *titles* and *alternative titles* (B) to the disease MeSH terms, with term normalisation (blue squares), without normalisation (green empty squares), and with the method developed by Ha-Thuc (red triangles). The data have been ordered according to the score and the precision is calculated at increasing recall intervals.

The three procedures were evaluated in terms of trade-off between recall and precision (Fig. 3). As already noticed with exact matches, the global performance was better with OMIM terms rather than with Swiss-Prot disease names. This is because of the richer terminology used to define OMIM phenotypes. Likewise, we did not observe significant differences due to term pre-processing. This lack of effect could be explained by the fact that the MeSH vocabulary already includes lexical and orthographic variants, therefore reducing the utility of term normalization.

The performance of the Ha-Thuc's synonym-based similarity scoring was slightly lower than the simpler scoring system we developed. This could be due to the fact that their program calculated a vector similarity measure using the *cosine coefficient*. Indeed, in a first attempt to set up a scoring schema, we noticed that the *cosine coefficient* was less effective on our data. It appears therefore that this similarity measure, although widely used in information retrieval from texts, is less efficient for terminology mapping.

Based on these evaluations, we decided to set up the complete mapping procedure using the scoring method we developed. The word normalisation pre-treatment was included in the procedure even though it did not result in a real gain of performance. The reason for this choice was due to our intention to map Swiss-Prot diseases to ICD-10, which does not include lexical resources. Therefore, a word normalization step could be essential.

**Figure 4:**
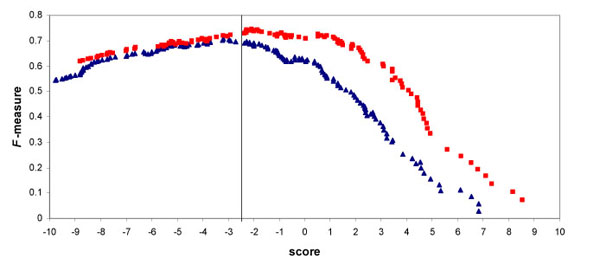
*F*-measure in function of the score of partial matching to MeSH terms with Swiss-Prot disease names (blue triangles) or OMIM terms (red squares).

With the choice of the scoring schema, we proceeded to select a similarity score threshold above which a partial mapping could be considered as correct. The threshold was selected by determining the maximal performance of the system estimated with the *F*- measure, which is the weighted harmonic average of precision and recall (Fig. 4). As the prerequisite for a fully automatic mapping process was high precision, the *F*-measure was parameterized accordingly. We chose a score threshold of -2.5 around which maxima of *F*-measure were found for both OMIM and Swiss-Prot mappings.

The overall system performance was assessed using this threshold for partial matches of the benchmark dataset (Table 1, columns 4-6). It was found that when combining exact and partial matches of Swiss-Prot disease names and OMIM terms, a recall of 64% for a precision of 86% were obtained (Table 1, columns 7-9). While this precision is clearly sufficient to aid manual curation, we could further improve the mapping procedure in terms of precision. For this purpose, we took advantage of the independence of mappings from Swiss-Prot and OMIM, and included an additional condition: the respective mappings should point to the same MeSH descriptor in case of partial matches. Under this condition, and keeping the union of exact matches, the precision increase to 92%, with a drop in recall to 51.5%. This means that more than the half of the benchmark disease names can be mapped to MeSH with a precision above 90%. This value could be considered as sufficient to completely automate the mapping procedure.

**Table 1:** Evaluation of the mapping of 200 UniProtKB/Swiss-Prot disease lines (173 with a reference to OMIM)

	**Exact match**			**Partial match**			**Total**		

	*Retrieval*	*Recall*	*Precision*	*Retrieval*	*Recall*	*Precision*	*Retrieval*	*Recall*	*Precision*
**SP**	35(17.5%)	35(17.5%)	100.0%	91(45.5%)	73(36.5%)	80.0%	126(63%)	108(54%)	86.0%
**OMIM**	43(21.5%)	40(20%)	93.0%	84(42%)	68(34%)	81.0%	127(63.5%)	108(54%)	85.0%
**SP ∩ OMIM**	23(11.5%)	23(11.5%)	100.0%	58(29%)	51(25.5%)	88.0%	93(46.5%)	86(43%)	92.5%
**SP ∪ OMIM**	54(27%)	52(26%)	96.5%	95(47.5%)	76(38%)	80.0%	149(74.5%)	128(64%)	86.0%

SP: UniProtKB/Swiss-Prot

SP ∩ OMIM: both mappings correspond to the same MeSH descriptor.

The mappings of the benchmark, both manual and automatic, are available in additional file 1.

### Automatic mapping of UniprotKB/Swiss-Prot disease comment lines

The mapping procedure was used to map the 3,408 disease comment lines present in UniProtKB/Swiss-Prot. About 76% of them had a corresponding OMIM entry. The results of the mapping are presented in Table 2 (see additional file 2 for the detailed results). Following the safe combination method described previously, we obtained a global performance of 1613 mapped terms, representing 47% of the total number of disease comment lines. The decrease in mapping coverage with OMIM terms (53% compared to 63% of the benchmark) can be explained by the higher proportion of lines having an OMIM citation in the benchmark (87%). Of course, the precision of the mapping cannot be assessed, and the results are expressed in terms of retrieval instead of recall. However, as the figures above do not differ significantly from the benchmark, it is likely that the performance is comparable.

**Table 2:** Mapping on MeSH of the 3408 UniProtKB/Swiss-Prot disease lines (2601 with a corresponding OMIM entry)

	**Exact match**	**Partial match**	**Total**
**SP**	637 (18.7%)	1332 (39%)	1969 (57.8%)
**OMIM**	745 (21.9%)	1063 (31.2%)	1808 (53.1%)
**SP ∩ OMIM**	397 (11.6%)	645 (18.9%)	1289 (37.8%)
**SP ∪ OMIM**	968 (28.4%)	1362 (40%)	2330 (68.4%)

SP: UniProtKB/Swiss-Prot

SP ∩ OMIM: both mappings correspond to the same MeSH descriptor.

As a first assessment, we checked if, in case of exact matches, corresponding Swiss-Prot and OMIM terms mapped to identical MeSH descriptors. This statement was confirmed in all but 17 cases. These discrepancies in descriptor matching were mainly due to differences in classification, with OMIM synonyms corresponding to distinct descriptors in MeSH. Another minor cause was the mention of multiple diseases in the UniProtKB/Swiss-Prot comment line. In these cases, the disease name with an OMIM reference was different from the one extracted.

## Discussion

In this study, we designed a mapping procedure to link the UniProtKB/Swiss-Prot human protein entries and the corresponding OMIM entries to the MeSH disease terminology. MeSH was chosen as it is interlinked with many biomedical terminologies within the UMLS. More importantly, its intimate association with literature will provide us with a valuable means for knowledge discovery using data-mining in the future.

To derive an efficient mapping procedure, alternative methods were tested in order to evaluate the effect of term pre-processing and the use of different similarity scoring systems. It was found that these methods did not differ drastically in terms of performance. Clearly, the benchmark dataset used for evaluation could be too small to draw definite conclusions. However, the fact that MeSH includes many lexical and orthographic term variations does provide an explanation for the low benefit obtained from term normalisation. On the other hand, as both MeSH and OMIM have synonym resources, the mapping procedure should have been improved with the Ha-Thuc's method which cleverly takes into account the word frequency in a set of synonyms. It is possible that the parameters used in Ha-Thuc's program, which was initially developed for gene name entity recognition in textual documents, need to be re-adjusted to better suit the purpose of terminology mapping.

The final mapping procedure we set up by combining exact and partial matches of disease names from OMIM and Swiss-Prot was able to provide a high precision mapping for more than half of the total number of disease comment lines in UniProtKB/Swiss-Prot. Although this retrieval could be considered as low for certain applications, it should be noted that stringent conditions were chosen on purpose to provide a high quality fully automated mapping procedure. If manual curation could be solicited, we could accept a reduced precision.

Recently, the same approach was used to map diagnosis-related annotations of tumor tissue microarrays to the NCI thesaurus [25] with better results (a mapping coverage of 86% and an estimated precision of 86%). These differences in performance could be simply explained by the richness of the domain-specific NCI-T vocabulary compared to the MeSH. Indeed, one of the main problems encountered in the mapping process lay in the difference of granularity between the terminologies, with MeSH being relatively coarse-grained for genetic diseases. Therefore, one strategy to increase the performance of the system would be to allow the mapping to less specific concepts. For instance, the system should be able to map the disease name, *pyruvate dehydrogenase e3-binding protein deficiency*, to its correct parent, *pyruvate dehydrogenase complex deficiency disease*, which currently had a similarity score below the threshold value. To achieve this, one can try to improve the word weighting in order to get rid of rare words without disease-related meaning, such as *e3-binding protein* . This can be done by considering either a common English word thesaurus or a greater biomedical resource, such as the whole MEDLINE database, for the word frequency calculation. More sophisticated linguistic methods could also be applied to analyse the syntactic and semantic structure of the term. Finally, it may be worth integrating information from the MeSH terminology structure in the score calculation as such a strategy has been successfully used for categorising OMIM phenotypes using MeSH terms [26].

Apart from the direct mapping strategy, preliminary work was done to evaluate several indirect mapping strategies that exploit the textual information provided by UniProtKB/Swiss-Prot and OMIM. The first method consisted in using a generic categorizer, XMap [21], to associate Swiss-Prot diseases comment lines with a ranked set of MeSH descriptors. The preliminary results on the benchmark were not convincing (data not shown). This is in agreement with other studies using MetaMap – a similar program developed by the NLM [22] - which reported that these complex methods did not outperform simpler heuristics such as ours in categorising structured database annotations [23,24]. Nevertheless, the method could be more efficient on longer texts such as the OMIM disease *description* fields.

The second method consisted in using the textual information from the biomedical literature cited in Swiss-Prot and OMIM. Indeed MeSH is used to index MEDLINE documents and this information can be used to find the correct term. In a preliminary attempt, all disease MeSH terms in OMIM's citations were extracted and ranked according to their frequency. The precision for the first ranked terms was found to be 57%. The result was rather promising given the fact that the method was not based on term similarity. In future developments, we may consider using this complementary method in combination with the direct mapping.

Nevertheless, the problem of MeSH granularity will hardly be completely solved by these methods. We need definitely to explore the use of other medical terminology resources, such as ICD-10 or SNOMED-CT.

## Conclusions

In conclusion, this work represents the first step in standardizing the medical vocabularies in the UniProt Knowledgebase. Through this effort, we provide a bridge for the medical informatics community to explore the genomic and proteomic data present in biological databases which could be of value for disease understanding.

## Methods

### Extraction of disease names

In UniProtKB/Swiss-Prot, disease information related to a protein entry is expressed in free text comment lines (category ‘Involvement in disease’). We proceeded by first manually establishing a list of regular expressions that indicated the presence of disease names within a Swiss-Prot comment line such as ‘cause(s)’, ‘cause of’, ‘involved in’, ‘contribute(s) to’. The expressions are listed in the additional file 3. The extraction of complete disease names was relatively easy as they are usually located at the end of a sentence or before a conjunction or a relative clause or directly followed by a corresponding OMIM identifier.

In parallel, the fields *Title* and *Alternative titles; symbols* were extracted from the cited OMIM entries. These two fields provide the disease names in OMIM as well as a set of synonyms. For names coming from “gene and phenotype (+)” entries, both gene names and diseases names were included in the disease list.

### Term pre-processing

The mapping procedure was tested with and without word normalisation. The word normalisation was done using the program *Norm* from the lexical tools provided by the NLM [16]. *Norm* removes stop words and plural forms, uninflects verbs, lowercases words etc. For the mapping without word normalisation, we simply lowercased the term components, removed punctuation signs and unspecific words such as “susceptibility to”, “development of” from the disease names extracted from Swiss-Prot (see additional file 3). The word “included” which qualifies a synonym of closely related meaning was also removed from OMIM *Alternative titles*. The terms were transformed into “bags of words”, without taking collocations into account, except for hyphenated words.

### Mapping procedures

The extracted disease names were mapped to the MeSH terms in two successive term matching steps (Fig. 1). First, we looked for exact matches, where all words composing the name had an identical correspondent in a MeSH term and vice versa. The word order and the case were not taken in consideration. When this step failed, we looked for partial matches by calculating a similarity score which is a function of the number of words in common minus the number of words which differ. The similarity score was calculated according to the following formula:

$$S=\frac{\sum_{cw}\log_{2}\left(\frac{1}{freq\left(cw\right)}\right)-\sum_{ncw}\log_{2}\left(\frac{1}{freq\left(ncw\right)}\right)}{size\left(disease\right)}$$

Where *freq=n/N*, with n the number of occurrence of the word in all OMIM (Titles, Alternative titles), MeSH terms (disease category) and Swiss-Prot disease comment lines, and N the total number of words in these documents. *cw* and *ncw* stand for words in common and not in common, respectively, between the two mapped terms, and *size(disease)* is a normalization factor consisting of the number of words composing the disease name to be mapped.

We also calculated term similarity using the program kindly provided by Ha-Thuc and Srinivasan [18]. The implemented procedure uses a ‘soft’ TFIDF approach which introduces a character-based similarity between words [19,20]. In addition, it takes into account the word frequencies in a set of synonym names by increasing the TF scores of words that are common to several synonyms of a disease name.

### Mapping evaluation

In order to evaluate the mapping procedure, 200 disease comment lines from 95 UniProtKB/Swiss-Prot entries were manually mapped to MeSH by a medical expert. Swiss-Prot entries were selected randomly. However, care was taken so that the chosen sample of entries would be representative and lead to a proportion of exact and partial matches similar to that found in a preliminary mapping attempt.

The mapping procedure was assessed in terms of precision, *p=TP/(TP+FP)* and recall, *r=TP/total number of terms*, where *TP* is the number of correct mapping (true positive) and *FP* is the number of incorrect mapping (false positives). Since the system was forced to retain only the best match, we considered, in case of diseases manually mapped to several MeSH terms, that the automatic mapping was correct if at least one of these terms was mapped.

To estimate the performance of the system, the *F*-measure was also calculated according to this formula:

$$F_{\beta }=\left(1+\beta^{2}\right)\frac{pr}{r+\beta^{2}p}$$

The β value was set to 0.5 so as to favor the precision of the mapping.

## Competing interests

The authors declare that there are no competing interests.

## Authors' contributions

AM developed the matching procedure and did the manual mapping. YLY participated in the study's design and helped write the manuscript. PR participated in the study's design. ALV conceived, coordinated the study and wrote the manuscript. All authors read and approved the final manuscript.

## Supplementary Material

Additional file 1 This file contains the manual mapping of 200 Swiss-Prot disease names to Mesh terms, and corresponding automatic mapping with scores. (html format). Click here for file

Additional file 2 This file contains the automatic mapping of all Swiss-Prot disease names with a matching score above the threshold (html format). Click here for file

Additional file 3 This file contains the regular expressions used to extract disease names from the UniProtKB/Swiss-Prot disease comment lines (pdf format). Click here for file

## References

[B1] (2007). The Universal Protein Resource (UniProt). Nucleic Acids Res.

[B2] Nelson SJ, Schopen M, Savage AG, Schulman JL, Arluk N (2004). The MeSH Translation Maintenance System: Structure, Interface Design, and Implementation. Medinfo.

[B3] International Statistical Classification of Diseases and Health Related Problems. (The) ICD-10.

[B4] Donnelly K, SNOMED-CT (2006). The advanced terminology and coding system for eHealth. Stud Health Techno Inform.

[B5] Bodenreider O (2004). The Unified Medical Language System (UMLS): integrating biomedical terminology. Nucleic Acids Res.

[B6] (2006). The Gene Ontology (GO) project in 2006. Nucleic Acids Res.

[B7] Ashburner M, Mungall CJ, Lewis SE (2003). Ontologies for biologists: a community model for the annotation of genomic data. Cold Spring Harbor Symp Quant Biol.

[B8] UMLS Lexical Tools. http://www.nlm.nih.gov/research/umls/tools.html.

[B9] Sarkar IN, Cantor MN, Gelman R, Hartel F, Lussier YA (2003). Linking biomedical language information and knowledge resources: GO and UMLS. Pac Symp Biocomput.

[B10] Cantor MN, Sarkar IN, Gelman R, Hartel F, Bodenreider O, Lussier YA (2003). An evaluation of hybrid methods for matching biomedical terminologies: Mapping the Gene Ontology to the UMLS. Stud Health Technol Inform.

[B11] Zhang S, Mork P, Bodenreider O, Bernstein PA (2007). Comparing two approaches for aligning representations of anatomy. Artif Intell Med.

[B12] Lussier YA, Li J (2004). Terminological mapping for high throughput comparative biology of phenotypes. Pac Symp Biocomput.

[B13] Cantor MN, Sarkar IN, Bodenreider O, Lussier YA (2005). GenesTrace: Phenomic knowledge discovery via structured terminology. Pac Symp Biocomput.

[B14] Johnson HL, Cohen KB, Baumgartner WA, Lu Z, Bada M, Kester T, Kim H, Hunter L (2006). Evaluation of lexical methods for detecting relationships between concepts from multiple ontologies. Pac Symp Biocomput.

[B15] Hamosh A, Scott AF, Amberger JS, Bocchini CA, McKusick VA (2005). Online Mendelian Inheritance in Man (OMIM), a knowledgebase of human genes and genetic disorders. Nucleic Acids Res.

[B16] The Specialist Lexical Tools. http://lexsrv3.nlm.nih.gov/SPECIALIST/index.html.

[B17] Shatkay H (2005). Hairpins in a bookstacks: Information retrieval from biomedical text. Brief Bioinform.

[B18] Ha-Thuc V, Srinivasan P (2007). Exploiting synonym relationships in biomedical named entity matching. BioLINK SIG 2007, ISMB/ECCB.

[B19] Bilenko M, Mooney R, Cohen W, Ravikumar P, Fienberg S (2003). Adaptive name matching in information integration. IEEE Intellig Sys.

[B20] Cohen W, Ravikumar P, Fienberg S (2003). A comparison of string distance metrics. for name-matching tasks. Proc JCCAI Conf.

[B21] Ruch P (2006). Automatic assignment of biomedical categories: toward a generic approach. Bioinformatics.

[B22] Aronson AR (2001). Effective mapping of biomedical text to the UMLS Metathesaurus: the MetaMap program. AMIA Annu SympProc.

[B23] Butte AJ, Kohane IS (2006). Creation and implications of a phenome-genome network. Nat Biotechnol.

[B24] Butte AJ, Chen R (2006). Finding disease-related genomic experiments within an international repository: first steps in translational bioinformatics. AMIA Annu SympProc.

[B25] Shah NH, Rubin DL, Espinosa I, Montgomery K, Musen MA (2007). Annotation and query of tissue microarray data using the NCI Thesaurus. BMC Bioinformatics.

[B26] van Driel MA, Bruggeman J, Vriend G, Brunner HG, Leunissen JA (2006). A text-mining analysis of the human phenome. Eur J Hum Genet.

