# Supplementary material for: Mapping proteins to disease terminologies: from UniProt to MeSH
Source: BMC Bioinformatics. 2008 Apr 29;9(Suppl 5):S3. doi: 10.1186/1471-2105-9-S5-S3 (PMC2367626; doi:10.1186/1471-2105-9-S5-S3)
Supplement: Additional file 1 — This file contains the manual mapping of 200 Swiss-Prot disease names to Mesh terms, and corresponding automatic mapping with scores. (html format). [file 1471-2105-9-S5-S3-S1.htm]

| Mapping of Swiss-Prot disease comment lines to MeSH terms: results on the benchmark | | | |  |  |  |  |  |  |  |
| Colors correspond to a score threshold of -2.5 | | |  |  |  |  |  |  |  |  |
| True positive SP ∩ OMIM |  |  |  |  |  |  |  |  |  |  |
| True Positive SP U OMIM |  |  |  |  |  |  |  |  |  |  |
| False negative |  |  |  |  |  |  |  |  |  |  |
| True negative |  |  |  |  |  |  |  |  |  |  |
| False positive |  |  |  |  |  |  |  |  |  |  |
|  |  |  |  |  |  |  |  |  |  |  |
| Origine | True / False | Score | Disease | Mapped term | DescriptorUI | Correct term | DescriptorUI | AC | cc line | |
| SP | T | 3.1126 | idiopathic generalized epilepsy | epilepsy, generalized | D004829 | Generalized Epilepsy | D004829 | O00305 | 1 |  |
| OMIM | T | 3.1126 | epilepsy, idiopathic generalized | epilepsy, generalized | D004829 | Generalized Epilepsy | D004829 | O00305 | 1 |  |
| SP | T | exact | juvenile myoclonic epilepsy | juvenile myoclonic epilepsy | D020190 | Juvenile Myoclonic Epilepsy | D020190 | O00305 | 2 |  |
| OMIM | T | exact | epilepsy, juvenile myoclonic | epilepsy, juvenile myoclonic | D020190 | Juvenile Myoclonic Epilepsy | D020190 | O00305 | 2 |  |
| SP | T | 5.2698 | torsion dystonia 1 | dystonias, torsion | D004422 | Idiopathic Torsion Dystonia | D004422 | O14656 | 1 |  |
| OMIM | T | 8.5451 | dystonia musculorum deformans 1 | dystonia musculorum deformans | D004422 | Idiopathic Torsion Dystonia | D004422 | O14656 | 1 |  |
| SP | T | 1.2915 | squamous cell carcinoma of the head and neck | carcinoma, squamous cell | D002294 | Squamous Cell Carcinoma | Head and Neck Cancer | D002294 D006258 | O14763 | 1 |  |
| OMIM | T | 1.2915 | squamous cell carcinoma, head and neck | carcinoma, squamous cell | D002294 | Squamous Cell Carcinoma | Head and Neck Cancer | D002294 D006258 | O14763 | 1 |  |
| SP | T | -1.2171 | progressive familial intrahepatic cholestasis type 1 | cholestases, intrahepatic | D002780 | Genetic Disease, Inborn | Intrahepatic Cholestasis | D030342 D002780 | O43520 | 1 |  |
| OMIM | T | 4.4199 | cholestasis, fatal intrahepatic | cholestases, intrahepatic | D002780 | Genetic Disease, Inborn | Intrahepatic Cholestasis | D030342 D002780 | O43520 | 1 |  |
| SP | T | 1.1308 | benign recurrent intrahepatic cholestasis | cholestases, intrahepatic | D002780 | Genetic Disease, Inborn | Intrahepatic Cholestasis | D030342 D002780 | O43520 | 2 |  |
| OMIM | T | -0.7496 | cholestasis, benign recurrent intrahepatic 1 | cholestases, intrahepatic | D002780 | Genetic Disease, Inborn | Intrahepatic Cholestasis | D030342 D002780 | O43520 | 2 |  |
| SP | T | 0.8412 | recurrent intrahepatic cholestasis of pregnancy | cholestases, intrahepatic | D002780 | Intrahepatic Cholestasis | Pregnancy Complications | D002780 D011248 | O43520 | 3 |  |
| OMIM | T | 4.7828 | cholestasis, intrahepatic, of pregnancy | cholestases, intrahepatic | D002780 | Intrahepatic Cholestasis | Pregnancy Complications | D002780 D011248 | O43520 | 3 |  |
| SP | T | 0.6919 | autosomal recessive limb girdle muscular dystrophy type 2b | muscular dystrophies, limb girdle | D049288 | Limb-Girdle Muscular Dystrophy | D049288 | O75923 | 1 |  |
| OMIM | T | 4.205 | muscular dystrophy, limb-girdle, type 3 | muscular dystrophies, limb girdle | D049288 | Limb-Girdle Muscular Dystrophy | D049288 | O75923 | 1 |  |
| SP | T | exact | bladder cancer | bladder cancer | D001749 | Bladder Cancer | D001749 | P01112 | 3 |  |
| OMIM | T | exact | bladder cancer | bladder cancer | D001749 | Bladder Cancer | D001749 | P01112 | 3 |  |
| SP | T | 1.9776 | spondyloepiphyseal dysplasia congenital type | spondyloepiphyseal dysplasias | D010009 | Genetic Disease, Inborn | Abnormalities, Multiple | Spondyloepiphyseal Dysplasia | Dwarfism | D030342 D000015 D010009 D004392 | P02458 | 2 |  |
| OMIM | T | 3.7594 | spondyloepiphyseal dysplasia congenita | spondyloepiphyseal dysplasias | D010009 | Genetic Disease, Inborn | Abnormalities, Multiple | Spondyloepiphyseal Dysplasia | Dwarfism | D030342 D000015 D010009 D004392 | P02458 | 2 |  |
| SP | T | 0.1118 | primary avascular necrosis of femoral head | necrosis, avascular, of femur head | D005271 | Avascular Necrosis of Femur Head | D005271 | P02458 | 6 |  |
| OMIM | T | 2.4825 | femoral head, avascular necrosis of | necrosis, avascular, of femur head | D005271 | Avascular Necrosis of Femur Head | D005271 | P02458 | 6 |  |
| SP | T | -0.8968 | multiple epiphyseal dysplasia with myopia and conductive deafness | dysplasias, multiple epiphyseal | D010009 | Genetic Disease, Inborn | Abnormalities, Multiple | Multiple Epiphyseal Dysplasia | Eye Diseases | Hearing Loss, Conductive | Dwarfism | D030342 D000015 D010009 D005128 D006314 D004392 | P02458 | 9 |  |
| OMIM | T | -0.8968 | epiphyseal dysplasia, multiple, with myopia and conductive deafness | dysplasias, multiple epiphyseal | D010009 | Genetic Disease, Inborn | Abnormalities, Multiple | Multiple Epiphyseal Dysplasia | Eye Diseases | Hearing Loss, Conductive | Dwarfism | D030342 D000015 D010009 D005128 D006314 D004392 | P02458 | 9 |  |
| SP | T | -2.0082 | autosomal dominant rhegmatogenous retinal detachment | detachments, retinal | D012163 | Eye Disease, Hereditary | Retinal Detachment | D015785 D012163 | P02458 | 13 |  |
| OMIM | T | -2.0082 | rhegmatogenous retinal detachment, autosomal dominant | detachments, retinal | D012163 | Eye Disease, Hereditary | Retinal Detachment | D015785 D012163 | P02458 | 13 |  |
| SP | T | -1.4586 | low hdl levels observed in high density lipoprotein deficiency type 1 | high density lipoprotein deficiency, type i | D013631 | Tangier Disease | D013631 | P02647 | 2 |  |
| OMIM | T | exact | tangier disease | tangier disease | D013631 | Tangier Disease | D013631 | P02647 | 2 |  |
| SP | T | exact | congenital insensitivity to pain with anhidrosis | congenital insensitivity to pain with anhidrosis | D009477 | Congenital Insensitivity to Pain with Anhidrosis | D009477 | P04629 | 1 |  |
| OMIM | T | exact | insensitivity to pain, congenital, with anhidrosis | insensitivity to pain with anhidrosis, congenital | D009477 | Congenital Insensitivity to Pain with Anhidrosis | D009477 | P04629 | 1 |  |
| SP | T | 3.4424 | thyroid papillary carcinoma | carcinoma, papillary | D002291 | Thyroid Carcinoma | Papillary Carcinoma | D013964 D002291 | P04629 | 2 |  |
| OMIM | T | 3.4424 | thyroid carcinoma, papillary | carcinoma, papillary | D002291 | Thyroid Carcinoma | Papillary Carcinoma | D013964 D002291 | P04629 | 2 |  |
| SP | T | 3.4424 | thyroid papillary carcinoma | carcinoma, papillary | D002291 | Thyroid Carcinoma | Papillary Carcinoma | D013964 D002291 | P04629 | 3 |  |
| OMIM | T | 3.4424 | thyroid carcinoma, papillary | carcinoma, papillary | D002291 | Thyroid Carcinoma | Papillary Carcinoma | D013964 D002291 | P04629 | 3 |  |
| SP | T | 2.1909 | hypophosphatasia infantile | hypophosphatasias | D007014 | Hypophosphatasia | D007014 | P05186 | 1 |  |
| OMIM | T | 2.1909 | hypophosphatasia, infantile | hypophosphatasias | D007014 | Hypophosphatasia | D007014 | P05186 | 1 |  |
| SP | T | 2.3017 | hypophosphatasia childhood | hypophosphatasias | D007014 | Hypophosphatasia | D007014 | P05186 | 2 |  |
| OMIM | T | 2.3017 | hypophosphatasia, childhood | hypophosphatasias | D007014 | Hypophosphatasia | D007014 | P05186 | 2 |  |
| SP | T | -0.8011 | hypophosphatasia adult type | hypophosphatasias | D007014 | Hypophosphatasia | D007014 | P05186 | 3 |  |
| OMIM | T | 1.9116 | hypophosphatasia, mild | hypophosphatasias | D007014 | Hypophosphatasia | D007014 | P05186 | 3 |  |
| SP | T | 1.9252 | nemaline myopathy type 1 | myopathies, nemaline | D017696 | Childhood Onset Nemaline Myopathy | Autosomal Dominant Nemaline Myopathy | D017696 D017696 | P06753 | 1 |  |
| OMIM | T | 4.825 | nemaline myopathy 1 | myopathies, nemaline | D017696 | Childhood Onset Nemaline Myopathy | Autosomal Dominant Nemaline Myopathy | D017696 D017696 | P06753 | 1 |  |
| SP | T | 3.4424 | thyroid papillary carcinoma | carcinoma, papillary | D002291 | Thyroid Carcinoma | Papillary Carcinoma | D013964 D002291 | P06753 | 2 |  |
| OMIM | T | 3.4424 | thyroid carcinoma, papillary | carcinoma, papillary | D002291 | Thyroid Carcinoma | Papillary Carcinoma | D013964 D002291 | P06753 | 2 |  |
| SP | T | -1.7911 | autosomal dominant cataract | cataract | D002386 | Genetic Disease, Inborn | Cataract | D030342 D002386 | P07315 | 1 |  |
| OMIM | T | -1.7911 | cataract, autosomal dominant | cataract | D002386 | Genetic Disease, Inborn | Cataract | D030342 D002386 | P07315 | 1 |  |
| SP | T | 2.3801 | familial hypertrophic cardiomyopathy type 8 | cardiomyopathies, familial hypertrophic | D024741 | Cardiomyopathy, Hypertrophic, Familial | D024741 | P08590 | 1 |  |
| OMIM | T | exact | cardiomyopathy, familial hypertrophic | cardiomyopathy, familial hypertrophic | D024741 | Cardiomyopathy, Hypertrophic, Familial | D024741 | P08590 | 1 |  |
| SP | T | exact | fructose-1,6- bisphosphatase deficiency | fructose 1,6 bisphosphatase deficiency | D015319 | Fructose-1,6-Bisphosphatase Deficiency | D015319 | P09467 | 1 |  |
| OMIM | T | exact | fructose-1,6-bisphosphatase deficiency | fructose-1,6-bisphosphatase deficiency | D015319 | Fructose-1,6-Bisphosphatase Deficiency | D015319 | P09467 | 1 |  |
| SP | T | exact | pfeiffer syndrome | pfeiffer syndrome | D000168 | Pfeiffer Syndrome | D000168 | P11362 | 1 |  |
| OMIM | T | exact | pfeiffer syndrome | pfeiffer syndrome | D000168 | Pfeiffer Syndrome | D000168 | P11362 | 1 |  |
| SP | T | 4.6839 | isolated hypogonadotropic hypogonadism | hypogonadotropic hypogonadism | D007006 | Hypogonadotropic Hypogonadism | D007006 | P11362 | 2 |  |
| OMIM | T | exact | hypogonadotropic hypogonadism | hypogonadotropic hypogonadism | D007006 | Hypogonadotropic Hypogonadism | D007006 | P11362 | 2 |  |
| SP | T | 3.2191 | kallmann syndrome type 2 | kallmann syndrome 2 | D017436 | Kallmann Syndrome 2 | D017436 | P11362 | 3 |  |
| OMIM | T | exact | kallmann syndrome 2 | kallmann syndrome 2 | D017436 | Kallmann Syndrome 2 | D017436 | P11362 | 3 |  |
| SP | T | 2.9655 | epidermolysis bullosa simplex dowling-meara type | epidermolysis bullosa herpetiformis dowling meara | D016110 | Epidermolysis Bullosa Herpetiformis Dowling-Meara | D016110 | P13647 | 2 |  |
| OMIM | T | 10.39 | epidermolysis bullosa herpetiformis, dowling-meara type | epidermolysis bullosa herpetiformis dowling meara | D016110 | Epidermolysis Bullosa Herpetiformis Dowling-Meara | D016110 | P13647 | 2 |  |
| SP | T | -1.671 | epidermolysis bullosa simplex with migratory circinate erythema | epidermolysis bullosa simplex | D016110 | Epidermolysis Bullosa Simplex | D016110 | P13647 | 3 |  |
| OMIM | T | -1.671 | epidermolysis bullosa simplex with migratory circinate erythema | epidermolysis bullosa simplex | D016110 | Epidermolysis Bullosa Simplex | D016110 | P13647 | 3 |  |
| SP | T | 2.3614 | epidermolysis bullosa simplex koebner type | epidermolysis bullosa simplex | D016110 | Epidermolysis Bullosa Simplex Kobner | D016110 | P13647 | 5 |  |
| OMIM | T | 2.3614 | epidermolysis bullosa simplex, koebner type | epidermolysis bullosa simplex | D016110 | Epidermolysis Bullosa Simplex Kobner | D016110 | P13647 | 5 |  |
| SP | T | 1.4035 | epidermolysis bullosa simplex with mottled pigmentation | epidermolysis bullosa simplex | D016110 | Epidermolysis Bullosa Simplex | D016110 | P13647 | 6 |  |
| OMIM | T | 1.4035 | epidermolysis bullosa simplex with mottled pigmentation | epidermolysis bullosa simplex | D016110 | Epidermolysis Bullosa Simplex | D016110 | P13647 | 6 |  |
| SP | T | 4.5728 | acute hepatic porphyria | porphyria, hepatic | D017094 | hepatic porphyria | D017094 | P13716 | 1 |  |
| OMIM | T | 4.5728 | porphyria, acute hepatic | porphyria, hepatic | D017094 | hepatic porphyria | D017094 | P13716 | 1 |  |
| SP | T | exact | metachromatic leukodystrophy | metachromatic leukodystrophy | D007966 | Metachromatic Leukodystrophy | D007966 | P15289 | 1 |  |
| OMIM | T | exact | metachromatic leukodystrophy | metachromatic leukodystrophy | D007966 | Metachromatic Leukodystrophy | D007966 | P15289 | 1 |  |
| SP | T | exact | multiple sulfatase deficiency | multiple sulfatase deficiency | D052517 | Multiple Sulfatase Deficiency Disease | D052517 | P15289 | 2 |  |
| OMIM | T | exact | multiple sulfatase deficiency | multiple sulfatase deficiency | D052517 | Multiple Sulfatase Deficiency Disease | D052517 | P15289 | 2 |  |
| SP | T | -1.1222 | autosomal dominant nocturnal frontal lobe epilepsy type 3 | frontal lobe epilepsies | D017034 | Genetic Disease, Inborn | Epilepsy, Frontal Lobe | D030342 D017034 | P17787 | 1 |  |
| OMIM | T | 1.184 | epilepsy, nocturnal frontal lobe, type 3 | frontal lobe epilepsies | D017034 | Genetic Disease, Inborn | Epilepsy, Frontal Lobe | D030342 D017034 | P17787 | 1 |  |
| SP | T | -2.1573 | familial erythrocytosis type 1 | erythrocytoses | D011086 | Genetic Disease, Inborn | Erythrocytosis | D030342 D011086 | P19235 | 1 |  |
| OMIM | T | -0.6183 | erythrocytosis, familial, 1 | erythrocytoses | D011086 | Genetic Disease, Inborn | Erythrocytosis | D030342 D011086 | P19235 | 1 |  |
| SP | T | exact | melnick-needles syndrome | melnick-needles syndrome | D010009 | Melnick-Needles Syndrome | D010009 | P21333 | 6 |  |
| OMIM | T | exact | melnick-needles syndrome | melnick-needles syndrome | D010009 | Melnick-Needles Syndrome | D010009 | P21333 | 6 |  |
| SP | T | 2.6771 | x-linked congenital idiopathic intestinal pseudoobstruction | idiopathic intestinal pseudo-obstructions | D007418 | Genetic Disease, X-Linked | Digestive System Abnormalities | Intestinal Pseudo-Obstruction | D040181 D004065 D007418 | P21333 | 8 |  |
| OMIM | T | 7.3371 | congenital idiopathic intestinal pseudoobstruction | idiopathic intestinal pseudo-obstructions | D007418 | Genetic Disease, X-Linked | Digestive System Abnormalities | Intestinal Pseudo-Obstruction | D040181 D004065 D007418 | P21333 | 8 |  |
| SP | T | 2.3769 | autosomal recessive lamellar ichthyosis | ichthyosis, lamellar | D017490 | Lamellar Ichthyosis | D017490 | P22735 | 1 |  |
| OMIM | T | exact | lamellar ichthyosis | lamellar ichthyosis | D017490 | Lamellar Ichthyosis | D017490 | P22735 | 1 |  |
| SP | T | 0.678 | waardenburg syndrome type i | waardenburg syndrome | D014849 | Waardenburg's Syndrome | D014849 | P23760 | 1 |  |
| OMIM | T | 0.678 | waardenburg syndrome, type i | waardenburg syndrome | D014849 | Waardenburg's Syndrome | D014849 | P23760 | 1 |  |
| SP | T | 0.3996 | waardenburg syndrome type iii | waardenburg syndrome | D014849 | Waardenburg-Klein Syndrome | D014849 | P23760 | 2 |  |
| OMIM | T | exact | klein-waardenburg syndrome | klein-waardenburg syndrome | D014849 | Waardenburg-Klein Syndrome | D014849 | P23760 | 2 |  |
| SP | T | 6.8316 | fuchs endothelial corneal dystrophy | fuchs endothelial dystrophy | D005642 | Fuchs Endothelial Dystrophy | D005642 | P25067 | 2 |  |
| OMIM | T | 3.8111 | corneal dystrophy, fuchs endothelial, 1 | fuchs endothelial dystrophy | D005642 | Fuchs Endothelial Dystrophy | D005642 | P25067 | 2 |  |
| SP | T | -0.3954 | adrenal hyperplasia type 2 | adrenal hyperplasias, congenital | D000312 | Congenital Adrenal Hyperplasia | D000312 | P26439 | 1 |  |
| OMIM | T | 1.5101 | adrenal hyperplasia ii | adrenal hyperplasias, congenital | D000312 | Congenital Adrenal Hyperplasia | D000312 | P26439 | 1 |  |
| SP | T | 6.8255 | congenital pulmonary alveolar proteinosis | alveolar proteinosis, pulmonary | D011649 | Genetic Disease, Inborn | Pulmonary Alveolar Proteinosis | D030342 D011649 | P32927 | 1 |  |
| OMIM | T | -1.8404 | pulmonary alveolar proteinosis due to surfactant protein b deficiency | alveolar proteinosis, pulmonary | D011649 | Genetic Disease, Inborn | Pulmonary Alveolar Proteinosis | D030342 D011649 | P32927 | 1 |  |
| SP | T | exact | pilomatrixoma | pilomatrixoma | D018296 | Pilomatrixoma | D018296 | P35222 | 2 |  |
| OMIM | T | exact | pilomatrixoma | pilomatrixoma | D018296 | Pilomatrixoma | D018296 | P35222 | 2 |  |
| SP | T | exact | medulloblastoma | medulloblastoma | D008527 | Medulloblastoma | D008527 | P35222 | 3 |  |
| OMIM | T | exact | medulloblastoma | medulloblastoma | D008527 | Medulloblastoma | D008527 | P35222 | 3 |  |
| SP | T | -0.2049 | paramyotonia congenita of von eulenburg | congenita, paramyotonia | D020967 | Eulenburg Disease | D020967 | P35499 | 1 |  |
| OMIM | T | -0.2049 | paramyotonia congenita of von eulenburg | congenita, paramyotonia | D020967 | Eulenburg Disease | D020967 | P35499 | 1 |  |
| SP | T | exact | hypokalemic periodic paralysis | hypokalemic periodic paralysis | D020514 | Hypokalemic Periodic Paralysis | D020514 | P35499 | 2 |  |
| OMIM | T | exact | hypokalemic periodic paralysis | hypokalemic periodic paralysis | D020514 | Hypokalemic Periodic Paralysis | D020514 | P35499 | 2 |  |
| SP | T | exact | hyperkalemic periodic paralysis | hyperkalemic periodic paralysis | D020513 | Hyperkalemic Periodic Paralysis | D020513 | P35499 | 3 |  |
| OMIM | T | exact | hyperkalemic periodic paralysis | hyperkalemic periodic paralysis | D020513 | Hyperkalemic Periodic Paralysis | D020513 | P35499 | 3 |  |
| SP | T | exact | marfan syndrome | marfan syndrome | D008382 | Marfan Syndrome | D008382 | P35555 | 1 |  |
| OMIM | T | exact | marfan syndrome | marfan syndrome | D008382 | Marfan Syndrome | D008382 | P35555 | 1 |  |
| SP | T | 6.1018 | isolated ectopia lentis | ectopia lentis | D004479 | Genetic Disease, Inborn | Ectopia Lentis | D030342 D004479 | P35555 | 2 |  |
| OMIM | T | 7.1052 | ectopia lentis, familial | ectopia lentis | D004479 | Genetic Disease, Inborn | Ectopia Lentis | D030342 D004479 | P35555 | 2 |  |
| SP | T | exact | hereditary coproporphyria | hereditary coproporphyria | D046349 | Hereditary Coproporphyria | D046349 | P36551 | 1 |  |
| OMIM | T | exact | coproporphyrinogen oxidase deficiency | coproporphyrinogen oxidase deficiency | D046349 | Hereditary Coproporphyria | D046349 | P36551 | 1 |  |
| SP | T | exact | cowden disease | cowden disease | D006223 | Cowden Disease | D006223 | P36894 | 2 |  |
| OMIM | T | exact | cowden disease | cowden disease | D006223 | Cowden Disease | D006223 | P36894 | 2 |  |
| SP | T | 2.7763 | x-linked alpha- thalassemia/mental retardation syndrome | x-linked mental retardation syndromes | D038901 | Mental Retardation, X-Linked | alpha-Thalassemia | Abnormalities, Multiple | Urogenital Abnormalities | Craniofacial Abnormalities | D038901 D017085 D000015 D014564 D019465 | P46100 | 1 |  |
| OMIM | T | -0.5131 | alpha-thalassemia/mental retardation syndrome, nondeletion type, x-linked | x-linked mental retardation syndromes | D038901 | Mental Retardation, X-Linked | alpha-Thalassemia | Abnormalities, Multiple | Urogenital Abnormalities | Craniofacial Abnormalities | D038901 D017085 D000015 D014564 D019465 | P46100 | 1 |  |
| SP | T | -0.0746 | mental retardation x- linked with hypotonic facies syndrome type 1 | x-linked mental retardation syndromes | D038901 | Mental Retardation, X-Linked | Abnormalities, Multiple | Craniofacial Abnormalities | D038901 D000015 D019465 | P46100 | 2 |  |
| OMIM | T | 0.8827 | mental retardation, x-linked, with growth retardation, deafness, and microgenitalism | mental retardation, x linked | D038901 | Mental Retardation, X-Linked | Abnormalities, Multiple | Craniofacial Abnormalities | D038901 D000015 D019465 | P46100 | 2 |  |
| SP | T | 0.5159 | alpha-thalassemia myelodysplasia syndrome | alpha-thalassemia | D017085 | alpha-Thalassemia | Hematologic Diseases | Genetic Diseases, X-Linked | D017085 D006402 D040181 | P46100 | 3 |  |
| OMIM | T | 2.1204 | hemoglobin h disease, acquired | hemoglobin h diseases | D017085 | alpha-Thalassemia | Hematologic Diseases | Genetic Diseases, X-Linked | D017085 D006402 D040181 | P46100 | 3 |  |
| SP | T | 1.8158 | familial early-onset alzheimer disease type 3 | early onset alzheimer disease | D000544 | Early Onset Alzheimer Disease | Genetic Disease, Inborn | D000544 D030342 | P49768 | 1 |  |
| OMIM | T | 5.6049 | alzheimer disease 3, early-onset | early onset alzheimer disease | D000544 | Early Onset Alzheimer Disease | Genetic Disease, Inborn | D000544 D030342 | P49768 | 1 |  |
| SP | T | 6.5376 | familial hemiplegic migraine 2 | familial hemiplegic migraines | D020325 | Hemiplegic Migraine, Familial | D020325 | P50993 | 1 |  |
| OMIM | T | 6.5376 | migraine, familial hemiplegic, 2 | familial hemiplegic migraines | D020325 | Hemiplegic Migraine, Familial | D020325 | P50993 | 1 |  |
| SP | T | 2.0627 | long qt syndrome type 1 | long qt syndrome 1 | D029597 | Long QT Syndrome 1 | D029597 | P51787 | 1 |  |
| OMIM | T | exact | long qt syndrome 1 | long qt syndrome 1 | D029597 | Long QT Syndrome 1 | D029597 | P51787 | 1 |  |
| SP | T | exact | jervell and lange-nielsen syndrome | jervell and lange-nielsen syndrome | D029593 | Jervell and Lange-Nielsen Syndrome | D029593 | P51787 | 2 |  |
| OMIM | T | exact | jervell and lange-nielsen syndrome | jervell and lange-nielsen syndrome | D029593 | Jervell and Lange-Nielsen Syndrome | D029593 | P51787 | 2 |  |
| SP | T | exact | atrial fibrillation | atrial fibrillation | D001281 | Genetic Disease, Inborn | Atrial Fibrillation | D030342 D001281 | P51787 | 3 |  |
| OMIM | T | 2.2346 | atrial fibrillation, autosomal dominant | fibrillations, atrial | D001281 | Genetic Disease, Inborn | Atrial Fibrillation | D030342 D001281 | P51787 | 3 |  |
| SP | T | 0.8697 | bartter syndrome type 3 | syndrome, bartter | D001477 | Bartter Syndrome | D001477 | P51801 | 1 |  |
| OMIM | T | 2.3043 | bartter syndrome, classic | syndrome, bartter | D001477 | Bartter Syndrome | D001477 | P51801 | 1 |  |
| SP | T | -2.0166 | hemochromatosis type 2b | hemochromatoses | D006432 | Hemochromatosis | D006432 | P81172 | 1 |  |
| OMIM | T | 1.8331 | hemochromatosis, juvenile | hemochromatoses | D006432 | Hemochromatosis | D006432 | P81172 | 1 |  |
| SP | T | exact | pachyonychia congenita type 2 | type 2 pachyonychia congenita | D053549 | Pachyonychia Congenita, Type 2 | D053549 | Q04695 | 1 |  |
| OMIM | T | exact | pachyonychia congenita, type 2 | pachyonychia congenita, type 2 | D053549 | Pachyonychia Congenita, Type 2 | D053549 | Q04695 | 1 |  |
| SP | T | exact | thyroid dysgenesis | thyroid dysgenesis | D050033 | Thyroid Dysgenesis | D050033 | Q06710 | 1 |  |
| OMIM | T | exact | thyroid dysgenesis | thyroid dysgenesis | D050033 | Thyroid Dysgenesis | D050033 | Q06710 | 1 |  |
| SP | T | exact | zellweger syndrome | zellweger syndrome | D015211 | Zellweger Syndrome | D015211 | Q13608 | 2 |  |
| OMIM | T | exact | zellweger syndrome | zellweger syndrome | D015211 | Zellweger Syndrome | D015211 | Q13608 | 2 |  |
| SP | T | exact | hypokalemic periodic paralysis | hypokalemic periodic paralysis | D020514 | Hypokalemic Periodic Paralysis | D020514 | Q13698 | 1 |  |
| OMIM | T | exact | hypokalemic periodic paralysis | hypokalemic periodic paralysis | D020514 | Hypokalemic Periodic Paralysis | D020514 | Q13698 | 1 |  |
| SP | T | 1.124 | malignant hyperthermia susceptibility 5 | malignant hyperthermias | D008305 | Malignant Hyperthermia | Genetic Predisposition to Disease | D008305 D020022 | Q13698 | 2 |  |
| OMIM | T | 1.124 | malignant hyperthermia, susceptibility to, 5 | malignant hyperthermias | D008305 | Malignant Hyperthermia | Genetic Predisposition to Disease | D008305 D020022 | Q13698 | 2 |  |
| SP | T | exact | cleidocranial dysplasia | cleidocranial dysplasia | D002973 | Cleidocranial Dysplasia | D002973 | Q13950 | 1 |  |
| OMIM | T | exact | cleidocranial dysplasia | cleidocranial dysplasia | D002973 | Cleidocranial Dysplasia | D002973 | Q13950 | 1 |  |
| SP | T | 1.3122 | ocular coloboma | colobomas | D003103 | Coloboma | D003103 | Q15465 | 1 |  |
| OMIM | T | 1.3122 | coloboma, ocular | colobomas | D003103 | Coloboma | D003103 | Q15465 | 1 |  |
| SP | T | -1.0034 | holoprosencephaly type 3 | holoprosencephalies | D016142 | Holoprosencephaly | D016142 | Q15465 | 2 |  |
| OMIM | T | 1.882 | holoprosencephaly 3 | holoprosencephalies | D016142 | Holoprosencephaly | D016142 | Q15465 | 2 |  |
| SP | T | 3.2182 | hereditary multiple exostoses type 1 | exostosis, hereditary multiple | D005097 | Hereditary Multiple Exostoses | D005097 | Q16394 | 1 |  |
| OMIM | T | exact | multiple cartilaginous exostoses | multiple cartilaginous exostoses | D005097 | Hereditary Multiple Exostoses | D005097 | Q16394 | 1 |  |
| SP | T | exact | chondrosarcoma | chondrosarcoma | D002813 | Chondrosarcoma | D002813 | Q16394 | 3 |  |
| OMIM | T | exact | chondrosarcoma | chondrosarcoma | D002813 | Chondrosarcoma | D002813 | Q16394 | 3 |  |
| SP | T | exact | dyslexia | dyslexia | D004410 | Dyslexia | Genetic Predisposition to Disease | D004410 D020022 | Q5VV43 | 1 |  |
| OMIM | T | -1.3579 | reading disability, specific, 2 | developmental reading disabilities | D004410 | Dyslexia | Genetic Predisposition to Disease | D004410 D020022 | Q5VV43 | 1 |  |
| SP | T | -0.7092 | autosomal recessive osteopetrosis | osteopetrosis | D010022 | Osteopetrosis | Genetic Disease, Inborn | D010022 D030342 | Q86WC4 | 1 |  |
| OMIM | T | 4.0659 | albers-schonberg disease, autosomal recessive | disease, albers-schoenberg | D010022 | Osteopetrosis | Genetic Disease, Inborn | D010022 D030342 | Q86WC4 | 1 |  |
| SP | T | 2.7339 | bardet-biedl syndrome type 10 | bardet-biedl syndrome | D020788 | Bardet-Biedl Syndrome | D020788 | Q8TAM1 | 1 |  |
| OMIM | T | exact | bardet-biedl syndrome | bardet-biedl syndrome | D020788 | Bardet-Biedl Syndrome | D020788 | Q8TAM1 | 1 |  |
| SP | T | 0.7775 | primary open angle glaucoma type 1e | glaucoma, open-angle | D005902 | Open Angle Glaucoma | D005902 | Q96CV9 | 1 |  |
| OMIM | T | 6.7831 | glaucoma, primary open angle | glaucoma, open-angle | D005902 | Open Angle Glaucoma | D005902 | Q96CV9 | 1 |  |
| SP | T | -1.4367 | severe combined immunodeficiency with sensitivity to ionizing radiation | immunodeficiencies, severe combined | D016511 | Severe Combined Immunodeficiency | D016511 | Q96SD1 | 1 |  |
| OMIM | T | 4.9303 | severe combined immunodeficiency, partial | immunodeficiencies, severe combined | D016511 | Severe Combined Immunodeficiency | D016511 | Q96SD1 | 1 |  |
| SP | T | -1.5294 | athabascan scid | scid | D016511 | Severe Combined Immunodeficiency | D016511 | Q96SD1 | 2 |  |
| OMIM | T | 4.9303 | severe combined immunodeficiency, partial | immunodeficiencies, severe combined | D016511 | Severe Combined Immunodeficiency | D016511 | Q96SD1 | 2 |  |
| SP | T | 4.9303 | partial severe combined immunodeficiency | immunodeficiencies, severe combined | D016511 | Severe Combined Immunodeficiency | D016511 | Q96SD1 | 3 |  |
| OMIM | T | 4.9303 | severe combined immunodeficiency, partial | immunodeficiencies, severe combined | D016511 | Severe Combined Immunodeficiency | D016511 | Q96SD1 | 3 |  |
| SP | T | exact | macular corneal dystrophy | macular dystrophy, corneal | D003317 | Macular Dystrophy, Corneal | D003317 | Q9GZX3 | 1 |  |
| OMIM | T | 6.1515 | corneal dystrophy, macular type | macular dystrophy, corneal | D003317 | Macular Dystrophy, Corneal | D003317 | Q9GZX3 | 1 |  |
| SP | T | -2.2768 | congenital muscular dystrophy type 1c | dystrophies, muscular | D009136 | Muscular Dystrophy | D009136 | Q9H9S5 | 1 |  |
| OMIM | T | -1.1525 | muscular dystrophy, congenital, 1c | dystrophies, muscular | D009136 | Muscular Dystrophy | D009136 | Q9H9S5 | 1 |  |
| SP | T | 3.0717 | limb-girdle muscular dystrophy type 2i | muscular dystrophies, limb girdle | D049288 | Limb-Girdle Muscular Dystrophy | D049288 | Q9H9S5 | 2 |  |
| OMIM | T | 3.0717 | muscular dystrophy, limb-girdle, type 2i | muscular dystrophies, limb girdle | D049288 | Limb-Girdle Muscular Dystrophy | D049288 | Q9H9S5 | 2 |  |
| SP | T | -1.0666 | hemochromatosis type 4 | hemochromatoses | D006432 | Hemochromatosis | D006432 | Q9NP59 | 1 |  |
| OMIM | T | -0.7757 | hemochromatosis, autosomal dominant | hemochromatoses | D006432 | Hemochromatosis | D006432 | Q9NP59 | 1 |  |
| SP | T | exact | sialuria | sialuria | D029461 | Sialuria | D029461 | Q9Y223 | 1 |  |
| OMIM | T | exact | sialuria | sialuria | D029461 | Sialuria | D029461 | Q9Y223 | 1 |  |
| SP | F | -3.7089 | miyoshi myopathy | myopathy | D009135 | Distal Muscular Dystrophy | D049310 | O75923 | 2 |  |
| OMIM | T | -0.8909 | muscular dystrophy, distal, late-onset, autosomal recessive | distal muscular dystrophy | D049310 | Distal Muscular Dystrophy | D049310 | O75923 | 2 |  |
| SP | T | 0.6097 | stem cell myeloproliferative disorder | disorder, myeloproliferative | D009196 | Neoplastic Syndrome, Hereditary | Lymphoma, Lymphoblastic | Myeloproliferative Disorder | Eosinophilia | D009386 D016401 D009196 D004802 | O95684 | 1 |  |
| OMIM | no OMIM |  |  |  |  | Neoplastic Syndrome, Hereditary | Lymphoma, Lymphoblastic | Myeloproliferative Disorder | Eosinophilia | D009386 D016401 D009196 D004802 | O95684 | 1 |  |
| SP | T | 4.5277 | oral squamous cell carcinoma | carcinoma, squamous cell | D002294 | Squamous Cell Carcinoma | Oral Cancer | D002294 D009062 | P01112 | 4 |  |
| OMIM | no OMIM |  |  |  |  | Squamous Cell Carcinoma | Oral Cancer | D002294 D009062 | P01112 | 4 |  |
| SP | F | 5.3478 | high density lipoprotein deficiency type 2 | high density lipoprotein deficiency, type i | D013631 | Hypoalphalipoproteinemia, Familial | D052456 | P02647 | 1 |  |
| OMIM | T | exact | hypoalphalipoproteinemia, familial | hypoalphalipoproteinemia, familial | D052456 | Hypoalphalipoproteinemia, Familial | D052456 | P02647 | 1 |  |
| SP | F | -5.9912 | systemic non-neuropathic amyloidosis | amyloidoses | D000686 | Amyloidosis, Familial | D028226 | P02647 | 3 |  |
| OMIM | T | 3.5746 | amyloidosis, familial renal | familial amyloidoses | D028226 | Amyloidosis, Familial | D028226 | P02647 | 3 |  |
| SP | T | -1.0552 | senile cataract | cataract | D002386 | Genetic Predisposition to Disease | Cataract | D020022 D002386 | P07315 | 3 |  |
| OMIM | no OMIM |  |  |  |  | Genetic Predisposition to Disease | Cataract | D020022 D002386 | P07315 | 3 |  |
| SP | F | -5.5317 | hypertrophic cardiomyopathy with mid-left ventricular chamber type 1 | hypertrophic cardiomyopathies | D002312 | Cardiomyopathy, Hypertrophic, Familial | D024741 | P08590 | 2 |  |
| OMIM | T | 4.6686 | cardiomyopathy, familial hypertrophic, 8 | cardiomyopathies, familial hypertrophic | D024741 | Cardiomyopathy, Hypertrophic, Familial | D024741 | P08590 | 2 |  |
| SP | F | 3.4296 | glycogen storage disease type 2 | glycogen storage disease type i | D005953 | Glycogen Storage Disease Type II | D006009 | P10253 | 1 |  |
| OMIM | T | exact | pompe disease | pompe disease | D006009 | Glycogen Storage Disease Type II | D006009 | P10253 | 1 |  |
| SP | F | -8.4762 | osteoglophonic dysplasia | bone dysplasia | D001848 | Dwarfism | Osteochondrodysplasia | Craniosynostosis | D004392 D010009 D003398 | P11362 | 4 |  |
| OMIM | T | -2.0749 | osteoglophonic dwarfism | dwarfism | D004392 | Dwarfism | Osteochondrodysplasia | Craniosynostosis | D004392 D010009 D003398 | P11362 | 4 |  |
| SP | F | -22.885 | non-syndromic trigonocephaly | lymphoma, non hodgkin's | D008228 | Craniosynostosis | D003398 | P11362 | 5 |  |
| OMIM | T | -2.2773 | craniosynostosis, metopic | craniosynostoses | D003398 | Craniosynostosis | D003398 | P11362 | 5 |  |
| SP | T | 0.6097 | stem cell myeloproliferative disorder | disorder, myeloproliferative | D009196 | Neoplastic Syndrome, Hereditary | Lymphoma, Lymphoblastic | Myeloproliferative Disorder | Eosinophilia | D009386 D016401 D009196 D004802 | P11362 | 7 |  |
| OMIM | no OMIM |  |  |  |  | Neoplastic Syndrome, Hereditary | Lymphoma, Lymphoblastic | Myeloproliferative Disorder | Eosinophilia | D009386 D016401 D009196 D004802 | P11362 | 7 |  |
| SP | T | exact | epidermolysis bullosa simplex | epidermolysis bullosa simplex | D016110 | Epidermolysis Bullosa Simplex | D016110 | P13647 | 1 |  |
| OMIM | F | exact | epidermolysis bullosa dystrophica, cockayne-touraine type | epidermolysis bullosa dystrophica, cockayne-touraine type | D016108 | Epidermolysis Bullosa Simplex | D016110 | P13647 | 1 |  |
| SP | T | exact | schizophrenia | schizophrenia | D012559 | Schizophrenia | D012559 | P21918 | 1 |  |
| OMIM | no OMIM |  |  |  |  | Schizophrenia | D012559 | P21918 | 1 |  |
| SP | T | exact | blepharospasm | blepharospasm | D001764 | Blepharospasm | D001764 | P21918 | 2 |  |
| OMIM | F | -0.7357 | blepharospasm, benign essential | essential tremors, benign | D020329 | Blepharospasm | D001764 | P21918 | 2 |  |
| SP | F | 2.3495 | non-bullous congenital ichthyosiform erythroderma | congenital ichthyosiform erythroderma | D016113 | Nonbullous Congenital Ichthyosiform Erythroderma | D017490 | P22735 | 2 |  |
| OMIM | T | 8.1821 | ichthyosiform erythroderma, congenital, nonbullous, 1 | ichthyosiform erythroderma, nonbullous congenital | D017490 | Nonbullous Congenital Ichthyosiform Erythroderma | D017490 | P22735 | 2 |  |
| SP | F | 2.4413 | rhabdomyosarcoma 2 | rhabdomyosarcoma | D012208 | Alveolar Rhabdomyosarcoma | D018232 | P23760 | 4 |  |
| OMIM | T | exact | rhabdomyosarcoma, alveolar | rhabdomyosarcoma, alveolar | D018232 | Alveolar Rhabdomyosarcoma | D018232 | P23760 | 4 |  |
| SP | T | -3.8405 | posterior polymorphous corneal dystrophy | hereditary corneal dystrophies | D003317 | Corneal Dystrophy, Hereditary | D003317 | P25067 | 1 |  |
| OMIM | T | 0.5274 | corneal dystrophy, hereditary polymorphous posterior | hereditary corneal dystrophies | D003317 | Corneal Dystrophy, Hereditary | D003317 | P25067 | 1 |  |
| SP | T | -0.8576 | tumor development | tumors | D009369 | Neoplasms | D009369 | P35222 | 1 |  |
| OMIM | no OMIM |  |  |  |  | Neoplasms | D009369 | P35222 | 1 |  |
| SP | F | -6.6606 | autosomal dominant potassium-aggravated myotonia | myotonias | D009222 | Myotonic Disorder | D020967 | P35499 | 4 |  |
| OMIM | T | exact | myotonia fluctuans | myotonia fluctuans | D020967 | Myotonic Disorder | D020967 | P35499 | 4 |  |
| SP | T | exact | congenital myasthenic syndrome | congenital myasthenic syndrome | D020294 | Congenital Myasthenic Syndrome | D020294 | P35499 | 5 |  |
| OMIM | T | -5.1729 | myasthenic syndrome due to mutation in scn4a | congenital myasthenic syndrome | D020294 | Congenital Myasthenic Syndrome | D020294 | P35499 | 5 |  |
| SP | F | -1.1235 | mass syndrome | mass behaviors | D008399 | Bone Diseases, Developmental | Heart Defects, Congenital | Abnormalities, Multiple | Genetic Diseases, Inborn | Connective Tissue Diseases | D001848 D006330 D000015 D030342 D003240 | P35555 | 5 |  |
| OMIM | T | 3.8553 | overlap connective tissue disease | diseases, connective tissue | D003240 | Bone Diseases, Developmental | Heart Defects, Congenital | Abnormalities, Multiple | Genetic Diseases, Inborn | Connective Tissue Diseases | D001848 D006330 D000015 D030342 D003240 | P35555 | 5 |  |
| SP | F | -0.1875 | juvenile polyposis syndrome | familial polyposis syndrome | D011125 | Intestinal Polyposis | Neoplastic Syndrome, Hereditary | Gastrointestinal Neoplasms | Hamartomas | D044483 D009386 D005770 D006222 | P36894 | 1 |  |
| OMIM | T | 4.4418 | polyposis, juvenile intestinal | intestinal polyposis | D044483 | Intestinal Polyposis | Neoplastic Syndrome, Hereditary | Gastrointestinal Neoplasms | Hamartomas | D044483 D009386 D005770 D006222 | P36894 | 1 |  |
| SP | T | -1.9767 | maternal acute fatty liver of pregnancy | liver, fatty | D005234 | Pregnancy Complications | Fatty Liver | D011248 D005234 | P40939 | 3 |  |
| OMIM | F | -7.5206 | lchad deficiency | deficiency diseases | D003677 | Pregnancy Complications | Fatty Liver | D011248 D005234 | P40939 | 3 |  |
| SP | T | -0.0964 | pro-lymphocytic t-cell leukemia | t-cell leukemia | D015458 | Leukemia, T-Cell | D015458 | P46736 | 1 |  |
| OMIM | no OMIM |  |  |  |  | Leukemia, T-Cell | D015458 | P46736 | 1 |  |
| SP | T | -2.9147 | frontotemporal dementia | lobar degenerations, frontotemporal | D003704 | Frontotemporal Lobar Degeneration | D003704 | P49768 | 2 |  |
| OMIM | T | exact | frontotemporal lobar degeneration | frontotemporal lobar degeneration | D003704 | Frontotemporal Lobar Degeneration | D003704 | P49768 | 2 |  |
| SP | F | 4.3752 | myotonic dystrophy 2 | dystrophies, myotonic | D009223 | Proximal Myotonic Myopathy | D020967 | P62633 | 1 |  |
| OMIM | T | exact | proximal myotonic myopathy | proximal myotonic myopathy | D020967 | Proximal Myotonic Myopathy | D020967 | P62633 | 1 |  |
| SP | T | 1.617 | non-syndromal x-linked mental retardation | mental retardation, x linked | D038901 | Mental Retardation, X-Linked | D038901 | P98174 | 2 |  |
| OMIM | no OMIM |  |  |  |  | Mental Retardation, X-Linked | D038901 | P98174 | 2 |  |
| SP | F | -8.7353 | steatocystoma multiplex | mononeuropathy multiplex | D020422 | Skin Disease, Genetic | Sebaceous Cysts | D012873 D004814 | Q04695 | 2 |  |
| OMIM | T | 4.6464 | sebaceous cysts, multiple | cysts, sebaceous | D004814 | Skin Disease, Genetic | Sebaceous Cysts | D012873 D004814 | Q04695 | 2 |  |
| SP | T | exact | acute promyelocytic leukemia | acute promyelocytic leukemia | D015473 | Acute Promyelocytic Leukemia | D015473 | Q05516 | 1 |  |
| OMIM | no OMIM |  |  |  |  | Acute Promyelocytic Leukemia | D015473 | Q05516 | 1 |  |
| SP | T | -2.7175 | myokymia with periodic ataxia | myokymia | D020385 | Genetic Disease, Inborn | Neuromuscular Disease | Ataxia | Myokymia | D030342 D009468 D001259 D020385 | Q09470 | 1 |  |
| OMIM | T | exact | myokymia | myokymia | D020385 | Genetic Disease, Inborn | Neuromuscular Disease | Ataxia | Myokymia | D030342 D009468 D001259 D020385 | Q09470 | 1 |  |
| SP | T | 3.8352 | x-linked mental retardation in xq13 | mental retardation, x linked | D038901 | Mental Retardation, X-Linked | D038901 | Q14202 | 1 |  |
| OMIM | no OMIM |  |  |  |  | Mental Retardation, X-Linked | D038901 | Q14202 | 1 |  |
| SP | T | exact | endometrial stromal tumors | endometrial stromal tumors | D036821 | Endometrial Stromal Tumors | D036821 | Q15022 | 1 |  |
| OMIM | no OMIM |  |  |  |  | Endometrial Stromal Tumors | D036821 | Q15022 | 1 |  |
| SP | T | 4.228 | form of b-cell leukemia | b-cell leukemias | D015448 | B-Cell Leukemia | D015448 | Q16633 | 1 |  |
| OMIM | no OMIM |  |  |  |  | B-Cell Leukemia | D015448 | Q16633 | 1 |  |
| SP | T | -4.7981 | ataxia-oculomotor apraxia 1 | apraxia | D001072 | Early Onset Cerebellar Ataxia | Peripheral Neuropathies | Apraxia, Motor | Hypoalbuminemia | D013132 D010523 D001072 D034141 | Q7Z2E3 | 1 |  |
| OMIM | T | 4.4515 | cerebellar ataxia, early-onset, with hypoalbuminemia | cerebellar ataxia, early onset | D013132 | Early Onset Cerebellar Ataxia | Peripheral Neuropathies | Apraxia, Motor | Hypoalbuminemia | D013132 D010523 D001072 D034141 | Q7Z2E3 | 1 |  |
| SP | T | -5.0948 | female-specific osteoarthritis susceptibility | osteoarthritis | D010003 | Genetic Predisposition to Disease | Osteoarthritis | D020022 D010003 | Q92765 | 1 |  |
| OMIM | T | exact | osteoarthritis | osteoarthritis | D010003 | Genetic Predisposition to Disease | Osteoarthritis | D020022 D010003 | Q92765 | 1 |  |
| SP | F | -5.223 | omenn syndrome | syndromes | D013577 | Severe Combined Immunodeficiency | D016511 | Q96SD1 | 4 |  |
| OMIM | T | 3.4379 | severe combined immunodeficiency with hypereosinophilia | immunodeficiencies, severe combined | D016511 | Severe Combined Immunodeficiency | D016511 | Q96SD1 | 4 |  |
| SP | T | exact | zellweger syndrome | zellweger syndrome | D015211 | Zellweger Syndrome | D015211 | Q99424 | 1 |  |
| OMIM | no OMIM |  |  |  |  | Zellweger Syndrome | D015211 | Q99424 | 1 |  |
| SP | T | exact | breast cancer | breast cancer | D001943 | Breast Cancer | D001943 | Q9H6U6 | 1 |  |
| OMIM | no OMIM |  |  |  |  | Breast Cancer | D001943 | Q9H6U6 | 1 |  |
| SP | F | -7.6241 | walker-warburg syndrome | syndromes | D013577 | Genetic Disease, Inborn | Abnormalities, Multiple | Muscular Dystrophy | Brain Diseases | Retinal Dysplasia | D030342 D000015 D009136 D001927 D015792 | Q9H9S5 | 4 |  |
| OMIM | T | -2.2899 | hydrocephalus, agyria, and retinal dysplasia | retinal dysplasia | D015792 | Genetic Disease, Inborn | Abnormalities, Multiple | Muscular Dystrophy | Brain Diseases | Retinal Dysplasia | D030342 D000015 D009136 D001927 D015792 | Q9H9S5 | 4 |  |
| SP | T | -2.2617 | azoospermia or oligospermia | oligospermia | D009845 | Azoospermia | Oligospermia | D053713 D009845 | Q9NQZ3 | 1 |  |
| OMIM | no OMIM |  |  |  |  | Azoospermia | Oligospermia | D053713 D009845 | Q9NQZ3 | 1 |  |
| SP | T | exact | chronic neutrophilic leukemia | chronic neutrophilic leukemia | D015467 | Chronic Neutrophilic Leukemia | D015467 | Q9NVA2 | 1 |  |
| OMIM | no OMIM |  |  |  |  | Chronic Neutrophilic Leukemia | D015467 | Q9NVA2 | 1 |  |
| SP | F | -3.2089 | nonaka myopathy | myopathy | D009135 | Distal Myopathy | D049310 | Q9Y223 | 3 |  |
| OMIM | T | 1.4836 | nonaka distal myopathy | myopathies, distal | D049310 | Distal Myopathy | D049310 | Q9Y223 | 3 |  |
| SP | F | -6.139 | lacticacidemia | acidosis, lactic | D000140 | Pyruvate Dehydrogenase Complex Deficiency Disease | D015325 | O00330 | 1 |  |
| OMIM | T | -3.8486 | pyruvate dehydrogenase e3-binding protein deficiency | pyruvate dehydrogenase complex deficiency disease | D015325 | Pyruvate Dehydrogenase Complex Deficiency Disease | D015325 | O00330 | 1 |  |
| SP | T | -4.5341 | variety of human tumors | tumors | D009369 | Neoplasms | D009369 | P01112 | 2 |  |
| OMIM | no OMIM |  |  |  |  | Neoplasms | D009369 | P01112 | 2 |  |
| SP | F | -5.4305 | kniest syndrome | syndromes | D013577 | Genetic Disease, Inborn | Abnormalities, Multiple | Osteochondrodysplasia | Dwarfism | Craniofacial Abnormalities | D030342 D000015 D010009 D004392 D019465 | P02458 | 5 |  |
| OMIM | T | -5.1473 | metatropic dwarfism, type ii | dwarfism | D004392 | Genetic Disease, Inborn | Abnormalities, Multiple | Osteochondrodysplasia | Dwarfism | Craniofacial Abnormalities | D030342 D000015 D010009 D004392 D019465 | P02458 | 5 |  |
| SP | T | -3.1863 | osteoarthritis with mild chondrodysplasia | osteoarthritis | D010003 | Genetic Disease, Inborn | Osteochondrodysplasia | Osteoarthritis | D030342 D010009 D010003 | P02458 | 7 |  |
| OMIM | T | -3.1863 | osteoarthritis with mild chondrodysplasia | osteoarthritis | D010003 | Genetic Disease, Inborn | Osteochondrodysplasia | Osteoarthritis | D030342 D010009 D010003 | P02458 | 7 |  |
| SP | T | -5.5957 | coppock-like cataract | cataract | D002386 | Genetic Disease, Inborn | Cataract | D030342 D002386 | P07315 | 2 |  |
| OMIM | T | -5.2227 | cataract, embryonic nuclear | cataract | D002386 | Genetic Disease, Inborn | Cataract | D030342 D002386 | P07315 | 2 |  |
| SP | T | -5.319 | autosomal recessive severe combined immunodeficiency t-cell-negative/b-cell- positive/nk cell-positive | immunodeficiencies, severe combined | D016511 | Severe Combined Immunodeficiency | D016511 | P08575 | 1 |  |
| OMIM | T | -5.319 | severe combined immunodeficiency, autosomal recessive, t cell-negative, b cell-positive, nk cell-positive | immunodeficiencies, severe combined | D016511 | Severe Combined Immunodeficiency | D016511 | P08575 | 1 |  |
| SP | F | -8.3797 | trismus- pseudocamptodactyly syndrome | syndromes | D013577 | Abnormalities, Multiple | Arthrogryposis | D000015 D001176 | P13535 | 2 |  |
| OMIM | T | -3.9776 | arthrogryposis, distal, type 7 | arthrogryposis | D001176 | Abnormalities, Multiple | Arthrogryposis | D000015 D001176 | P13535 | 2 |  |
| SP | T | -4.79 | posterior polymorphous corneal dystrophy 2 | hereditary corneal dystrophies | D003317 | Corneal Dystrophy, Hereditary | D003317 | P25067 | 3 |  |
| OMIM | T | -4.79 | corneal dystrophy, posterior polymorphous, 2 | hereditary corneal dystrophies | D003317 | Corneal Dystrophy, Hereditary | D003317 | P25067 | 3 |  |
| SP | T | -5.269 | familial hyperinsulinemic hypoglycemia type 3 | hypoglycemia | D007003 | Metabolism, Inborn Errors | Hyperinsulinism | Hypoglycemia | D008661 D006946 D007003 | P35557 | 2 |  |
| OMIM | T | -4.8927 | hyperinsulinemic hypoglycemia, familial, 3 | hypoglycemia | D007003 | Metabolism, Inborn Errors | Hyperinsulinism | Hypoglycemia | D008661 D006946 D007003 | P35557 | 2 |  |
| SP | T | -3.8688 | alternating hemiplegia of childhood | hemiplegia | D006429 | Genetic Disease, Inborn | Hemiplegia, Infantile | D030342 D006429 | P50993 | 2 |  |
| OMIM | T | -3.8688 | alternating hemiplegia of childhood | hemiplegia | D006429 | Genetic Disease, Inborn | Hemiplegia, Infantile | D030342 D006429 | P50993 | 2 |  |
| SP | T | -2.9542 | the uterine cervix and in psoriasis vulgaris | cancer of the uterine cervix | D002583 | Psoriasis | Uterine Cervical Diseases | Uterine Cervical Neoplasms | D011565 D002577 D002583 | Q04695 | 3 |  |
| OMIM | no OMIM |  |  |  |  | Psoriasis | Uterine Cervical Diseases | Uterine Cervical Neoplasms | D011565 D002577 D002583 | Q04695 | 3 |  |
| SP | F | -8.77 | peroxisome biogenesis disorder complementation group 4 | t-group | D012681 | Peroxisomal Disorder | D018901 | Q13608 | 1 |  |
| OMIM | T | -7.628 | peroxisomal assembly factor 2 | peroxisomal disorder | D018901 | Peroxisomal Disorder | D018901 | Q13608 | 1 |  |
| SP | F | -9.4008 | triphalangeal thumb- polysyndactyly syndrome | syndromes | D013577 | Limb Deformities, Congenital | Genetic Disease, Inborn | Polydactyly | Syndactyly | D017880 D030342 D017689 D013576 | Q15465 | 4 |  |
| OMIM | T | -4.0164 | triphalangeal thumb with polysyndactyly | polysyndactyly | D013576 | Limb Deformities, Congenital | Genetic Disease, Inborn | Polydactyly | Syndactyly | D017880 D030342 D017689 D013576 | Q15465 | 4 |  |
| SP | F | -9.7325 | amme complex | wagr complex | D017624 | Genetic Disease, X-Linked | Abnormalities, Multiple | Nephritis, Hereditary | Elliptocytosis, Hereditary | Craniofacial Abnormalities | Mental Retardation, X-Linked | D040181 D000015 D009394 D004612 D019465 D038901 | Q9Y4X0 | 1 |  |
| OMIM | T | -5.5813 | alport syndrome, mental retardation, midface hypoplasia, and elliptocytosis | alport syndromes | D009394 | Genetic Disease, X-Linked | Abnormalities, Multiple | Nephritis, Hereditary | Elliptocytosis, Hereditary | Craniofacial Abnormalities | Mental Retardation, X-Linked | D040181 D000015 D009394 D004612 D019465 D038901 | Q9Y4X0 | 1 |  |
| SP | F | -7.9264 | peeling skin syndrome acral type | skin diseases | D012871 | Skin Disease, Genetic | Skin Abnormalities | Skin Disease, Vesiculobullous | D012873 D012868 D012872 | O43548 | 1 |  |
| OMIM | F | -7.9264 | peeling skin syndrome, acral type | skin diseases | D012871 | Skin Disease, Genetic | Skin Abnormalities | Skin Disease, Vesiculobullous | D012873 D012868 D012872 | O43548 | 1 |  |
| SP | F | -5.4305 | costello syndrome | syndromes | D013577 | Genetic Disease, Inborn | Abnormalities, Multiple | Craniofacial Abnormalities | Skin Abnormalities | Heart Defects, Congenital | D030342 D000015 D019465 D012868 D006330 | P01112 | 1 |  |
| OMIM | F | -5.4305 | costello syndrome | syndromes | D013577 | Genetic Disease, Inborn | Abnormalities, Multiple | Craniofacial Abnormalities | Skin Abnormalities | Heart Defects, Congenital | D030342 D000015 D019465 D012868 D006330 | P01112 | 1 |  |
| SP | F | -7.412 | variety of chondrodysplasia including hypochondrogenesis and osteoarthritis | osteoarthritis | D010003 | Osteochondrodysplasia | D010009 | P02458 | 1 |  |
| OMIM | no OMIM |  |  |  |  | Osteochondrodysplasia | D010009 | P02458 | 1 |  |
| SP | F | -9.5244 | strudwick type spondyloepimetaphyseal dysplasia | bone dysplasia | D001848 | Genetic Disease, Inborn | Abnormalities, Multiple | Osteochondrodysplasia | Dwarfism | D030342 D000015 D010009 D004392 | P02458 | 3 |  |
| OMIM | F | -4.9305 | strudwick syndrome | syndromes | D013577 | Genetic Disease, Inborn | Abnormalities, Multiple | Osteochondrodysplasia | Dwarfism | D030342 D000015 D010009 D004392 | P02458 | 3 |  |
| SP | F | -9.1653 | achondrogenesis hypochondrogenesis type 2 | 2, neurofibromatosis type | D016518 | Genetic Disease, Inborn | Osteochondrodysplasia | Dwarfism | Craniofacial Abnormalities | D030342 D010009 D004392 D019465 | P02458 | 4 |  |
| OMIM | F | -6.656 | achondrogenesis, type ii | type ii, neurofibromatosis | D016518 | Genetic Disease, Inborn | Osteochondrodysplasia | Dwarfism | Craniofacial Abnormalities | D030342 D010009 D004392 D019465 | P02458 | 4 |  |
| SP | F | -8.6837 | spondyloperipheral dysplasia | bone dysplasia | D001848 | Genetic Disease, Inborn | Abnormalities, Multiple | Spondyloepiphyseal Dysplasia | Sensorineural Hearing Loss | Dwarfism | Craniofacial Abnormalities | D030342 D000015 D010009 D006319 D004392 D019465 | P02458 | 10 |  |
| OMIM | F | -8.0854 | spondyloperipheral dysplasia with short ulna | fracture, ulna | D014458 | Genetic Disease, Inborn | Abnormalities, Multiple | Spondyloepiphyseal Dysplasia | Sensorineural Hearing Loss | Dwarfism | Craniofacial Abnormalities | D030342 D000015 D010009 D006319 D004392 D019465 | P02458 | 10 |  |
| SP | F | -3.9683 | wagner syndrome type ii | usher syndrome, type ii | D052245 | Eye Disease, Hereditary | D015785 | P02458 | 11 |  |
| OMIM | no OMIM |  |  |  |  | Eye Disease, Hereditary | D015785 | P02458 | 11 |  |
| SP | F | -5.7579 | stickler syndrome type 1 | syndromes | D013577 | Genetic Disease, Inborn | Abnormalities, Multiple | Eye Disease, Hereditary | Bone Diseases | Sensorineural Hearing Loss | Craniofacial Abnormalities | D030342 D000015 D015785 D001847 D006319 D019465 | P02458 | 12 |  |
| OMIM | F | -3.4339 | stickler syndrome, type i | usher syndrome, type i | D052245 | Genetic Disease, Inborn | Abnormalities, Multiple | Eye Disease, Hereditary | Bone Diseases | Sensorineural Hearing Loss | Craniofacial Abnormalities | D030342 D000015 D015785 D001847 D006319 D019465 | P02458 | 12 |  |
| SP | F | -3.7086 | acid phosphatase deficiency | acid deficiency, folic | D005494 | Lysosomal Storage Disease | D016464 | P11117 | 1 |  |
| OMIM | F | -3.7086 | acid phosphatase deficiency | acid deficiency, folic | D005494 | Lysosomal Storage Disease | D016464 | P11117 | 1 |  |
| SP | F | -8.6929 | carney complex variant | migraine variants | D008881 | Neoplastic Syndromes, Hereditary | Abnormalities, Multiple | D009386 D000015 | P13535 | 1 |  |
| OMIM | F | -8.6929 | carney complex variant | migraine variants | D008881 | Neoplastic Syndromes, Hereditary | Abnormalities, Multiple | D009386 D000015 | P13535 | 1 |  |
| SP | F | -8.1507 | dowling-degos disease | diseases | D004194 | Skin Disease, Genetic | Hyperpigmentation | D012873 D017495 | P13647 | 7 |  |
| OMIM | F | -8.1507 | dowling-degos disease | diseases | D004194 | Skin Disease, Genetic | Hyperpigmentation | D012873 D017495 | P13647 | 7 |  |
| SP | F | -9.3543 | muscle-specific enolase- beta deficiency | deficiency diseases | D003677 | Glycogen Storage Disease | Myopathy | D006008 D009135 | P13929 | 1 |  |
| OMIM | F | -7.7061 | enolase 3 deficiency | antithrombin 3 deficiency | D020152 | Glycogen Storage Disease | Myopathy | D006008 D009135 | P13929 | 1 |  |
| SP | F | -8.0911 | periventricular nodular heterotopia 1 | nodular lymphomas | D008224 | Genetic Disease, X-Linked | Nervous System Malformation | D040181 D009421 | P21333 | 1 |  |
| OMIM | F | -5.2688 | heterotopia, periventricular, x-linked dominant | hypophosphatemic rickets, x linked dominant | D053098 | Genetic Disease, X-Linked | Nervous System Malformation | D040181 D009421 | P21333 | 1 |  |
| SP | F | -8.5683 | periventricular nodular heterotopia 4 | nodular lymphomas | D008224 | Genetic Disease, X-Linked | Abnormalities, Multiple | Nervous System Malformation | Joint Hypermobility | D040181 D000015 D009421 D007593 | P21333 | 2 |  |
| OMIM | F | -3.7967 | heterotopia, periventricular, ehlers-danlos variant | syndrome, ehlers-danlos | D004535 | Genetic Disease, X-Linked | Abnormalities, Multiple | Nervous System Malformation | Joint Hypermobility | D040181 D000015 D009421 D007593 | P21333 | 2 |  |
| SP | F | -8.6973 | otopalatodigital syndrome type 1 | syndromes | D013577 | Genetic Disease, X-Linked | Abnormalities, Multiple | Osteochondrodysplasia | Craniofacial Abnormalities | D040181 D000015 D010009 D019465 | P21333 | 3 |  |
| OMIM | F | -6.6228 | opd syndrome, type 1 | syndromes | D013577 | Genetic Disease, X-Linked | Abnormalities, Multiple | Osteochondrodysplasia | Craniofacial Abnormalities | D040181 D000015 D010009 D019465 | P21333 | 3 |  |
| SP | F | -8.7501 | otopalatodigital syndrome type 2 | syndromes | D013577 | Genetic Disease, X-Linked | Abnormalities, Multiple | Osteochondrodysplasia | Craniofacial Abnormalities | D040181 D000015 D010009 D019465 | P21333 | 4 |  |
| OMIM | F | -5.723 | cranioorodigital syndrome | syndromes | D013577 | Genetic Disease, X-Linked | Abnormalities, Multiple | Osteochondrodysplasia | Craniofacial Abnormalities | D040181 D000015 D010009 D019465 | P21333 | 4 |  |
| SP | F | -8.4762 | frontometaphyseal dysplasia | bone dysplasia | D001848 | Genetic Disease, X-Linked | Abnormalities, Multiple | Osteochondrodysplasia | Craniofacial Abnormalities | D040181 D000015 D010009 D019465 | P21333 | 5 |  |
| OMIM | F | -8.4762 | frontometaphyseal dysplasia | bone dysplasia | D001848 | Genetic Disease, X-Linked | Abnormalities, Multiple | Osteochondrodysplasia | Craniofacial Abnormalities | D040181 D000015 D010009 D019465 | P21333 | 5 |  |
| SP | F | -5.4305 | cerebrofrontofacial syndrome | syndromes | D013577 | Genetic Disease, X-Linked | Abnormalities, Multiple | Nervous System Malformation | Craniofacial Abnormalities | D040181 D000015 D009421 D019465 | P21333 | 7 |  |
| OMIM | F | -5.4305 | cerebrofrontofacial syndrome | syndromes | D013577 | Genetic Disease, X-Linked | Abnormalities, Multiple | Nervous System Malformation | Craniofacial Abnormalities | D040181 D000015 D009421 D019465 | P21333 | 7 |  |
| SP | F | -6.9626 | craniofacial-deafness- hand syndrome | syndromes | D013577 | Abnormalities, Multiple | Craniofacial Abnormalities | Sensorineural Hearing Loss | D000015 D019465 D006319 | P23760 | 3 |  |
| OMIM | F | -6.9626 | craniofacial-deafness-hand syndrome | syndromes | D013577 | Abnormalities, Multiple | Craniofacial Abnormalities | Sensorineural Hearing Loss | D000015 D019465 D006319 | P23760 | 3 |  |
| SP | F | -6.7679 | characteristic traits of polycystic ovary syndrome, such as insulin resistance and luteinizing hormon hypersecretion | syndrome, polycystic ovary | D011085 | Insulin Resistance | Pituitary LH Hypersecretion | D007333 D006964 | P26439 | 2 |  |
| OMIM | no OMIM |  |  |  |  | Insulin Resistance | Pituitary LH Hypersecretion | D007333 D006964 | P26439 | 2 |  |
| SP | F | -6.9894 | hyperprolinemia type ii | type ii, neurofibromatosis | D016518 | Amino Acid Metabolism, Inborn Error | D000592 | P30038 | 1 |  |
| OMIM | F | -6.9894 | hyperprolinemia, type ii | type ii, neurofibromatosis | D016518 | Amino Acid Metabolism, Inborn Error | D000592 | P30038 | 1 |  |
| SP | F | -4.2149 | certain cardiovascular and musculo-skeletal abnormalities observed in williams-beuren syndrome | syndrome, williams-beuren | D018980 | Musculoskeletal Abnormalities | Cardiovascular Abnormalities | D009139 D018376 | P35250 | 1 |  |
| OMIM | no OMIM |  |  |  |  | Musculoskeletal Abnormalities | Cardiovascular Abnormalities | D009139 D018376 | P35250 | 1 |  |
| SP | F | -4.6331 | autosomal dominant weill-marchesani syndrome | alport syndrome, autosomal dominant | D009394 | Abnormalities, Multiple | Connective Tissue Disease | Bone Disease, Developmental | Eye Disease, Hereditary | D000015 D003240 D001848 D015785 | P35555 | 3 |  |
| OMIM | F | -4.6331 | weill-marchesani syndrome, autosomal dominant | alport syndrome, autosomal dominant | D009394 | Abnormalities, Multiple | Connective Tissue Disease | Bone Disease, Developmental | Eye Disease, Hereditary | D000015 D003240 D001848 D015785 | P35555 | 3 |  |
| SP | F | -3.8203 | hereditary mixed polyposis syndrome 2 | familial polyposis syndrome | D011125 | Intestinal Polyposis | Neoplastic Syndrome, Hereditary | Colonic Neoplasms | D044483 D009386 D003110 | P36894 | 3 |  |
| OMIM | F | -3.8203 | polyposis syndrome, hereditary mixed, 2 | familial polyposis syndrome | D011125 | Intestinal Polyposis | Neoplastic Syndrome, Hereditary | Colonic Neoplasms | D044483 D009386 D003110 | P36894 | 3 |  |
| SP | F | -8.2603 | long-chain 3-hydroxyl- coa dehydrogenase deficiency | deficiencies, glucosephosphate dehydrogenase | D005955 | Lipid Metabolism, Inborn Error | Mitochondrial Disease | D008052 D028361 | P40939 | 2 |  |
| OMIM | F | -7.5206 | lchad deficiency | deficiency diseases | D003677 | Lipid Metabolism, Inborn Error | Mitochondrial Disease | D008052 D028361 | P40939 | 2 |  |
| SP | F | -4.8884 | short qt syndrome type 2 | bowel syndromes, short | D012778 | Genetic Disease, Inborn | Arrhythmia | D030342 D001145 | P51787 | 4 |  |
| OMIM | F | -4.4169 | short qt syndrome 2 | bowel syndromes, short | D012778 | Genetic Disease, Inborn | Arrhythmia | D030342 D001145 | P51787 | 4 |  |
| SP | F | -5.672 | norrie disease | diseases | D004194 | Genetic Disease, X-Linked | Eye Disease, Hereditary | Retinal Dysplasia | D040181 D015785 D015792 | Q00604 | 1 |  |
| OMIM | F | -3.8874 | episkopi blindness | blindness | D001766 | Genetic Disease, X-Linked | Eye Disease, Hereditary | Retinal Dysplasia | D040181 D015785 D015792 | Q00604 | 1 |  |
| SP | F | -6.0099 | x-linked familial exudative vitreoretinopathy | ichthyosis, x-linked | D016114 | Genetic Disease, X-Linked | Eye Disease, Hereditary | Retinal Dysplasia | D040181 D015785 D015792 | Q00604 | 2 |  |
| OMIM | F | -2.8909 | fevr, x-linked | ichthyosis, x-linked | D016114 | Genetic Disease, X-Linked | Eye Disease, Hereditary | Retinal Dysplasia | D040181 D015785 D015792 | Q00604 | 2 |  |
| SP | F | -8.8007 | axenfeld-rieger syndrome | syndromes | D013577 | Abnormalities, Multiple | Eye Disease, Hereditary | Glaucoma, Angle-Closure | Eye Abnormalities | Craniofacial Abnormalities | D000015 D015785 D015812 D005124 D019465 | Q12948 | 1 |  |
| OMIM | no OMIM |  |  |  |  | Abnormalities, Multiple | Eye Disease, Hereditary | Glaucoma, Angle-Closure | Eye Abnormalities | Craniofacial Abnormalities | D000015 D015785 D015812 D005124 D019465 | Q12948 | 1 |  |
| SP | F | -8.7841 | iridogoniodysgenesis anomaly | anomalies, pupillary | D011681 | Abnormalities, Multiple | Eye Disease, Hereditary | Glaucoma, Angle-Closure | Eye Abnormalities | D000015 D015785 D015812 D005124 | Q12948 | 2 |  |
| OMIM | F | -5.0515 | glaucoma iridogoniodysplasia, familial | glaucoma | D005901 | Abnormalities, Multiple | Eye Disease, Hereditary | Glaucoma, Angle-Closure | Eye Abnormalities | D000015 D015785 D015812 D005124 | Q12948 | 2 |  |
| SP | F | -8.3804 | peters anomaly | anomalies, pupillary | D011681 | Eye Disease, Hereditary | Eye Abnormalities | D015785 D005124 | Q12948 | 3 |  |
| OMIM | F | -8.3804 | peters anomaly | anomalies, pupillary | D011681 | Eye Disease, Hereditary | Eye Abnormalities | D015785 D005124 | Q12948 | 3 |  |
| SP | F | -4.16 | autosomal dominant filaminopathy | dominant parkinsonism, autosomal | D020734 | Muscular Dystrophy | D009136 | Q14315 | 1 |  |
| OMIM | F | -4.16 | filaminopathy, autosomal dominant | dominant parkinsonism, autosomal | D020734 | Muscular Dystrophy | D009136 | Q14315 | 1 |  |
| SP | F | -8.9993 | solitary median maxillary central incisor | disease, maxillary | D008439 | Tooth Abnormality | D014071 | Q15465 | 3 |  |
| OMIM | F | -5.223 | smmci syndrome | syndromes | D013577 | Tooth Abnormality | D014071 | Q15465 | 3 |  |
| SP | F | -4.717 | multiple exostoses observed in langer-giedon syndrome | multiple exostoses | D005097 | Exostoses | D005096 | Q16394 | 2 |  |
| OMIM | no OMIM |  |  |  |  | Exostoses | D005096 | Q16394 | 2 |  |
| SP | F | -10.845 | bietti crystalline corneoretinal dystrophy | diseases, retinal | D012164 | Eye Disease, Hereditary | Retinal Degeneration | Corneal Dystrophy, Hereditary | D015785 D012162 D003317 | Q6ZWL3 | 1 |  |
| OMIM | F | -4.4198 | bietti tapetoretinal degeneration with marginal corneal dystrophy | degeneration, tapetoretinal | D012174 | Eye Disease, Hereditary | Retinal Degeneration | Corneal Dystrophy, Hereditary | D015785 D012162 D003317 | Q6ZWL3 | 1 |  |
| SP | F | -9.7806 | coenzyme q10 deficiency | deficiency diseases | D003677 | Abnormalities, Multiple | Brain Diseases, Metabolic, Inborn | Cerebellar Ataxia | D000015 D020739 D002524 | Q7Z2E3 | 2 |  |
| OMIM | F | -8.8041 | coq10 deficiency, primary | deficiency diseases | D003677 | Abnormalities, Multiple | Brain Diseases, Metabolic, Inborn | Cerebellar Ataxia | D000015 D020739 D002524 | Q7Z2E3 | 2 |  |
| SP | F | -7.7281 | icos deficiency | deficiency diseases | D003677 | Common Variable Immunodeficiency | D017074 | Q9Y6W8 | 1 |  |
| OMIM | F | -7.7281 | icos deficiency | deficiency diseases | D003677 | Common Variable Immunodeficiency | D017074 | Q9Y6W8 | 1 |  |
| SP | F | -2.3535 | distal myopathy with anterior tibial onset | anterior tibial syndromes | D000868 | Distal Muscular Dystrophy | D049310 | O75923 | 3 |  |
| OMIM | F | -2.3535 | myopathy, distal, with anterior tibial onset | anterior tibial syndromes | D000868 | Distal Muscular Dystrophy | D049310 | O75923 | 3 |  |
| SP | F | -10.055 | platyspondylic lethal skeletal dysplasia torrance type | lethal catatonia | D002389 | Genetic Disease, Inborn | Osteochondrodysplasia | Dwarfism | D030342 D010009 D004392 | P02458 | 8 |  |
| OMIM | F | -1.0708 | thanatophoric dysplasia, torrance variant | dysplasia, thanatophoric | D013796 | Genetic Disease, Inborn | Osteochondrodysplasia | Dwarfism | D030342 D010009 D004392 | P02458 | 8 |  |
| SP | F | -0.3982 | cryptogenic cirrhosis | cirrhosis | D005355 | Liver Cirrhosis | D008103 | P05783 | 1 |  |
| OMIM | F | 1.8468 | cirrhosis, familial | cirrhosis | D005355 | Liver Cirrhosis | D008103 | P05783 | 1 |  |
| SP | F | -0.3982 | cryptogenic cirrhosis | cirrhosis | D005355 | Liver Cirrhosis | D008103 | P05787 | 1 |  |
| OMIM | F | 1.8468 | cirrhosis, familial | cirrhosis | D005355 | Liver Cirrhosis | D008103 | P05787 | 1 |  |
| SP | T | -6.3083 | gelatinous drop-like corneal dystrophy | hereditary corneal dystrophies | D003317 | Corneal Dystrophy, Hereditary | D003317 | P09758 | 1 |  |
| OMIM | F | 0.5231 | amyloidosis, corneal | amyloidoses | D000686 | Corneal Dystrophy, Hereditary | D003317 | P09758 | 1 |  |
| SP | F | -2.1702 | stem cell leukemia lymphoma syndrome | adult t-cell leukemia-lymphoma | D015460 | Neoplastic Syndrome, Hereditary | Lymphoma, Lymphoblastic | Myeloproliferative Disorder | Eosinophilia | D009386 D016401 D009196 D004802 | P11362 | 6 |  |
| OMIM | no OMIM |  |  |  |  | Neoplastic Syndrome, Hereditary | Lymphoma, Lymphoblastic | Myeloproliferative Disorder | Eosinophilia | D009386 D016401 D009196 D004802 | P11362 | 6 |  |
| SP | T | 0.3964 | epidermolysis bullosa simplex weber-cockayne type | epidermolysis bullosa simplex | D016110 | Weber-Cockayne Syndrome | D016110 | P13647 | 4 |  |
| OMIM | F | exact | epidermolysis bullosa dystrophica, cockayne-touraine type | epidermolysis bullosa dystrophica, cockayne-touraine type | D016108 | Weber-Cockayne Syndrome | D016110 | P13647 | 4 |  |
| SP | F | -1.9877 | hematopoietic tumors such as b-cell lymphomas | lymphoma, b cell | D016393 | Hematologic Neoplasms | D019337 | P26196 | 1 |  |
| OMIM | no OMIM |  |  |  |  | Hematologic Neoplasms | D019337 | P26196 | 1 |  |
| SP | T | -6.2552 | shprintzen-goldberg craniosynostosis syndrome | craniosynostoses | D003398 | Abnormalities, Multiple | Connective Tissue Disease | Bone Disease, Developmental | Craniosynostosis | Heart Defects, Congenital | Eye Disease, Hereditary | D000015 D003240 D001848 D003398 D006330 D015785 | P35555 | 4 |  |
| OMIM | F | -1.0442 | craniosynostosis with arachnodactyly and abdominal hernias | abdominal hernias | D046449 | Abnormalities, Multiple | Connective Tissue Disease | Bone Disease, Developmental | Craniosynostosis | Heart Defects, Congenital | Eye Disease, Hereditary | D000015 D003240 D001848 D003398 D006330 D015785 | P35555 | 4 |  |
| SP | T | -0.974 | maturity onset diabetes of the young type 2 | maturity onset diabetes mellitus | D003924 | Genetic Disease, Inborn | Maturity-Onset Diabetes Mellitus | D030342 D003924 | P35557 | 1 |  |
| OMIM | F | exact | diabetes, gestational | diabetes, gestational | D016640 | Genetic Disease, Inborn | Maturity-Onset Diabetes Mellitus | D030342 D003924 | P35557 | 1 |  |
| SP | F | 0.0504 | trifunctional protein deficiency | protein deficiency | D011488 | Lipid Metabolism, Inborn Error | Mitochondrial Disease | D008052 D028361 | P40939 | 1 |  |
| OMIM | F | 0.0504 | trifunctional protein deficiency | protein deficiency | D011488 | Lipid Metabolism, Inborn Error | Mitochondrial Disease | D008052 D028361 | P40939 | 1 |  |
| SP | F | -8.8076 | microphthalmia syndromic type 3 | type 3 gaucher disease | D005776 | Anophthalmia | Microphthalmos | D000853 D008850 | P48431 | 1 |  |
| OMIM | F | 1.37 | microphthalmia and esophageal atresia syndrome | esophageal atresia | D004933 | Anophthalmia | Microphthalmos | D000853 D008850 | P48431 | 1 |  |
| SP | F | -5.8108 | leukoencephalopathy with vanishing white matter | spongy disease of white matter | D017825 | Hereditary Central Nervous System Demyelinating Diseases | D020279 | P49770 | 1 |  |
| OMIM | F | -2.2785 | childhood ataxia with central nervous system hypomyelinization | central nervous system diseases | D002493 | Hereditary Central Nervous System Demyelinating Diseases | D020279 | P49770 | 1 |  |
| SP | no result |  | ovarioleukodystrophy |  |  | Hereditary Central Nervous System Demyelinating Diseases | Ovarian Failure, Premature | D020279 D016649 | P49770 | 2 |  |
| OMIM | F | -2.2785 | childhood ataxia with central nervous system hypomyelinization | central nervous system diseases | D002493 | Hereditary Central Nervous System Demyelinating Diseases | Ovarian Failure, Premature | D020279 D016649 | P49770 | 2 |  |
| SP | F | 2.5812 | bleeding disorder | bleeding | D006470 | Genetic Disease, Inborn | Hemorrhagic Disorder | D030342 D006474 | P51575 | 1 |  |
| OMIM | F | -5.5163 | bleeding disorder due to p2rx1 defect | bleeding | D006470 | Genetic Disease, Inborn | Hemorrhagic Disorder | D030342 D006474 | P51575 | 1 |  |
| SP | F | -9.2414 | aarskog-scott syndrome | syndromes | D013577 | Genetic Disease, X-Linked | Abnormalities, Multiple | Craniofacial Abnormalities | Urogenital Abnormalities | D040181 D000015 D019465 D014564 | P98174 | 1 |  |
| OMIM | F | 3.446 | faciogenital dysplasia with attention deficit-hyperactivity disorder | attention deficit disorders with hyperactivity | D001289 | Genetic Disease, X-Linked | Abnormalities, Multiple | Craniofacial Abnormalities | Urogenital Abnormalities | D040181 D000015 D019465 D014564 | P98174 | 1 |  |
| SP | F | -8.4762 | gnathodiaphyseal dysplasia | bone dysplasia | D001848 | Genetic Disease, Inborn | Osteochondrodysplasia | D030342 D010009 | Q75V66 | 1 |  |
| OMIM | F | -2.1352 | osteogenesis imperfecta with unusual skeletal lesions | osteogenesis imperfecta | D010013 | Genetic Disease, Inborn | Osteochondrodysplasia | D030342 D010009 | Q75V66 | 1 |  |
| SP | F | -2.0012 | chromosome 22q13.3 deletion syndrome | chromosome deletions | D002872 | Genetic Disease, Inborn | Abnormalities, Multiple | D030342 D000015 | Q8NEU8 | 1 |  |
| OMIM | F | -2.0012 | chromosome 22q13.3 deletion syndrome | chromosome deletions | D002872 | Genetic Disease, Inborn | Abnormalities, Multiple | D030342 D000015 | Q8NEU8 | 1 |  |
| SP | F | -0.7282 | normal pressure glaucoma | hydrocephalus, normal pressure | D006850 | Glaucoma | Genetic Predisposition to Disease | D005901 D020022 | Q96CV9 | 2 |  |
| OMIM | F | -3.024 | glaucoma, normal pressure, susceptibility to | hydrocephalus, normal pressure | D006850 | Glaucoma | Genetic Predisposition to Disease | D005901 D020022 | Q96CV9 | 2 |  |
| SP | F | -0.5309 | muscle-eye-brain disease | muscle-liver-brain-eye nanism | D050336 | Genetic Disease, Inborn | Abnormalities, Multiple | Muscular Dystrophy | Eye Diseases | Nervous System Diseases | D030342 D000015 D009136 D005128 D009422 | Q9H9S5 | 3 |  |
| OMIM | F | -0.5309 | muscle-eye-brain disease | muscle-liver-brain-eye nanism | D050336 | Genetic Disease, Inborn | Abnormalities, Multiple | Muscular Dystrophy | Eye Diseases | Nervous System Diseases | D030342 D000015 D009136 D005128 D009422 | Q9H9S5 | 3 |  |
| SP | F | 0.8696 | inclusion body myopathy type 2 | myopathy, inclusion body, sporadic | D018979 | Myopathy | D009135 | Q9Y223 | 2 |  |
| OMIM | F | 0.7779 | inclusion body myopathy, autosomal recessive | myopathy, inclusion body, sporadic | D018979 | Myopathy | D009135 | Q9Y223 | 2 |  |
|  |  |  |  |  |  |  |  |  |  |  |
