# Supplementary material for: Mapping proteins to disease terminologies: from UniProt to MeSH
Source: BMC Bioinformatics. 2008 Apr 29;9(Suppl 5):S3. doi: 10.1186/1471-2105-9-S5-S3 (PMC2367626; doi:10.1186/1471-2105-9-S5-S3)
Supplement: Additional file 2 — This file contains the automatic mapping of all Swiss-Prot disease names with a matching score above the threshold (html format). [file 1471-2105-9-S5-S3-S2.html]

# Swiss-Prot MeSH Mapping

### Score threshold=-2.5

|  |  |  |  |  |  |  |  |  |  |  |  |  |  |  |  |  |  |  |  |  |  |  |  |  |  |  |  |  |  |  |  |  |  |  |  |  |  |  |  |  |  |  |  |  |  |  |  |  |  |  |  |  |  |  |  |  |  |  |  |  |  |  |  |  |  |  |  |  |  |  |  |  |  |  |  |  |  |  |  |  |  |  |  |  |  |  |  |  |  |  |  |  |  |  |  |  |  |  |  |  |  |  |  |  |  |  |  |  |  |  |  |  |  |  |  |  |  |  |  |  |  |  |  |  |  |  |  |  |  |  |  |  |  |  |  |  |  |  |  |  |  |  |  |  |  |  |  |  |  |  |  |  |  |  |  |  |  |  |  |  |  |  |  |  |  |  |  |  |  |  |  |  |  |  |  |  |  |  |  |  |  |  |  |  |  |  |  |  |  |  |  |  |  |  |  |  |  |  |  |  |  |  |  |  |  |  |  |  |  |  |  |  |  |  |  |  |  |  |  |  |  |  |  |  |  |  |  |  |  |  |  |  |  |  |  |  |  |  |  |  |  |  |  |  |  |  |  |  |  |  |  |  |  |  |  |  |  |  |  |  |  |  |  |  |  |  |  |  |  |  |  |  |  |  |  |  |  |  |  |  |  |  |  |  |  |  |  |  |  |  |  |  |  |  |  |  |  |  |  |  |  |  |  |  |  |  |  |  |  |  |  |  |  |  |  |  |  |  |  |  |  |  |  |  |  |  |  |  |  |  |  |  |  |  |  |  |  |  |  |  |  |  |  |  |  |  |  |  |  |  |  |  |  |  |  |  |  |  |  |  |  |  |  |  |  |  |  |  |  |  |  |  |  |  |  |  |  |  |  |  |  |  |  |  |  |  |  |  |  |  |  |  |  |  |  |  |  |  |  |  |  |  |  |  |  |  |  |  |  |  |  |  |  |  |  |  |  |  |  |  |  |  |  |  |  |  |  |  |  |  |  |  |  |  |  |  |  |  |  |  |  |  |  |  |  |  |  |  |  |  |  |  |  |  |  |  |  |  |  |  |  |  |  |  |  |  |  |  |  |  |  |  |  |  |  |  |  |  |  |  |  |  |  |  |  |  |  |  |  |  |  |  |  |  |  |  |  |  |  |  |  |  |  |  |  |  |  |  |  |  |  |  |  |  |  |  |  |  |  |  |  |  |  |  |  |  |  |  |  |  |  |  |  |  |  |  |  |  |  |  |  |  |  |  |  |  |  |  |  |  |  |  |  |  |  |  |  |  |  |  |  |  |  |  |  |  |  |  |  |  |  |  |  |  |  |  |  |  |  |  |  |  |  |  |  |  |  |  |  |  |  |  |  |  |  |  |  |  |  |  |  |  |  |  |  |  |  |  |  |  |  |  |  |  |  |  |  |  |  |  |  |  |  |  |  |  |  |  |  |  |  |  |  |  |  |  |  |  |  |  |  |  |  |  |  |  |  |  |  |  |  |  |  |  |  |  |  |  |  |  |  |  |  |  |  |  |  |  |  |  |  |  |  |  |  |  |  |  |  |  |  |  |  |  |  |  |  |  |  |  |  |  |  |  |  |  |  |  |  |  |  |  |  |  |  |  |  |  |  |  |  |  |  |  |  |  |  |  |  |  |  |  |  |  |  |  |  |  |  |  |  |  |  |  |  |  |  |  |  |  |  |  |  |  |  |  |  |  |  |  |  |  |  |  |  |  |  |  |  |  |  |  |  |  |  |  |  |  |  |  |  |  |  |  |  |  |  |  |  |  |  |  |  |  |  |  |  |  |  |  |  |  |  |  |  |  |  |  |  |  |  |  |  |  |  |  |  |  |  |  |  |  |  |  |  |  |  |  |  |  |  |  |  |  |  |  |  |  |  |  |  |  |  |  |  |  |  |  |  |  |  |  |  |  |  |  |  |  |  |  |  |  |  |  |  |  |  |  |  |  |  |  |  |  |  |  |  |  |  |  |  |  |  |  |  |  |  |  |  |  |  |  |  |  |  |  |  |  |  |  |  |  |  |  |  |  |  |  |  |  |  |  |  |  |  |  |  |  |  |  |  |  |  |  |  |  |  |  |  |  |  |  |  |  |  |  |  |  |  |  |  |  |  |  |  |  |  |  |  |  |  |  |  |  |  |  |  |  |  |  |  |  |  |  |  |  |  |  |  |  |  |  |  |  |  |  |  |  |  |  |  |  |  |  |  |  |  |  |  |  |  |  |  |  |  |  |  |  |  |  |  |  |  |  |  |  |  |  |  |  |  |  |  |  |  |  |  |  |  |  |  |  |  |  |  |  |  |  |  |  |  |  |  |  |  |  |  |  |  |  |  |  |  |  |  |  |  |  |  |  |  |  |  |  |  |  |  |  |  |  |  |  |  |  |  |  |  |  |  |  |  |  |  |  |  |  |  |  |  |  |  |  |  |  |  |  |  |  |  |  |  |  |  |  |  |  |  |  |  |  |  |  |  |  |  |  |  |  |  |  |  |  |  |  |  |  |  |  |  |  |  |  |  |  |  |  |  |  |  |  |  |  |  |  |  |  |  |  |  |  |  |  |  |  |  |  |  |  |  |  |  |  |  |  |  |  |  |  |  |  |  |  |  |  |  |  |  |  |  |  |  |  |  |  |  |  |  |  |  |  |  |  |  |  |  |  |  |  |  |  |  |  |  |  |  |  |  |  |  |  |  |  |  |  |  |  |  |  |  |  |  |  |  |  |  |  |  |  |  |  |  |  |  |  |  |  |  |  |  |  |  |  |  |  |  |  |  |  |  |  |  |  |  |  |  |  |  |  |  |  |  |  |  |  |  |  |  |  |  |  |  |  |  |  |  |  |  |  |  |  |  |  |  |  |  |  |  |  |  |  |  |  |  |  |  |  |  |  |  |  |  |  |  |  |  |  |  |  |  |  |  |  |  |  |  |  |  |  |  |  |  |  |  |  |  |  |  |  |  |  |  |  |  |  |  |  |  |  |  |  |  |  |  |  |  |  |  |  |  |  |  |  |  |  |  |  |  |  |  |  |  |  |  |  |  |  |  |  |  |  |  |  |  |  |  |  |  |  |  |  |  |  |  |  |  |  |  |  |  |  |  |  |  |  |  |  |  |  |  |  |  |  |  |  |  |  |  |  |  |  |  |  |  |  |  |  |  |  |  |  |  |  |  |  |  |  |  |  |  |  |  |  |  |  |  |  |  |  |  |  |  |  |  |  |  |  |  |  |  |  |  |  |  |  |  |  |  |  |  |  |  |  |  |  |  |  |  |  |  |  |  |  |  |  |  |  |  |  |  |  |  |  |  |  |  |  |  |  |  |  |  |  |  |  |  |  |  |  |  |  |  |  |  |  |  |  |  |  |  |  |  |  |  |  |  |  |  |  |  |  |  |  |  |  |  |  |  |  |  |  |  |  |  |  |  |  |  |  |  |  |  |  |  |  |  |  |  |  |  |  |  |  |  |  |  |  |  |  |  |  |  |  |  |  |  |  |  |  |  |  |  |  |  |  |  |  |  |  |  |  |  |  |  |  |  |  |  |  |  |  |  |  |  |  |  |  |  |  |  |  |  |  |  |  |  |  |  |  |  |  |  |  |  |  |  |  |  |  |  |  |  |  |  |  |  |  |  |  |  |  |  |  |  |  |  |  |  |  |  |  |  |  |  |  |  |  |  |  |  |  |  |  |  |  |  |  |  |  |  |  |  |  |  |  |  |  |  |  |  |  |  |  |  |  |  |  |  |  |  |  |  |  |  |  |  |  |  |  |  |  |  |  |  |  |  |  |  |  |  |  |  |  |  |  |  |  |  |  |  |  |  |  |  |  |  |  |  |  |  |  |  |  |  |  |  |  |  |  |  |  |  |  |  |  |  |  |  |  |  |  |  |  |  |  |  |  |  |  |  |  |  |  |  |  |  |  |  |  |  |  |  |  |  |  |  |  |  |  |  |  |  |  |  |  |  |  |  |  |  |  |  |  |  |  |  |  |  |  |  |  |  |  |  |  |  |  |  |  |  |  |  |  |  |  |  |  |  |  |  |  |  |  |  |  |  |  |  |  |  |  |  |  |  |  |  |  |  |  |  |  |  |  |  |  |  |  |  |  |  |  |  |  |  |  |  |  |  |  |  |  |  |  |  |  |  |  |  |  |  |  |  |  |  |  |  |  |  |  |  |  |  |  |  |  |  |  |  |  |  |  |  |  |  |  |  |  |  |  |  |  |  |  |  |  |  |  |  |  |  |  |  |  |  |  |  |  |  |  |  |  |  |  |  |  |  |  |  |  |  |  |  |  |  |  |  |  |  |  |  |  |  |  |  |  |  |  |  |  |  |  |  |  |  |  |  |  |  |  |  |  |  |  |  |  |  |  |  |  |  |  |  |  |  |  |  |  |  |  |  |  |  |  |  |  |  |  |  |  |  |  |  |  |  |  |  |  |  |  |  |  |  |  |  |  |  |  |  |  |  |  |  |  |  |  |  |  |  |  |  |  |  |  |  |  |  |  |  |  |  |  |  |  |  |  |  |  |  |  |  |  |  |  |  |  |  |  |  |  |  |  |  |  |  |  |  |  |  |  |  |  |  |  |  |  |  |  |  |  |  |  |  |  |  |  |  |  |  |  |  |  |  |  |  |  |  |  |  |  |  |  |  |  |  |  |  |  |  |  |  |  |  |  |  |  |  |  |  |  |  |  |  |  |  |  |  |  |  |  |  |  |  |  |  |  |  |  |  |  |  |  |  |  |  |  |  |  |  |  |  |  |  |  |  |  |  |  |  |  |  |  |  |  |  |  |  |  |  |  |  |  |  |  |  |  |  |  |  |  |  |  |  |  |  |  |  |  |  |  |  |  |  |  |  |  |  |  |  |  |  |  |  |  |  |  |  |  |  |  |  |  |  |  |  |  |  |  |  |  |  |  |  |  |  |  |  |  |  |  |  |  |  |  |  |  |  |  |  |  |  |  |  |  |  |  |  |  |  |  |  |  |  |  |  |  |  |  |  |  |  |  |  |  |  |  |  |  |  |  |  |  |  |  |  |  |  |  |  |  |  |  |  |  |  |  |  |  |  |  |  |  |  |  |  |  |  |  |  |  |  |  |  |  |  |  |  |  |  |  |  |  |  |  |  |  |  |  |  |  |  |  |  |  |  |  |  |  |  |  |  |  |  |  |  |  |  |  |  |  |  |  |  |  |  |  |  |  |  |  |  |  |  |  |  |  |  |  |  |  |  |  |  |  |  |  |  |  |  |  |  |  |  |  |  |  |  |  |  |  |  |  |  |  |  |  |  |  |  |  |  |  |  |  |  |  |  |  |  |  |  |  |  |  |  |  |  |  |  |  |  |  |  |  |  |  |  |  |  |  |  |  |  |  |  |  |  |  |  |  |  |  |  |  |  |  |  |  |  |  |  |  |  |  |  |  |  |  |  |  |  |  |  |  |  |  |  |  |  |  |  |  |  |  |  |  |  |  |  |  |  |  |  |  |  |  |  |  |  |  |  |  |  |  |  |  |  |  |  |  |  |  |  |  |  |  |  |  |  |  |  |  |  |  |  |  |  |  |  |  |  |  |  |  |  |  |  |  |  |  |  |  |  |  |  |  |  |  |  |  |  |  |  |  |  |  |  |  |  |  |  |  |  |  |  |  |  |  |  |  |  |  |  |  |  |  |  |  |  |  |  |  |  |  |  |  |  |  |  |  |  |  |  |  |  |  |  |  |  |  |  |  |  |  |  |  |  |  |  |  |  |  |  |  |  |  |  |  |  |  |  |  |  |  |  |  |  |  |  |  |  |  |  |  |  |  |  |  |  |  |  |  |  |  |  |  |  |  |  |  |  |  |  |  |  |  |  |  |  |  |  |  |  |  |  |  |  |  |  |  |  |  |  |  |  |  |  |  |  |  |  |  |  |  |  |  |  |  |  |  |  |  |  |  |  |  |  |  |  |  |  |  |  |  |  |  |  |  |  |  |  |  |  |  |  |  |  |  |  |  |  |  |  |  |  |  |  |  |  |  |  |  |  |  |  |  |  |  |  |  |  |  |  |  |  |  |  |  |  |  |  |  |  |  |  |  |  |  |  |  |  |  |  |  |  |  |  |  |  |  |  |  |  |  |  |  |  |  |  |  |  |  |  |  |  |  |  |  |  |  |  |  |  |  |  |  |  |  |  |  |  |  |  |  |  |  |  |  |  |  |  |  |  |  |  |  |  |  |  |  |  |  |  |  |  |  |  |  |  |  |  |  |  |  |  |  |  |  |  |  |  |  |  |  |  |  |  |  |  |  |  |  |  |  |  |  |  |  |  |  |  |  |  |  |  |  |  |  |  |  |  |  |  |  |  |  |  |  |  |  |  |  |  |  |  |  |  |  |  |  |  |  |  |  |  |  |  |  |  |  |  |  |  |  |  |  |  |  |  |  |  |  |  |  |  |  |  |  |  |  |  |  |  |  |  |  |  |  |  |  |  |  |  |  |  |  |  |  |  |  |  |  |  |  |  |  |  |  |  |  |  |  |  |  |  |  |  |  |  |  |  |  |  |  |  |  |  |  |  |  |  |  |  |  |  |  |  |  |  |  |  |  |  |  |  |  |  |  |  |  |  |  |  |  |  |  |  |  |  |  |  |  |  |  |  |  |  |  |  |  |  |  |  |  |  |  |  |  |  |  |  |  |  |  |  |  |  |  |  |  |  |  |  |  |  |  |  |  |  |  |  |  |  |  |  |  |  |  |  |  |  |  |  |  |  |  |  |  |  |  |  |  |  |  |  |  |  |  |  |  |  |  |  |  |  |  |  |  |  |  |  |  |  |  |  |  |  |  |  |  |  |  |  |  |  |  |  |  |  |  |  |  |  |  |  |  |  |  |  |  |  |  |  |  |  |  |  |  |  |  |  |  |  |  |  |  |  |  |  |  |  |  |  |  |  |  |  |  |  |  |  |  |  |  |  |  |  |  |  |  |  |  |  |  |  |  |  |  |  |  |  |  |  |  |  |  |  |  |  |  |  |  |  |  |  |  |  |  |  |  |  |  |  |  |  |  |  |  |  |  |  |  |  |  |  |  |  |  |  |  |  |  |  |  |  |  |  |  |  |  |  |  |  |  |  |  |  |  |  |  |  |  |  |  |  |  |  |  |  |  |  |  |  |  |  |  |  |  |  |  |  |  |  |  |  |  |  |  |  |  |  |  |  |  |  |  |  |  |  |  |  |  |  |  |  |  |  |  |  |  |  |  |  |  |  |  |  |  |  |  |  |  |  |  |  |  |  |  |  |  |  |  |  |  |  |  |  |  |  |  |  |  |  |  |  |  |  |  |  |  |  |  |  |  |  |  |  |  |  |  |  |  |  |  |  |  |  |  |  |  |  |  |  |  |  |  |  |  |  |  |  |  |  |  |  |  |  |  |  |  |  |  |  |  |  |  |  |  |  |  |  |  |  |  |  |  |  |  |  |  |  |  |  |  |  |  |  |  |  |  |  |  |  |  |  |  |  |  |  |  |  |  |  |  |  |  |  |  |  |  |  |  |  |  |  |  |  |  |  |  |  |  |  |  |  |  |  |  |  |  |  |  |  |  |  |  |  |  |  |  |  |  |  |  |  |  |  |  |  |  |  |  |  |  |  |  |  |  |  |  |  |  |  |  |  |  |  |  |  |  |  |  |  |  |  |  |  |  |  |  |  |  |  |  |  |  |  |  |  |  |  |  |  |  |  |  |  |  |  |  |  |  |  |  |  |  |  |  |  |  |  |  |  |  |  |  |  |  |  |  |  |  |  |  |  |  |  |  |  |  |  |  |  |  |  |  |  |  |  |  |  |  |  |  |  |  |  |  |  |  |  |  |  |  |  |  |  |  |  |  |  |  |  |  |  |  |  |  |  |  |  |  |  |  |  |  |  |  |  |  |  |  |  |  |  |  |  |  |  |  |  |  |  |  |  |  |  |  |  |  |  |  |  |  |  |  |  |  |  |  |  |  |  |  |  |  |  |  |  |  |  |  |  |  |  |  |  |  |  |  |  |  |  |  |  |  |  |  |  |  |  |  |  |  |  |  |  |  |  |  |  |  |  |  |  |  |  |  |  |  |  |  |  |  |  |  |  |  |  |  |  |  |  |  |  |  |  |  |  |  |  |  |  |  |  |  |  |  |  |  |  |  |  |  |  |  |  |  |  |  |  |  |  |  |  |  |  |  |  |  |  |  |  |  |  |  |  |  |  |  |  |  |  |  |  |  |  |  |  |  |  |  |  |  |  |  |  |  |  |  |  |  |  |  |  |  |  |  |  |  |  |  |  |  |  |  |  |  |  |  |  |  |  |  |  |  |  |  |  |  |  |  |  |  |  |  |  |  |  |  |  |  |  |  |  |  |  |  |  |  |  |  |  |  |  |  |  |  |  |  |  |  |  |  |  |  |  |  |  |  |  |  |  |  |  |  |  |  |  |  |  |  |  |  |  |  |  |  |  |  |  |  |  |  |  |  |  |  |  |  |  |  |  |  |  |  |  |  |  |  |  |  |  |  |  |  |  |  |  |  |  |  |  |  |  |  |  |  |  |  |  |  |  |  |  |  |  |  |  |  |  |  |  |  |  |  |  |  |  |  |  |  |  |  |  |  |  |  |  |  |  |  |  |  |  |  |  |  |  |  |  |  |  |  |  |  |  |  |  |  |  |  |  |  |  |  |  |  |  |  |  |  |  |  |  |  |  |  |  |  |  |  |  |  |  |  |  |  |  |  |  |  |  |  |  |  |  |  |  |  |  |  |  |  |  |  |  |  |  |  |  |  |  |  |  |  |  |  |  |  |  |  |  |  |  |  |  |  |  |  |  |  |  |  |  |  |  |  |  |  |  |  |  |  |  |  |  |  |  |  |  |  |  |  |  |  |  |  |  |  |  |  |  |  |  |  |  |  |  |  |  |  |  |  |  |  |  |  |  |  |  |  |  |  |  |  |  |  |  |  |  |  |  |  |  |  |  |  |  |  |  |  |  |  |  |  |  |  |  |  |  |  |  |  |  |  |  |  |  |  |  |  |  |  |  |  |  |  |  |  |  |  |  |  |  |  |  |  |  |  |  |  |  |  |  |  |  |  |  |  |  |  |  |  |  |  |  |  |  |  |  |  |  |  |  |  |  |  |  |  |  |  |  |  |  |  |  |  |  |  |  |  |  |  |  |  |  |  |  |  |  |  |  |  |  |  |  |  |  |  |  |  |  |  |  |  |  |  |  |  |  |  |  |  |  |  |  |  |  |  |  |  |  |  |  |  |  |  |  |  |  |  |  |  |  |  |  |  |  |  |  |  |  |  |  |  |  |  |  |  |  |  |  |  |  |  |  |  |  |  |  |  |  |  |  |  |  |  |  |  |  |  |  |  |  |  |  |  |  |  |  |  |  |  |  |  |  |  |  |  |  |  |  |  |  |  |  |  |  |  |  |  |  |  |  |  |  |  |  |  |  |  |  |  |  |  |  |  |  |  |  |  |  |  |  |  |  |  |  |  |  |  |  |  |  |  |  |  |  |  |  |  |  |  |  |  |  |  |  |  |  |  |  |  |  |  |  |  |  |  |  |  |  |  |  |  |  |  |  |  |  |  |  |  |  |  |  |  |  |  |  |  |  |  |  |  |  |  |  |  |  |  |  |  |  |  |  |  |  |  |  |  |  |  |  |  |  |  |  |  |  |  |  |  |  |  |  |  |  |  |  |  |  |  |  |  |  |  |  |  |  |  |  |  |  |  |  |  |  |  |  |  |  |  |  |  |  |  |  |  |  |  |  |  |  |  |  |  |  |  |  |  |  |  |  |  |  |  |  |  |  |  |  |  |  |  |  |  |  |  |  |  |  |  |  |  |  |  |  |  |  |  |  |  |  |  |  |  |  |  |  |  |  |  |  |  |  |  |  |  |  |  |  |  |  |  |  |  |  |  |  |  |  |  |  |  |  |  |  |  |  |  |  |  |  |  |  |  |  |  |  |  |  |  |  |  |  |  |  |  |  |  |  |  |  |  |  |  |  |  |  |  |  |  |  |  |  |  |  |  |  |  |  |  |  |  |  |  |  |  |  |  |  |  |  |  |  |  |  |  |  |  |  |  |  |  |  |  |  |  |  |  |  |  |  |  |  |  |  |  |  |  |  |  |  |  |  |  |  |  |  |  |  |  |  |  |  |  |  |  |  |  |  |  |  |  |  |  |  |  |  |  |  |  |  |  |  |  |  |  |  |  |  |  |  |  |  |  |  |  |  |  |  |  |  |  |  |  |  |  |  |  |  |  |  |  |  |  |  |  |  |  |  |  |  |  |  |  |  |  |  |  |  |  |  |  |  |  |  |  |  |  |  |  |  |  |  |  |  |  |  |  |  |  |  |  |  |  |  |  |  |  |  |  |  |  |  |  |  |  |  |  |  |  |  |  |  |  |  |  |  |  |  |  |  |  |  |  |  |  |  |  |  |  |  |  |  |  |  |  |  |  |  |  |  |  |  |  |  |  |  |  |  |  |  |  |  |  |  |  |  |  |  |  |  |  |  |  |  |  |  |  |  |  |  |  |  |  |  |  |  |  |  |  |  |  |  |  |  |  |  |  |  |  |  |  |  |  |  |  |  |  |  |  |  |  |  |  |  |  |  |  |  |  |  |  |  |  |  |  |  |  |  |  |  |  |  |  |  |  |  |  |  |  |  |  |  |  |  |  |  |  |  |  |  |  |  |  |  |  |  |  |  |  |  |  |  |  |  |  |  |  |  |  |  |  |  |  |  |  |  |  |  |  |  |  |  |  |  |  |  |  |  |  |  |  |  |  |  |  |  |  |  |  |  |  |  |  |  |  |  |  |  |  |  |  |  |  |  |  |  |  |  |  |  |  |  |  |  |  |  |  |  |  |  |  |  |  |  |  |  |  |  |  |  |  |  |  |  |  |  |  |  |  |  |  |  |  |  |  |  |  |  |  |  |  |  |  |  |  |  |  |  |  |  |  |  |  |  |  |  |  |  |  |  |  |  |  |  |  |  |  |  |  |  |  |  |  |  |  |  |  |  |  |  |  |  |  |  |  |  |  |  |  |  |  |  |  |  |  |  |  |  |  |  |  |  |  |  |  |  |  |  |  |  |  |  |  |  |  |  |  |  |  |  |  |  |  |  |  |  |  |  |  |  |  |  |  |  |  |  |  |  |  |  |  |  |  |  |  |  |  |  |  |  |  |  |  |  |  |  |  |  |  |  |  |  |  |  |  |  |  |  |  |  |  |  |  |  |  |  |  |  |  |  |  |  |  |  |  |  |  |  |  |  |  |  |  |  |  |  |  |  |  |  |  |  |  |  |  |  |  |  |  |  |  |  |  |  |  |  |  |  |  |  |  |  |  |  |  |  |  |  |  |  |  |  |  |  |  |  |  |  |  |  |  |  |  |  |  |  |  |  |  |  |  |  |  |  |  |  |  |  |  |  |  |  |  |  |  |  |  |  |  |  |  |  |  |  |  |  |  |  |  |  |  |  |  |  |  |  |  |  |  |  |  |  |  |  |  |  |  |  |  |  |  |  |  |  |  |  |  |  |  |  |  |  |  |  |  |  |  |  |  |  |  |  |  |  |  |  |  |  |  |  |  |  |  |  |  |  |  |  |  |  |  |  |  |  |  |  |  |  |  |  |  |  |  |  |  |  |  |  |  |  |  |  |  |  |  |  |  |  |  |  |  |  |  |  |  |  |  |  |  |  |  |  |  |  |  |  |  |  |  |  |  |  |  |  |  |  |  |  |  |  |  |  |  |  |  |  |  |  |  |  |  |  |  |  |  |  |  |  |  |  |  |  |  |  |  |  |  |  |  |  |  |  |  |  |  |  |  |  |  |  |  |  |  |  |  |  |  |  |  |  |  |  |  |  |  |  |  |  |  |  |  |  |  |  |  |  |  |  |  |  |  |  |  |  |  |  |  |  |  |  |  |  |  |  |  |  |  |  |  |  |  |  |  |  |  |  |  |  |  |  |  |  |  |  |  |  |  |  |  |  |  |  |  |  |  |  |  |  |  |  |  |  |  |  |  |  |  |  |  |  |  |  |  |  |  |  |  |  |  |  |  |  |  |  |  |  |  |  |  |  |  |  |  |  |  |  |  |  |  |  |  |  |  |  |  |  |  |  |  |  |  |  |  |  |  |  |  |  |  |  |  |  |  |  |  |  |  |  |  |  |  |  |  |  |  |  |  |  |  |  |  |  |  |  |  |  |  |  |  |  |  |  |  |  |  |  |  |  |  |  |  |  |  |  |  |  |  |  |  |  |  |  |  |  |  |  |  |  |  |  |  |  |  |  |  |  |  |  |  |  |  |  |  |  |  |  |  |  |  |  |  |  |  |  |  |  |  |  |  |  |  |  |  |  |  |  |  |  |  |  |  |  |  |  |  |  |  |  |  |  |  |  |  |  |  |  |  |  |  |  |  |  |  |  |  |  |  |  |  |  |  |  |  |  |  |  |  |  |  |  |  |  |  |  |  |  |  |  |  |  |  |  |  |  |  |  |  |  |  |  |  |  |  |  |  |  |  |  |  |  |  |  |  |  |  |  |  |  |  |  |  |  |  |  |  |  |  |  |  |  |  |  |  |  |  |  |  |  |  |  |  |  |  |  |  |  |  |  |  |  |  |  |  |  |  |  |  |  |  |  |  |  |  |  |  |  |  |  |  |  |  |  |  |  |  |  |  |  |  |  |  |  |  |  |  |  |  |  |  |  |  |  |  |  |  |  |  |  |  |  |  |  |  |  |  |  |  |  |  |  |  |  |  |  |  |  |  |  |  |  |  |  |  |  |  |  |  |  |  |  |  |  |  |  |  |  |  |  |  |  |  |  |  |  |  |  |  |  |  |  |  |  |  |  |  |  |  |  |  |  |  |  |  |  |  |  |  |  |  |  |  |  |  |  |  |  |  |  |  |  |  |  |  |  |  |  |  |  |  |  |  |  |  |  |  |  |  |  |  |  |  |  |  |  |  |  |  |  |  |  |  |  |  |  |  |  |  |  |  |  |  |  |  |  |  |  |  |  |  |  |  |  |  |  |  |  |  |  |  |  |  |  |  |  |  |  |  |  |  |  |  |  |  |  |  |  |  |  |  |  |  |  |  |  |  |  |  |  |  |  |  |  |  |  |  |  |  |  |  |  |  |  |  |  |  |  |  |  |  |  |  |  |  |  |  |  |  |  |  |  |  |  |  |  |  |  |  |  |  |  |  |  |  |  |  |  |  |  |  |  |  |  |  |  |  |  |  |  |  |  |  |  |  |  |  |  |  |  |  |  |  |  |  |  |  |  |  |  |  |  |  |  |  |  |  |  |  |  |  |  |  |  |  |  |  |  |  |  |  |  |  |  |  |  |  |  |  |  |  |  |  |  |  |  |  |  |  |  |  |  |  |  |  |  |  |  |  |  |  |  |  |  |  |  |  |  |  |  |  |  |  |  |  |  |  |  |  |  |  |  |  |  |  |  |  |  |  |  |  |  |  |  |  |  |  |  |  |  |  |  |  |  |  |  |  |  |  |  |  |  |  |  |  |  |  |  |  |  |  |  |  |  |  |  |  |  |  |  |  |  |  |  |  |  |  |  |  |  |  |  |  |  |  |  |  |  |  |  |  |  |  |  |  |  |  |  |  |  |  |  |  |  |  |  |  |  |  |  |  |  |  |  |  |  |  |  |  |  |  |  |  |  |  |  |  |  |  |  |  |  |  |  |  |  |  |  |  |  |  |  |  |  |  |  |  |  |  |  |  |  |  |  |  |  |  |  |  |  |  |  |  |  |  |  |  |  |  |  |  |  |  |  |  |  |  |  |  |  |  |  |  |  |  |  |  |  |  |  |  |  |  |  |  |  |  |  |  |  |  |  |  |  |  |  |  |  |  |  |  |  |  |  |  |  |  |  |  |  |  |  |  |  |  |  |  |  |  |  |  |  |  |  |  |  |  |  |  |  |  |  |  |  |  |  |  |  |  |  |  |  |  |  |  |  |  |  |  |  |  |  |  |  |  |  |  |  |  |  |  |  |  |  |  |  |  |  |  |  |  |  |  |  |  |  |  |  |  |  |  |  |  |  |  |  |  |  |  |  |  |  |  |  |  |  |  |  |  |  |  |  |  |  |  |  |  |  |  |  |  |  |  |  |  |  |  |  |  |  |  |  |  |  |  |  |  |  |  |  |  |  |  |  |  |  |  |  |  |  |  |  |  |  |  |  |  |  |  |  |  |  |  |  |  |  |  |  |  |  |  |  |  |  |  |  |  |  |  |  |  |  |  |  |  |  |  |  |  |  |  |  |  |  |  |  |  |  |  |  |  |  |  |  |  |  |  |  |  |  |  |  |  |  |  |  |  |  |  |  |  |  |  |  |  |  |  |  |  |  |  |  |  |  |  |  |  |  |  |  |  |  |  |  |  |  |  |  |  |  |  |  |  |  |  |  |  |  |  |  |  |  |  |  |  |  |  |  |  |  |  |  |  |  |  |  |  |  |  |  |  |  |  |  |  |  |  |  |  |  |  |  |  |  |  |  |  |  |  |  |  |  |  |  |  |  |  |  |  |  |  |  |  |  |  |  |  |  |  |  |  |  |  |  |  |  |  |  |  |  |  |  |  |  |  |  |  |  |  |  |  |  |  |  |  |  |  |  |  |  |  |  |  |  |  |  |  |  |  |  |  |  |  |  |  |  |  |  |  |  |  |  |  |  |  |  |  |  |  |  |  |  |  |  |  |  |  |  |  |  |  |  |  |  |  |  |  |  |  |  |  |  |  |  |  |  |  |  |  |  |  |  |  |  |  |  |  |  |  |  |  |  |  |  |  |  |  |  |  |  |  |  |  |  |  |  |  |  |  |  |  |  |  |  |  |  |  |  |  |  |  |  |  |  |  |  |  |  |  |  |  |  |  |  |  |  |  |  |  |  |  |  |  |  |  |  |  |  |  |  |  |  |  |  |  |  |  |  |  |  |  |  |  |  |  |  |  |  |  |  |  |  |  |  |  |  |  |  |  |  |  |  |  |  |  |  |  |  |  |  |  |  |  |  |  |  |  |  |  |  |  |  |  |  |  |  |  |  |  |  |  |  |  |  |  |  |  |  |  |  |  |  |  |  |  |  |  |  |  |  |  |  |  |  |  |  |  |  |  |  |  |  |  |  |  |  |  |  |  |  |  |  |  |  |  |  |  |  |  |  |  |  |  |  |  |  |  |  |  |  |  |  |  |  |  |  |  |  |  |  |  |  |  |  |  |  |  |  |  |  |  |  |  |  |  |  |  |  |  |  |  |  |  |  |  |  |  |  |  |  |  |  |  |  |  |  |  |  |  |  |  |  |  |  |  |  |  |  |  |  |  |  |  |  |  |  |  |  |  |  |  |  |  |  |  |  |  |  |  |  |  |  |  |  |  |  |  |  |  |  |  |  |  |  |  |  |  |  |  |  |  |  |  |  |  |  |  |  |  |  |  |  |  |  |  |  |  |  |  |  |  |  |  |  |  |  |  |  |  |  |  |  |  |  |  |  |  |  |  |  |  |  |  |  |  |  |  |  |  |  |  |  |  |  |  |  |  |  |  |  |  |  |  |  |  |  |  |  |  |  |  |  |  |  |  |  |  |  |  |  |  |  |  |  |  |  |  |  |  |  |  |  |  |  |  |  |  |  |  |  |  |  |  |  |  |  |  |  |  |  |  |  |  |  |  |  |  |  |  |  |  |  |  |  |  |  |  |  |  |  |  |  |  |  |  |  |  |  |  |  |  |  |  |  |  |  |  |  |  |  |  |  |  |  |  |  |  |  |  |  |  |  |  |  |  |  |  |  |  |  |  |  |  |  |  |  |  |  |  |  |  |  |  |  |  |  |  |  |  |  |  |  |  |  |  |  |  |  |  |  |  |  |  |  |  |  |  |  |  |  |  |  |  |  |  |  |  |  |  |  |  |  |  |  |  |  |  |  |  |  |  |  |  |  |  |  |  |  |  |  |  |  |  |  |  |  |  |  |  |  |  |  |  |  |  |  |  |  |  |  |  |  |  |  |  |  |  |  |  |  |  |  |  |  |  |  |  |  |  |  |  |  |  |  |  |  |  |  |  |  |  |  |  |  |  |  |  |  |  |  |  |  |  |  |  |  |  |  |  |  |  |  |  |  |  |  |  |  |  |  |  |  |  |  |  |  |  |  |  |  |  |  |  |  |  |  |  |  |  |  |  |  |  |  |  |  |  |  |  |  |  |  |  |  |  |  |  |  |  |  |  |  |  |  |  |  |  |  |  |  |  |  |  |  |  |  |  |  |  |  |  |  |  |  |  |  |  |  |  |  |  |  |  |  |  |  |  |  |  |  |  |  |  |  |  |  |  |  |  |  |  |  |  |  |  |  |  |  |  |  |  |  |  |  |  |  |  |  |  |  |  |  |  |  |  |  |  |  |  |  |  |  |  |  |  |  |  |  |  |  |  |  |  |  |  |  |  |  |  |  |  |  |  |  |  |  |  |  |  |  |  |  |  |  |  |  |  |  |  |  |  |  |  |  |  |  |  |  |  |  |  |  |  |  |  |  |  |  |  |  |  |  |  |  |  |  |  |  |  |  |  |  |  |  |  |  |  |  |  |  |  |  |  |  |  |  |  |  |  |  |  |  |  |  |  |  |  |  |  |  |  |  |  |  |  |  |  |  |  |  |  |  |  |  |  |  |  |  |  |  |  |  |  |  |  |  |  |  |  |  |  |  |  |  |  |  |  |  |  |  |  |  |  |  |  |  |  |  |  |  |  |  |  |  |  |  |  |  |  |  |  |  |  |  |  |  |  |  |  |  |  |  |  |  |  |  |  |  |  |  |  |  |  |  |  |  |  |  |  |  |  |  |  |  |  |  |  |  |  |  |  |  |  |  |  |  |  |  |  |  |  |  |  |  |  |  |  |  |  |  |  |  |  |  |  |  |  |  |  |  |  |  |  |  |  |  |  |  |  |  |  |  |  |  |  |  |  |  |  |  |  |  |  |  |  |  |  |  |  |  |  |  |  |  |  |  |  |  |  |  |  |  |  |  |  |  |  |  |  |  |  |  |  |  |  |  |  |  |  |  |  |  |  |  |  |  |  |  |  |  |  |  |  |  |  |  |  |  |  |  |  |  |  |  |  |  |  |  |  |  |  |  |  |  |  |  |  |  |  |  |  |  |  |  |  |  |  |  |  |  |  |  |  |  |  |  |  |  |  |  |  |  |  |  |  |  |  |  |  |  |  |  |  |  |  |  |  |  |  |  |  |  |  |  |  |  |  |  |  |  |  |  |  |  |  |  |  |  |  |  |  |  |  |  |  |  |  |  |  |  |  |  |  |  |  |  |  |  |  |  |  |  |  |  |  |  |  |  |  |  |  |  |  |  |  |  |  |  |  |  |  |  |  |  |  |  |  |  |  |  |  |  |  |  |  |  |  |  |  |  |  |  |  |  |  |  |  |  |  |  |  |  |  |  |  |  |  |  |  |  |  |  |  |  |  |  |  |  |  |  |  |  |  |  |  |  |  |  |  |  |  |  |  |  |  |  |  |  |  |  |  |  |  |  |  |  |  |  |  |  |  |  |  |  |  |  |  |  |  |  |  |  |  |  |  |  |  |  |  |  |  |  |  |  |  |  |  |  |  |  |  |  |  |  |  |  |  |  |  |  |  |  |  |  |  |  |  |  |  |  |  |  |  |  |  |  |  |  |  |  |  |  |  |  |  |  |  |  |  |  |  |  |  |  |  |  |  |  |  |  |  |  |  |  |  |  |  |  |  |  |  |  |  |  |  |  |  |  |  |  |  |  |  |  |  |  |  |  |  |  |  |  |  |  |  |  |  |  |  |  |  |  |  |  |  |  |  |  |  |  |  |  |  |  |  |  |  |  |  |  |  |  |  |  |  |  |  |  |  |  |  |  |  |  |  |  |  |  |  |  |  |  |  |  |  |  |  |  |  |  |  |  |  |  |  |  |  |  |  |  |  |  |  |  |  |  |  |  |  |  |  |  |  |  |  |  |  |  |  |  |  |  |  |  |  |  |  |  |  |  |  |  |  |  |  |  |  |  |  |  |  |  |  |  |  |  |  |  |  |  |  |  |  |  |  |  |  |  |  |  |  |  |  |  |  |  |  |  |  |  |  |  |  |  |  |  |  |  |  |  |  |  |  |  |  |  |  |  |  |  |  |  |  |  |  |  |  |  |  |  |  |  |  |  |  |  |  |  |  |  |  |  |  |  |  |  |  |  |  |  |  |  |  |  |  |  |  |  |  |  |  |  |  |  |  |  |  |  |  |  |  |  |  |  |  |  |  |  |  |  |  |  |  |  |  |  |  |  |  |  |  |  |  |  |  |  |  |  |  |  |  |  |  |  |  |  |  |  |  |  |  |  |  |  |  |  |  |  |  |  |  |  |  |  |  |  |  |  |  |  |  |  |  |  |  |  |  |  |  |  |  |  |  |  |  |  |  |  |  |  |  |  |  |  |  |  |  |  |  |  |  |  |  |  |  |  |  |  |  |  |  |  |  |  |  |  |  |  |  |  |  |  |  |  |  |  |  |  |  |  |  |  |  |  |  |  |  |  |  |  |  |  |  |  |  |  |  |  |  |  |  |  |  |  |  |  |  |  |  |  |  |  |  |  |  |  |  |  |  |  |  |  |  |  |  |  |  |  |  |  |  |  |  |  |  |  |  |  |  |  |  |  |  |  |  |  |  |  |  |  |  |  |  |  |  |  |  |  |  |  |  |  |  |  |  |  |  |  |  |  |  |  |  |  |  |  |  |  |  |  |  |  |  |  |  |  |  |  |  |  |  |  |  |  |  |  |  |  |  |  |  |  |  |  |  |  |  |  |  |  |  |  |  |  |  |  |  |  |  |  |  |  |  |  |  |  |  |  |  |  |  |  |  |  |  |  |  |  |  |  |  |  |  |  |  |  |  |  |  |  |  |  |  |  |  |  |  |  |  |  |  |  |  |  |  |  |  |  |  |  |  |  |  |  |  |  |  |  |  |  |  |  |  |  |  |  |  |  |  |  |  |  |  |  |  |  |  |  |  |  |  |  |  |  |  |  |  |  |  |  |  |  |  |  |  |  |  |  |  |  |  |  |  |  |  |  |  |  |  |  |  |  |  |  |  |  |  |  |  |  |  |  |  |  |  |  |  |  |  |  |  |  |  |  |  |  |  |  |  |  |  |  |  |  |  |  |  |  |  |  |  |  |  |  |  |  |  |  |  |  |  |  |  |  |  |  |  |  |  |  |  |  |  |  |  |  |  |  |  |  |  |  |  |  |  |  |  |  |  |  |  |  |  |  |  |  |  |  |  |  |  |  |  |  |  |  |  |  |  |  |  |  |  |  |  |  |  |  |  |  |  |  |  |  |  |  |  |  |  |  |  |  |  |  |  |  |  |  |  |  |  |  |  |  |  |  |  |  |  |  |  |  |  |  |  |  |  |  |  |  |  |  |  |  |  |  |  |  |  |  |  |  |  |  |  |  |  |  |  |  |  |  |  |  |  |  |  |  |  |  |  |  |  |  |  |  |  |  |  |  |  |  |  |  |  |  |  |  |  |  |  |  |  |  |  |  |  |  |  |  |  |  |  |  |  |  |  |  |  |  |  |  |  |  |  |  |  |  |  |  |  |  |  |  |  |  |  |  |  |  |  |  |  |  |  |  |  |  |  |  |  |  |  |  |  |  |  |  |  |  |  |  |  |  |  |  |  |  |  |  |  |  |  |  |  |  |  |  |  |  |  |  |  |  |  |  |  |  |  |  |  |  |  |  |  |  |  |  |  |  |  |  |  |  |  |  |  |  |  |  |  |  |  |  |  |  |  |  |  |  |  |  |  |  |  |  |  |  |  |  |  |  |  |  |  |  |  |  |  |  |  |  |  |  |  |  |  |  |  |  |  |  |  |  |  |  |  |  |  |  |  |  |  |  |  |  |  |  |  |  |  |  |  |  |  |  |  |  |  |  |  |  |  |  |  |  |  |  |  |  |  |  |  |  |  |  |  |  |  |  |  |  |  |  |  |  |  |  |  |  |  |  |  |  |  |  |  |  |  |  |  |  |  |  |  |  |  |  |  |  |  |  |  |  |  |  |  |  |  |  |  |  |  |  |  |  |  |  |  |  |  |  |  |  |  |  |  |  |  |  |  |  |  |  |  |  |  |  |  |  |  |  |  |  |  |  |  |  |  |  |  |  |  |  |  |  |  |  |  |  |  |  |  |  |  |  |  |  |  |  |  |  |  |  |  |  |  |  |  |  |  |  |  |  |  |  |  |  |  |  |  |  |  |  |  |  |  |  |  |  |  |  |  |  |  |  |  |  |  |  |  |  |  |  |  |  |  |  |  |  |  |  |  |  |  |  |  |  |  |  |  |  |  |  |  |  |  |  |  |  |  |  |  |  |  |  |  |  |  |  |  |  |  |  |  |  |  |  |  |  |  |  |  |  |  |  |  |  |  |  |  |  |  |  |  |  |  |  |  |  |  |  |  |  |  |  |  |  |  |  |  |  |  |  |  |  |  |  |  |  |  |  |  |  |  |  |  |  |  |  |  |  |  |  |  |  |  |  |  |  |  |  |  |  |  |  |  |  |  |  |  |  |  |  |  |  |  |  |  |  |  |  |  |  |  |  |  |  |  |  |  |  |  |  |  |  |  |  |  |  |  |  |  |  |  |  |  |  |  |  |  |  |  |  |  |  |  |  |  |  |  |  |  |  |  |  |  |  |  |  |  |  |  |  |  |  |  |  |  |  |  |  |  |  |  |  |  |  |  |  |  |  |  |  |  |  |  |  |  |  |  |  |  |  |  |  |  |  |  |  |  |  |  |  |  |  |  |  |  |  |  |  |  |  |  |  |  |  |  |  |  |  |  |  |  |  |  |  |  |  |  |  |  |  |  |  |  |  |  |  |  |  |  |  |  |  |  |  |  |  |  |  |  |  |  |  |  |  |  |  |  |  |  |  |  |  |  |  |  |  |  |  |  |  |  |  |  |  |  |  |  |  |  |  |  |  |  |  |  |  |  |  |  |  |  |  |  |  |  |  |  |  |  |  |  |  |  |  |  |  |  |  |  |  |  |  |  |  |  |  |  |  |  |  |  |  |  |  |  |  |  |  |  |  |  |  |  |  |  |  |  |  |  |  |  |  |  |  |  |  |  |  |  |  |  |  |  |  |  |  |  |  |  |  |  |  |  |  |  |  |  |  |  |  |  |  |  |  |  |  |  |  |  |  |  |  |  |  |  |  |  |  |  |  |  |  |  |  |  |  |  |  |  |  |  |  |  |  |  |  |  |  |  |  |  |  |  |  |  |  |  |  |  |  |  |  |  |  |  |  |  |  |  |  |  |  |  |  |  |  |  |  |  |  |  |  |  |  |  |  |  |  |  |  |  |  |  |  |  |  |  |  |  |  |  |  |  |  |  |  |  |  |  |  |  |  |  |  |  |  |  |  |  |  |  |  |  |  |  |  |  |  |  |  |  |  |  |  |  |  |  |  |  |  |  |  |  |  |  |  |  |  |  |  |  |  |  |  |  |  |  |  |  |  |  |  |  |  |  |  |  |  |  |  |  |  |  |  |  |  |  |  |  |  |  |  |  |  |  |  |  |  |  |  |  |  |  |  |  |  |  |  |  |  |  |  |  |  |  |  |  |  |  |  |  |  |  |  |  |  |  |  |  |  |  |  |  |  |  |  |  |  |  |  |  |  |  |  |  |  |  |  |  |  |  |  |  |  |  |  |  |  |  |  |  |  |  |  |  |  |  |  |  |  |  |  |  |  |  |  |  |  |  |  |  |  |  |  |  |  |  |  |  |  |  |  |  |  |  |  |  |  |  |  |  |  |  |  |  |  |  |  |  |  |  |  |  |  |  |  |  |  |  |  |  |  |  |  |  |  |  |  |  |  |  |  |  |  |  |  |  |  |  |  |  |  |  |  |  |  |  |  |  |  |  |  |  |  |  |  |  |  |  |  |  |  |  |  |  |  |  |  |  |  |  |  |  |  |  |  |  |  |  |  |  |  |  |  |  |  |  |  |  |  |  |  |  |  |  |  |  |  |  |  |  |  |  |  |  |  |  |  |  |  |  |  |  |  |  |  |  |  |  |  |  |  |  |  |  |  |  |  |  |  |  |  |  |  |  |  |  |  |  |  |  |  |  |  |  |  |  |  |  |  |  |  |  |  |  |  |  |  |  |  |  |  |  |  |  |  |  |  |  |  |  |  |  |  |  |  |  |  |  |  |  |  |  |  |  |  |  |  |  |  |  |  |  |  |  |  |  |  |  |  |  |  |  |  |  |  |  |  |  |  |  |  |  |  |  |  |  |  |  |  |  |  |  |  |  |  |  |  |  |  |  |  |  |  |  |  |  |  |  |  |  |  |  |  |  |  |  |  |  |  |  |  |  |  |  |  |  |  |  |  |  |  |  |  |  |  |  |  |  |  |  |  |  |  |  |  |  |  |  |  |  |  |  |  |  |  |  |  |  |  |  |  |  |  |  |  |  |  |  |  |  |  |  |  |  |  |  |  |  |  |  |  |  |  |  |  |  |  |  |  |  |  |  |  |  |  |  |  |  |  |  |  |  |  |  |  |  |  |  |  |  |  |  |  |  |  |  |  |  |  |  |  |  |  |  |  |  |  |  |  |  |  |  |  |  |  |  |  |  |  |  |  |  |  |  |  |  |  |  |  |  |  |  |  |  |  |  |  |  |  |  |  |  |  |  |  |  |  |  |  |  |  |  |  |  |  |  |  |  |  |  |  |  |  |  |  |  |  |  |  |  |  |  |  |  |  |  |  |  |  |  |  |  |  |  |  |  |  |  |  |  |  |  |  |  |  |  |  |  |  |  |  |  |  |  |  |  |  |  |  |  |  |  |  |  |  |  |  |  |  |  |  |  |  |  |  |  |  |  |  |  |  |  |  |  |  |  |  |  |  |  |  |  |  |  |  |  |  |  |  |  |  |  |  |  |  |  |  |  |  |  |  |  |  |  |  |  |  |  |  |  |  |  |  |  |  |  |  |  |  |  |  |  |  |  |  |  |  |  |  |  |  |  |  |  |  |  |  |  |  |  |  |  |  |  |  |  |  |  |  |  |  |  |  |  |  |  |  |  |  |  |  |  |  |  |  |  |  |  |  |  |  |  |  |  |  |  |  |  |  |  |  |  |  |  |  |  |  |  |  |  |  |  |  |  |  |  |  |  |  |  |  |  |  |  |  |  |  |  |  |  |  |  |  |  |  |  |  |  |  |  |  |  |  |  |  |  |  |  |  |  |  |  |  |  |  |  |  |  |  |  |  |  |  |  |  |  |  |  |  |  |  |  |  |  |  |  |  |  |  |  |  |  |  |  |  |  |  |  |  |  |  |  |  |  |  |  |  |  |  |  |  |  |  |  |  |  |  |  |  |  |  |  |  |  |  |  |  |  |  |  |  |  |  |  |  |  |  |  |  |  |  |  |  |  |  |  |  |  |  |  |  |  |  |  |  |  |  |  |  |  |  |  |  |  |  |  |  |  |  |  |  |  |  |  |  |  |  |  |  |  |  |  |  |  |  |  |  |  |  |  |  |  |  |  |  |  |  |  |  |  |  |  |  |  |  |  |  |  |  |  |  |  |  |  |  |  |  |  |  |  |  |  |  |  |  |  |  |  |  |  |  |  |  |  |  |  |  |  |  |  |  |  |  |  |  |  |  |  |  |  |  |  |  |  |  |  |  |  |  |  |  |  |  |  |  |  |  |  |  |  |  |  |  |  |  |  |  |  |  |  |  |  |  |  |  |  |  |  |  |  |  |  |  |  |  |  |  |  |  |  |  |  |  |  |  |  |  |  |  |  |  |  |  |  |  |  |  |  |  |  |  |  |  |  |  |  |  |  |  |  |  |  |  |  |  |  |  |  |  |  |  |  |  |  |  |  |  |  |  |  |  |  |  |  |  |  |  |  |  |  |  |  |  |  |  |  |  |  |  |  |  |  |  |  |  |  |  |  |  |  |  |  |  |  |  |  |  |  |  |  |  |  |  |  |  |  |  |  |  |  |  |  |  |  |  |  |  |  |  |  |  |  |  |  |  |  |  |  |  |  |  |  |  |  |  |  |  |  |  |  |  |  |  |  |  |  |  |  |  |  |  |  |  |  |  |  |  |  |  |  |  |  |  |  |  |  |  |  |  |  |  |  |  |  |  |  |  |  |  |  |  |  |  |  |  |  |  |  |  |  |  |  |  |  |  |  |  |  |  |  |  |  |  |  |  |  |  |  |  |  |  |  |  |  |  |  |  |  |  |  |  |  |  |  |  |  |  |  |  |  |  |  |  |  |  |  |  |  |  |  |  |  |  |  |  |  |  |  |  |  |  |  |  |  |  |  |  |  |  |  |  |  |  |  |  |  |  |  |  |  |  |  |  |  |  |  |  |  |  |  |  |  |  |  |  |  |  |  |  |  |  |  |  |  |  |  |  |  |  |  |  |  |  |  |  |  |  |  |  |  |  |  |  |  |  |  |  |  |  |  |  |  |  |  |  |  |  |  |  |  |  |  |  |  |  |  |  |  |  |  |  |  |  |  |  |  |  |  |  |  |  |  |  |  |  |  |  |  |  |  |  |  |  |  |  |  |  |  |  |  |  |  |  |  |  |  |  |  |  |  |  |  |  |  |  |  |  |  |  |  |  |  |  |  |  |  |  |  |  |  |  |  |  |  |  |  |  |  |  |  |  |  |  |  |  |  |  |  |  |  |  |  |  |  |  |  |  |  |  |  |  |  |  |  |  |  |  |  |  |  |  |  |  |  |  |  |  |  |  |  |  |  |  |  |  |  |  |  |  |  |  |  |  |  |  |  |  |  |  |  |  |  |  |  |  |  |  |  |  |  |  |  |  |  |  |  |  |  |  |  |  |  |  |  |  |  |  |  |  |  |  |  |  |  |  |  |  |  |  |  |  |  |  |  |  |  |  |  |  |  |  |  |  |  |  |  |  |  |  |  |  |  |  |  |  |  |  |  |  |  |  |  |  |  |  |  |  |  |  |  |  |  |  |  |  |  |  |  |  |  |  |  |  |  |  |  |  |  |  |  |  |  |  |  |  |  |  |  |  |  |  |  |  |  |  |  |  |  |  |  |  |  |  |  |  |  |  |  |  |  |  |  |  |  |  |  |  |  |  |  |  |  |  |  |  |  |  |  |  |  |  |  |  |  |  |  |  |  |  |  |  |  |  |  |  |  |  |  |  |  |  |  |  |  |  |  |  |  |  |  |  |  |  |  |  |  |  |  |  |  |  |  |  |  |  |  |  |  |  |  |  |  |  |  |  |  |  |  |  |  |  |  |  |  |  |  |  |  |  |  |  |  |  |  |  |  |  |  |  |  |  |  |  |  |  |  |  |  |  |  |  |  |  |  |  |  |  |  |  |  |  |  |  |  |  |  |  |  |  |  |  |  |  |  |  |  |  |  |  |  |  |  |  |  |  |  |  |  |  |  |  |  |  |  |  |  |  |  |  |  |  |  |  |  |  |  |  |  |  |  |  |  |  |  |  |  |  |  |  |  |  |  |  |  |  |  |  |  |  |  |  |  |  |  |  |  |  |  |  |  |  |  |  |  |  |  |  |  |  |  |  |  |  |  |  |  |  |  |  |  |  |  |  |  |  |  |  |  |  |  |  |  |  |  |  |  |  |  |  |  |  |  |  |  |  |  |  |  |  |  |  |  |  |  |  |  |  |  |  |  |  |  |  |  |  |  |  |  |  |  |  |  |  |  |  |  |  |  |  |  |  |  |  |  |  |  |  |  |  |  |  |  |  |  |  |  |  |  |  |  |  |  |  |  |  |  |  |  |  |  |  |  |  |  |  |  |  |  |  |  |  |  |  |  |  |  |  |  |  |  |  |  |  |  |  |  |  |  |  |  |  |  |  |  |  |  |  |  |  |  |  |  |  |  |  |  |  |  |  |  |  |  |  |  |  |  |  |  |  |  |  |  |  |  |  |  |  |  |  |  |  |  |  |  |  |  |  |  |  |  |  |  |  |  |  |  |  |  |  |  |  |  |  |  |  |  |  |  |  |  |  |  |  |  |  |  |  |  |  |  |  |  |  |  |  |  |  |  |  |  |  |  |  |  |  |  |  |  |  |  |  |  |  |  |  |  |  |  |  |  |  |  |  |  |  |  |  |  |  |  |  |  |  |  |  |  |  |  |
| --- | --- | --- | --- | --- | --- | --- | --- | --- | --- | --- | --- | --- | --- | --- | --- | --- | --- | --- | --- | --- | --- | --- | --- | --- | --- | --- | --- | --- | --- | --- | --- | --- | --- | --- | --- | --- | --- | --- | --- | --- | --- | --- | --- | --- | --- | --- | --- | --- | --- | --- | --- | --- | --- | --- | --- | --- | --- | --- | --- | --- | --- | --- | --- | --- | --- | --- | --- | --- | --- | --- | --- | --- | --- | --- | --- | --- | --- | --- | --- | --- | --- | --- | --- | --- | --- | --- | --- | --- | --- | --- | --- | --- | --- | --- | --- | --- | --- | --- | --- | --- | --- | --- | --- | --- | --- | --- | --- | --- | --- | --- | --- | --- | --- | --- | --- | --- | --- | --- | --- | --- | --- | --- | --- | --- | --- | --- | --- | --- | --- | --- | --- | --- | --- | --- | --- | --- | --- | --- | --- | --- | --- | --- | --- | --- | --- | --- | --- | --- | --- | --- | --- | --- | --- | --- | --- | --- | --- | --- | --- | --- | --- | --- | --- | --- | --- | --- | --- | --- | --- | --- | --- | --- | --- | --- | --- | --- | --- | --- | --- | --- | --- | --- | --- | --- | --- | --- | --- | --- | --- | --- | --- | --- | --- | --- | --- | --- | --- | --- | --- | --- | --- | --- | --- | --- | --- | --- | --- | --- | --- | --- | --- | --- | --- | --- | --- | --- | --- | --- | --- | --- | --- | --- | --- | --- | --- | --- | --- | --- | --- | --- | --- | --- | --- | --- | --- | --- | --- | --- | --- | --- | --- | --- | --- | --- | --- | --- | --- | --- | --- | --- | --- | --- | --- | --- | --- | --- | --- | --- | --- | --- | --- | --- | --- | --- | --- | --- | --- | --- | --- | --- | --- | --- | --- | --- | --- | --- | --- | --- | --- | --- | --- | --- | --- | --- | --- | --- | --- | --- | --- | --- | --- | --- | --- | --- | --- | --- | --- | --- | --- | --- | --- | --- | --- | --- | --- | --- | --- | --- | --- | --- | --- | --- | --- | --- | --- | --- | --- | --- | --- | --- | --- | --- | --- | --- | --- | --- | --- | --- | --- | --- | --- | --- | --- | --- | --- | --- | --- | --- | --- | --- | --- | --- | --- | --- | --- | --- | --- | --- | --- | --- | --- | --- | --- | --- | --- | --- | --- | --- | --- | --- | --- | --- | --- | --- | --- | --- | --- | --- | --- | --- | --- | --- | --- | --- | --- | --- | --- | --- | --- | --- | --- | --- | --- | --- | --- | --- | --- | --- | --- | --- | --- | --- | --- | --- | --- | --- | --- | --- | --- | --- | --- | --- | --- | --- | --- | --- | --- | --- | --- | --- | --- | --- | --- | --- | --- | --- | --- | --- | --- | --- | --- | --- | --- | --- | --- | --- | --- | --- | --- | --- | --- | --- | --- | --- | --- | --- | --- | --- | --- | --- | --- | --- | --- | --- | --- | --- | --- | --- | --- | --- | --- | --- | --- | --- | --- | --- | --- | --- | --- | --- | --- | --- | --- | --- | --- | --- | --- | --- | --- | --- | --- | --- | --- | --- | --- | --- | --- | --- | --- | --- | --- | --- | --- | --- | --- | --- | --- | --- | --- | --- | --- | --- | --- | --- | --- | --- | --- | --- | --- | --- | --- | --- | --- | --- | --- | --- | --- | --- | --- | --- | --- | --- | --- | --- | --- | --- | --- | --- | --- | --- | --- | --- | --- | --- | --- | --- | --- | --- | --- | --- | --- | --- | --- | --- | --- | --- | --- | --- | --- | --- | --- | --- | --- | --- | --- | --- | --- | --- | --- | --- | --- | --- | --- | --- | --- | --- | --- | --- | --- | --- | --- | --- | --- | --- | --- | --- | --- | --- | --- | --- | --- | --- | --- | --- | --- | --- | --- | --- | --- | --- | --- | --- | --- | --- | --- | --- | --- | --- | --- | --- | --- | --- | --- | --- | --- | --- | --- | --- | --- | --- | --- | --- | --- | --- | --- | --- | --- | --- | --- | --- | --- | --- | --- | --- | --- | --- | --- | --- | --- | --- | --- | --- | --- | --- | --- | --- | --- | --- | --- | --- | --- | --- | --- | --- | --- | --- | --- | --- | --- | --- | --- | --- | --- | --- | --- | --- | --- | --- | --- | --- | --- | --- | --- | --- | --- | --- | --- | --- | --- | --- | --- | --- | --- | --- | --- | --- | --- | --- | --- | --- | --- | --- | --- | --- | --- | --- | --- | --- | --- | --- | --- | --- | --- | --- | --- | --- | --- | --- | --- | --- | --- | --- | --- | --- | --- | --- | --- | --- | --- | --- | --- | --- | --- | --- | --- | --- | --- | --- | --- | --- | --- | --- | --- | --- | --- | --- | --- | --- | --- | --- | --- | --- | --- | --- | --- | --- | --- | --- | --- | --- | --- | --- | --- | --- | --- | --- | --- | --- | --- | --- | --- | --- | --- | --- | --- | --- | --- | --- | --- | --- | --- | --- | --- | --- | --- | --- | --- | --- | --- | --- | --- | --- | --- | --- | --- | --- | --- | --- | --- | --- | --- | --- | --- | --- | --- | --- | --- | --- | --- | --- | --- | --- | --- | --- | --- | --- | --- | --- | --- | --- | --- | --- | --- | --- | --- | --- | --- | --- | --- | --- | --- | --- | --- | --- | --- | --- | --- | --- | --- | --- | --- | --- | --- | --- | --- | --- | --- | --- | --- | --- | --- | --- | --- | --- | --- | --- | --- | --- | --- | --- | --- | --- | --- | --- | --- | --- | --- | --- | --- | --- | --- | --- | --- | --- | --- | --- | --- | --- | --- | --- | --- | --- | --- | --- | --- | --- | --- | --- | --- | --- | --- | --- | --- | --- | --- | --- | --- | --- | --- | --- | --- | --- | --- | --- | --- | --- | --- | --- | --- | --- | --- | --- | --- | --- | --- | --- | --- | --- | --- | --- | --- | --- | --- | --- | --- | --- | --- | --- | --- | --- | --- | --- | --- | --- | --- | --- | --- | --- | --- | --- | --- | --- | --- | --- | --- | --- | --- | --- | --- | --- | --- | --- | --- | --- | --- | --- | --- | --- | --- | --- | --- | --- | --- | --- | --- | --- | --- | --- | --- | --- | --- | --- | --- | --- | --- | --- | --- | --- | --- | --- | --- | --- | --- | --- | --- | --- | --- | --- | --- | --- | --- | --- | --- | --- | --- | --- | --- | --- | --- | --- | --- | --- | --- | --- | --- | --- | --- | --- | --- | --- | --- | --- | --- | --- | --- | --- | --- | --- | --- | --- | --- | --- | --- | --- | --- | --- | --- | --- | --- | --- | --- | --- | --- | --- | --- | --- | --- | --- | --- | --- | --- | --- | --- | --- | --- | --- | --- | --- | --- | --- | --- | --- | --- | --- | --- | --- | --- | --- | --- | --- | --- | --- | --- | --- | --- | --- | --- | --- | --- | --- | --- | --- | --- | --- | --- | --- | --- | --- | --- | --- | --- | --- | --- | --- | --- | --- | --- | --- | --- | --- | --- | --- | --- | --- | --- | --- | --- | --- | --- | --- | --- | --- | --- | --- | --- | --- | --- | --- | --- | --- | --- | --- | --- | --- | --- | --- | --- | --- | --- | --- | --- | --- | --- | --- | --- | --- | --- | --- | --- | --- | --- | --- | --- | --- | --- | --- | --- | --- | --- | --- | --- | --- | --- | --- | --- | --- | --- | --- | --- | --- | --- | --- | --- | --- | --- | --- | --- | --- | --- | --- | --- | --- | --- | --- | --- | --- | --- | --- | --- | --- | --- | --- | --- | --- | --- | --- | --- | --- | --- | --- | --- | --- | --- | --- | --- | --- | --- | --- | --- | --- | --- | --- | --- | --- | --- | --- | --- | --- | --- | --- | --- | --- | --- | --- | --- | --- | --- | --- | --- | --- | --- | --- | --- | --- | --- | --- | --- | --- | --- | --- | --- | --- | --- | --- | --- | --- | --- | --- | --- | --- | --- | --- | --- | --- | --- | --- | --- | --- | --- | --- | --- | --- | --- | --- | --- | --- | --- | --- | --- | --- | --- | --- | --- | --- | --- | --- | --- | --- | --- | --- | --- | --- | --- | --- | --- | --- | --- | --- | --- | --- | --- | --- | --- | --- | --- | --- | --- | --- | --- | --- | --- | --- | --- | --- | --- | --- | --- | --- | --- | --- | --- | --- | --- | --- | --- | --- | --- | --- | --- | --- | --- | --- | --- | --- | --- | --- | --- | --- | --- | --- | --- | --- | --- | --- | --- | --- | --- | --- | --- | --- | --- | --- | --- | --- | --- | --- | --- | --- | --- | --- | --- | --- | --- | --- | --- | --- | --- | --- | --- | --- | --- | --- | --- | --- | --- | --- | --- | --- | --- | --- | --- | --- | --- | --- | --- | --- | --- | --- | --- | --- | --- | --- | --- | --- | --- | --- | --- | --- | --- | --- | --- | --- | --- | --- | --- | --- | --- | --- | --- | --- | --- | --- | --- | --- | --- | --- | --- | --- | --- | --- | --- | --- | --- | --- | --- | --- | --- | --- | --- | --- | --- | --- | --- | --- | --- | --- | --- | --- | --- | --- | --- | --- | --- | --- | --- | --- | --- | --- | --- | --- | --- | --- | --- | --- | --- | --- | --- | --- | --- | --- | --- | --- | --- | --- | --- | --- | --- | --- | --- | --- | --- | --- | --- | --- | --- | --- | --- | --- | --- | --- | --- | --- | --- | --- | --- | --- | --- | --- | --- | --- | --- | --- | --- | --- | --- | --- | --- | --- | --- | --- | --- | --- | --- | --- | --- | --- | --- | --- | --- | --- | --- | --- | --- | --- | --- | --- | --- | --- | --- | --- | --- | --- | --- | --- | --- | --- | --- | --- | --- | --- | --- | --- | --- | --- | --- | --- | --- | --- | --- | --- | --- | --- | --- | --- | --- | --- | --- | --- | --- | --- | --- | --- | --- | --- | --- | --- | --- | --- | --- | --- | --- | --- | --- | --- | --- | --- | --- | --- | --- | --- | --- | --- | --- | --- | --- | --- | --- | --- | --- | --- | --- | --- | --- | --- | --- | --- | --- | --- | --- | --- | --- | --- | --- | --- | --- | --- | --- | --- | --- | --- | --- | --- | --- | --- | --- | --- | --- | --- | --- | --- | --- | --- | --- | --- | --- | --- | --- | --- | --- | --- | --- | --- | --- | --- | --- | --- | --- | --- | --- | --- | --- | --- | --- | --- | --- | --- | --- | --- | --- | --- | --- | --- | --- | --- | --- | --- | --- | --- | --- | --- | --- | --- | --- | --- | --- | --- | --- | --- | --- | --- | --- | --- | --- | --- | --- | --- | --- | --- | --- | --- | --- | --- | --- | --- | --- | --- | --- | --- | --- | --- | --- | --- | --- | --- | --- | --- | --- | --- | --- | --- | --- | --- | --- | --- | --- | --- | --- | --- | --- | --- | --- | --- | --- | --- | --- | --- | --- | --- | --- | --- | --- | --- | --- | --- | --- | --- | --- | --- | --- | --- | --- | --- | --- | --- | --- | --- | --- | --- | --- | --- | --- | --- | --- | --- | --- | --- | --- | --- | --- | --- | --- | --- | --- | --- | --- | --- | --- | --- | --- | --- | --- | --- | --- | --- | --- | --- | --- | --- | --- | --- | --- | --- | --- | --- | --- | --- | --- | --- | --- | --- | --- | --- | --- | --- | --- | --- | --- | --- | --- | --- | --- | --- | --- | --- | --- | --- | --- | --- | --- | --- | --- | --- | --- | --- | --- | --- | --- | --- | --- | --- | --- | --- | --- | --- | --- | --- | --- | --- | --- | --- | --- | --- | --- | --- | --- | --- | --- | --- | --- | --- | --- | --- | --- | --- | --- | --- | --- | --- | --- | --- | --- | --- | --- | --- | --- | --- | --- | --- | --- | --- | --- | --- | --- | --- | --- | --- | --- | --- | --- | --- | --- | --- | --- | --- | --- | --- | --- | --- | --- | --- | --- | --- | --- | --- | --- | --- | --- | --- | --- | --- | --- | --- | --- | --- | --- | --- | --- | --- | --- | --- | --- | --- | --- | --- | --- | --- | --- | --- | --- | --- | --- | --- | --- | --- | --- | --- | --- | --- | --- | --- | --- | --- | --- | --- | --- | --- | --- | --- | --- | --- | --- | --- | --- | --- | --- | --- | --- | --- | --- | --- | --- | --- | --- | --- | --- | --- | --- | --- | --- | --- | --- | --- | --- | --- | --- | --- | --- | --- | --- | --- | --- | --- | --- | --- | --- | --- | --- | --- | --- | --- | --- | --- | --- | --- | --- | --- | --- | --- | --- | --- | --- | --- | --- | --- | --- | --- | --- | --- | --- | --- | --- | --- | --- | --- | --- | --- | --- | --- | --- | --- | --- | --- | --- | --- | --- | --- | --- | --- | --- | --- | --- | --- | --- | --- | --- | --- | --- | --- | --- | --- | --- | --- | --- | --- | --- | --- | --- | --- | --- | --- | --- | --- | --- | --- | --- | --- | --- | --- | --- | --- | --- | --- | --- | --- | --- | --- | --- | --- | --- | --- | --- | --- | --- | --- | --- | --- | --- | --- | --- | --- | --- | --- | --- | --- | --- | --- | --- | --- | --- | --- | --- | --- | --- | --- | --- | --- | --- | --- | --- | --- | --- | --- | --- | --- | --- | --- | --- | --- | --- | --- | --- | --- | --- | --- | --- | --- | --- | --- | --- | --- | --- | --- | --- | --- | --- | --- | --- | --- | --- | --- | --- | --- | --- | --- | --- | --- | --- | --- | --- | --- | --- | --- | --- | --- | --- | --- | --- | --- | --- | --- | --- | --- | --- | --- | --- | --- | --- | --- | --- | --- | --- | --- | --- | --- | --- | --- | --- | --- | --- | --- | --- | --- | --- | --- | --- | --- | --- | --- | --- | --- | --- | --- | --- | --- | --- | --- | --- | --- | --- | --- | --- | --- | --- | --- | --- | --- | --- | --- | --- | --- | --- | --- | --- | --- | --- | --- | --- | --- | --- | --- | --- | --- | --- | --- | --- | --- | --- | --- | --- | --- | --- | --- | --- | --- | --- | --- | --- | --- | --- | --- | --- | --- | --- | --- | --- | --- | --- | --- | --- | --- | --- | --- | --- | --- | --- | --- | --- | --- | --- | --- | --- | --- | --- | --- | --- | --- | --- | --- | --- | --- | --- | --- | --- | --- | --- | --- | --- | --- | --- | --- | --- | --- | --- | --- | --- | --- | --- | --- | --- | --- | --- | --- | --- | --- | --- | --- | --- | --- | --- | --- | --- | --- | --- | --- | --- | --- | --- | --- | --- | --- | --- | --- | --- | --- | --- | --- | --- | --- | --- | --- | --- | --- | --- | --- | --- | --- | --- | --- | --- | --- | --- | --- | --- | --- | --- | --- | --- | --- | --- | --- | --- | --- | --- | --- | --- | --- | --- | --- | --- | --- | --- | --- | --- | --- | --- | --- | --- | --- | --- | --- | --- | --- | --- | --- | --- | --- | --- | --- | --- | --- | --- | --- | --- | --- | --- | --- | --- | --- | --- | --- | --- | --- | --- | --- | --- | --- | --- | --- | --- | --- | --- | --- | --- | --- | --- | --- | --- | --- | --- | --- | --- | --- | --- | --- | --- | --- | --- | --- | --- | --- | --- | --- | --- | --- | --- | --- | --- | --- | --- | --- | --- | --- | --- | --- | --- | --- | --- | --- | --- | --- | --- | --- | --- | --- | --- | --- | --- | --- | --- | --- | --- | --- | --- | --- | --- | --- | --- | --- | --- | --- | --- | --- | --- | --- | --- | --- | --- | --- | --- | --- | --- | --- | --- | --- | --- | --- | --- | --- | --- | --- | --- | --- | --- | --- | --- | --- | --- | --- | --- | --- | --- | --- | --- | --- | --- | --- | --- | --- | --- | --- | --- | --- | --- | --- | --- | --- | --- | --- | --- | --- | --- | --- | --- | --- | --- | --- | --- | --- | --- | --- | --- | --- | --- | --- | --- | --- | --- | --- | --- | --- | --- | --- | --- | --- | --- | --- | --- | --- | --- | --- | --- | --- | --- | --- | --- | --- | --- | --- | --- | --- | --- | --- | --- | --- | --- | --- | --- | --- | --- | --- | --- | --- | --- | --- | --- | --- | --- | --- | --- | --- | --- | --- | --- | --- | --- | --- | --- | --- | --- | --- | --- | --- | --- | --- | --- | --- | --- | --- | --- | --- | --- | --- | --- | --- | --- | --- | --- | --- | --- | --- | --- | --- | --- | --- | --- | --- | --- | --- | --- | --- | --- | --- | --- | --- | --- | --- | --- | --- | --- | --- | --- | --- | --- | --- | --- | --- | --- | --- | --- | --- | --- | --- | --- | --- | --- | --- | --- | --- | --- | --- | --- | --- | --- | --- | --- | --- | --- | --- | --- | --- | --- | --- | --- | --- | --- | --- | --- | --- | --- | --- | --- | --- | --- | --- | --- | --- | --- | --- | --- | --- | --- | --- | --- | --- | --- | --- | --- | --- | --- | --- | --- | --- | --- | --- | --- | --- | --- | --- | --- | --- | --- | --- | --- | --- | --- | --- | --- | --- | --- | --- | --- | --- | --- | --- | --- | --- | --- | --- | --- | --- | --- | --- | --- | --- | --- | --- | --- | --- | --- | --- | --- | --- | --- | --- | --- | --- | --- | --- | --- | --- | --- | --- | --- | --- | --- | --- | --- | --- | --- | --- | --- | --- | --- | --- | --- | --- | --- | --- | --- | --- | --- | --- | --- | --- | --- | --- | --- | --- | --- | --- | --- | --- | --- | --- | --- | --- | --- | --- | --- | --- | --- | --- | --- | --- | --- | --- | --- | --- | --- | --- | --- | --- | --- | --- | --- | --- | --- | --- | --- | --- | --- | --- | --- | --- | --- | --- | --- | --- | --- | --- | --- | --- | --- | --- | --- | --- | --- | --- | --- | --- | --- | --- | --- | --- | --- | --- | --- | --- | --- | --- | --- | --- | --- | --- | --- | --- | --- | --- | --- | --- | --- | --- | --- | --- | --- | --- | --- | --- | --- | --- | --- | --- | --- | --- | --- | --- | --- | --- | --- | --- | --- | --- | --- | --- | --- | --- | --- | --- | --- | --- | --- | --- | --- | --- | --- | --- | --- | --- | --- | --- | --- | --- | --- | --- | --- | --- | --- | --- | --- | --- | --- | --- | --- | --- | --- | --- | --- | --- | --- | --- | --- | --- | --- | --- | --- | --- | --- | --- | --- | --- | --- | --- | --- | --- | --- | --- | --- | --- | --- | --- | --- | --- | --- | --- | --- | --- | --- | --- | --- | --- | --- | --- | --- | --- | --- | --- | --- | --- | --- | --- | --- | --- | --- | --- | --- | --- | --- | --- | --- | --- | --- | --- | --- | --- | --- | --- | --- | --- | --- | --- | --- | --- | --- | --- | --- | --- | --- | --- | --- | --- | --- | --- | --- | --- | --- | --- | --- | --- | --- | --- | --- | --- | --- | --- | --- | --- | --- | --- | --- | --- | --- | --- | --- | --- | --- | --- | --- | --- | --- | --- | --- | --- | --- | --- | --- | --- | --- | --- | --- | --- | --- | --- | --- | --- | --- | --- | --- | --- | --- | --- | --- | --- | --- | --- | --- | --- | --- | --- | --- | --- | --- | --- | --- | --- | --- | --- | --- | --- | --- | --- | --- | --- | --- | --- | --- | --- | --- | --- | --- | --- | --- | --- | --- | --- | --- | --- | --- | --- | --- | --- | --- | --- | --- | --- | --- | --- | --- | --- | --- | --- | --- | --- | --- | --- | --- | --- | --- | --- | --- | --- | --- | --- | --- | --- | --- | --- | --- | --- | --- | --- | --- | --- | --- | --- | --- | --- | --- | --- | --- | --- | --- | --- | --- | --- | --- | --- | --- | --- | --- | --- | --- | --- | --- | --- | --- | --- | --- | --- | --- | --- | --- | --- | --- | --- | --- | --- | --- | --- | --- | --- | --- | --- | --- | --- | --- | --- | --- | --- | --- | --- | --- | --- | --- | --- | --- | --- | --- | --- | --- | --- | --- | --- | --- | --- | --- | --- | --- | --- | --- | --- | --- | --- | --- | --- | --- | --- | --- | --- | --- | --- | --- | --- | --- | --- | --- | --- | --- | --- | --- | --- | --- | --- | --- | --- | --- | --- | --- | --- | --- | --- | --- | --- | --- | --- | --- | --- | --- | --- | --- | --- | --- | --- | --- | --- | --- | --- | --- | --- | --- | --- | --- | --- | --- | --- | --- | --- | --- | --- | --- | --- | --- | --- | --- | --- | --- | --- | --- | --- | --- | --- | --- | --- | --- | --- | --- | --- | --- | --- | --- | --- | --- | --- | --- | --- | --- | --- | --- | --- | --- | --- | --- | --- | --- | --- | --- | --- | --- | --- | --- | --- | --- | --- | --- | --- | --- | --- | --- | --- | --- | --- | --- | --- | --- | --- | --- | --- | --- | --- | --- | --- | --- | --- | --- | --- | --- | --- | --- | --- | --- | --- | --- | --- | --- | --- | --- | --- | --- | --- | --- | --- | --- | --- | --- | --- | --- | --- | --- | --- | --- | --- | --- | --- | --- | --- | --- | --- | --- | --- | --- | --- | --- | --- | --- | --- | --- | --- | --- | --- | --- | --- | --- | --- | --- | --- | --- | --- | --- | --- | --- | --- | --- | --- | --- | --- | --- | --- | --- | --- | --- | --- | --- | --- | --- | --- | --- | --- | --- | --- | --- | --- | --- | --- | --- | --- | --- | --- | --- | --- | --- | --- | --- | --- | --- | --- | --- | --- | --- | --- | --- | --- | --- | --- | --- | --- | --- | --- | --- | --- | --- | --- | --- | --- | --- | --- | --- | --- | --- | --- | --- | --- | --- | --- | --- | --- | --- | --- | --- | --- | --- | --- | --- | --- | --- | --- | --- | --- | --- | --- | --- | --- | --- | --- | --- | --- | --- | --- | --- | --- | --- | --- | --- | --- | --- | --- | --- | --- | --- | --- | --- | --- | --- | --- | --- | --- | --- | --- | --- | --- | --- | --- | --- | --- | --- | --- | --- | --- | --- | --- | --- | --- | --- | --- | --- | --- | --- | --- | --- | --- | --- | --- | --- | --- | --- | --- | --- | --- | --- | --- | --- | --- | --- | --- | --- | --- | --- | --- | --- | --- | --- | --- | --- | --- | --- | --- | --- | --- | --- | --- | --- | --- | --- | --- | --- | --- | --- | --- | --- | --- | --- | --- | --- | --- | --- | --- | --- | --- | --- | --- | --- | --- | --- | --- | --- | --- | --- | --- | --- | --- | --- | --- | --- | --- | --- | --- | --- | --- | --- | --- | --- | --- | --- | --- | --- | --- | --- | --- | --- | --- | --- | --- | --- | --- | --- | --- | --- | --- | --- | --- | --- | --- | --- | --- | --- | --- | --- | --- | --- | --- | --- | --- | --- | --- | --- | --- | --- | --- | --- | --- | --- | --- | --- | --- | --- | --- | --- | --- | --- | --- | --- | --- | --- | --- | --- | --- | --- | --- | --- | --- | --- | --- | --- | --- | --- | --- | --- | --- | --- | --- | --- | --- | --- | --- | --- | --- | --- | --- | --- | --- | --- | --- | --- | --- | --- | --- | --- | --- | --- | --- | --- | --- | --- | --- | --- | --- | --- | --- | --- | --- | --- | --- | --- | --- | --- | --- | --- | --- | --- | --- | --- | --- | --- | --- | --- | --- | --- | --- | --- | --- | --- | --- | --- | --- | --- | --- | --- | --- | --- | --- | --- | --- | --- | --- | --- | --- | --- | --- | --- | --- | --- | --- | --- | --- | --- | --- | --- | --- | --- | --- | --- | --- | --- | --- | --- | --- | --- | --- | --- | --- | --- | --- | --- | --- | --- | --- | --- | --- | --- | --- | --- | --- | --- | --- | --- | --- | --- | --- | --- | --- | --- | --- | --- | --- | --- | --- | --- | --- | --- | --- | --- | --- | --- | --- | --- | --- | --- | --- | --- | --- | --- | --- | --- | --- | --- | --- | --- | --- | --- | --- | --- | --- | --- | --- | --- | --- | --- | --- | --- | --- | --- | --- | --- | --- | --- | --- | --- | --- | --- | --- | --- | --- | --- | --- | --- | --- | --- | --- | --- | --- | --- | --- | --- | --- | --- | --- | --- | --- | --- | --- | --- | --- | --- | --- | --- | --- | --- | --- | --- | --- | --- | --- | --- | --- | --- | --- | --- | --- | --- | --- | --- | --- | --- | --- | --- | --- | --- | --- | --- | --- | --- | --- | --- | --- | --- | --- | --- | --- | --- | --- | --- | --- | --- | --- | --- | --- | --- | --- | --- | --- | --- | --- | --- | --- | --- | --- | --- | --- | --- | --- | --- | --- | --- | --- | --- | --- | --- | --- | --- | --- | --- | --- | --- | --- | --- | --- | --- | --- | --- | --- | --- | --- | --- | --- | --- | --- | --- | --- | --- | --- | --- | --- | --- | --- | --- | --- | --- | --- | --- | --- | --- | --- | --- | --- | --- | --- | --- | --- | --- | --- | --- | --- | --- | --- | --- | --- | --- | --- | --- | --- | --- | --- | --- | --- | --- | --- | --- | --- | --- | --- | --- | --- | --- | --- | --- | --- | --- | --- | --- | --- | --- | --- | --- | --- | --- | --- | --- | --- | --- | --- | --- | --- | --- | --- | --- | --- | --- | --- | --- | --- | --- | --- | --- | --- | --- | --- | --- | --- | --- | --- | --- | --- | --- | --- | --- | --- | --- | --- | --- | --- | --- | --- | --- | --- | --- | --- | --- | --- | --- | --- | --- | --- | --- | --- | --- | --- | --- | --- | --- | --- | --- | --- | --- | --- | --- | --- | --- | --- | --- | --- | --- | --- | --- | --- | --- | --- | --- | --- | --- | --- | --- | --- | --- | --- | --- | --- | --- | --- | --- | --- | --- | --- | --- | --- | --- | --- | --- | --- | --- | --- | --- | --- | --- | --- | --- | --- | --- | --- | --- | --- | --- | --- | --- | --- | --- | --- | --- | --- | --- | --- | --- | --- | --- | --- | --- | --- | --- | --- | --- | --- | --- | --- | --- | --- | --- | --- | --- | --- | --- | --- | --- | --- | --- | --- | --- | --- | --- | --- | --- | --- | --- | --- | --- | --- | --- | --- | --- | --- | --- | --- | --- | --- | --- | --- | --- | --- | --- | --- | --- | --- | --- | --- | --- | --- | --- | --- | --- | --- | --- | --- | --- | --- | --- | --- | --- | --- | --- | --- | --- | --- | --- | --- | --- | --- | --- | --- | --- | --- | --- | --- | --- | --- | --- | --- | --- | --- | --- | --- | --- | --- | --- | --- | --- | --- | --- | --- | --- | --- | --- | --- | --- | --- | --- | --- | --- | --- | --- | --- | --- | --- | --- | --- | --- | --- | --- | --- | --- | --- | --- | --- | --- | --- | --- | --- | --- | --- | --- | --- | --- | --- | --- | --- | --- | --- | --- | --- | --- | --- | --- | --- | --- | --- | --- | --- | --- | --- | --- | --- | --- | --- | --- | --- | --- | --- | --- | --- | --- | --- | --- | --- | --- | --- | --- | --- | --- | --- | --- | --- | --- | --- | --- | --- | --- | --- | --- | --- | --- | --- | --- | --- | --- | --- | --- | --- | --- | --- | --- | --- | --- | --- | --- | --- | --- | --- | --- | --- | --- | --- | --- | --- | --- | --- | --- | --- | --- | --- | --- | --- | --- | --- | --- | --- | --- | --- | --- | --- | --- | --- | --- | --- | --- | --- | --- | --- | --- | --- | --- | --- | --- | --- | --- | --- | --- | --- | --- | --- | --- | --- | --- | --- | --- | --- | --- | --- | --- | --- | --- | --- | --- | --- | --- | --- | --- | --- | --- | --- | --- | --- | --- | --- | --- | --- | --- | --- | --- | --- | --- | --- | --- | --- | --- | --- | --- | --- | --- | --- | --- | --- | --- | --- | --- | --- | --- | --- | --- | --- | --- | --- | --- | --- | --- | --- | --- | --- | --- | --- | --- | --- | --- | --- | --- | --- | --- | --- | --- | --- | --- | --- | --- | --- | --- | --- | --- | --- | --- | --- | --- | --- | --- | --- | --- | --- | --- | --- | --- | --- | --- | --- | --- | --- | --- | --- | --- | --- | --- | --- | --- | --- | --- | --- | --- | --- | --- | --- | --- | --- | --- | --- | --- | --- | --- | --- | --- | --- | --- | --- | --- | --- | --- | --- | --- | --- | --- | --- | --- | --- | --- | --- | --- | --- | --- | --- | --- | --- | --- | --- | --- | --- | --- | --- | --- | --- | --- | --- | --- | --- | --- | --- | --- | --- | --- | --- | --- | --- | --- | --- | --- | --- | --- | --- | --- | --- | --- | --- | --- | --- | --- | --- | --- | --- | --- | --- | --- | --- | --- | --- | --- | --- | --- | --- | --- | --- | --- | --- | --- | --- | --- | --- | --- | --- | --- | --- | --- | --- | --- | --- | --- | --- | --- | --- | --- | --- | --- | --- | --- | --- | --- | --- | --- | --- | --- | --- | --- | --- | --- | --- | --- | --- | --- | --- | --- | --- | --- | --- | --- | --- | --- | --- | --- | --- | --- | --- | --- | --- | --- | --- | --- | --- | --- | --- | --- | --- | --- | --- | --- | --- | --- | --- | --- | --- | --- | --- | --- | --- | --- | --- | --- | --- | --- | --- | --- | --- | --- | --- | --- | --- | --- | --- | --- | --- | --- | --- | --- | --- | --- | --- | --- | --- | --- | --- | --- | --- | --- | --- | --- | --- | --- | --- | --- | --- | --- | --- | --- | --- | --- | --- | --- | --- | --- | --- | --- | --- | --- | --- | --- | --- | --- | --- | --- | --- | --- | --- | --- | --- | --- | --- | --- | --- | --- | --- | --- | --- | --- | --- | --- | --- | --- | --- | --- | --- | --- | --- | --- | --- | --- | --- | --- | --- | --- | --- | --- | --- | --- | --- | --- | --- | --- | --- | --- | --- | --- | --- | --- | --- | --- | --- | --- | --- | --- | --- | --- | --- | --- | --- | --- | --- | --- | --- | --- | --- | --- | --- | --- | --- | --- | --- | --- | --- | --- | --- | --- | --- | --- | --- | --- | --- | --- | --- | --- | --- | --- | --- | --- | --- | --- | --- | --- | --- | --- | --- | --- | --- | --- | --- | --- | --- | --- | --- | --- | --- | --- | --- | --- | --- | --- | --- | --- | --- | --- | --- | --- | --- | --- | --- | --- | --- | --- | --- | --- | --- | --- | --- | --- | --- | --- | --- | --- | --- | --- | --- | --- | --- | --- | --- | --- | --- | --- | --- | --- | --- | --- | --- | --- | --- | --- | --- | --- | --- | --- | --- | --- | --- | --- | --- | --- | --- | --- | --- | --- | --- | --- | --- | --- | --- | --- | --- | --- | --- | --- | --- | --- | --- | --- | --- | --- | --- | --- | --- | --- | --- | --- | --- | --- | --- | --- | --- | --- | --- | --- | --- | --- | --- | --- | --- | --- | --- | --- | --- | --- | --- | --- | --- | --- | --- | --- | --- | --- | --- | --- | --- | --- | --- | --- | --- | --- | --- | --- | --- | --- | --- | --- | --- | --- | --- | --- | --- | --- | --- | --- | --- | --- | --- | --- | --- | --- | --- | --- | --- | --- | --- | --- | --- | --- | --- | --- | --- | --- | --- | --- | --- | --- | --- | --- | --- | --- | --- | --- | --- | --- | --- | --- | --- | --- | --- | --- | --- | --- | --- | --- | --- | --- | --- | --- | --- | --- | --- | --- | --- | --- | --- | --- | --- | --- | --- | --- | --- | --- | --- | --- | --- | --- | --- | --- | --- | --- | --- | --- | --- | --- | --- | --- | --- | --- | --- | --- | --- | --- | --- | --- | --- | --- | --- | --- | --- | --- | --- | --- | --- | --- | --- | --- | --- | --- | --- | --- | --- | --- | --- | --- | --- | --- | --- | --- | --- | --- | --- | --- | --- | --- | --- | --- | --- | --- | --- | --- | --- | --- | --- | --- | --- | --- | --- | --- | --- | --- | --- | --- | --- | --- | --- | --- | --- | --- | --- | --- | --- | --- | --- | --- | --- | --- | --- | --- | --- | --- | --- | --- | --- | --- | --- | --- | --- | --- | --- | --- | --- | --- | --- | --- | --- | --- | --- | --- | --- | --- | --- | --- | --- | --- | --- | --- | --- | --- | --- | --- | --- | --- | --- | --- | --- | --- | --- | --- | --- | --- | --- | --- | --- | --- | --- | --- | --- | --- | --- | --- | --- | --- | --- | --- | --- | --- | --- | --- | --- | --- | --- | --- | --- | --- | --- | --- | --- | --- | --- | --- | --- | --- | --- | --- | --- | --- | --- | --- | --- | --- | --- | --- | --- | --- | --- | --- | --- | --- | --- | --- | --- | --- | --- | --- | --- | --- | --- | --- | --- | --- | --- | --- | --- | --- | --- | --- | --- | --- | --- | --- | --- | --- | --- | --- | --- | --- | --- | --- | --- | --- | --- | --- | --- | --- | --- | --- | --- | --- | --- | --- | --- | --- | --- | --- | --- | --- | --- | --- | --- | --- | --- | --- | --- | --- | --- | --- | --- | --- | --- | --- | --- | --- | --- | --- | --- | --- | --- | --- | --- | --- | --- | --- | --- | --- | --- | --- | --- | --- | --- | --- | --- | --- | --- | --- | --- | --- | --- | --- | --- | --- | --- | --- | --- | --- | --- | --- | --- | --- | --- | --- | --- | --- | --- | --- | --- | --- | --- | --- | --- | --- | --- | --- | --- | --- | --- | --- | --- | --- | --- | --- | --- | --- | --- | --- | --- | --- | --- | --- | --- | --- | --- | --- | --- | --- | --- | --- | --- | --- | --- | --- | --- | --- | --- | --- | --- | --- | --- | --- | --- | --- | --- | --- | --- | --- | --- | --- | --- | --- | --- | --- | --- | --- | --- | --- | --- | --- | --- | --- | --- | --- | --- | --- | --- | --- | --- | --- | --- | --- | --- | --- | --- | --- | --- | --- | --- | --- | --- | --- | --- | --- | --- | --- | --- | --- | --- | --- | --- | --- | --- | --- | --- | --- | --- | --- | --- | --- | --- | --- | --- | --- | --- | --- | --- | --- | --- | --- | --- | --- | --- | --- | --- | --- | --- | --- | --- | --- | --- | --- | --- | --- | --- | --- | --- | --- | --- | --- | --- | --- | --- | --- | --- | --- | --- | --- | --- | --- | --- | --- | --- | --- | --- | --- | --- | --- | --- | --- | --- | --- | --- | --- | --- | --- | --- | --- | --- | --- | --- | --- | --- | --- | --- | --- | --- | --- | --- | --- | --- | --- | --- | --- | --- | --- | --- | --- | --- | --- | --- | --- | --- | --- | --- | --- | --- | --- | --- | --- | --- | --- | --- | --- | --- | --- | --- | --- | --- | --- | --- | --- | --- | --- | --- | --- | --- | --- | --- | --- | --- | --- | --- | --- | --- | --- | --- | --- | --- | --- | --- | --- | --- | --- | --- | --- | --- | --- | --- | --- | --- | --- | --- | --- | --- | --- | --- | --- | --- | --- | --- | --- | --- | --- | --- | --- | --- | --- | --- | --- | --- | --- | --- | --- | --- | --- | --- | --- | --- | --- | --- | --- | --- | --- | --- | --- | --- | --- | --- | --- | --- | --- | --- | --- | --- | --- | --- | --- | --- | --- | --- | --- | --- | --- | --- | --- | --- | --- | --- | --- | --- | --- | --- | --- | --- | --- | --- | --- | --- | --- | --- | --- | --- | --- | --- | --- | --- | --- | --- | --- | --- | --- | --- | --- | --- | --- | --- | --- | --- | --- | --- | --- | --- | --- | --- | --- | --- | --- | --- | --- | --- | --- | --- | --- | --- | --- | --- | --- | --- | --- | --- | --- | --- | --- | --- | --- | --- | --- | --- | --- | --- | --- | --- | --- | --- | --- | --- | --- | --- | --- | --- | --- | --- | --- | --- | --- | --- | --- | --- | --- | --- | --- | --- | --- | --- | --- | --- | --- | --- | --- | --- | --- | --- | --- | --- | --- | --- | --- | --- | --- | --- | --- | --- | --- | --- | --- | --- | --- | --- | --- | --- | --- | --- | --- | --- | --- | --- | --- | --- | --- | --- | --- | --- | --- | --- | --- | --- | --- | --- | --- | --- | --- | --- | --- | --- | --- | --- | --- | --- | --- | --- | --- | --- | --- | --- | --- | --- | --- | --- | --- | --- | --- | --- | --- | --- | --- | --- | --- | --- | --- | --- | --- | --- | --- | --- | --- | --- | --- | --- | --- | --- | --- | --- | --- | --- | --- | --- | --- | --- | --- | --- | --- | --- | --- | --- | --- | --- | --- | --- | --- | --- | --- | --- | --- | --- | --- | --- | --- | --- | --- | --- | --- | --- | --- | --- | --- | --- | --- | --- | --- | --- | --- | --- | --- | --- | --- | --- | --- | --- | --- | --- | --- | --- | --- | --- | --- | --- | --- | --- | --- | --- | --- | --- | --- | --- | --- | --- | --- | --- | --- | --- | --- | --- | --- | --- | --- | --- | --- | --- | --- | --- | --- | --- | --- | --- | --- | --- | --- | --- | --- | --- | --- | --- | --- | --- | --- | --- | --- | --- | --- | --- | --- | --- | --- | --- | --- | --- | --- | --- | --- | --- | --- | --- | --- | --- | --- | --- | --- | --- | --- | --- | --- | --- | --- | --- | --- | --- | --- | --- | --- | --- | --- | --- | --- | --- | --- | --- | --- | --- | --- | --- | --- | --- | --- | --- | --- | --- | --- | --- | --- | --- | --- | --- | --- | --- | --- | --- | --- | --- | --- | --- | --- | --- | --- | --- | --- | --- | --- | --- | --- | --- | --- | --- | --- | --- | --- | --- | --- | --- | --- | --- | --- | --- | --- | --- | --- | --- | --- | --- | --- | --- | --- | --- | --- | --- | --- | --- | --- | --- | --- | --- | --- | --- | --- | --- | --- | --- | --- | --- | --- | --- | --- | --- | --- | --- | --- | --- | --- | --- | --- | --- | --- | --- | --- | --- | --- | --- | --- | --- | --- | --- | --- | --- | --- | --- | --- | --- | --- | --- | --- | --- | --- | --- | --- | --- | --- | --- | --- | --- | --- | --- | --- | --- | --- | --- | --- | --- | --- | --- | --- | --- | --- | --- | --- | --- | --- | --- | --- | --- | --- | --- | --- | --- | --- | --- | --- | --- | --- | --- | --- | --- | --- | --- | --- | --- | --- | --- | --- | --- | --- | --- | --- | --- | --- | --- | --- | --- | --- | --- | --- | --- | --- | --- | --- | --- | --- | --- | --- | --- | --- | --- | --- | --- | --- | --- | --- | --- | --- | --- | --- | --- | --- | --- | --- | --- | --- | --- | --- | --- | --- | --- | --- | --- | --- | --- | --- | --- | --- | --- | --- | --- | --- | --- | --- | --- | --- | --- | --- | --- | --- | --- | --- | --- | --- | --- | --- | --- | --- | --- | --- | --- | --- | --- | --- | --- | --- | --- | --- | --- | --- | --- | --- | --- | --- | --- | --- | --- | --- | --- | --- | --- | --- | --- | --- | --- | --- | --- | --- | --- | --- | --- | --- | --- | --- | --- | --- | --- | --- | --- | --- | --- | --- | --- | --- | --- | --- | --- | --- | --- | --- | --- | --- | --- | --- | --- | --- | --- | --- | --- | --- | --- | --- | --- | --- | --- | --- | --- | --- | --- | --- | --- | --- | --- | --- | --- | --- | --- | --- | --- | --- | --- | --- | --- | --- | --- | --- | --- | --- | --- | --- | --- | --- | --- | --- | --- | --- | --- | --- | --- | --- | --- | --- | --- | --- | --- | --- | --- | --- | --- | --- | --- | --- | --- | --- | --- | --- | --- | --- | --- | --- | --- | --- | --- | --- | --- | --- | --- | --- | --- | --- | --- | --- | --- | --- | --- | --- | --- | --- | --- | --- | --- | --- | --- | --- | --- | --- | --- | --- | --- | --- | --- | --- | --- | --- | --- | --- | --- | --- | --- | --- | --- | --- | --- | --- | --- | --- | --- | --- | --- | --- | --- | --- | --- | --- | --- | --- | --- | --- | --- | --- | --- | --- | --- | --- | --- | --- | --- | --- | --- | --- | --- | --- | --- | --- | --- | --- | --- | --- | --- | --- | --- | --- | --- | --- | --- | --- | --- | --- | --- | --- | --- | --- | --- | --- | --- | --- | --- | --- | --- | --- | --- | --- | --- | --- | --- | --- | --- | --- | --- | --- | --- | --- | --- | --- | --- | --- | --- | --- | --- | --- | --- | --- | --- | --- | --- | --- | --- | --- | --- | --- | --- | --- | --- | --- | --- | --- | --- | --- | --- | --- | --- | --- | --- | --- | --- | --- | --- | --- | --- | --- | --- | --- | --- | --- | --- | --- | --- | --- | --- | --- | --- | --- | --- | --- | --- | --- | --- | --- | --- | --- | --- | --- | --- | --- | --- | --- | --- | --- | --- | --- | --- | --- | --- | --- | --- | --- | --- | --- | --- | --- | --- | --- | --- | --- | --- | --- | --- | --- | --- | --- | --- | --- | --- | --- | --- | --- | --- | --- | --- | --- | --- | --- | --- | --- | --- | --- | --- | --- | --- | --- | --- | --- | --- | --- | --- | --- | --- | --- | --- | --- | --- | --- | --- | --- | --- | --- | --- | --- | --- | --- | --- | --- | --- | --- | --- | --- | --- | --- | --- | --- | --- | --- | --- | --- | --- | --- | --- | --- | --- | --- | --- | --- | --- | --- | --- | --- | --- | --- | --- | --- | --- | --- | --- | --- | --- | --- | --- | --- | --- | --- | --- | --- | --- | --- | --- | --- | --- | --- | --- | --- | --- | --- | --- | --- | --- | --- | --- | --- | --- | --- | --- | --- | --- | --- | --- | --- | --- | --- | --- | --- | --- | --- | --- | --- | --- | --- | --- | --- | --- | --- | --- | --- | --- | --- | --- | --- | --- | --- | --- | --- | --- | --- | --- | --- | --- | --- | --- | --- | --- | --- | --- | --- | --- | --- | --- | --- | --- | --- | --- | --- | --- | --- | --- | --- | --- | --- | --- | --- | --- | --- | --- | --- | --- | --- | --- | --- | --- | --- | --- | --- | --- | --- | --- | --- | --- | --- | --- | --- | --- | --- | --- | --- | --- | --- | --- | --- | --- | --- | --- | --- | --- | --- | --- | --- | --- | --- | --- | --- | --- | --- | --- | --- | --- | --- | --- | --- | --- | --- | --- | --- | --- | --- | --- | --- | --- | --- | --- | --- | --- | --- | --- | --- | --- | --- | --- | --- | --- | --- | --- | --- | --- | --- | --- | --- | --- | --- | --- | --- | --- | --- | --- | --- | --- | --- | --- | --- | --- | --- | --- | --- | --- | --- | --- | --- | --- | --- | --- | --- | --- | --- | --- | --- | --- | --- | --- | --- | --- | --- | --- | --- | --- | --- | --- | --- | --- | --- | --- | --- | --- | --- | --- | --- | --- | --- | --- | --- | --- | --- | --- | --- | --- | --- | --- | --- | --- | --- | --- | --- | --- | --- | --- | --- | --- | --- | --- | --- | --- | --- | --- | --- | --- | --- | --- | --- | --- | --- | --- | --- | --- | --- | --- | --- | --- | --- | --- | --- | --- | --- | --- | --- | --- | --- | --- | --- | --- | --- | --- | --- | --- | --- | --- | --- | --- | --- | --- | --- | --- | --- | --- | --- | --- | --- | --- | --- | --- | --- | --- | --- | --- | --- | --- | --- | --- | --- | --- | --- | --- | --- | --- | --- | --- | --- | --- | --- | --- | --- | --- | --- | --- | --- | --- | --- | --- | --- | --- | --- | --- | --- | --- | --- | --- | --- | --- | --- | --- | --- | --- | --- | --- | --- | --- | --- | --- | --- | --- | --- | --- | --- | --- | --- | --- | --- | --- | --- | --- | --- | --- | --- | --- | --- | --- | --- | --- | --- | --- | --- | --- | --- | --- | --- | --- | --- | --- | --- | --- | --- | --- | --- | --- | --- | --- | --- | --- | --- | --- | --- | --- | --- | --- | --- | --- | --- | --- | --- | --- | --- | --- | --- | --- | --- | --- | --- | --- | --- | --- | --- | --- | --- | --- | --- | --- | --- | --- | --- | --- | --- | --- | --- | --- | --- | --- | --- | --- | --- | --- | --- | --- | --- | --- | --- | --- | --- | --- | --- | --- | --- | --- | --- | --- | --- | --- | --- | --- | --- | --- | --- | --- | --- | --- | --- | --- | --- | --- | --- | --- | --- | --- | --- | --- | --- | --- | --- | --- | --- | --- | --- | --- | --- | --- | --- | --- | --- | --- | --- | --- | --- | --- | --- | --- | --- | --- | --- | --- | --- | --- | --- | --- | --- | --- | --- | --- | --- | --- | --- | --- | --- | --- | --- | --- | --- | --- | --- | --- | --- | --- | --- | --- | --- | --- | --- | --- | --- | --- | --- | --- | --- | --- | --- | --- | --- | --- | --- | --- | --- | --- | --- | --- | --- | --- | --- | --- | --- | --- | --- | --- | --- | --- | --- | --- | --- | --- | --- | --- | --- | --- | --- | --- | --- | --- | --- | --- | --- | --- | --- | --- | --- | --- | --- | --- | --- | --- | --- | --- | --- | --- | --- | --- | --- | --- | --- | --- | --- | --- | --- | --- | --- | --- | --- | --- | --- | --- | --- | --- | --- | --- | --- | --- | --- | --- | --- | --- | --- | --- | --- | --- | --- | --- | --- | --- | --- | --- | --- | --- | --- | --- | --- | --- | --- | --- | --- | --- | --- | --- | --- | --- | --- | --- | --- | --- | --- | --- | --- | --- | --- | --- | --- | --- | --- | --- | --- | --- | --- | --- | --- | --- | --- | --- | --- | --- | --- | --- | --- | --- | --- | --- | --- | --- | --- | --- | --- | --- | --- | --- | --- | --- | --- | --- | --- | --- | --- | --- | --- | --- | --- | --- | --- | --- | --- | --- | --- | --- | --- | --- | --- | --- | --- | --- | --- | --- | --- | --- | --- | --- | --- | --- | --- | --- | --- | --- | --- | --- | --- | --- | --- | --- | --- | --- | --- | --- | --- | --- | --- | --- | --- | --- | --- | --- | --- | --- | --- | --- | --- | --- | --- | --- | --- | --- | --- | --- | --- | --- | --- | --- | --- | --- | --- | --- | --- | --- | --- | --- | --- | --- | --- | --- | --- | --- | --- | --- | --- | --- | --- | --- | --- | --- | --- | --- | --- | --- | --- | --- | --- | --- | --- | --- | --- | --- | --- | --- | --- | --- | --- | --- | --- | --- | --- | --- | --- | --- | --- | --- | --- | --- | --- | --- | --- | --- | --- | --- | --- | --- | --- | --- | --- | --- | --- | --- | --- | --- | --- | --- | --- | --- | --- | --- | --- | --- | --- | --- | --- | --- | --- | --- | --- | --- | --- | --- | --- | --- | --- | --- | --- | --- | --- | --- | --- | --- | --- | --- | --- | --- | --- | --- | --- | --- | --- | --- | --- | --- | --- | --- | --- | --- | --- | --- | --- | --- | --- | --- | --- | --- | --- | --- | --- | --- | --- | --- | --- | --- | --- | --- | --- | --- | --- | --- | --- | --- | --- | --- | --- | --- | --- | --- | --- | --- | --- | --- | --- | --- | --- | --- | --- | --- | --- | --- | --- | --- | --- | --- | --- | --- | --- | --- | --- | --- | --- | --- | --- | --- | --- | --- | --- | --- | --- | --- | --- | --- | --- | --- | --- | --- | --- | --- | --- | --- | --- | --- | --- | --- | --- | --- | --- | --- | --- | --- | --- | --- | --- | --- | --- | --- | --- | --- | --- | --- | --- | --- | --- | --- | --- | --- | --- | --- | --- | --- | --- | --- | --- | --- | --- | --- | --- | --- | --- | --- | --- | --- | --- | --- | --- | --- | --- | --- | --- | --- | --- | --- | --- | --- | --- | --- | --- | --- | --- | --- | --- | --- | --- | --- | --- | --- | --- | --- | --- | --- | --- | --- | --- | --- | --- | --- | --- | --- | --- | --- | --- | --- | --- | --- | --- | --- | --- | --- | --- | --- | --- | --- | --- | --- | --- | --- | --- | --- | --- | --- | --- | --- | --- | --- | --- | --- | --- | --- | --- | --- | --- | --- | --- | --- | --- | --- | --- | --- | --- | --- | --- | --- | --- | --- | --- | --- | --- | --- | --- | --- | --- | --- | --- | --- | --- | --- | --- | --- | --- | --- | --- | --- | --- | --- | --- | --- | --- | --- | --- | --- | --- | --- | --- | --- | --- | --- | --- | --- | --- | --- | --- | --- | --- | --- | --- | --- | --- | --- | --- | --- | --- | --- | --- | --- | --- | --- | --- | --- | --- | --- | --- | --- | --- | --- | --- | --- | --- | --- | --- | --- | --- | --- | --- | --- | --- | --- | --- | --- | --- | --- | --- | --- | --- | --- | --- | --- | --- | --- | --- | --- | --- | --- | --- | --- | --- | --- | --- | --- | --- | --- | --- | --- | --- | --- | --- | --- | --- | --- | --- | --- | --- | --- | --- | --- | --- | --- | --- | --- | --- | --- | --- | --- | --- | --- | --- | --- | --- | --- | --- | --- | --- | --- | --- | --- | --- | --- | --- | --- | --- | --- | --- | --- | --- | --- | --- | --- | --- | --- | --- | --- | --- | --- | --- | --- | --- | --- | --- | --- | --- | --- | --- | --- | --- | --- | --- | --- | --- | --- | --- | --- | --- | --- | --- | --- | --- | --- | --- | --- | --- | --- | --- | --- | --- | --- | --- | --- | --- | --- | --- | --- | --- | --- | --- | --- | --- | --- | --- | --- | --- | --- | --- | --- | --- | --- | --- | --- | --- | --- | --- | --- | --- | --- | --- | --- | --- | --- | --- | --- | --- | --- | --- | --- | --- | --- | --- | --- | --- | --- | --- | --- | --- | --- | --- | --- | --- | --- | --- | --- | --- | --- | --- | --- | --- | --- | --- | --- | --- | --- | --- | --- | --- | --- | --- | --- | --- | --- | --- | --- | --- | --- | --- | --- | --- | --- | --- | --- | --- | --- | --- | --- | --- | --- | --- | --- | --- | --- | --- | --- | --- | --- | --- | --- | --- | --- | --- | --- | --- | --- | --- | --- | --- | --- | --- | --- | --- | --- | --- | --- | --- | --- | --- | --- | --- | --- | --- | --- | --- | --- | --- | --- | --- | --- | --- | --- | --- | --- | --- | --- | --- | --- | --- | --- | --- | --- | --- | --- | --- | --- | --- | --- | --- | --- | --- | --- | --- | --- | --- | --- | --- | --- | --- | --- | --- | --- | --- | --- | --- | --- | --- | --- | --- | --- | --- | --- | --- | --- | --- | --- | --- | --- | --- | --- | --- | --- | --- | --- | --- | --- | --- | --- | --- | --- | --- | --- | --- | --- | --- | --- | --- | --- | --- | --- | --- | --- | --- | --- | --- | --- | --- | --- | --- | --- | --- | --- | --- | --- | --- | --- | --- | --- | --- | --- | --- | --- | --- | --- | --- | --- | --- | --- | --- | --- | --- | --- | --- | --- | --- | --- | --- | --- | --- | --- | --- | --- | --- | --- | --- | --- | --- | --- | --- | --- | --- | --- | --- | --- | --- | --- | --- | --- | --- | --- | --- | --- | --- | --- | --- | --- | --- | --- | --- | --- | --- | --- | --- | --- | --- | --- | --- | --- | --- | --- | --- | --- | --- | --- | --- | --- | --- | --- | --- | --- | --- | --- | --- | --- | --- | --- | --- | --- | --- | --- | --- | --- | --- | --- | --- | --- | --- | --- | --- | --- | --- | --- | --- | --- | --- | --- | --- | --- | --- | --- | --- | --- | --- | --- | --- | --- | --- | --- | --- | --- | --- | --- | --- | --- | --- | --- | --- | --- | --- | --- | --- | --- | --- | --- | --- | --- | --- | --- | --- | --- | --- | --- | --- | --- | --- | --- | --- | --- | --- | --- | --- | --- | --- | --- | --- | --- | --- | --- | --- | --- | --- | --- | --- | --- | --- | --- | --- | --- | --- | --- | --- | --- | --- | --- | --- | --- | --- | --- | --- | --- | --- | --- | --- | --- | --- | --- | --- | --- | --- | --- | --- | --- | --- | --- | --- | --- | --- | --- | --- | --- | --- | --- | --- | --- | --- | --- | --- | --- | --- | --- | --- | --- | --- | --- | --- | --- | --- | --- | --- | --- | --- | --- | --- | --- | --- | --- | --- | --- | --- | --- | --- | --- | --- | --- | --- | --- | --- | --- | --- | --- | --- | --- | --- | --- | --- | --- | --- | --- | --- | --- | --- | --- | --- | --- | --- | --- | --- | --- | --- | --- | --- | --- | --- | --- | --- | --- | --- | --- | --- | --- | --- | --- | --- | --- | --- | --- | --- | --- | --- | --- | --- | --- | --- | --- | --- | --- | --- | --- | --- | --- | --- | --- | --- | --- | --- | --- | --- | --- | --- | --- | --- | --- | --- | --- | --- | --- | --- | --- | --- | --- | --- | --- | --- | --- | --- | --- | --- | --- | --- | --- | --- | --- | --- | --- | --- | --- | --- | --- | --- | --- | --- | --- | --- | --- | --- | --- | --- | --- | --- | --- | --- | --- | --- | --- | --- | --- | --- | --- | --- | --- | --- | --- | --- | --- | --- | --- | --- | --- | --- | --- | --- | --- | --- | --- | --- | --- | --- | --- | --- | --- | --- | --- | --- | --- | --- | --- | --- | --- | --- | --- | --- | --- | --- | --- | --- | --- | --- | --- | --- | --- | --- | --- | --- | --- | --- | --- | --- | --- | --- | --- | --- | --- | --- | --- | --- | --- | --- | --- | --- | --- | --- | --- | --- | --- | --- | --- | --- | --- | --- | --- | --- | --- | --- | --- | --- | --- | --- | --- | --- | --- | --- | --- | --- | --- | --- | --- | --- | --- | --- | --- | --- | --- | --- | --- | --- | --- | --- | --- | --- | --- | --- | --- | --- | --- | --- | --- | --- | --- | --- | --- | --- | --- | --- | --- | --- | --- | --- | --- | --- | --- | --- | --- | --- | --- | --- | --- | --- | --- | --- | --- | --- | --- | --- | --- | --- | --- | --- | --- | --- | --- | --- | --- | --- | --- | --- | --- | --- | --- | --- | --- | --- | --- | --- | --- | --- | --- | --- | --- | --- | --- | --- | --- | --- | --- | --- | --- | --- | --- | --- | --- | --- | --- | --- | --- | --- | --- | --- | --- | --- | --- | --- | --- | --- | --- | --- | --- | --- | --- | --- | --- | --- | --- | --- | --- | --- | --- | --- | --- | --- | --- | --- | --- | --- | --- | --- | --- | --- | --- | --- | --- | --- | --- | --- | --- | --- | --- | --- | --- | --- | --- | --- | --- | --- | --- | --- | --- | --- | --- | --- | --- | --- | --- | --- | --- | --- | --- | --- | --- | --- | --- | --- | --- | --- | --- | --- | --- | --- | --- | --- | --- | --- | --- | --- | --- | --- | --- | --- | --- | --- | --- | --- | --- | --- | --- | --- | --- | --- | --- | --- | --- | --- | --- | --- | --- | --- | --- | --- | --- | --- | --- | --- | --- | --- | --- | --- | --- | --- | --- | --- | --- | --- | --- | --- | --- | --- | --- | --- | --- | --- | --- | --- | --- | --- | --- | --- | --- | --- | --- | --- | --- | --- | --- | --- | --- | --- | --- | --- | --- | --- | --- | --- | --- | --- | --- | --- | --- | --- | --- | --- | --- | --- | --- | --- | --- | --- | --- | --- | --- | --- | --- | --- | --- | --- | --- | --- | --- | --- | --- | --- | --- | --- | --- | --- | --- | --- | --- | --- | --- | --- | --- | --- | --- | --- | --- | --- | --- | --- | --- | --- | --- | --- | --- | --- | --- | --- | --- | --- | --- | --- | --- | --- | --- | --- | --- | --- | --- | --- | --- | --- | --- | --- | --- | --- | --- | --- | --- | --- | --- | --- | --- | --- | --- | --- | --- | --- | --- | --- | --- | --- | --- | --- | --- | --- | --- | --- | --- | --- | --- | --- | --- | --- | --- | --- | --- | --- | --- | --- | --- | --- | --- | --- | --- | --- | --- | --- | --- | --- | --- | --- | --- | --- | --- | --- | --- | --- | --- | --- | --- | --- | --- | --- | --- | --- | --- | --- | --- | --- | --- | --- | --- | --- | --- | --- | --- | --- | --- | --- | --- | --- | --- | --- | --- | --- | --- | --- | --- | --- | --- | --- | --- | --- | --- | --- | --- | --- | --- | --- | --- | --- | --- | --- | --- | --- | --- | --- | --- | --- | --- | --- | --- | --- | --- | --- | --- | --- | --- | --- | --- | --- | --- | --- | --- | --- | --- | --- | --- | --- | --- | --- | --- | --- | --- | --- | --- | --- | --- | --- | --- | --- | --- | --- | --- | --- | --- | --- | --- | --- | --- | --- | --- | --- | --- | --- | --- | --- | --- | --- | --- | --- | --- | --- | --- | --- | --- | --- | --- | --- | --- | --- | --- | --- | --- | --- | --- | --- | --- | --- | --- | --- | --- | --- | --- | --- | --- | --- | --- | --- | --- | --- | --- | --- | --- | --- | --- | --- | --- | --- | --- | --- | --- | --- | --- | --- | --- | --- | --- | --- | --- | --- | --- | --- | --- | --- | --- | --- | --- | --- | --- | --- | --- | --- | --- | --- | --- | --- | --- | --- | --- | --- | --- | --- | --- | --- | --- | --- | --- | --- | --- | --- | --- | --- | --- | --- | --- | --- | --- | --- | --- | --- | --- | --- | --- | --- | --- | --- | --- | --- | --- | --- | --- | --- | --- | --- | --- | --- | --- | --- | --- | --- | --- | --- | --- | --- | --- | --- | --- | --- | --- | --- | --- | --- | --- | --- | --- | --- | --- | --- | --- | --- | --- | --- | --- | --- | --- | --- | --- | --- | --- | --- | --- | --- | --- | --- | --- | --- | --- | --- | --- | --- | --- | --- | --- | --- | --- | --- | --- | --- | --- | --- | --- | --- | --- | --- | --- | --- | --- | --- | --- | --- | --- | --- | --- | --- | --- | --- | --- | --- | --- | --- | --- | --- | --- | --- | --- | --- | --- | --- | --- | --- | --- | --- | --- | --- | --- | --- | --- | --- | --- | --- | --- | --- | --- | --- | --- | --- | --- | --- | --- | --- | --- | --- | --- | --- | --- | --- | --- | --- | --- | --- | --- | --- | --- | --- | --- | --- | --- | --- | --- | --- | --- | --- | --- | --- | --- | --- | --- | --- | --- | --- | --- | --- | --- | --- | --- | --- | --- | --- | --- | --- | --- | --- | --- | --- | --- | --- | --- | --- | --- | --- | --- | --- | --- | --- | --- | --- | --- | --- | --- | --- | --- | --- | --- | --- | --- | --- | --- | --- | --- | --- | --- | --- | --- | --- | --- | --- | --- | --- | --- | --- | --- | --- | --- | --- | --- | --- | --- | --- | --- | --- | --- | --- | --- | --- | --- | --- | --- | --- | --- | --- | --- | --- | --- | --- | --- | --- | --- | --- | --- | --- | --- | --- | --- | --- | --- | --- | --- | --- | --- | --- | --- | --- | --- | --- | --- | --- | --- | --- | --- | --- | --- | --- | --- | --- | --- | --- | --- | --- | --- | --- | --- | --- | --- | --- | --- | --- | --- | --- | --- | --- | --- | --- | --- | --- | --- | --- | --- | --- | --- | --- | --- | --- | --- | --- | --- | --- | --- | --- | --- | --- | --- | --- | --- | --- | --- | --- | --- | --- | --- | --- | --- | --- | --- | --- | --- | --- | --- | --- | --- | --- | --- | --- | --- | --- | --- | --- | --- | --- | --- | --- | --- | --- | --- | --- | --- | --- | --- | --- | --- | --- | --- | --- | --- | --- | --- | --- | --- | --- | --- | --- | --- | --- | --- | --- | --- | --- | --- | --- | --- | --- | --- | --- | --- | --- | --- | --- | --- | --- | --- | --- | --- | --- | --- | --- | --- | --- | --- | --- | --- | --- | --- | --- | --- | --- | --- | --- | --- | --- | --- | --- | --- | --- | --- | --- | --- | --- | --- | --- | --- | --- | --- | --- | --- | --- | --- | --- | --- | --- | --- | --- | --- | --- | --- | --- | --- | --- | --- | --- | --- | --- | --- | --- | --- | --- | --- | --- | --- | --- | --- | --- | --- | --- | --- | --- | --- | --- | --- | --- | --- | --- | --- | --- | --- | --- | --- | --- | --- | --- | --- | --- | --- | --- | --- | --- | --- | --- | --- | --- | --- | --- | --- | --- | --- | --- | --- | --- | --- | --- | --- | --- | --- | --- | --- | --- | --- | --- | --- | --- | --- | --- | --- | --- | --- | --- | --- | --- | --- | --- | --- | --- | --- | --- | --- | --- | --- | --- | --- | --- | --- | --- | --- | --- | --- | --- | --- | --- | --- | --- | --- | --- | --- | --- | --- | --- | --- | --- | --- | --- | --- | --- | --- | --- | --- | --- | --- | --- | --- | --- | --- | --- | --- | --- | --- | --- | --- | --- | --- | --- | --- | --- | --- | --- | --- | --- | --- | --- | --- | --- | --- | --- | --- | --- | --- | --- | --- | --- | --- | --- | --- | --- | --- | --- | --- | --- | --- | --- | --- | --- | --- | --- | --- | --- | --- | --- | --- | --- | --- | --- | --- | --- | --- | --- | --- | --- | --- | --- | --- | --- | --- | --- | --- | --- | --- | --- | --- | --- | --- | --- | --- | --- | --- | --- | --- | --- | --- | --- | --- | --- | --- | --- | --- | --- | --- | --- | --- | --- | --- | --- | --- | --- | --- | --- | --- | --- | --- | --- | --- | --- | --- | --- | --- | --- | --- | --- | --- | --- | --- | --- | --- | --- | --- | --- | --- | --- | --- | --- | --- | --- | --- | --- | --- | --- | --- | --- | --- | --- | --- | --- | --- | --- | --- | --- | --- | --- | --- | --- | --- | --- | --- | --- | --- | --- | --- | --- | --- | --- | --- | --- | --- | --- | --- | --- | --- | --- | --- | --- | --- | --- | --- | --- | --- | --- | --- | --- | --- | --- | --- | --- | --- | --- | --- | --- | --- | --- | --- | --- | --- | --- | --- | --- | --- | --- | --- | --- | --- | --- | --- | --- | --- | --- | --- | --- | --- | --- | --- | --- | --- | --- | --- | --- | --- | --- | --- | --- | --- | --- | --- | --- | --- | --- | --- | --- | --- | --- | --- | --- | --- | --- | --- | --- | --- | --- | --- | --- | --- | --- | --- | --- | --- | --- | --- | --- | --- | --- | --- | --- | --- | --- | --- | --- | --- | --- | --- | --- | --- | --- | --- | --- | --- | --- | --- | --- | --- | --- | --- | --- | --- | --- | --- | --- | --- | --- | --- | --- | --- | --- | --- | --- | --- | --- | --- | --- | --- | --- | --- | --- | --- | --- | --- | --- | --- | --- | --- | --- | --- | --- | --- | --- | --- | --- | --- | --- | --- | --- | --- | --- | --- | --- | --- | --- | --- | --- | --- | --- | --- | --- | --- | --- | --- | --- | --- | --- | --- | --- | --- | --- | --- | --- | --- | --- | --- | --- | --- | --- | --- | --- | --- | --- | --- | --- | --- | --- | --- | --- | --- | --- | --- | --- | --- | --- | --- | --- | --- | --- | --- | --- | --- | --- | --- | --- | --- | --- | --- | --- | --- | --- | --- | --- | --- | --- | --- | --- | --- | --- | --- | --- | --- | --- | --- | --- | --- | --- | --- | --- | --- | --- | --- | --- | --- | --- | --- | --- | --- | --- | --- | --- | --- | --- | --- | --- | --- | --- | --- | --- | --- | --- | --- | --- | --- | --- | --- | --- | --- | --- | --- | --- | --- | --- | --- | --- | --- | --- | --- | --- | --- | --- | --- | --- | --- | --- | --- | --- | --- | --- | --- | --- | --- | --- | --- | --- | --- | --- | --- | --- | --- | --- | --- | --- | --- | --- | --- | --- | --- | --- | --- | --- | --- | --- | --- | --- | --- | --- | --- | --- | --- | --- | --- | --- | --- | --- | --- | --- | --- | --- | --- | --- | --- | --- | --- | --- | --- | --- | --- | --- | --- | --- | --- | --- | --- | --- | --- | --- | --- | --- | --- | --- | --- | --- | --- | --- | --- | --- | --- | --- | --- | --- | --- | --- | --- | --- | --- | --- | --- | --- | --- | --- | --- | --- | --- | --- | --- | --- | --- | --- | --- | --- | --- | --- | --- | --- | --- | --- | --- | --- | --- | --- | --- | --- | --- | --- | --- | --- | --- | --- | --- | --- | --- | --- | --- | --- | --- | --- | --- | --- | --- | --- | --- | --- | --- | --- | --- | --- | --- | --- | --- | --- | --- | --- | --- | --- | --- | --- | --- | --- | --- | --- | --- | --- | --- | --- | --- | --- | --- | --- | --- | --- | --- | --- | --- | --- | --- | --- | --- | --- | --- | --- | --- | --- | --- | --- | --- | --- | --- | --- | --- | --- | --- | --- | --- | --- | --- | --- | --- | --- | --- | --- | --- | --- | --- | --- | --- | --- | --- | --- | --- | --- | --- | --- | --- | --- | --- | --- | --- | --- | --- | --- | --- | --- | --- | --- | --- | --- | --- | --- | --- | --- | --- | --- | --- | --- | --- | --- | --- | --- | --- | --- | --- | --- | --- | --- | --- | --- | --- | --- | --- | --- | --- | --- | --- | --- | --- | --- | --- | --- | --- | --- | --- | --- | --- | --- | --- | --- | --- | --- | --- | --- | --- | --- | --- | --- | --- | --- | --- | --- | --- | --- | --- | --- | --- | --- | --- | --- | --- | --- | --- | --- | --- | --- | --- | --- | --- | --- | --- | --- | --- | --- | --- | --- | --- | --- | --- | --- | --- | --- | --- | --- | --- | --- | --- | --- | --- | --- | --- | --- | --- | --- | --- | --- | --- | --- | --- | --- | --- | --- | --- | --- | --- | --- | --- | --- | --- | --- | --- | --- | --- | --- | --- | --- | --- | --- | --- | --- | --- | --- | --- | --- | --- | --- | --- | --- | --- | --- | --- | --- | --- | --- | --- | --- | --- | --- | --- | --- | --- | --- | --- | --- | --- | --- | --- | --- | --- | --- | --- | --- | --- | --- | --- | --- | --- | --- | --- | --- | --- | --- | --- | --- | --- | --- | --- | --- | --- | --- | --- | --- | --- | --- | --- | --- | --- | --- | --- | --- | --- | --- | --- | --- | --- | --- | --- | --- | --- | --- | --- | --- | --- | --- | --- | --- | --- | --- | --- | --- | --- | --- | --- | --- | --- | --- | --- | --- | --- | --- | --- | --- | --- | --- | --- | --- | --- | --- | --- | --- | --- | --- | --- | --- | --- | --- | --- | --- | --- | --- | --- | --- | --- | --- | --- | --- | --- | --- | --- | --- | --- | --- | --- | --- | --- | --- | --- | --- | --- | --- | --- | --- | --- | --- | --- | --- | --- | --- | --- | --- | --- | --- | --- | --- | --- | --- | --- | --- | --- | --- | --- | --- | --- | --- | --- | --- | --- | --- | --- | --- | --- | --- | --- | --- | --- | --- | --- | --- | --- | --- | --- | --- | --- | --- | --- | --- | --- | --- | --- | --- | --- | --- | --- | --- | --- | --- | --- | --- | --- | --- | --- | --- | --- | --- | --- | --- | --- | --- | --- | --- | --- | --- | --- | --- | --- | --- | --- | --- | --- | --- | --- | --- | --- | --- | --- | --- | --- | --- | --- | --- | --- | --- | --- | --- | --- | --- | --- | --- | --- | --- | --- | --- | --- | --- | --- | --- | --- | --- | --- | --- | --- | --- | --- | --- | --- | --- | --- | --- | --- | --- | --- | --- | --- | --- | --- | --- | --- | --- | --- | --- | --- | --- | --- | --- | --- | --- | --- | --- | --- | --- | --- | --- | --- | --- | --- | --- | --- | --- | --- | --- | --- | --- | --- | --- | --- | --- | --- | --- | --- | --- | --- | --- | --- | --- | --- | --- | --- | --- | --- | --- | --- | --- | --- | --- | --- | --- | --- | --- | --- | --- | --- | --- | --- | --- | --- | --- | --- | --- | --- | --- | --- | --- | --- | --- | --- | --- | --- | --- | --- | --- | --- | --- | --- | --- | --- | --- | --- | --- | --- | --- | --- | --- | --- | --- | --- | --- | --- | --- | --- | --- | --- | --- | --- | --- | --- | --- | --- | --- | --- | --- | --- | --- | --- | --- | --- | --- | --- | --- | --- | --- | --- | --- | --- | --- | --- | --- | --- | --- | --- | --- | --- | --- | --- | --- | --- | --- | --- | --- | --- | --- | --- | --- | --- | --- | --- | --- | --- | --- | --- | --- | --- | --- | --- | --- | --- | --- | --- | --- | --- | --- | --- | --- | --- | --- | --- | --- | --- | --- | --- | --- | --- | --- | --- | --- | --- | --- | --- | --- | --- | --- | --- | --- | --- | --- | --- | --- | --- | --- | --- | --- | --- | --- | --- | --- | --- | --- | --- | --- | --- | --- | --- | --- | --- | --- | --- | --- | --- | --- | --- | --- | --- | --- | --- | --- | --- | --- | --- | --- | --- | --- | --- | --- | --- | --- | --- | --- | --- | --- | --- | --- | --- | --- | --- | --- | --- | --- | --- | --- | --- | --- | --- | --- | --- | --- | --- | --- | --- | --- | --- | --- | --- | --- | --- | --- | --- | --- | --- | --- | --- | --- | --- | --- | --- | --- | --- | --- | --- | --- | --- | --- | --- | --- | --- | --- | --- | --- | --- | --- | --- | --- | --- | --- | --- | --- | --- | --- | --- | --- | --- | --- | --- | --- | --- | --- | --- | --- | --- | --- | --- | --- | --- | --- | --- | --- | --- | --- | --- | --- | --- | --- | --- | --- | --- | --- | --- | --- | --- | --- | --- | --- | --- | --- | --- | --- | --- | --- | --- | --- | --- | --- | --- | --- | --- | --- | --- | --- | --- | --- | --- | --- | --- | --- | --- | --- | --- | --- | --- | --- | --- | --- | --- | --- | --- | --- | --- | --- | --- | --- | --- | --- | --- | --- | --- | --- | --- | --- | --- | --- | --- | --- | --- | --- | --- | --- | --- | --- | --- | --- | --- | --- | --- | --- | --- | --- | --- | --- | --- | --- | --- | --- | --- | --- | --- | --- | --- | --- | --- | --- | --- | --- | --- | --- | --- | --- | --- | --- | --- | --- | --- | --- | --- | --- | --- | --- | --- | --- | --- | --- | --- | --- | --- | --- | --- | --- | --- | --- | --- | --- | --- | --- | --- | --- | --- | --- | --- | --- | --- | --- | --- | --- | --- | --- | --- | --- | --- | --- | --- | --- | --- | --- | --- | --- | --- | --- | --- | --- | --- | --- | --- | --- | --- | --- | --- | --- | --- | --- | --- | --- | --- | --- | --- | --- | --- | --- | --- | --- | --- | --- | --- | --- | --- | --- | --- | --- | --- | --- | --- | --- | --- | --- | --- | --- | --- | --- | --- | --- | --- | --- | --- | --- | --- | --- | --- | --- | --- | --- | --- | --- | --- | --- | --- | --- | --- | --- | --- | --- | --- | --- | --- | --- | --- | --- | --- | --- | --- | --- | --- | --- | --- | --- | --- | --- | --- | --- | --- | --- | --- | --- | --- | --- | --- | --- | --- | --- | --- | --- | --- | --- | --- | --- | --- | --- | --- | --- | --- | --- | --- | --- | --- | --- | --- | --- | --- | --- | --- | --- | --- | --- | --- | --- | --- | --- | --- | --- | --- | --- | --- | --- | --- | --- | --- | --- | --- | --- | --- | --- | --- | --- | --- | --- | --- | --- | --- | --- | --- | --- | --- | --- | --- | --- | --- | --- | --- | --- | --- | --- | --- | --- | --- | --- | --- | --- | --- | --- | --- | --- | --- | --- | --- | --- | --- | --- | --- | --- | --- | --- | --- | --- | --- | --- | --- | --- | --- | --- | --- | --- | --- | --- | --- | --- | --- | --- | --- | --- | --- | --- | --- | --- | --- | --- | --- | --- | --- | --- | --- | --- | --- | --- | --- | --- | --- | --- | --- | --- | --- | --- | --- | --- | --- | --- | --- | --- | --- | --- | --- | --- | --- | --- | --- | --- | --- | --- | --- | --- | --- | --- | --- | --- | --- | --- | --- | --- | --- | --- | --- | --- | --- | --- | --- | --- | --- | --- | --- | --- | --- | --- | --- | --- | --- | --- | --- | --- | --- | --- | --- | --- | --- | --- | --- | --- | --- | --- | --- | --- | --- | --- | --- | --- | --- | --- | --- | --- | --- | --- | --- | --- | --- | --- | --- | --- | --- | --- | --- | --- | --- | --- | --- | --- | --- | --- | --- | --- | --- | --- | --- | --- | --- | --- | --- | --- | --- | --- | --- | --- | --- | --- | --- | --- | --- | --- | --- | --- | --- | --- | --- | --- | --- | --- | --- | --- | --- | --- | --- | --- | --- | --- | --- | --- | --- | --- | --- | --- | --- | --- | --- | --- | --- | --- | --- | --- | --- | --- | --- | --- | --- | --- | --- | --- | --- | --- | --- | --- | --- | --- | --- | --- | --- | --- | --- | --- | --- | --- | --- | --- | --- | --- | --- | --- | --- | --- | --- | --- | --- | --- | --- | --- | --- | --- | --- | --- | --- | --- | --- | --- | --- | --- | --- | --- | --- | --- | --- | --- | --- | --- | --- | --- | --- | --- | --- | --- | --- | --- | --- | --- | --- | --- | --- | --- | --- | --- | --- | --- | --- | --- | --- | --- | --- | --- | --- | --- | --- | --- | --- | --- | --- | --- | --- | --- | --- | --- | --- | --- | --- | --- | --- | --- | --- | --- | --- | --- | --- | --- | --- | --- | --- | --- | --- | --- | --- | --- | --- | --- | --- | --- | --- | --- | --- | --- | --- | --- | --- | --- | --- | --- | --- | --- | --- | --- | --- | --- | --- | --- | --- | --- | --- | --- | --- | --- | --- | --- | --- | --- | --- | --- | --- | --- | --- | --- | --- | --- | --- | --- | --- | --- | --- | --- | --- | --- | --- | --- | --- | --- | --- | --- | --- | --- | --- | --- | --- | --- | --- | --- | --- | --- | --- | --- | --- | --- | --- | --- | --- | --- | --- | --- | --- | --- | --- | --- | --- | --- | --- | --- | --- | --- | --- | --- | --- | --- | --- | --- | --- | --- | --- | --- | --- | --- | --- | --- | --- | --- | --- | --- | --- | --- | --- | --- | --- | --- | --- | --- | --- | --- | --- | --- | --- | --- | --- | --- | --- | --- | --- | --- | --- | --- | --- | --- | --- | --- | --- | --- | --- | --- | --- | --- | --- | --- | --- | --- | --- | --- | --- | --- | --- | --- | --- | --- | --- | --- | --- | --- | --- | --- | --- | --- | --- | --- | --- | --- | --- | --- | --- | --- | --- | --- | --- | --- | --- | --- | --- | --- | --- | --- | --- | --- | --- | --- | --- | --- | --- | --- | --- | --- | --- | --- | --- | --- | --- | --- | --- | --- | --- | --- | --- | --- | --- | --- | --- | --- | --- | --- | --- | --- | --- | --- | --- | --- | --- | --- | --- | --- | --- | --- | --- | --- | --- | --- | --- | --- | --- | --- | --- | --- | --- | --- | --- | --- | --- | --- | --- | --- | --- | --- | --- | --- | --- | --- | --- | --- | --- | --- | --- | --- | --- | --- | --- | --- | --- | --- | --- | --- | --- | --- | --- | --- | --- | --- | --- | --- | --- | --- | --- | --- | --- | --- | --- | --- | --- | --- | --- | --- | --- | --- | --- | --- | --- | --- | --- | --- | --- | --- | --- | --- | --- | --- | --- | --- | --- | --- | --- | --- | --- | --- | --- | --- | --- | --- | --- | --- | --- | --- | --- | --- | --- | --- | --- | --- | --- | --- | --- | --- | --- | --- | --- | --- | --- | --- | --- | --- | --- | --- | --- | --- | --- | --- | --- | --- | --- | --- | --- | --- | --- | --- | --- | --- | --- | --- | --- | --- | --- | --- | --- | --- | --- | --- | --- | --- | --- | --- | --- | --- | --- | --- | --- | --- | --- | --- | --- | --- | --- | --- | --- | --- | --- | --- | --- | --- | --- | --- | --- | --- | --- | --- | --- | --- | --- | --- | --- | --- | --- | --- | --- | --- | --- | --- | --- | --- | --- | --- | --- | --- | --- | --- | --- | --- | --- | --- | --- | --- | --- | --- | --- | --- | --- | --- | --- | --- | --- | --- | --- | --- | --- | --- | --- | --- | --- | --- | --- | --- | --- | --- | --- | --- | --- | --- | --- | --- | --- | --- | --- | --- | --- | --- | --- | --- | --- | --- | --- | --- | --- | --- | --- | --- | --- | --- | --- | --- | --- | --- | --- | --- | --- | --- | --- | --- | --- | --- | --- | --- | --- | --- | --- | --- | --- | --- | --- | --- | --- | --- | --- | --- | --- | --- | --- | --- | --- | --- | --- | --- | --- | --- | --- | --- | --- | --- | --- | --- | --- | --- | --- | --- | --- | --- | --- | --- | --- | --- | --- | --- | --- | --- | --- | --- | --- | --- | --- | --- | --- | --- | --- | --- | --- | --- | --- | --- | --- | --- | --- | --- | --- | --- | --- | --- | --- | --- | --- | --- | --- | --- | --- | --- | --- | --- | --- | --- | --- | --- | --- | --- | --- | --- | --- | --- | --- | --- | --- | --- | --- | --- | --- | --- | --- | --- | --- | --- | --- | --- | --- | --- | --- | --- | --- | --- | --- | --- | --- | --- | --- | --- | --- | --- | --- | --- | --- | --- | --- | --- | --- |
| Swiss-Prot Accession Number | MIM Number | MeSH Unique Identifier | MeSH Heading | Score |||  |  |  |  |  |
| --- | --- | --- | --- | --- |
| O00116 | 600121 | D018902 | chondrodysplasia punctata, rhizomelic | 4.51799434341882 |
| O00142 | 609560 | D017240 | mitochondrial myopathies | -1.07637989171772 |
| O00165 | 610738 | D000380 | agranulocytosis | 2.63739705964813 |
| O00170 | - | D010911 | pituitary neoplasms | 2.98206552448654 |
| O00192 | - | D004062 | digeorge syndrome | exact |
| O00203 | 608233 203300 | D022861 | hermanski-pudlak syndrome | exact |
| O00217 | 256000 | D007888 | leigh disease | exact |
| O00253 | 601665 | D009765 | obesity | exact |
| O00255 | 131100 | D018761 | multiple endocrine neoplasia type 1 | exact |
| O00255 | 145000 | D006961 / D010282 | hyperparathyroidism / parathyroid neoplasms | -2.23962904623455 / 4.81795303089275 |
| O00287 | 209920 | D016511 | severe combined immunodeficiency | exact |
| O00294 | 600132 | D012174 | retinitis pigmentosa | 3.46942749164458 |
| O00300 | 239000 | D010001 | osteitis deformans | -0.473825919945487 |
| O00305 | 600669 | D004829 | epilepsy, generalized | 3.11259367809365 |
| O00305 | 606904 | D020190 | myoclonic epilepsy, juvenile | exact |
| O00425 | - | D002277 | carcinoma | -1.27287059546172 |
| O00462 | - | D010523 | peripheral nervous system diseases | -1.88791466926095 |
| O00462 | 248510 | D044905 | beta-mannosidosis | exact |
| O00507 | 415000 | D053713 | azoospermia | -2.27980843360295 |
| O00512 | - | D015456 | leukemia, mixed-cell | 1.4389797606945 |
| O00522 | 116860 | D020786 / D006392 | hemangioma, cavernous, central nervous system / hemangioma, cavernous | -2.34359769161866 / 5.90639510334001 |
| O00555 | 183086 | D020754 | spinocerebellar ataxias | exact |
| O00555 | 141500 | D020325 | migraine with aura | exact |
| O00555 | 108500 | D020754 | spinocerebellar ataxias | -1.7930892189747 |
| O00559 | - | D001943 | breast neoplasms | -0.892756447976702 |
| O00623 | 214100 | D015211 | zellweger syndrome | exact |
| O00628 | 215100 | D018902 | chondrodysplasia punctata, rhizomelic | 7.44882270413786 |
| O00628 | 266500 | D012035 | refsum disease | exact |
| O00754 | 248500 | D008363 | alpha-mannosidosis | exact |
| O00755 | 276820 | D004480 | ectromelia | -1.9439866168541 |
| O14521 | 168000 | D010235 | paraganglioma | exact |
| O14521 | 171300 | D010673 | pheochromocytoma | exact |
| O14521 | 114900 | D002276 | carcinoid tumor | 3.67609385900666 |
| O14543 | 605805 | D003876 | dermatitis, atopic | 4.74731197994585 |
| O14582 | 313400 | D010009 | osteochondrodysplasias | 4.04093238242959 |
| O14593 | 209920 | D016511 | severe combined immunodeficiency | exact |
| O14656 | 128100 | D004422 | dystonia musculorum deformans | 8.54510543871918 |
| O14662 | 603233 | D011547 | pseudohypoparathyroidism | -1.79395536550144 |
| O14763 | 275355 | D002294 | carcinoma, squamous cell | 1.29152651980015 |
| O14773 | 204500 | D009472 | neuronal ceroid-lipofuscinoses | exact |
| O14810 | - | D010300 | parkinson disease | exact |
| O14832 | 266500 | D012035 | refsum disease | exact |
| O14836 | 240500 | D017074 | common variable immunodeficiency | exact |
| O14896 | 119500 | D011625 | pterygium | -1.76254644477949 |
| O14901 | 610508 606391 | D003924 | diabetes mellitus, type 2 | exact |
| O14926 | 607921 | D012174 | retinitis pigmentosa | 3.31420630086164 |
| O14958 | 604772 | D017180 | tachycardia, ventricular | -2.14086407579149 |
| O15020 | 600224 | D020754 | spinocerebellar ataxias | exact |
| O15055 | 604348 | D020178 | sleep disorders, circadian rhythm | 7.040678663151 |
| O15078 | - | D016410 | lymphoma, t-cell, cutaneous | exact |
| O15118 | 257220 | D052536 / D052556 | niemann-pick disease, type a / niemann-pick disease, type c | 3.18512826808075 / exact |
| O15118 | 257220 | D052556 | niemann-pick disease, type c | exact |
| O15120 | 608594 | D052497 | lipodystrophy, congenital generalized | exact |
| O15146 | - | D020294 | myasthenic syndromes, congenital | 2.03576885177875 |
| O15164 | 188550 | D002291 | carcinoma, papillary | 3.4424308069019 |
| O15169 | 114550 | D006528 | carcinoma, hepatocellular | exact |
| O15178 | 182940 | D009436 | neural tube defects | exact |
| O15228 | 222765 | D018902 | chondrodysplasia punctata, rhizomelic | 4.63168723423471 |
| O15232 | 607078 | D010009 | osteochondrodysplasias | 5.31375614139795 |
| O15232 | 607850 | D010003 | osteoarthritis | 1.02290184480656 |
| O15259 | 256100 | D020734 | parkinsonian disorders | -2.42353278963974 |
| O15259 | 266900 | D015792 | retinal dysplasia | -0.776911957260617 |
| O15265 | 164500 | D020754 | spinocerebellar ataxias | exact |
| O15266 | 127300 | D008069 | lipomatosis, multiple symmetrical | -1.06331794766995 |
| O15269 | 162400 | D009477 / D002607 | hereditary sensory and autonomic neuropathies / charcot-marie-tooth disease | 4.73522600882901 / 5.01856058095501 |
| O15273 | 192600 | D024741 | cardiomyopathy, hypertrophic, familial | exact |
| O15273 | 601954 | D049288 | muscular dystrophies, limb-girdle | 2.85137522959833 |
| O15273 | 607487 | D002311 | cardiomyopathy, dilated | 1.73448321138946 |
| O15287 | 227650 | D005199 | fanconi anemia | exact |
| O15305 | 212065 | D018981 | carbohydrate-deficient glycoprotein syndrome | 3.99441938629333 |
| O15315 | - | D007889 | leiomyoma | 1.09293327265567 |
| O15344 | 300000 | D019082 / D053632 | smith-lemli-opitz syndrome / x-linked combined immunodeficiency diseases | -0.481329123329771 / -0.184608218412302 |
| O15350 | - | D009369 | neoplasms | -1.05114052514487 |
| O15360 | 227650 | D005199 | fanconi anemia | exact |
| O15409 | 602081 | D007805 / D001072 | language development disorders / apraxias | 1.27045456822331 / 5.57563673614132 |
| O15499 | - | D004062 | digeorge syndrome | 2.25845877406531 |
| O15527 | - | D009369 | neoplasms | -1.64725631865348 |
| O15527 | 144700 | D002292 | carcinoma, renal cell | exact |
| O15528 | 264700 | D053098 | hypophosphatemic rickets, x-linked dominant | -0.493810118971547 |
| O15537 | 312700 | D041441 | retinoschisis | 6.70339721571125 |
| O15553 | 249100 | D010505 | familial mediterranean fever | exact |
| O15553 | 134610 | D010505 | familial mediterranean fever | 3.27783179337005 |
| O43181 | 252010 | D028361 | mitochondrial diseases | 3.48654516268259 |
| O43186 | 204000 602225 | D001766 | blindness | -2.41211670188054 |
| O43186 | 120970 | D012174 | retinitis pigmentosa | exact |
| O43186 | 268000 | D012174 | retinitis pigmentosa | exact |
| O43272 | 600850 181500 | D012559 | schizophrenia | exact |
| O43295 | - | D008607 | mental retardation | -1.90159316931268 |
| O43307 | 300607 149400 | D016750 | stiff-person syndrome | 6.4222726824933 |
| O43323 | 607080 | D006061 | gonadal dysgenesis, 46,xy | -1.57620074662308 |
| O43395 | 601414 | D012174 | retinitis pigmentosa | 3.16816593006434 |
| O43405 | 156000 | D008575 | meniere's disease | exact |
| O43464 | 610297 168600 | D010300 | parkinson disease | exact |
| O43490 | 604365 | D012162 | retinal degeneration | -2.18336179099654 |
| O43511 | 274600 | D006042 | goiter | 1.59293327265567 |
| O43520 | 211600 | D002780 | cholestasis, intrahepatic | 4.41990370285314 |
| O43520 | 243300 | D002780 | cholestasis, intrahepatic | 1.13079506546235 |
| O43520 | 147480 | D002780 | cholestasis, intrahepatic | 4.78278957612661 |
| O43524 | - | D015447 | leukemia, lymphocytic, acute | -1.23455881925638 |
| O43525 | 121201 | D020936 | epilepsy, benign neonatal | 6.34574398571579 |
| O43526 | 121200 | D020936 | epilepsy, benign neonatal | 6.40914984481069 |
| O43526 | 606437 | D020936 | epilepsy, benign neonatal | 5.42523695968196 |
| O43542 | - | D008545 | melanoma | 3.88948703942647 |
| O43556 | 159900 | D009207 / D004421 | myoclonus / dystonia | 0.541708004093818 / -0.113902958761406 |
| O43602 | 300067 | D016114 | ichthyosis, x-linked | -2.41917853991304 |
| O43612 | 161400 | D009290 / D002385 | narcolepsy / cataplexy | exact / exact |
| O43623 | - | D009436 | neural tube defects | exact |
| O43623 | 608890 193500 | D014849 | waardenburg's syndrome | 0.678021719672773 |
| O43683 | - | D009369 | neoplasms | -1.64725631865348 |
| O43707 | - | D009362 | neoplasm metastasis | 2.72697824428557 |
| O43707 | 603278 | D005923 | glomerulosclerosis, focal segmental | exact |
| O43709 | 194050 | D018980 | williams syndrome | exact |
| O43819 | 604377 220110 | D030401 | cytochrome-c oxidase deficiency | exact |
| O43826 | 232220 | D005953 / D006008 | glycogen storage disease type i / glycogen storage disease | 2.38345637621233 / 3.81006281428742 |
| O43826 | 232240 | D005953 / D006008 | glycogen storage disease type i / glycogen storage disease | 2.15595567146234 / 3.637093388128 |
| O43826 | 232240 | D005953 / D006008 | glycogen storage disease type i / glycogen storage disease | 2.18345637621233 / 3.637093388128 |
| O43914 | 221770 | D000544 | alzheimer disease | 1.14052344313828 |
| O43918 | 240300 | D016884 | polyendocrinopathies, autoimmune | exact |
| O43930 | - | D012735 | sexual dysfunction, physiological | -2.34162926833566 |
| O43933 | 202370 | D018901 | peroxisomal disorders | exact |
| O43933 | 266510 | D052919 | refsum disease, infantile | exact |
| O60220 | 311150 | D012513 | sarcoma, experimental | -0.351338416808661 |
| O60229 | 608901 | D003327 / D003324 | coronary disease / coronary arteriosclerosis | 2.11939757636375 / 1.78271641191777 |
| O60260 | 168600 | D010300 | parkinson disease | exact |
| O60260 | 600116 | D020734 | parkinsonian disorders | exact |
| O60260 | - | D010051 | ovarian neoplasms | -2.1554623650737 |
| O60312 | 105830 | D017204 | angelman syndrome | exact |
| O60313 | 165500 | D029241 | optic atrophy, autosomal dominant | exact |
| O60313 | 125250 | D029241 | optic atrophy, autosomal dominant | 0.757270877358101 |
| O60333 | 118210 | D002607 / D015417 | charcot-marie-tooth disease / hereditary motor and sensory neuropathies | 3.03516657125508 / 4.22953183327741 |
| O60343 | - | D003876 | dermatitis, atopic | exact |
| O60346 | - | D003110 | colonic neoplasms | exact |
| O60488 | 300387 | D038901 | mental retardation, x-linked | 3.95216648067739 |
| O60494 | 261100 | D000749 | anemia, megaloblastic | 5.17616006721624 |
| O60500 | 256300 | D009404 / D009401 | nephrotic syndrome / nephrosis | -1.86508303253834 / 2.92899049756379 |
| O60566 | - | D009369 | neoplasms | -1.64725631865348 |
| O60602 | 601744 | D008180 | lupus erythematosus, systemic | 3.4773681083266 |
| O60610 | 124900 | D034381 | hearing loss | -2.39374129018224 |
| O60663 | 161200 | D009261 | nail-patella syndrome | exact |
| O60674 | 600880 | D006502 | hepatic vein thrombosis | exact |
| O60674 | 263300 | D011087 | polycythemia vera | exact |
| O60674 | 187950 | D013920 | thrombocythemia, hemorrhagic | exact |
| O60674 | 254450 | D009191 | myelofibrosis | 3.70808208236657 |
| O60674 | 601626 | D007950 | leukemia, myelocytic, acute | exact |
| O60683 | 214100 | D015211 | zellweger syndrome | exact |
| O60683 | 202370 | D018901 | peroxisomal disorders | exact |
| O60687 | 300643 | D038901 | mental retardation, x-linked | -1.71207227168448 |
| O60706 | 608569 | D002311 | cardiomyopathy, dilated | 1.73448321138946 |
| O60733 | 256600 | D019150 | neuroaxonal dystrophies | exact |
| O60779 | 249270 | D000749 | anemia, megaloblastic | -1.85151985899435 |
| O60806 | 201400 | D047748 | pituitary acth hypersecretion | -1.96033094650538 |
| O60828 | 309500 | D038901 | mental retardation, x-linked | 4.15216648067739 |
| O60832 | 305000 | D019871 | dyskeratosis congenita | exact |
| O60840 | 300476 | D012174 | retinitis pigmentosa | 0.955097976986772 |
| O60858 | - | D015451 | leukemia, b-cell, chronic | exact |
| O60861 | - | D007950 | leukemia, myelocytic, acute | 4.54951692806327 |
| O60879 | 300511 | D016649 | ovarian failure, premature | 5.99462799003953 |
| O60880 | 308240 | D008232 | lymphoproliferative disorders | 5.71497396422955 |
| O60882 | 204700 | D000567 | amelogenesis imperfecta | -1.57692150179535 |
| O60890 | 300486 | D038901 | mental retardation, x-linked | 4.37526192416138 |
| O60931 | 219800 219900 219750 | D003554 | cystinosis | exact |
| O60934 | 251260 | D049932 | nijmegen breakage syndrome | exact |
| O60934 | 114480 | D001943 | breast neoplasms | exact |
| O60934 | 609135 | D000741 | anemia, aplastic | exact |
| O60934 | - | D015454 | leukemia, lymphocytic, acute, l1 | exact |
| O60938 | - | D007640 | keratoconus | 0.868482797083103 |
| O75027 | 301310 | D000756 | anemia, sideroblastic | 0.887376341726064 |
| O75030 | 193510 | D014849 | waardenburg's syndrome | -0.373691172937395 |
| O75030 | 103470 | D016117 | albinism, ocular | 0.544838309588166 |
| O75030 | 103500 | D013991 | tietze's syndrome | exact |
| O75072 | 253800 | D009136 | muscular dystrophies | 0.133575041251881 |
| O75072 | 236670 | D015792 | retinal dysplasia | -2.2899137831277 |
| O75078 | - | D010051 | ovarian neoplasms | 2.98268049532772 |
| O75112 | 115200 | D002311 | cardiomyopathy, dilated | exact |
| O75161 | 606996 266900 | D015792 | retinal dysplasia | -0.776911957260617 |
| O75197 | 133780 601813 | D020821 / D012178 | dystonic disorders / retinopathy of prematurity | -2.12382014299548 / exact |
| O75197 | 259770 | D010013 | osteogenesis imperfecta | 1.42580719352241 |
| O75197 | 144750 | D015576 / D010026 | hyperostosis / osteosclerosis | -1.82767591430628 / -0.420982228325784 |
| O75197 | 144750 | D010026 | osteosclerosis | -0.420982228325784 |
| O75197 | 607634 | D010022 / D011546 | osteopetrosis / pseudohypoaldosteronism | -0.748467951318351 / -1.00557706593684 |
| O75251 | 256000 | D007888 | leigh disease | exact |
| O75251 | 252010 | D028361 | mitochondrial diseases | 3.48654516268259 |
| O75306 | 252010 | D028361 | mitochondrial diseases | 3.48654516268259 |
| O75342 | 242100 | D016113 / D017490 | ichthyosiform erythroderma, congenital / ichthyosis, lamellar | 2.34951558019456 / 8.18206058101934 |
| O75344 | 194050 | D018980 | williams syndrome | exact |
| O75360 | 262600 | D007018 | hypopituitarism | exact |
| O75364 | 602669 | D002386 | cataract | 1.18779411402348 |
| O75365 | - | D015179 | colorectal neoplasms | exact |
| O75369 | 108720 | D005870 | giant cell tumors | -0.51832586264023 |
| O75369 | 150250 | D009394 | nephritis, hereditary | -2.11815233473355 |
| O75444 | - | D009101 | multiple myeloma | 4.93847918382014 |
| O75445 | 276901 | D052245 | usher syndromes | 0.648469843015194 |
| O75445 | 268000 | D012174 | retinitis pigmentosa | exact |
| O75503 | 256731 | D009472 | neuronal ceroid-lipofuscinoses | 6.38523518436492 |
| O75558 | 603552 | D051359 | lymphohistiocytosis, hemophagocytic | 6.21994618133606 |
| O75581 | 610947 | D003324 | coronary arteriosclerosis | 0.499070307149148 |
| O75602 | - | D007246 | infertility | -0.590286122820909 |
| O75636 | - | D008180 | lupus erythematosus, systemic | -1.41122092089593 |
| O75665 | 311200 | D009958 | orofaciodigital syndromes | 4.20792464057471 |
| O75665 | 300209 311200 | D009958 | orofaciodigital syndromes | 4.20792464057471 |
| O75695 | 312600 | D012174 | retinitis pigmentosa | 1.86652898298563 |
| O75718 | 610682 | D010013 | osteogenesis imperfecta | 1.63661751885106 |
| O75718 | 610854 | D010013 | osteogenesis imperfecta | 1.27697426541615 |
| O75787 | 300423 | D038901 | mental retardation, x-linked | 5.47852315217222 |
| O75792 | 610333 225750 | D004660 | encephalitis | -2.14884027432034 |
| O75800 | - | D002289 | carcinoma, non-small-cell lung | 2.24284003207922 |
| O75880 | 220110 | D030401 | cytochrome-c oxidase deficiency | exact |
| O75914 | 300558 | D038901 | mental retardation, x-linked | 4.71363746508891 |
| O75923 | 253601 | D049288 | muscular dystrophies, limb-girdle | 4.204964149155 |
| O75923 | 254130 | D049310 | distal myopathies | -0.89089356118266 |
| O75923 | 606768 | D000868 | anterior compartment syndrome | -2.353482499053 |
| O76024 | 222300 | D014929 | wolfram syndrome | exact |
| O76027 | - | D010392 | pemphigus | exact |
| O76039 | 308350 | D013036 | spasms, infantile | 0.884962179277794 |
| O76039 | 312750 | D015518 | rett syndrome | exact |
| O76041 | - | D002311 | cardiomyopathy, dilated | -1.15982366883245 |
| O76082 | 266600 | D003424 | crohn disease | exact |
| O76083 | - | D001714 | bipolar disorder | 2.99630283596713 |
| O76090 | 153700 | D003317 | corneal dystrophies, hereditary | -0.477223953805215 |
| O94761 | 268400 | D011038 | rothmund-thomson syndrome | exact |
| O94761 | 218600 | D003398 | craniosynostoses | -2.30759177484652 |
| O94833 | - | D010391 | pemphigoid, bullous | exact |
| O94915 | - | D015447 | leukemia, lymphocytic, acute | 1.37461500344422 |
| O94972 | 253250 | D050336 | mulibrey nanism | exact |
| O95140 | 609260 | D002607 / D015417 | charcot-marie-tooth disease / hereditary motor and sensory neuropathies | 3.03516657125508 / 4.22953183327741 |
| O95140 | 601152 | D002607 / D015417 | charcot-marie-tooth disease / hereditary motor and sensory neuropathies | 3.88307704479681 / 5.13424422448881 |
| O95163 | 223900 | D004402 | dysautonomia, familial | exact |
| O95255 | 177850 | D011561 | pseudoxanthoma elasticum | 5.71199675080214 |
| O95255 | 264800 | D011561 | pseudoxanthoma elasticum | exact |
| O95278 | 254780 | D020191 / D020192 | myoclonic epilepsies, progressive / lafora disease | 2.76694641728193 / exact |
| O95292 | 608627 | D000690 | amyotrophic lateral sclerosis | 6.00445849813384 |
| O95342 | 601847 | D002780 | cholestasis, intrahepatic | -0.169107357163502 |
| O95342 | 605479 | D002780 | cholestasis, intrahepatic | -0.812966204801999 |
| O95343 | 157170 | D016142 | holoprosencephaly | 2.16622298340068 |
| O95409 | 609637 | D016142 | holoprosencephaly | 1.2983221528844 |
| O95450 | 225410 | D004535 | ehlers-danlos syndrome | 1.52898291585314 |
| O95452 | 129500 | D004476 | ectodermal dysplasia | exact |
| O95461 | 608840 | D009136 | muscular dystrophies | -2.24931843955821 |
| O95467 | 219080 | D003480 | cushing syndrome | -1.87245777468612 |
| O95467 | 603233 | D011547 | pseudohypoparathyroidism | -1.79395536550144 |
| O95477 | 205400 | D013631 | tangier disease | exact |
| O95477 | 604091 | D013631 / D052456 | tangier disease / hypoalphalipoproteinemias | 5.34782704177438 / exact |
| O95631 | - | D009447 | neuroblastoma | 2.54661873943849 |
| O95633 | - | D015451 | leukemia, b-cell, chronic | 5.75667838456512 |
| O95677 | 605362 | D002311 | cardiomyopathy, dilated | 1.92947071162984 |
| O95684 | - | D009196 | myeloproliferative disorders | 0.609720507513883 |
| O95967 | 219100 | D003483 | cutis laxa | 2.81085466105711 |
| O95970 | 600512 | D004833 | epilepsy, temporal lobe | 3.38936175394153 |
| O95972 | 300510 | D016649 | ovarian failure, premature | 5.5966365179248 |
| O95995 | - | D007248 | infertility, male | -1.51774367961544 |
| O95999 | - | D009369 | neoplasms | exact |
| O96017 | 609265 | D016864 | li-fraumeni syndrome | 6.69999176981514 |
| O96017 | 176807 | D011471 | prostatic neoplasms | exact |
| O96017 | 259500 | D012516 | osteosarcoma | exact |
| O96028 | - | D009101 | multiple myeloma | 4.98889067579694 |
| P00156 | 535000 | D029242 | optic atrophy, hereditary, leber | exact |
| P00338 | - | D009212 | myoglobinuria | -0.368482797083103 |
| P00387 | 250800 | D008708 | methemoglobinemia | 3.13922472911024 |
| P00395 | 535000 | D029242 | optic atrophy, hereditary, leber | exact |
| P00395 | - | D000744 | anemia, hemolytic, autoimmune | 0.204860680617326 |
| P00395 | 220110 | D030401 | cytochrome-c oxidase deficiency | exact |
| P00395 | 550500 | D009212 | myoglobinuria | 1.69386507664155 |
| P00403 | - | D009369 | neoplasms | -1.64725631865348 |
| P00414 | 535000 | D029242 | optic atrophy, hereditary, leber | exact |
| P00414 | - | D017241 | melas syndrome | 5.00970127599674 |
| P00414 | 220110 | D030401 | cytochrome-c oxidase deficiency | exact |
| P00414 | 550500 | D009212 | myoglobinuria | 1.69386507664155 |
| P00439 | 261600 | D010661 | phenylketonurias | exact |
| P00439 | 261600 | D010661 | phenylketonurias | exact |
| P00439 | 261600 | D010661 | phenylketonurias | exact |
| P00441 | 105400 | D000690 | amyotrophic lateral sclerosis | 6.81087538647617 |
| P00451 | 306700 | D006467 | hemophilia a | exact |
| P00480 | 311250 | D022124 / D020163 | hyperammonemia / ornithine carbamoyltransferase deficiency disease | -0.469299727667928 / 10.7359524593755 |
| P00488 | 134570 | D005177 | factor xiii deficiency | 2.85314956970842 |
| P00491 | 164050 | D006012 | glycogen storage disease type v | -1.42011126311571 |
| P00492 | 300322 | D007926 | lesch-nyhan syndrome | exact |
| P00492 | 300323 | D006073 | gout | exact |
| P00519 | 608232 | D015464 | leukemia, myeloid, chronic | exact |
| P00533 | 211980 | D008175 | lung neoplasms | exact |
| P00558 | - | D000743 | anemia, hemolytic | 4.06604258324254 |
| P00568 | 103000 | D000743 | anemia, hemolytic | 4.19195705491373 |
| P00734 | 176930 | D007020 | hypoprothrombinemias | exact |
| P00734 | 601367 | D020521 | cerebrovascular accident | exact |
| P00740 | 306900 | D002836 | hemophilia b | exact |
| P00747 | 188050 | D019851 | thrombophilia | exact |
| P00747 | 217090 | D003231 | conjunctivitis | -1.8639602272816 |
| P00748 | 234000 | D005171 / D005175 | factor x deficiency / factor xii deficiency | -1.0539965336839 / exact |
| P00748 | 610618 | D000799 | angioneurotic edema | 1.05718989067856 |
| P00846 | 551500 | D012174 | retinitis pigmentosa | 1.08718562691483 |
| P00846 | 535000 | D029242 | optic atrophy, hereditary, leber | exact |
| P00846 | 256000 | D007888 | leigh disease | exact |
| P00846 | 500003 | D020955 | striatonigral degeneration | 0.846213398154422 |
| P00918 | 259730 | D000141 | acidosis, renal tubular | 4.84833304054306 |
| P00966 | 215700 | D020159 | citrullinemia | exact |
| P00995 | 167800 | D010195 / D050500 | pancreatitis / pancreatitis, chronic | 2.16212657391681 / exact |
| P01008 | 107300 188050 | D020152 | antithrombin iii deficiency | exact |
| P01009 | - | D029424 | pulmonary disease, chronic obstructive | exact |
| P01009 | - | D004198 | disease susceptibility | 1.21647970363805 |
| P01011 | - | D029424 | pulmonary disease, chronic obstructive | exact |
| P01019 | 145500 | D006973 | hypertension | -0.627827437734098 |
| P01031 | 120900 | D008103 | liver cirrhosis | exact |
| P01034 | 105150 | D028226 / D028243 | amyloidosis, familial / cerebral amyloid angiopathy, familial | 0.338952295734033 / exact |
| P01106 | - | D015451 | leukemia, b-cell, chronic | 6.06538328112731 |
| P01111 | 607785 | D015479 | leukemia, myelomonocytic, acute | 0.960874583246403 |
| P01112 | 109800 | D001749 | urinary bladder neoplasms | exact |
| P01112 | - | D002294 | carcinoma, squamous cell | 4.52772362236314 |
| P01116 | 601626 | D007950 | leukemia, myelocytic, acute | exact |
| P01116 | 607785 | D015479 | leukemia, myelomonocytic, acute | 0.960874583246403 |
| P01116 | 609942 163950 | D009634 | noonan syndrome | exact |
| P01116 | - | D009369 | neoplasms | -1.02511715126007 |
| P01124 | - | D002051 | burkitt lymphoma | 5.18756600966134 |
| P01127 | 607907 | D018223 | dermatofibrosarcoma | -0.368482797083102 |
| P01130 | 143890 | D006938 | hyperlipoproteinemia type ii | exact |
| P01137 | 131300 | D003966 | camurati-engelmann syndrome | exact |
| P01138 | 608654 | D009477 / D000699 | hereditary sensory and autonomic neuropathies / pain insensitivity, congenital | exact / exact |
| P01185 | 125700 | D020790 | diabetes insipidus, neurogenic | 7.65344871145922 |
| P01185 | 125700 | D020790 | diabetes insipidus, neurogenic | 7.65344871145922 |
| P01189 | 601665 | D009765 | obesity | exact |
| P01225 | 229070 | D006964 | hyperpituitarism | -1.13217340503033 |
| P01229 | 152780 | D007006 | hypogonadism | exact |
| P01241 | 262400 | D004393 | dwarfism, pituitary | 4.58606284218636 |
| P01241 | 262650 | D004393 | dwarfism, pituitary | 3.86621422363226 |
| P01241 | 173100 | D004393 | dwarfism, pituitary | -0.798774660137663 |
| P01266 | 188450 | D006042 | goiter | 2.11537050131346 |
| P01266 | 608175 | D013959 | thyroid diseases | -1.88993475826045 |
| P01270 | 146200 | D007011 / D006996 | hypoparathyroidism / hypocalcemia | -2.09047605391081 / 2.36904612981025 |
| P01308 | 176730 | D007333 | insulin resistance | -0.187278568192582 |
| P01343 | 608747 | D008607 | mental retardation | -0.516026861676404 |
| P01579 | 609135 | D000741 | anemia, aplastic | exact |
| P01857 | 254500 | D009101 | multiple myeloma | exact |
| P02042 | - | D017086 | beta-thalassemia | 3.73325008111945 |
| P02452 | 114000 | D006958 | hyperostosis, cortical, congenital | exact |
| P02452 | 130000 | D004535 | ehlers-danlos syndrome | 2.95173134906298 |
| P02452 | 130060 | D004535 | ehlers-danlos syndrome | 1.12898291585315 |
| P02452 | 166200 | D010013 | osteogenesis imperfecta | 3.54471768916804 |
| P02452 | 166210 | D010013 | osteogenesis imperfecta | 4.39110395953738 |
| P02452 | 259420 | D010013 | osteogenesis imperfecta | 1.96499238705526 |
| P02452 | 166220 | D010013 | osteogenesis imperfecta | 1.70354146465198 |
| P02452 | 607907 | D018223 | dermatofibrosarcoma | -0.368482797083102 |
| P02458 | 183900 | D010009 | osteochondrodysplasias | 3.75941722197425 |
| P02458 | 608805 | D005271 | femur head necrosis | 2.48248241064345 |
| P02458 | 151210 | D013796 | thanatophoric dysplasia | -1.07075454447799 |
| P02458 | 132450 | D010009 | osteochondrodysplasias | -0.896845567484706 |
| P02458 | 609508 | D012163 | retinal detachment | -2.00817488570778 |
| P02461 | 130020 | D004535 | ehlers-danlos syndrome | 2.72898291585315 |
| P02461 | 130050 | D004535 | ehlers-danlos syndrome | 2.72443890055313 |
| P02462 | 607595 | D020300 / D010291 | intracranial hemorrhages / paresis | -2.20693718305178 / 2.31135871135829 |
| P02462 | 175780 | D006429 | hemiplegia | 2.74722130184603 |
| P02489 | 123580 604219 | D002386 | cataract | -1.79112355822367 |
| P02489 | - | D002386 | cataract | -1.05521199484682 |
| P02511 | 203450 | D038261 | alexander disease | exact |
| P02511 | - | D002386 | cataract | -1.05521199484682 |
| P02533 | 131760 131800 131900 601001 | D016110 / D016108 | epidermolysis bullosa simplex / epidermolysis bullosa dystrophica | exact / exact |
| P02533 | 131760 | D016110 | epidermolysis bullosa simplex | 10.3902777727887 |
| P02533 | 131800 | D016110 / D016108 | epidermolysis bullosa simplex / epidermolysis bullosa dystrophica | 0.396432498403869 / exact |
| P02533 | 131900 | D016110 | epidermolysis bullosa simplex | 2.36143982935086 |
| P02538 | 167200 | D053549 | pachyonychia congenita | exact |
| P02545 | 181350 | D020389 | muscular dystrophy, emery-dreifuss | exact |
| P02545 | 604929 | D020389 | muscular dystrophy, emery-dreifuss | exact |
| P02545 | 115200 | D002311 | cardiomyopathy, dilated | exact |
| P02545 | 607920 | D002311 | cardiomyopathy, dilated | -0.833694753334302 |
| P02545 | 151660 | D052496 | lipodystrophy, familial partial | exact |
| P02545 | 159001 | D049288 | muscular dystrophies, limb-girdle | 3.42794716603788 |
| P02545 | 605588 | D002607 | charcot-marie-tooth disease | 2.98151188877386 |
| P02545 | 176670 | D011371 | progeria | exact |
| P02545 | 607554 | D001281 | atrial fibrillation | 5.4932847670704 |
| P02545 | 277700 | D014898 | werner syndrome | exact |
| P02549 | 130600 | D004612 | elliptocytosis, hereditary | -2.13187906901289 |
| P02647 | 604091 | D013631 / D052456 | tangier disease / hypoalphalipoproteinemias | 5.34782704177438 / exact |
| P02647 | 205400 | D013631 | tangier disease | exact |
| P02647 | 105200 | D028226 | amyloidosis, familial | 3.57457139122741 |
| P02649 | 107741 | D006952 | hyperlipoproteinemia type iii | exact |
| P02649 | 104310 | D000544 | alzheimer disease | 5.96109798634939 |
| P02655 | 207750 | D008072 | hyperlipoproteinemia type i | exact |
| P02671 | 202400 | D000347 | afibrinogenemia | 3.56904445716015 |
| P02671 | 105200 | D028226 | amyloidosis, familial | 3.72778565836074 |
| P02675 | - | D019851 | thrombophilia | exact |
| P02675 | 202400 | D000347 | afibrinogenemia | 3.56904445716015 |
| P02679 | - | D019851 | thrombophilia | exact |
| P02679 | 202400 | D000347 | afibrinogenemia | 3.56904445716015 |
| P02708 | 254200 | D009157 | myasthenia gravis | exact |
| P02708 | 601462 254200 | D020294 | myasthenic syndromes, congenital | exact |
| P02708 | 608930 | D020294 | myasthenic syndromes, congenital | 0.00627000693277147 |
| P02730 | 109270 130600 | D004612 | elliptocytosis, hereditary | -2.13187906901289 |
| P02730 | 109270 | D013103 | spherocytosis, hereditary | exact |
| P02730 | 179800 | D000141 | acidosis, renal tubular | exact |
| P02735 | - | D008258 | waldenstrom macroglobulinemia | exact |
| P02735 | - | D013167 | spondylitis, ankylosing | exact |
| P02735 | - | D001171 | arthritis, juvenile rheumatoid | exact |
| P02766 | 176300 | D000686 | amyloidosis | exact |
| P02766 | 105210 | D000686 | amyloidosis | -0.532065168709857 |
| P02766 | 176300 | D006981 / D000686 | hyperthyroxinemia / amyloidosis | exact / 1.29509010499609 |
| P02768 | 103600 | D050010 | hyperthyroxinemia, familial dysalbuminemic | exact |
| P02792 | 606159 | D001480 | basal ganglia diseases | 2.19963213747755 |
| P03886 | 535000 | D029242 | optic atrophy, hereditary, leber | exact |
| P03886 | 540000 | D017241 | melas syndrome | exact |
| P03886 | 502500 | D000544 | alzheimer disease | exact |
| P03886 | - | D003924 | diabetes mellitus, type 2 | exact |
| P03891 | 535000 | D029242 | optic atrophy, hereditary, leber | exact |
| P03891 | 502500 | D000544 | alzheimer disease | exact |
| P03901 | 535000 | D029242 | optic atrophy, hereditary, leber | exact |
| P03905 | 535000 | D029242 | optic atrophy, hereditary, leber | exact |
| P03905 | 500001 | D029242 | optic atrophy, hereditary, leber | 5.27375469159379 |
| P03905 | 540000 | D017241 | melas syndrome | exact |
| P03915 | 535000 | D029242 | optic atrophy, hereditary, leber | exact |
| P03923 | 535000 | D029242 | optic atrophy, hereditary, leber | exact |
| P03923 | 500001 | D029242 | optic atrophy, hereditary, leber | 5.27375469159379 |
| P03923 | 540000 | D017241 | melas syndrome | exact |
| P03951 | 264900 | D005173 | factor xi deficiency | exact |
| P03971 | 261550 | D011545 | pseudohermaphroditism | 3.83771255551425 |
| P03989 | 106300 | D013167 | spondylitis, ankylosing | exact |
| P03999 | 190900 | D003117 | color vision defects | exact |
| P04000 | 303900 | D003117 | color vision defects | exact |
| P04001 | 303800 | D003117 | color vision defects | exact |
| P04040 | 115500 | D020642 | acatalasia | exact |
| P04062 | 230800 | D005776 | gaucher disease | exact |
| P04062 | 230800 | D005776 | gaucher disease | exact |
| P04062 | 230900 | D005776 | gaucher disease | exact |
| P04062 | 231000 | D005776 | gaucher disease | exact |
| P04062 | 231005 | D005776 | gaucher disease | 0.547316208213036 |
| P04062 | 608013 | D005776 | gaucher disease | 0.297316208213036 |
| P04062 | 236750 | D015160 | hydrops fetalis | exact |
| P04062 | 168600 | D010300 | parkinson disease | exact |
| P04066 | 230000 | D005645 | fucosidosis | exact |
| P04070 | 176860 188050 | D020151 / D019851 | protein c deficiency / thrombophilia | exact / exact |
| P04075 | 103850 | D000743 | anemia, hemolytic | exact |
| P04080 | 254800 | D020191 | myoclonic epilepsies, progressive | exact |
| P04114 | 107730 200100 | D006995 / D000012 | hypobetalipoproteinemias / abetalipoproteinemia | 3.20808208236656 / exact |
| P04114 | 144010 | D006938 | hyperlipoproteinemia type ii | exact |
| P04156 | 123400 | D007562 | creutzfeldt-jakob syndrome | exact |
| P04156 | 600072 | D034062 | insomnia, fatal familial | exact |
| P04156 | 137440 | D016098 | gerstmann-straussler-scheinker disease | exact |
| P04156 | 245300 | D007729 | kuru | exact |
| P04156 | 606688 | D017096 | prion diseases | -2.31680071070762 |
| P04180 | 136120 | D007863 | lecithin acyltransferase deficiency | exact |
| P04180 | 245900 | D007863 | lecithin acyltransferase deficiency | exact |
| P04181 | 258870 | D015799 | gyrate atrophy | exact |
| P04198 | 164280 | D014138 | tracheoesophageal fistula | 0.225445839318967 |
| P04229 | 181000 | D012507 | sarcoidosis | exact |
| P04259 | 167210 | D053549 | pachyonychia congenita | exact |
| P04264 | 113800 | D017488 | hyperkeratosis, epidermolytic | exact |
| P04264 | 600962 | D007645 | keratoderma, palmoplantar | 3.129375028992 |
| P04264 | 607602 | D017488 | hyperkeratosis, epidermolytic | -0.172811823465093 |
| P04264 | 607654 | D007645 | keratoderma, palmoplantar | -0.141989018853866 |
| P04275 | 193400 277480 | D014842 | von willebrand disease | exact |
| P04424 | 207900 | D020159 | citrullinemia | -0.138371666673951 |
| P04629 | 256800 | D009477 | hereditary sensory and autonomic neuropathies | exact |
| P04629 | 188550 | D002291 | carcinoma, papillary | 3.4424308069019 |
| P04629 | 188550 | D002291 | carcinoma, papillary | 3.4424308069019 |
| P04637 | 133239 | D002294 / D004938 | carcinoma, squamous cell / esophageal neoplasms | 4.49953163982265 / exact |
| P04637 | 151623 | D016864 | li-fraumeni syndrome | exact |
| P04637 | 161550 | D009302 / D009303 | nasopharyngeal diseases / nasopharyngeal neoplasms | -0.472653065312674 / exact |
| P04637 | - | D008679 | metaplasia | -0.0963225389711999 |
| P04637 | 275355 | D002294 | carcinoma, squamous cell | 1.29152651980015 |
| P04637 | - | D002294 | carcinoma, squamous cell | 4.52772362236314 |
| P04637 | 211980 | D008175 | lung neoplasms | exact |
| P04637 | 260500 | D020288 | papilloma, choroid plexus | exact |
| P04792 | 606595 | D002607 | charcot-marie-tooth disease | 3.07900563889405 |
| P04792 | 608634 | D015417 / D002607 | hereditary motor and sensory neuropathies / charcot-marie-tooth disease | 1.69157942167269 / -1.44872029615095 |
| P04839 | 306400 | D006105 | granulomatous disease, chronic | 4.0517498484045 |
| P05019 | 608747 | D008607 | mental retardation | -0.516026861676404 |
| P05062 | 229600 | D005633 | fructose intolerance | 5.5988568440925 |
| P05067 | 104300 | D000544 | alzheimer disease | exact |
| P05067 | 609065 | D028243 | cerebral amyloid angiopathy, familial | -0.985976071171474 |
| P05089 | 207800 | D020162 | hyperargininemia | exact |
| P05093 | 202110 | D000312 | adrenal hyperplasia, congenital | 0.648481278792638 |
| P05106 | 273800 | D013915 | thrombasthenia | exact |
| P05107 | 116920 | D018370 | leukocyte-adhesion deficiency syndrome | 2.39071704391261 |
| P05108 | - | D000309 | adrenal insufficiency | 4.50457435237104 |
| P05108 | 201710 | D000312 | adrenal hyperplasia, congenital | 4.08438904594334 |
| P05112 | 601367 | D020521 | cerebrovascular accident | exact |
| P05121 | 188050 | D019851 | thrombophilia | exact |
| P05129 | 605361 | D020754 | spinocerebellar ataxias | 2.7483220207523 |
| P05155 | 106100 | D000799 | angioneurotic edema | 7.0722029042049 |
| P05156 | 235400 | D006463 | hemolytic-uremic syndrome | 5.20955669253049 |
| P05186 | 241500 | D007014 | hypophosphatasia | 2.19085466160639 |
| P05186 | 241510 | D007014 | hypophosphatasia | 2.301670515018 |
| P05186 | 146300 | D007014 | hypophosphatasia | 1.91156111895796 |
| P05231 | 604302 | D001171 | arthritis, juvenile rheumatoid | 5.73783349106195 |
| P05546 | 142360 188050 | D019851 | thrombophilia | exact |
| P05783 | 215600 | D005355 | fibrosis | 1.84684907013102 |
| P05787 | 215600 | D005355 | fibrosis | 1.84684907013102 |
| P05813 | - | D002386 | cataract | -1.05521199484682 |
| P05997 | 130000 | D004535 | ehlers-danlos syndrome | 2.95173134906298 |
| P05997 | 130010 | D004535 | ehlers-danlos syndrome | 2.93456411516712 |
| P06132 | 176100 | D017119 | porphyria cutanea tarda | exact |
| P06132 | 176100 | D017121 / D017119 | porphyria, hepatoerythropoietic / porphyria cutanea tarda | exact / exact |
| P06213 | 125853 | D007333 / D003924 | insulin resistance / diabetes mellitus, type 2 | exact / exact |
| P06213 | 125853 | D003924 | diabetes mellitus, type 2 | exact |
| P06213 | 609968 256450 | D046768 | nesidioblastosis | exact |
| P06213 | 610549 | D000052 | acanthosis nigricans | -2.35086138610088 |
| P06239 | - | D007938 | leukemia | exact |
| P06280 | 301500 | D000795 | fabry disease | exact |
| P06396 | 105120 | D028226 / D000686 | amyloidosis, familial / amyloidosis | 0.012381611093963 / -0.0403092301041417 |
| P06400 | 180200 | D012175 | retinoblastoma | exact |
| P06400 | 109800 | D001749 | urinary bladder neoplasms | exact |
| P06400 | 259500 | D012516 | osteosarcoma | exact |
| P06733 | - | D050031 | hashimoto disease | exact |
| P06737 | 232700 | D005953 / D006013 | glycogen storage disease type i / glycogen storage disease type vi | 2.97344823971241 / exact |
| P06744 | - | D000746 | anemia, hemolytic, congenital nonspherocytic | 4.73129181673449 |
| P06748 | - | D008228 | lymphoma, non-hodgkin | 5.72497016853913 |
| P06748 | - | D015473 | leukemia, promyelocytic, acute | 5.87638847914418 |
| P06748 | - | D009190 | myelodysplastic syndromes | exact |
| P06748 | - | D007950 | leukemia, myelocytic, acute | exact |
| P06753 | 609284 | D017696 | myopathies, nemaline | 4.82495552883605 |
| P06753 | 188550 | D002291 | carcinoma, papillary | 3.4424308069019 |
| P06858 | 238600 | D008072 | hyperlipoproteinemia type i | exact |
| P06858 | 238600 | D008072 | hyperlipoproteinemia type i | exact |
| P06865 | 272800 | D013661 | tay-sachs disease | exact |
| P07101 | 605407 | D009394 / D020734 | nephritis, hereditary / parkinsonian disorders | -1.93334672213838 / 4.37337621980393 |
| P07196 | 607734 | D002607 | charcot-marie-tooth disease | 3.03516657125508 |
| P07196 | 607684 | D002607 | charcot-marie-tooth disease | 3.07900563889405 |
| P07204 | 188040 | D009203 | myocardial infarction | 4.41555227952546 |
| P07225 | 176880 | D018455 | protein s deficiency | exact |
| P07315 | 604219 | D002386 | cataract | -1.79112355822367 |
| P07315 | - | D002386 | cataract | -1.05521199484682 |
| P07320 | 604219 | D002386 | cataract | -1.79112355822367 |
| P07339 | 610127 | D009472 | neuronal ceroid-lipofuscinoses | 6.88304706586268 |
| P07359 | 231200 | D001606 | bernard-soulier syndrome | exact |
| P07359 | 153670 | D001606 | bernard-soulier syndrome | 1.33878869043755 |
| P07359 | 177820 | D014842 | von willebrand disease | exact |
| P07359 | 258660 | D018917 | optic neuropathy, ischemic | 6.34523126560007 |
| P07477 | 167800 | D010195 / D050500 | pancreatitis / pancreatitis, chronic | 2.16212657391681 / exact |
| P07510 | 265000 | D011625 | pterygium | 3.9305434529977 |
| P07585 | 610048 | D003317 | corneal dystrophies, hereditary | 6.00844032164978 |
| P07602 | 249900 | D007966 | leukodystrophy, metachromatic | 4.70229012563054 |
| P07602 | 610539 | D005776 | gaucher disease | 2.32382184093873 |
| P07602 | - | D013661 | tay-sachs disease | 9.06495595142385 |
| P07686 | 268800 | D012497 | sandhoff disease | exact |
| P07902 | 230400 | D005693 | galactosemias | exact |
| P07911 | 162000 | D006073 | gout | -2.03565253012741 |
| P07911 | 603860 | D052177 | kidney diseases, cystic | 1.43242608655702 |
| P07949 | 155240 | D018276 | carcinoma, medullary | 3.60040786967937 |
| P07949 | 171400 | D018761 / D018813 | multiple endocrine neoplasia type 1 / multiple endocrine neoplasia type 2a | -1.02566208457419 / exact |
| P07949 | 162300 | D018761 / D018814 | multiple endocrine neoplasia type 1 / multiple endocrine neoplasia type 2b | -0.950021392069084 / exact |
| P07949 | 142623 | D006627 | hirschsprung disease | exact |
| P07949 | 188550 | D002291 | carcinoma, papillary | 3.4424308069019 |
| P07949 | 209880 | D020919 | sleep disorders, intrinsic | 2.37837563432222 |
| P07951 | 609285 | D017696 | myopathies, nemaline | 4.18862380085824 |
| P07954 | 150800 | D007889 | leiomyoma | -2.1809587910463 |
| P07988 | 265120 | D011649 | pulmonary alveolar proteinosis | 6.82553556739271 |
| P08034 | 302800 | D002607 / D015417 | charcot-marie-tooth disease / hereditary motor and sensory neuropathies | 0.99384531627708 / 3.41338872572424 |
| P08034 | 145900 | D015417 | hereditary motor and sensory neuropathies | exact |
| P08100 | 180380 | D012174 | retinitis pigmentosa | 4.21611118758889 |
| P08100 | 268000 | D012174 | retinitis pigmentosa | exact |
| P08123 | 130060 | D004535 | ehlers-danlos syndrome | 1.12898291585315 |
| P08123 | 166200 | D010013 | osteogenesis imperfecta | 3.54471768916804 |
| P08123 | 166210 | D010013 | osteogenesis imperfecta | 4.39110395953738 |
| P08123 | 225320 | D004535 | ehlers-danlos syndrome | 1.59336853483062 |
| P08123 | 259420 | D010013 | osteogenesis imperfecta | 1.96499238705526 |
| P08123 | 166220 | D010013 | osteogenesis imperfecta | 1.70354146465198 |
| P08235 | 177735 | D011546 | pseudohypoaldosteronism | exact |
| P08236 | 253220 | D016538 | mucopolysaccharidosis vii | exact |
| P08236 | 236750 | D015160 | hydrops fetalis | exact |
| P08237 | 232800 | D005953 / D006014 | glycogen storage disease type i / glycogen storage disease type vii | 2.82887015792151 / exact |
| P08246 | 162800 | D009503 | neutropenia | -0.716479703638052 |
| P08253 | 605156 | D031845 | hajdu-cheney syndrome | -0.786974673778079 |
| P08397 | 176000 | D017118 | porphyria, acute intermittent | exact |
| P08473 | - | D015447 | leukemia, lymphocytic, acute | 4.36202189218039 |
| P08514 | 273800 | D013915 | thrombasthenia | exact |
| P08519 | - | D050197 | atherosclerosis | exact |
| P08559 | 312170 | D015325 | pyruvate dehydrogenase complex deficiency disease | 6.7345223832546 |
| P08559 | 308930 | D007888 | leigh disease | 0.219807704914604 |
| P08579 | - | D008180 | lupus erythematosus, systemic | 2.34614781326417 |
| P08581 | - | D013274 | stomach neoplasms | exact |
| P08581 | 114550 | D006528 | carcinoma, hepatocellular | exact |
| P08581 | 605074 | D002292 | carcinoma, renal cell | exact |
| P08581 | - | D001321 | autistic disorder | -2.46740491396537 |
| P08590 | 608751 192600 | D024741 | cardiomyopathy, hypertrophic, familial | exact |
| P08590 | 608751 | D024741 | cardiomyopathy, hypertrophic, familial | 4.66861836621402 |
| P08603 | 609814 | D015432 | glomerulonephritis, membranoproliferative | -1.09924607773674 |
| P08603 | 235400 | D006463 | hemolytic-uremic syndrome | exact |
| P08603 | 610698 | D008268 | macular degeneration | -1.72856131185579 |
| P08621 | - | D008180 | lupus erythematosus, systemic | 0.119886167115403 |
| P08686 | 201910 | D000312 | adrenal hyperplasia, congenital | 5.54366461230625 |
| P08779 | 167200 | D053549 | pachyonychia congenita | exact |
| P08779 | 600962 | D007645 | keratoderma, palmoplantar | 3.129375028992 |
| P08779 | 144200 | D053546 | keratoderma, palmoplantar, epidermolytic | exact |
| P08842 | 308100 | D016114 | ichthyosis, x-linked | exact |
| P08922 | - | D005909 | glioblastoma | -1.72971580931865 |
| P09417 | 261630 | D010661 | phenylketonurias | exact |
| P09467 | 229700 | D015319 | fructose-1,6-diphosphatase deficiency | exact |
| P09493 | 115196 192600 | D024741 | cardiomyopathy, hypertrophic, familial | exact |
| P09619 | - | D015477 | leukemia, myelomonocytic, chronic | 5.57615825638577 |
| P09619 | - | D007950 | leukemia, myelocytic, acute | exact |
| P09619 | - | D015479 | leukemia, myelomonocytic, acute | 0.960874583246403 |
| P09622 | - | D000140 | acidosis, lactic | 1.37945446835121 |
| P09622 | 248600 | D008375 | maple syrup urine disease | exact |
| P09758 | 204870 | D000686 | amyloidosis | 0.523146826136967 |
| P09848 | 223000 223100 | D007787 | lactose intolerance | exact |
| P0C1Z6 | - | D015452 | leukemia, pre-b-cell | 2.9366553525661 |
| P10070 | 610829 | D016142 | holoprosencephaly | 0.435633342938899 |
| P10071 | 174200 | D017689 | polydactyly | -0.89213565447228 |
| P10071 | 174700 | D013576 | syndactyly | -1.29248125036058 |
| P10253 | 232300 | D005953 / D006009 | glycogen storage disease type i / glycogen storage disease type ii | 3.42956964676876 / exact |
| P10275 | 300068 | D013734 | androgen-insensitivity syndrome | exact |
| P10275 | 313200 | D009134 | muscular atrophy, spinal | 6.98972689181041 |
| P10275 | - | D011471 | prostatic neoplasms | 2.01548173001471 |
| P10275 | 308370 | D007248 | infertility, male | 6.06455077563211 |
| P10275 | 312300 | D013734 | androgen-insensitivity syndrome | exact |
| P10276 | - | D015473 | leukemia, promyelocytic, acute | exact |
| P10415 | 151430 | D008224 | lymphoma, follicular | exact |
| P10451 | - | D014545 | urinary calculi | 0.408155929295619 |
| P10515 | 245348 | D015325 | pyruvate dehydrogenase complex deficiency disease | -0.0639200551035621 |
| P10523 | - | D014605 | uveitis | 1.52944684452678 |
| P10619 | 256540 | D016537 | gangliosidosis, gm1 | 1.68976730564186 |
| P10636 | 600274 172700 | D003704 | dementia | exact |
| P10636 | 601104 260540 | D013494 | supranuclear palsy, progressive | exact |
| P10644 | 255960 | D009232 | myxoma | -1.5 |
| P10644 | 610489 | D000224 | addison disease | -2.49323240501931 |
| P10721 | 172800 | D016116 | piebaldism | exact |
| P10721 | 606764 | D046152 | gastrointestinal stromal tumors | exact |
| P10721 | 273300 | D013733 / D018239 | testicular diseases / seminoma | -0.951256292962171 / exact |
| P10746 | 263700 | D017092 | porphyria, erythropoietic | exact |
| P10746 | 236750 | D015160 | hydrops fetalis | exact |
| P10809 | 605280 | D010264 / D015419 | paraplegia / spastic paraplegia, hereditary | 0.501380847672458 / 3.17233428690669 |
| P10828 | 188570 274300 | D018382 | thyroid hormone resistance syndrome | exact |
| P10912 | 262500 | D046150 | laron syndrome | exact |
| P10914 | - | D007950 | leukemia, myelocytic, acute | -1.15712752799632 |
| P10916 | 608758 192600 | D024741 | cardiomyopathy, hypertrophic, familial | exact |
| P10916 | 608758 | D024741 | cardiomyopathy, hypertrophic, familial | 4.72608525168867 |
| P11021 | 180300 | D001172 | arthritis, rheumatoid | exact |
| P11161 | 605253 | D002607 | charcot-marie-tooth disease | 2.91233897222738 |
| P11161 | 607678 | D002607 / D015417 | charcot-marie-tooth disease / hereditary motor and sensory neuropathies | 3.22475049188007 / 4.72141815700487 |
| P11161 | 145900 | D015417 | hereditary motor and sensory neuropathies | exact |
| P11168 | 227810 | D006008 | glycogen storage disease | 3.2544647089533 |
| P11171 | 130500 | D004612 | elliptocytosis, hereditary | exact |
| P11177 | 312170 | D015325 | pyruvate dehydrogenase complex deficiency disease | 6.7345223832546 |
| P11182 | 248600 | D008375 | maple syrup urine disease | exact |
| P11217 | 232600 | D005953 / D006012 | glycogen storage disease type i / glycogen storage disease type v | 3.08240931456224 / exact |
| P11230 | 601462 254200 | D020294 | myasthenic syndromes, congenital | exact |
| P11230 | 608931 | D020294 | myasthenic syndromes, congenital | 2.43039544390362 |
| P11245 | - | D008180 | lupus erythematosus, systemic | 3.19425465741202 |
| P11274 | 608232 | D015464 | leukemia, myeloid, chronic | exact |
| P11277 | 182870 130600 | D004612 | elliptocytosis, hereditary | -2.13187906901289 |
| P11277 | 182870 | D004612 | elliptocytosis, hereditary | -2.4161112960526 |
| P11308 | 133450 | D012512 | sarcoma, ewing's | exact |
| P11308 | - | D007950 | leukemia, myelocytic, acute | 4.54951692806327 |
| P11362 | 101600 | D000168 | acrocephalosyndactylia | exact |
| P11362 | 146110 | D007006 | hypogonadism | exact |
| P11362 | 147950 | D017436 | kallmann syndrome | exact |
| P11362 | 166250 | D004392 | dwarfism | -2.07487355975234 |
| P11362 | 190440 | D003398 | craniosynostoses | -2.27729442583882 |
| P11362 | - | D015460 | leukemia-lymphoma, t-cell, acute, htlv-i-associated | -2.17019389346424 |
| P11362 | - | D009196 | myeloproliferative disorders | 0.609720507513883 |
| P11387 | - | D009190 | myelodysplastic syndromes | -1.94047015761774 |
| P11413 | 305900 | D000746 | anemia, hemolytic, congenital nonspherocytic | -0.748767879401284 |
| P11473 | 277440 | D006938 / D053098 | hyperlipoproteinemia type ii / hypophosphatemic rickets, x-linked dominant | -1.85298909739607 / 6.06673111920497 |
| P11498 | 266150 | D015324 | pyruvate carboxylase deficiency disease | 9.02590528987048 |
| P11511 | 139300 | D006177 | gynecomastia | 2.96536866878144 |
| P11532 | 310200 | D020388 | muscular dystrophy, duchenne | exact |
| P11532 | 300376 | D020388 | muscular dystrophy, duchenne | exact |
| P11532 | 302045 | D002311 | cardiomyopathy, dilated | exact |
| P11586 | 601634 | D009436 | neural tube defects | 0.874749199640761 |
| P11686 | 265120 | D011649 | pulmonary alveolar proteinosis | 6.82553556739271 |
| P11686 | 178500 | D011658 | pulmonary fibrosis | 6.48937991723684 |
| P11686 | - | D020151 | protein c deficiency | 0.402658267991717 |
| P11802 | - | D009369 | neoplasms | -1.64725631865348 |
| P11802 | 609048 | D008545 | melanoma | 0.62799634458515 |
| P12004 | - | D008180 | lupus erythematosus, systemic | exact |
| P12035 | 122100 | D053559 | corneal dystrophy, juvenile epithelial of meesmann | exact |
| P12036 | 105400 | D000690 | amyotrophic lateral sclerosis | exact |
| P12109 | 158810 | D009136 | muscular dystrophies | 0.116191121009144 |
| P12110 | 158810 | D009136 | muscular dystrophies | 0.116191121009144 |
| P12110 | 254090 | D009136 | muscular dystrophies | 0.800241707918547 |
| P12111 | 158810 | D009136 | muscular dystrophies | 0.116191121009144 |
| P12111 | 254090 | D009136 | muscular dystrophies | 0.800241707918547 |
| P12235 | 609283 | D017246 | ophthalmoplegia, chronic progressive external | 1.35959893395475 |
| P12259 | 227400 | D005166 | factor v deficiency | exact |
| P12259 | 188055 | D020016 | activated protein c resistance | exact |
| P12259 | 600880 | D006502 | hepatic vein thrombosis | exact |
| P12259 | 601367 | D020521 | cerebrovascular accident | exact |
| P12270 | 188550 | D002291 | carcinoma, papillary | 3.4424308069019 |
| P12271 | 268000 | D012174 | retinitis pigmentosa | exact |
| P12271 | 607476 | D012174 | retinitis pigmentosa | 3.88859760347527 |
| P12694 | 248600 | D008375 | maple syrup urine disease | exact |
| P12821 | 601367 | D020521 | cerebrovascular accident | exact |
| P12830 | 137215 | D013274 | stomach neoplasms | exact |
| P12830 | 608089 | D016889 | endometrial neoplasms | exact |
| P12883 | 192600 | D024741 | cardiomyopathy, hypertrophic, familial | exact |
| P12883 | 608358 | D017696 | myopathies, nemaline | -2.31953083371198 |
| P12883 | 115200 | D002311 | cardiomyopathy, dilated | exact |
| P12883 | 160500 | D049310 | distal myopathies | 4.15259769104974 |
| P12980 | - | D015459 | leukemia, t-cell, acute | 6.11107035394932 |
| P13051 | 608106 | D053306 | hyper-igm immunodeficiency syndrome | exact |
| P13224 | 231200 | D001606 | bernard-soulier syndrome | exact |
| P13473 | 300257 | D052120 | glycogen storage disease type iib | exact |
| P13498 | 233690 | D006105 | granulomatous disease, chronic | 2.35877943146277 |
| P13533 | 192600 | D024741 | cardiomyopathy, hypertrophic, familial | exact |
| P13533 | 160710 | D006344 | heart septal defects, atrial | 5.24096872404226 |
| P13569 | 219700 | D003550 | cystic fibrosis | exact |
| P13637 | 128235 | D004421 | dystonia | -0.437910294389635 |
| P13645 | 113800 | D017488 | hyperkeratosis, epidermolytic | exact |
| P13645 | 607602 | D017488 | hyperkeratosis, epidermolytic | -0.172811823465093 |
| P13645 | 600648 | D017488 | hyperkeratosis, epidermolytic | 0.669549994899532 |
| P13646 | 193900 | D053529 | leukokeratosis, hereditary mucosal | exact |
| P13647 | 131760 131800 131900 131960 | D016110 / D016108 | epidermolysis bullosa simplex / epidermolysis bullosa dystrophica | exact / exact |
| P13647 | 131760 | D016110 | epidermolysis bullosa simplex | 10.3902777727887 |
| P13647 | 609352 | D016110 | epidermolysis bullosa simplex | -1.67104476948927 |
| P13647 | 131800 | D016110 / D016108 | epidermolysis bullosa simplex / epidermolysis bullosa dystrophica | 0.396432498403869 / exact |
| P13647 | 131900 | D016110 | epidermolysis bullosa simplex | 2.36143982935086 |
| P13647 | 131960 | D016110 | epidermolysis bullosa simplex | 1.40352378528493 |
| P13716 | 125270 | D017094 | porphyrias, hepatic | 4.57281365578536 |
| P13805 | 605355 605355 | D017696 | myopathies, nemaline | 4.14067854333369 |
| P13942 | 184840 | D052245 | usher syndromes | -0.857272580145866 |
| P13942 | 277610 | D010855 | pierre robin syndrome | 2.73395259275044 |
| P14136 | 203450 | D038261 | alexander disease | exact |
| P14138 | 142623 | D006627 | hirschsprung disease | exact |
| P14138 | 209880 | D020919 | sleep disorders, intrinsic | 2.37837563432222 |
| P14138 | 277580 | D014849 | waardenburg's syndrome | 2.47230745042781 |
| P14174 | 604302 | D001171 | arthritis, juvenile rheumatoid | 5.73783349106195 |
| P14222 | 603553 | D051359 | lymphohistiocytosis, hemophagocytic | 6.61793765345078 |
| P14314 | 174050 173900 | D008107 / D007690 | liver diseases / polycystic kidney diseases | 1.37554529844664 / exact |
| P14373 | 188550 | D002291 | carcinoma, papillary | 3.4424308069019 |
| P14416 | 159900 | D009207 / D004421 | myoclonus / dystonia | 0.541708004093818 / -0.113902958761406 |
| P14598 | 233700 | D006105 | granulomatous disease, chronic | -0.967115068515101 |
| P14672 | 125853 | D003924 | diabetes mellitus, type 2 | exact |
| P14679 | 203100 | D016115 | albinism, oculocutaneous | 6.21044324922289 |
| P14679 | 606952 | D016115 | albinism, oculocutaneous | 7.69123409289994 |
| P14679 | 606952 | D016115 | albinism, oculocutaneous | 7.69123409289994 |
| P14770 | 231200 | D001606 | bernard-soulier syndrome | exact |
| P14867 | 606904 | D020190 | myoclonic epilepsy, juvenile | exact |
| P14923 | 601214 | D019571 | arrhythmogenic right ventricular dysplasia | -0.629197901349414 |
| P15056 | 211980 | D008175 | lung neoplasms | exact |
| P15056 | 605027 | D008228 | lymphoma, non-hodgkin | exact |
| P15144 | - | D008223 | lymphoma | -2.36998957665944 |
| P15259 | 261670 | D009135 | muscular diseases | 0.567408291314187 |
| P15260 | 209950 | D012480 | salmonella infections | -1.53648303645513 |
| P15289 | 250100 | D007966 | leukodystrophy, metachromatic | exact |
| P15289 | 272200 | D052517 | multiple sulfatase deficiency disease | exact |
| P15313 | 267300 | D000141 | acidosis, renal tubular | 6.83695652455818 |
| P15374 | - | D003110 | colonic neoplasms | exact |
| P15382 | 220400 | D029593 | jervell-lange nielsen syndrome | exact |
| P15382 | 176261 | D008133 | long qt syndrome | 1.69094708698018 |
| P15502 | 123700 | D003483 | cutis laxa | 2.74566027365228 |
| P15502 | 194050 | D018980 | williams syndrome | exact |
| P15502 | 185500 | D021921 | aortic stenosis, supravalvular | exact |
| P15529 | 235400 | D006463 | hemolytic-uremic syndrome | 5.20955669253049 |
| P15538 | 202010 | D000312 | adrenal hyperplasia, congenital | 0.818898883638687 |
| P15538 | 103900 | D006929 | hyperaldosteronism | -2.19633021290335 |
| P15586 | 252940 | D009084 | mucopolysaccharidosis iii | 3.05564803275837 |
| P15735 | 172471 | D005953 / D006008 | glycogen storage disease type i / glycogen storage disease | 1.49157005248487 / 2.71198345859273 |
| P15848 | 253200 | D009087 | mucopolysaccharidosis vi | exact |
| P15848 | 272200 | D052517 | multiple sulfatase deficiency disease | exact |
| P15918 | 601457 | D016511 | severe combined immunodeficiency | 0.266668263748314 |
| P15918 | 603554 | D016511 | severe combined immunodeficiency | 3.43786019655994 |
| P15923 | - | D015452 | leukemia, pre-b-cell | 1.27065599366172 |
| P15924 | 125647 | D007645 | keratoderma, palmoplantar | 0.114987480288605 |
| P15924 | 607450 | D019571 | arrhythmogenic right ventricular dysplasia | 6.74022140502188 |
| P15924 | 609638 | D016109 | epidermolysis bullosa, junctional | 1.13332012875727 |
| P15976 | 300367 | D000742 | anemia, dyserythropoietic, congenital | 0.952912970720499 |
| P15976 | 314050 | D017086 | beta-thalassemia | -1.42953685390669 |
| P16109 | 601367 | D020521 | cerebrovascular accident | exact |
| P16112 | 608361 | D010009 | osteochondrodysplasias | -0.258772666117091 |
| P16144 | 226730 | D016109 / D004476 | epidermolysis bullosa, junctional / ectodermal dysplasia | 2.53168983108552 / 2.68368487528973 |
| P16144 | 226650 | D016110 | epidermolysis bullosa simplex | -0.386916265556435 |
| P16150 | - | D014923 | wiskott-aldrich syndrome | 3.07807611276619 |
| P16157 | 182900 | D013103 | spherocytosis, hereditary | exact |
| P16234 | 607685 | D017681 | hypereosinophilic syndrome | exact |
| P16260 | - | D006111 | graves disease | exact |
| P16278 | 230500 | D016537 | gangliosidosis, gm1 | 8.44402237242076 |
| P16278 | 230600 | D016537 | gangliosidosis, gm1 | 3.85072794433202 |
| P16278 | 230650 | D016537 | gangliosidosis, gm1 | 3.59375144518955 |
| P16278 | 253010 | D009085 | mucopolysaccharidosis iv | exact |
| P16410 | 152700 | D008180 | lupus erythematosus, systemic | exact |
| P16410 | 275000 | D006111 | graves disease | exact |
| P16410 | 601388 | D003922 | diabetes mellitus, type 1 | 6.52672319885964 |
| P16410 | 609755 212750 | D002446 | celiac disease | exact |
| P16435 | 201750 | D000312 | adrenal hyperplasia, congenital | -1.05682226484886 |
| P16452 | - | D013103 | spherocytosis, hereditary | exact |
| P16473 | 603372 | D007037 / D013964 | hypothyroidism / thyroid neoplasms | -1.01781195486536 / 1.75211173814016 |
| P16473 | 603372 | D006980 / D013964 | hyperthyroidism / thyroid neoplasms | exact / 1.75211173814016 |
| P16473 | 275200 | D006964 | hyperpituitarism | -0.50480385847822 |
| P16473 | - | D013964 | thyroid neoplasms | exact |
| P16473 | 275000 | D006111 | graves disease | exact |
| P16473 | 609152 | D006980 | hyperthyroidism | -1.19615871138938 |
| P16499 | 268000 | D012174 | retinitis pigmentosa | exact |
| P16615 | 124200 | D007644 | keratosis follicularis | exact |
| P16930 | 276700 | D020176 | tyrosinemias | exact |
| P17050 | 609241 | D019150 | neuroaxonal dystrophies | 0.176467378485099 |
| P17302 | 186100 | D013576 | syndactyly | -1.41120504597346 |
| P17302 | 241550 | D018636 | hypoplastic left heart syndrome | exact |
| P17405 | 257200 | D052536 | niemann-pick disease, type a | exact |
| P17405 | 607616 | D052537 | niemann-pick disease, type b | exact |
| P17542 | - | D015459 | leukemia, t-cell, acute | exact |
| P17643 | 278400 | D016115 | albinism, oculocutaneous | 2.60893271514884 |
| P17643 | 203290 | D016115 | albinism, oculocutaneous | 2.01315365654086 |
| P17661 | 604765 | D002311 | cardiomyopathy, dilated | 1.92947071162984 |
| P17735 | 276600 | D020176 | tyrosinemias | exact |
| P17787 | 605375 | D017034 | epilepsy, frontal lobe | 1.1840406122729 |
| P17813 | 187300 | D013683 | telangiectasia, hereditary hemorrhagic | 3.50676973610655 |
| P17900 | 272750 | D049290 | tay-sachs disease, ab variant | exact |
| P18074 | 278730 | D014983 | xeroderma pigmentosum | 4.90027470238313 |
| P18074 | 278730 | D014983 | xeroderma pigmentosum | 4.90027470238313 |
| P18135 | - | D015462 | leukemia, lymphocytic, chronic | exact |
| P18136 | - | D015462 | leukemia, lymphocytic, chronic | exact |
| P18464 | - | D001528 | behcet syndrome | exact |
| P18507 | 607681 | D004832 | epilepsy, absence | 5.20766802907419 |
| P18507 | 604233 | D003294 | seizures, febrile | 1.46855467728936 |
| P18507 | 607208 | D004831 | epilepsies, myoclonic | 1.82178487470868 |
| P19013 | 193900 | D053529 | leukokeratosis, hereditary mucosal | exact |
| P19099 | 610600 | D030401 | cytochrome-c oxidase deficiency | -2.08949932754148 |
| P19099 | 103900 | D006929 | hyperaldosteronism | -2.19633021290335 |
| P19235 | 133100 | D011086 | polycythemia | -0.61827260855058 |
| P19320 | - | D001172 | arthritis, rheumatoid | -1.8508861695505 |
| P19367 | 235700 | D000743 | anemia, hemolytic | -0.640656903434843 |
| P19429 | 191044 192600 | D024741 | cardiomyopathy, hypertrophic, familial | exact |
| P19429 | 115210 | D002313 | cardiomyopathy, restrictive | 5.46361643171749 |
| P19438 | 142680 | D020821 | dystonic disorders | -1.66403748393148 |
| P19447 | - | D014983 | xeroderma pigmentosum | -0.92494065971229 |
| P19447 | - | D003057 | cockayne syndrome | -1.27989702739426 |
| P19532 | 606243 | D018234 | sarcoma, alveolar soft part | exact |
| P19532 | - | D002292 | carcinoma, renal cell | exact |
| P19532 | - | D018234 | sarcoma, alveolar soft part | exact |
| P19544 | 136680 | D052159 | frasier syndrome | exact |
| P19544 | 194070 | D009396 | wilms tumor | exact |
| P19544 | 194080 | D030321 | denys-drash syndrome | exact |
| P19544 | 256370 | D002549 | diffuse cerebral sclerosis of schilder | -1.01532145366225 |
| P19622 | - | D001321 | autistic disorder | -2.48977631258489 |
| P19835 | 609812 606391 | D003924 | diabetes mellitus, type 2 | exact |
| P19878 | 233710 | D006105 | granulomatous disease, chronic | 2.35877943146277 |
| P19971 | 603041 | D017237 / D007418 | mitochondrial encephalomyopathies / intestinal pseudo-obstruction | 2.85002960090521 / -0.226811791615573 |
| P20226 | 607136 | D020754 | spinocerebellar ataxias | 2.73995836043136 |
| P20292 | 601367 | D020521 | cerebrovascular accident | exact |
| P20292 | 608557 | D009203 | myocardial infarction | exact |
| P20585 | 608089 | D016889 | endometrial neoplasms | exact |
| P20700 | 169500 | D020371 | pelizaeus-merzbacher disease | -2.01350786533445 |
| P20749 | - | D015451 | leukemia, b-cell, chronic | exact |
| P20807 | 253600 | D049288 | muscular dystrophies, limb-girdle | 4.2997082248349 |
| P20823 | 142330 | D000236 / D018248 | adenoma / adenoma, liver cell | -0.0700888290241304 / 4.97705064245205 |
| P20823 | 600496 606391 | D003924 | diabetes mellitus, type 2 | exact |
| P20823 | - | D003922 | diabetes mellitus, type 1 | exact |
| P20839 | 180105 | D012174 | retinitis pigmentosa | 3.85384291174314 |
| P20849 | 120210 | D010009 | osteochondrodysplasias | 1.21670298592273 |
| P20908 | 130000 | D004535 | ehlers-danlos syndrome | 2.95173134906298 |
| P20908 | 130010 | D004535 | ehlers-danlos syndrome | 2.93456411516712 |
| P20929 | 256030 | D017696 | myopathies, nemaline | 4.71927909701121 |
| P20930 | 146700 | D016112 | ichthyosis vulgaris | exact |
| P20936 | 608354 | D001165 | arteriovenous malformations | 5.1086485413494 |
| P20936 | 608355 | D020526 | brain stem infarctions | 0.686456782221787 |
| P21333 | 309350 | D010009 | osteochondrodysplasias | exact |
| P21333 | 300048 | D007418 | intestinal pseudo-obstruction | 7.33705196841965 |
| P21359 | 162200 | D009456 | neurofibromatosis 1 | exact |
| P21359 | 607785 | D015479 | leukemia, myelomonocytic, acute | 0.960874583246403 |
| P21359 | 193520 | D009456 | neurofibromatosis 1 | exact |
| P21359 | 162210 | D017253 | neurofibromatoses | -2.12214472560095 |
| P21439 | 602347 | D002780 | cholestasis, intrahepatic | -0.282800247979384 |
| P21439 | 147480 | D002780 | cholestasis, intrahepatic | 4.78278957612661 |
| P21439 | 600803 | D002769 / D002764 | cholelithiasis / cholecystitis | exact / exact |
| P21549 | 259900 | D006960 | hyperoxaluria, primary | 2.09718730338726 |
| P21589 | - | D015451 | leukemia, b-cell, chronic | 4.47327176487493 |
| P21673 | 308800 | D007644 | keratosis follicularis | -1.01920389926271 |
| P21675 | 314250 | D004422 | dystonia musculorum deformans | -0.604305345133598 |
| P21802 | 123500 | D003394 | craniofacial dysostosis | 3.62804377350415 |
| P21802 | 101200 | D000168 | acrocephalosyndactylia | exact |
| P21802 | 101600 | D000168 | acrocephalosyndactylia | exact |
| P21817 | 145600 | D008305 | malignant hyperthermia | exact |
| P21817 | 117000 | D020512 | myopathy, central core | 5.18003674458667 |
| P21817 | 255320 | D009886 | ophthalmoplegia | 0.523627472654565 |
| P21860 | - | D001943 | breast neoplasms | 1.96312870849755 |
| P21912 | 171300 | D010673 | pheochromocytoma | exact |
| P21912 | 115310 | D010235 / D010673 | paraganglioma / pheochromocytoma | -2.13112693747584 / 7.07800211680535 |
| P21918 | - | D012559 | schizophrenia | exact |
| P21918 | 606798 | D001764 / D020329 | blepharospasm / essential tremor | exact / -0.735727868037917 |
| P21953 | 248600 | D008375 | maple syrup urine disease | exact |
| P22105 | - | D000312 | adrenal hyperplasia, congenital | exact |
| P22223 | 601553 | D012174 | retinitis pigmentosa | 4.84043633398967 |
| P22301 | 266600 | D003424 | crohn disease | exact |
| P22304 | 309900 | D016532 | mucopolysaccharidosis ii | exact |
| P22309 | 143500 | D005878 / D006932 | gilbert disease / hyperbilirubinemia | 1.59506524721584 / 2.15319362199579 |
| P22309 | 237900 143500 | D051556 / D006932 | hyperbilirubinemia, neonatal / hyperbilirubinemia | 0.854571834156525 / 2.15319362199579 |
| P22309 | 218800 | D003414 | crigler-najjar syndrome | exact |
| P22309 | 606785 | D003414 | crigler-najjar syndrome | 3.92176081659289 |
| P22413 | 602475 | D017887 | ossification of posterior longitudinal ligament | 8.28541397961078 |
| P22413 | 125853 | D003924 | diabetes mellitus, type 2 | exact |
| P22415 | 602491 | D006950 | hyperlipidemia, familial combined | 6.23567314818284 |
| P22557 | 301300 | D000756 / D000747 | anemia, sideroblastic / anemia, hypochromic | 1.56729090549799 / exact |
| P22607 | 100800 | D000130 | achondroplasia | exact |
| P22607 | 123500 | D003394 | craniofacial dysostosis | 3.62804377350415 |
| P22607 | 187600 187601 | D013796 | thanatophoric dysplasia | exact |
| P22607 | 109800 | D001749 | urinary bladder neoplasms | exact |
| P22607 | 603956 | D002583 | uterine cervical neoplasms | 3.96976499306381 |
| P22607 | - | D009101 | multiple myeloma | exact |
| P22607 | 254500 | D009101 | multiple myeloma | exact |
| P22735 | 242300 | D017490 | ichthyosis, lamellar | exact |
| P22735 | 242100 | D016113 / D017490 | ichthyosiform erythroderma, congenital / ichthyosis, lamellar | 2.34951558019456 / 8.18206058101934 |
| P22748 | 600852 | D012174 | retinitis pigmentosa | 3.46106383132363 |
| P22830 | 177000 | D046351 | protoporphyria, erythropoietic | exact |
| P22888 | 176410 | D011629 | puberty, precocious | 2.58703643532612 |
| P22888 | 152790 | D007984 | leydig cell tumor | 1.16104073400473 |
| P22914 | - | D002386 | cataract | -0.580232336096621 |
| P23025 | 278700 | D014983 | xeroderma pigmentosum | 5.62012332093724 |
| P23142 | - | D001943 | breast neoplasms | 2.92211242361713 |
| P23229 | 226730 | D004820 / D004476 | epidermolysis bullosa / ectodermal dysplasia | -1.00864940028252 / 2.68368487528973 |
| P23246 | - | D002292 | carcinoma, renal cell | exact |
| P23352 | 308700 | D017436 | kallmann syndrome | exact |
| P23378 | 605899 | D020158 | hyperglycinemia, nonketotic | exact |
| P23409 | 160150 | D020914 | myopathies, structural, congenital | exact |
| P23415 | 149400 | D013216 / D016750 | startle reaction / stiff-person syndrome | -1.59851261161565 / 6.4222726824933 |
| P23434 | 605899 | D020158 | hyperglycinemia, nonketotic | exact |
| P23497 | - | D008105 | liver cirrhosis, biliary | exact |
| P23560 | 209880 | D020919 | sleep disorders, intrinsic | 2.37837563432222 |
| P23759 | 268220 | D012208 / D018232 | rhabdomyosarcoma / rhabdomyosarcoma, alveolar | 2.44132152468092 / exact |
| P23760 | 193500 | D014849 | waardenburg's syndrome | 0.678021719672773 |
| P23760 | 148820 | D014849 | waardenburg's syndrome | exact |
| P23760 | 268220 | D012208 / D018232 | rhabdomyosarcoma / rhabdomyosarcoma, alveolar | 2.44132152468092 / exact |
| P23760 | - | D012208 | rhabdomyosarcoma | exact |
| P23942 | 608133 | D012174 | retinitis pigmentosa | 3.74560066899646 |
| P23942 | - | D012174 | retinitis pigmentosa | exact |
| P23945 | 233300 | D023961 | gonadal dysgenesis, 46,xx | 3.69839789978662 |
| P23945 | 608115 | D016471 | ovarian hyperstimulation syndrome | exact |
| P23975 | 604715 | D009449 | neurocirculatory asthenia | exact |
| P24385 | - | D020522 | lymphoma, mantle-cell | -2.01724424722143 |
| P24385 | - | D010282 | parathyroid neoplasms | exact |
| P24385 | 254500 | D009101 | multiple myeloma | exact |
| P24386 | 303100 | D015794 | choroideremia | exact |
| P24530 | 277580 | D014849 | waardenburg's syndrome | 2.47230745042781 |
| P24530 | 600155 | D006627 | hirschsprung disease | 3.43402023130986 |
| P24723 | 601367 | D020521 | cerebrovascular accident | exact |
| P24855 | - | D008107 | liver diseases | exact |
| P25054 | 175100 | D011125 | adenomatous polyposis coli | exact |
| P25054 | 135290 | D018222 | fibromatosis, aggressive | 0.34084351531187 |
| P25054 | 155255 | D008527 | medulloblastoma | exact |
| P25054 | 276300 | D016543 | central nervous system neoplasms | -1.50685036263698 |
| P25067 | 122000 | D003317 | corneal dystrophies, hereditary | 0.527397943934729 |
| P25067 | 136800 | D005642 | fuchs' endothelial dystrophy | 6.83164812534167 |
| P25189 | 118200 | D002607 | charcot-marie-tooth disease | exact |
| P25189 | 607677 | D002607 | charcot-marie-tooth disease | 3.03516657125508 |
| P25189 | 607736 | D002607 | charcot-marie-tooth disease | 3.14817855544052 |
| P25189 | 103100 | D015845 | tonic pupil | exact |
| P25189 | 607791 | D002607 | charcot-marie-tooth disease | 0.535115326290013 |
| P25189 | 145900 | D015417 | hereditary motor and sensory neuropathies | exact |
| P25189 | 605253 | D002607 | charcot-marie-tooth disease | 2.91233897222738 |
| P25189 | 180800 | D002607 | charcot-marie-tooth disease | exact |
| P25445 | 601859 | D016884 | polyendocrinopathies, autoimmune | -2.00376642617331 |
| P25791 | - | D015459 | leukemia, t-cell, acute | 6.11107035394932 |
| P25800 | - | D015459 | leukemia, t-cell, acute | 6.11107035394932 |
| P25942 | 606843 | D053306 | hyper-igm immunodeficiency syndrome | exact |
| P26196 | - | D016393 | lymphoma, b-cell | -1.98773679649435 |
| P26367 | 106210 | D015783 | aniridia | -0.305400841385172 |
| P26367 | 148190 | D007634 | keratitis | 2.13922472911024 |
| P26367 | 120200 | D003103 | coloboma | 1.3122454324539 |
| P26367 | 120430 | D009901 | optic nerve diseases | -0.0302973598108445 |
| P26367 | 165550 | D009901 | optic nerve diseases | -2.37729850252739 |
| P26439 | 201810 | D000312 | adrenal hyperplasia, congenital | 1.51013544569969 |
| P26678 | 609909 | D002311 | cardiomyopathy, dilated | 1.73448321138946 |
| P26998 | - | D002386 | cataract | -1.05521199484682 |
| P27352 | 261000 | D005171 | factor x deficiency | -1.92063414774627 |
| P27635 | - | D001321 | autistic disorder | -1.58492988443065 |
| P27986 | - | D007333 | insulin resistance | 4.68502616457109 |
| P28288 | 170995 | D015211 | zellweger syndrome | 3.37654679973432 |
| P28300 | 219100 | D003483 | cutis laxa | 2.81085466105711 |
| P28328 | 170993 | D015211 | zellweger syndrome | 3.18705864837452 |
| P28328 | 214100 | D015211 | zellweger syndrome | exact |
| P28328 | 266510 | D052919 | refsum disease, infantile | exact |
| P28329 | 254210 254200 | D009157 | myasthenia gravis | exact |
| P28331 | 252010 | D028361 | mitochondrial diseases | 3.48654516268259 |
| P28360 | 106600 | D000848 | anodontia | -0.683814193261215 |
| P28360 | 189500 | D000848 | anodontia | -2.2772473809268 |
| P28360 | 608874 | D002972 | cleft palate | -2.43292264273568 |
| P28715 | - | D014983 | xeroderma pigmentosum | -1.58390754651331 |
| P28715 | 216400 | D003057 | cockayne syndrome | exact |
| P29033 | 148350 | D007645 | keratoderma, palmoplantar | 4.9653065757249 |
| P29033 | 148210 | D009394 | nephritis, hereditary | -1.86815233473355 |
| P29323 | 176807 | D011471 | prostatic neoplasms | exact |
| P29323 | 603688 | D011471 | prostatic neoplasms | 2.25373323566112 |
| P29400 | 301050 | D009394 | nephritis, hereditary | exact |
| P29400 | 308940 | D009394 | nephritis, hereditary | -1.53913569738039 |
| P29401 | - | D020915 | korsakoff syndrome | 0.103370064474327 |
| P29460 | 209950 | D012480 | salmonella infections | -1.53648303645513 |
| P29536 | - | D013959 | thyroid diseases | 2.16032848930655 |
| P29590 | - | D015473 | leukemia, promyelocytic, acute | exact |
| P29965 | 308230 | D053307 | hyper-igm immunodeficiency syndrome, type 1 | exact |
| P29973 | 268000 | D012174 | retinitis pigmentosa | exact |
| P30154 | - | D015179 | colorectal neoplasms | 3.08664962429473 |
| P30464 | 608579 | D013262 / D004816 | stevens-johnson syndrome / epidermal necrolysis, toxic | exact / 7.85687128607807 |
| P30518 | 304800 | D018500 | diabetes insipidus, nephrogenic | exact |
| P30530 | - | D015464 | leukemia, myeloid, chronic | 1.64907445714417 |
| P30613 | 266200 | D000746 | anemia, hemolytic, congenital nonspherocytic | 4.70541087280454 |
| P30793 | 233910 | D010661 | phenylketonurias | 5.18637743029369 |
| P30793 | 128230 | D020821 / D004421 | dystonic disorders / dystonia | -1.84582835751808 / 1.10979946384668 |
| P30968 | 146110 | D007006 | hypogonadism | exact |
| P31040 | 252011 | D028361 | mitochondrial diseases | 3.47223913443604 |
| P31040 | 256000 | D007888 | leigh disease | exact |
| P31150 | 309541 | D038901 | mental retardation, x-linked | 5.45948068400117 |
| P31150 | 309541 | D038901 | mental retardation, x-linked | 5.45948068400117 |
| P31213 | 264600 | D011545 | pseudohermaphroditism | -2.07716245295031 |
| P31269 | - | D007950 | leukemia, myelocytic, acute | 1.23080265610631 |
| P31269 | - | D015464 | leukemia, myeloid, chronic | 2.02691617471708 |
| P31314 | - | D015459 | leukemia, t-cell, acute | 6.11107035394932 |
| P31327 | 237300 | D020165 | carbamoyl-phosphate synthase i deficiency disease | 8.24096343054084 |
| P31327 | 265380 | D010547 | persistent fetal circulation syndrome | 7.51679302320109 |
| P31639 | 233100 | D006030 | glycosuria, renal | exact |
| P31751 | - | D002277 | carcinoma | -1.14412248431554 |
| P31785 | 300400 | D053632 | x-linked combined immunodeficiency diseases | exact |
| P31785 | 312863 | D053632 | x-linked combined immunodeficiency diseases | exact |
| P31994 | - | D008224 | lymphoma, follicular | exact |
| P32004 | 303350 | D010264 | paraplegia | 1.76458858679923 |
| P32004 | 307000 | D006849 / D007418 | hydrocephalus / intestinal pseudo-obstruction | exact / 0.744220951478581 |
| P32004 | 303350 | D010264 | paraplegia | 1.76458858679923 |
| P32004 | 303350 | D010264 | paraplegia | 1.76458858679923 |
| P32004 | 142623 | D006627 | hirschsprung disease | exact |
| P32245 | 601665 | D009765 | obesity | exact |
| P32754 | 276710 | D020176 | tyrosinemias | exact |
| P32927 | 265120 | D011649 | pulmonary alveolar proteinosis | 6.82553556739271 |
| P33076 | 209920 | D016511 | severe combined immunodeficiency | exact |
| P33897 | 300100 | D000326 | adrenoleukodystrophy | exact |
| P34059 | 253000 | D009085 | mucopolysaccharidosis iv | 4.71706011769539 |
| P35070 | - | D048909 | diabetes complications | 0.655015372497759 |
| P35222 | - | D009369 | neoplasms | -0.85760944333625 |
| P35222 | 132600 | D018296 | pilomatrixoma | exact |
| P35222 | 155255 | D008527 | medulloblastoma | exact |
| P35232 | - | D001943 | breast neoplasms | 2.42971031682871 |
| P35240 | 101000 | D016518 | neurofibromatosis 2 | exact |
| P35240 | 162091 | D009442 | neurilemmoma | 0.893084796083487 |
| P35243 | - | D019572 | retinal neoplasms | -0.920173288024171 |
| P35354 | - | D001172 | arthritis, rheumatoid | -1.84645513413668 |
| P35453 | 186000 | D013576 | syndactyly | -1.0685697137835 |
| P35453 | 186300 | D013576 | syndactyly | -1.42647294251117 |
| P35462 | 190300 | D020329 | essential tremor | 1.72781029048427 |
| P35475 | 607014 | D008059 | mucopolysaccharidosis i | exact |
| P35475 | 607015 | D008059 | mucopolysaccharidosis i | exact |
| P35475 | 607016 | D008059 | mucopolysaccharidosis i | exact |
| P35498 | 604233 | D003294 | seizures, febrile | 1.46855467728936 |
| P35498 | 607208 | D004831 | epilepsies, myoclonic | 1.82178487470868 |
| P35498 | 609634 | D020325 | migraine with aura | 6.39547694191478 |
| P35498 | 604403 | D003294 | seizures, febrile | 1.59038113324142 |
| P35499 | 168300 | D020967 | myotonic disorders | -0.204856938589545 |
| P35499 | 170400 | D020514 | hypokalemic periodic paralysis | exact |
| P35499 | 170500 | D020513 | paralysis, hyperkalemic periodic | exact |
| P35499 | 608390 | D020967 | myotonic disorders | exact |
| P35499 | 603967 | D020294 | myasthenic syndromes, congenital | exact |
| P35520 | 236200 603174 | D006712 | homocystinuria | exact |
| P35523 | 160800 | D009224 | myotonia congenita | exact |
| P35523 | 255700 | D009224 | myotonia congenita | exact |
| P35527 | 144200 | D053546 | keratoderma, palmoplantar, epidermolytic | exact |
| P35548 | 168500 | D016136 | spina bifida occulta | -0.0945976553335299 |
| P35548 | 168550 | D002973 | cleidocranial dysplasia | -1.01591495416628 |
| P35548 | 604757 | D003398 | craniosynostoses | -0.87959146651376 |
| P35555 | 154700 | D008382 | marfan syndrome | exact |
| P35555 | 129600 | D004479 | ectopia lentis | 7.10522059958608 |
| P35555 | 182212 | D046449 | hernia, abdominal | -1.04418254483686 |
| P35555 | 604308 | D008399 / D003240 | mass behavior / connective tissue diseases | -1.12349867492057 / 3.8552907219196 |
| P35557 | 125851 606391 | D003924 / D016640 | diabetes mellitus, type 2 / diabetes, gestational | -0.973992545234476 / exact |
| P35568 | 125853 | D003924 | diabetes mellitus, type 2 | exact |
| P35573 | 232400 | D005953 / D006010 | glycogen storage disease type i / glycogen storage disease type iii | 3.31587675595288 / exact |
| P35575 | 232200 | D005953 | glycogen storage disease type i | exact |
| P35579 | 153650 | D009394 | nephritis, hereditary | 1.52297514824283 |
| P35579 | 153650 | D009394 | nephritis, hereditary | 1.52297514824283 |
| P35637 | - | D018208 | liposarcoma, myxoid | 2.33535001366238 |
| P35637 | - | D007950 | leukemia, myelocytic, acute | 4.54951692806327 |
| P35638 | 126337 | D018208 | liposarcoma, myxoid | exact |
| P35658 | - | D007950 | leukemia, myelocytic, acute | 4.54951692806327 |
| P35659 | - | D007950 | leukemia, myelocytic, acute | 4.54951692806327 |
| P35670 | 277900 | D006527 | hepatolenticular degeneration | exact |
| P35680 | 606391 | D003924 | diabetes mellitus, type 2 | exact |
| P35680 | 137920 | D003924 | diabetes mellitus, type 2 | -1.51516829116293 |
| P35680 | 125853 | D003924 | diabetes mellitus, type 2 | exact |
| P35749 | 132900 | D017545 | aortic aneurysm, thoracic | 3.2228971726663 |
| P35908 | 146800 | D053560 | ichthyosis bullosa of siemens | exact |
| P35913 | 268000 | D012174 | retinitis pigmentosa | exact |
| P35913 | 163500 | D014786 | vision disorders | exact |
| P35916 | 153100 | D008209 | lymphedema | exact |
| P35916 | - | D006391 | hemangioma | 1.07271521976078 |
| P36021 | 300523 | D038901 | mental retardation, x-linked | 4.95515654897059 |
| P36551 | 121300 | D046349 | coproporphyria, hereditary | exact |
| P36894 | 174900 | D011125 / D044483 | adenomatous polyposis coli / intestinal polyposis | -0.187508902524831 / 4.44180253794663 |
| P36894 | 158350 | D006223 | hamartoma syndrome, multiple | exact |
| P36897 | 609192 130050 | D004535 | ehlers-danlos syndrome | 2.72443890055313 |
| P36897 | 610168 154700 154705 | D008382 | marfan syndrome | exact |
| P36897 | 610380 | D017545 | aortic aneurysm, thoracic | 3.19413001815157 |
| P36969 | - | D007246 | infertility | exact |
| P37023 | 600376 | D013683 | telangiectasia, hereditary hemorrhagic | 3.44336387701164 |
| P37058 | 264300 | D011545 | pseudohermaphroditism | 3.33285483123766 |
| P37088 | 264350 | D011546 | pseudohypoaldosteronism | exact |
| P37173 | 190182 | D003123 | colorectal neoplasms, hereditary nonpolyposis | 4.29415441597567 |
| P37173 | 133239 | D004938 | esophageal neoplasms | exact |
| P37173 | 154705 | D008382 | marfan syndrome | 0.94093855824043 |
| P37173 | 608967 | D017545 | aortic aneurysm, thoracic | 3.4275974595422 |
| P37231 | 601665 | D009765 | obesity | exact |
| P37231 | - | D003110 | colonic neoplasms | exact |
| P37231 | 604367 | D052496 | lipodystrophy, familial partial | exact |
| P37243 | 188570 274300 | D018382 | thyroid hormone resistance syndrome | exact |
| P37287 | 311770 | D006457 | hemoglobinuria, paroxysmal | exact |
| P37840 | 168601 168600 | D020734 / D010300 | parkinsonian disorders / parkinson disease | 3.09498521817485 / exact |
| P37840 | 605543 168600 | D010300 | parkinson disease | exact |
| P37840 | 127750 | D020961 | lewy body disease | exact |
| P38398 | 113705 114480 | D001943 | breast neoplasms | exact |
| P38398 | 113705 | D001943 | breast neoplasms | 1.01563576986493 |
| P38398 | 113705 | D010051 / D001943 | ovarian neoplasms / breast neoplasms | exact / 1.01563576986493 |
| P38435 | 277450 | D020147 | coagulation protein disorders | -0.201005726723153 |
| P38484 | 209950 | D012480 | salmonella infections | -1.53648303645513 |
| P38571 | 278000 | D015223 | wolman disease | exact |
| P38571 | 278000 | D015217 / D015223 | cholesterol ester storage disease / wolman disease | exact / exact |
| P38935 | 604320 | D009134 | muscular atrophy, spinal | 3.71127338951879 |
| P39019 | 105650 | D029503 | anemia, diamond-blackfan | exact |
| P39060 | 267750 | D004677 | encephalocele | 0.873131381174823 |
| P39210 | 256810 | D001177 | arthropathy, neurogenic | 0.499638180447614 |
| P39905 | 142623 | D006627 | hirschsprung disease | exact |
| P39905 | 209880 | D020919 | sleep disorders, intrinsic | 2.37837563432222 |
| P40189 | - | D001172 | arthritis, rheumatoid | exact |
| P40225 | 187950 | D013920 | thrombocythemia, hemorrhagic | exact |
| P40337 | 193300 | D006623 | hippel-lindau disease | exact |
| P40424 | - | D015452 | leukemia, pre-b-cell | 2.9366553525661 |
| P40692 | 609310 | D003123 | colorectal neoplasms, hereditary nonpolyposis | 4.67425558852263 |
| P40692 | 276300 | D016543 | central nervous system neoplasms | -1.50685036263698 |
| P40692 | - | D002278 | carcinoma in situ | 3.7212645628076 |
| P40692 | 608089 | D016889 | endometrial neoplasms | exact |
| P40855 | 214100 | D015211 | zellweger syndrome | exact |
| P40939 | 609015 | D011488 | protein deficiency | 0.0504004844431911 |
| P40939 | 609016 | D005234 | fatty liver | -1.97668028719588 |
| P41159 | 601665 | D009765 | obesity | exact |
| P41180 | 145980 | D006934 | hypercalcemia | -1.38110407215883 |
| P41180 | 239200 | D049950 | hyperparathyroidism, primary | 0.912966230455462 |
| P41180 | 146200 | D007011 / D006996 | hypoparathyroidism / hypocalcemia | -0.754315561659118 / 2.36904612981025 |
| P41181 | 125800 | D018500 | diabetes insipidus, nephrogenic | exact |
| P41182 | - | D008228 | lymphoma, non-hodgkin | 2.88880845735543 |
| P41182 | - | D015448 | leukemia, b-cell | 4.22801841567191 |
| P41182 | - | D008223 | lymphoma | exact |
| P41212 | - | D015477 | leukemia, myelomonocytic, chronic | 5.57615825638577 |
| P41212 | - | D007950 | leukemia, myelocytic, acute | 1.23080265610631 |
| P41212 | - | D015454 | leukemia, lymphocytic, acute, l1 | exact |
| P41212 | - | D015452 | leukemia, pre-b-cell | -0.975117816370973 |
| P41212 | - | D009190 | myelodysplastic syndromes | 0.692357449466273 |
| P41212 | - | D009190 | myelodysplastic syndromes | exact |
| P41212 | - | D007950 | leukemia, myelocytic, acute | 4.14556035978551 |
| P41212 | - | D015472 | leukemia, eosinophilic, acute | exact |
| P41212 | 601626 | D007950 | leukemia, myelocytic, acute | exact |
| P41225 | 300123 | D038901 | mental retardation, x-linked | -0.469784696783234 |
| P41229 | 300534 | D038901 | mental retardation, x-linked | exact |
| P41235 | 125850 606391 | D003924 | diabetes mellitus, type 2 | exact |
| P41250 | 601472 | D002607 | charcot-marie-tooth disease | 3.11607104245012 |
| P41250 | 600794 | D009134 | muscular atrophy, spinal | 3.48717337285744 |
| P41732 | 300210 | D038901 | mental retardation, x-linked | 4.09955959951063 |
| P42224 | 209950 | D012480 | salmonella infections | -1.53648303645513 |
| P42336 | 114500 | D015179 | colorectal neoplasms | exact |
| P42336 | 114480 | D001943 | breast neoplasms | exact |
| P42336 | 604370 | D010051 | ovarian neoplasms | 2.5490583355412 |
| P42566 | - | D015447 | leukemia, lymphocytic, acute | 3.51758699420083 |
| P42568 | - | D015447 | leukemia, lymphocytic, acute | 3.51758699420083 |
| P42701 | 209950 | D012480 | salmonella infections | -1.53648303645513 |
| P42702 | 601559 | D010009 | osteochondrodysplasias | 6.25701447751363 |
| P42704 | 220111 | D030401 | cytochrome-c oxidase deficiency | 0.717556212367878 |
| P42768 | 301000 | D014923 | wiskott-aldrich syndrome | exact |
| P42768 | 313900 | D013921 | thrombocytopenia | 2.09983617241818 |
| P42771 | - | D009369 | neoplasms | -1.64725631865348 |
| P42771 | 155601 | D008545 | melanoma | 0.770112458105003 |
| P42771 | 606719 | D004416 | dysplastic nevus syndrome | -1.26876130210574 |
| P42771 | 151623 | D016864 | li-fraumeni syndrome | exact |
| P42772 | - | D009369 | neoplasms | -1.64725631865348 |
| P42773 | - | D009369 | neoplasms | -1.64725631865348 |
| P42858 | 143100 | D006816 | huntington disease | exact |
| P42892 | 142623 | D006627 | hirschsprung disease | exact |
| P42898 | 601367 | D020521 | cerebrovascular accident | exact |
| P42898 | 601634 | D009436 | neural tube defects | 0.874749199640761 |
| P43003 | 600111 | D020754 | spinocerebellar ataxias | -1.7930892189747 |
| P43026 | 201250 | D004392 | dwarfism | -1.17119609872354 |
| P43026 | 228900 201250 200700 | D004392 | dwarfism | -1.17119609872354 |
| P43026 | 610017 | D013580 | synostosis | -2.23790619824416 |
| P43080 | 602093 | D012174 | retinitis pigmentosa | -0.0667981925157767 |
| P43246 | 120435 | D003123 | colorectal neoplasms, hereditary nonpolyposis | exact |
| P43246 | 608089 | D016889 | endometrial neoplasms | exact |
| P43251 | 253260 | D028921 | biotinidase deficiency | exact |
| P43320 | - | D002386 | cataract | -1.05521199484682 |
| P43403 | 176947 | D012749 | sexually transmitted diseases | exact |
| P43681 | 600513 | D017034 | epilepsy, frontal lobe | 1.33162290386523 |
| P43694 | 607941 | D006344 | heart septal defects, atrial | 5.38308483756211 |
| P43699 | 118700 | D020822 | choreatic disorders | exact |
| P43897 | 610505 | D028361 | mitochondrial diseases | 2.89672130524771 |
| P45379 | 115195 192600 | D024741 | cardiomyopathy, hypertrophic, familial | exact |
| P45379 | 601494 115200 | D002311 | cardiomyopathy, dilated | exact |
| P45381 | 271900 | D017825 | canavan disease | exact |
| P46019 | 306000 | D005953 / D006015 | glycogen storage disease type i / glycogen storage disease type viii | 1.49157005248487 / 8.74647815996769 |
| P46020 | 300559 | D005953 / D006012 | glycogen storage disease type i / glycogen storage disease type v | 1.49157005248487 / 4.50719527184942 |
| P46100 | 301040 | D038901 | mental retardation, x-linked | 2.77633894534451 |
| P46100 | 309580 | D038901 | mental retardation, x-linked | 0.882659042483826 |
| P46100 | 300448 | D017085 | alpha-thalassemia | 2.120369653175 |
| P46527 | 610755 | D018761 | multiple endocrine neoplasia type 1 | 4.36614609447918 |
| P46531 | - | D015459 | leukemia, t-cell, acute | exact |
| P46734 | - | D003110 | colonic neoplasms | exact |
| P46736 | - | D015458 | leukemia, t-cell | -0.0964129580800105 |
| P47804 | 268000 | D012174 | retinitis pigmentosa | exact |
| P47871 | - | D003924 | diabetes mellitus, type 2 | 4.92422050243386 |
| P48023 | 601859 | D016884 | polyendocrinopathies, autoimmune | -2.00376642617331 |
| P48029 | 300352 | D053632 / D038901 | x-linked combined immunodeficiency diseases / mental retardation, x-linked | -1.55144439237832 / 0.392776610961138 |
| P48048 | 241200 | D001477 | bartter syndrome | 1.01183364613939 |
| P48382 | 209920 | D016511 | severe combined immunodeficiency | exact |
| P48431 | 206900 | D004933 | esophageal atresia | 1.37001898213833 |
| P48506 | 230450 | D000743 | anemia, hemolytic | exact |
| P48553 | - | D016884 | polyendocrinopathies, autoimmune | 0.571157894882422 |
| P48643 | 256840 | D015419 | spastic paraplegia, hereditary | 3.94368600873597 |
| P48728 | 605899 | D020158 | hyperglycinemia, nonketotic | exact |
| P48730 | 604348 | D020178 | sleep disorders, circadian rhythm | 7.040678663151 |
| P49257 | 227300 | D006467 | hemophilia a | 3.0791740643296 |
| P49281 | 206100 | D000747 | anemia, hypochromic | 3.10428877098111 |
| P49411 | 610678 | D028361 | mitochondrial diseases | 2.69202101837181 |
| P49418 | - | D016750 | stiff-person syndrome | exact |
| P49638 | 277460 | D014811 | vitamin e deficiency | -0.232953263492941 |
| P49639 | 601536 | D020295 | brain stem neoplasms | -2.36316350734366 |
| P49639 | 601536 | D020295 | brain stem neoplasms | -2.36316350734366 |
| P49675 | 201710 | D000312 | adrenal hyperplasia, congenital | 4.08438904594334 |
| P49747 | 132400 | D010009 | osteochondrodysplasias | 5.82696388052472 |
| P49747 | 177170 | D010009 | osteochondrodysplasias | 2.24636428493824 |
| P49768 | 607822 | D000544 | alzheimer disease | 5.60490800279944 |
| P49768 | 600274 | D003704 | dementia | exact |
| P49770 | 603896 | D002493 | central nervous system diseases | -2.27852450745258 |
| P49770 | 603896 | D002493 | central nervous system diseases | -2.27852450745258 |
| P49789 | 144700 | D002292 | carcinoma, renal cell | exact |
| P49789 | - | D004067 | digestive system neoplasms | 0.318994966376653 |
| P49798 | 604906 181500 | D012559 | schizophrenia | exact |
| P49810 | 606889 104300 | D000544 | alzheimer disease | exact |
| P49815 | 191100 | D014402 | tuberous sclerosis | exact |
| P49815 | 606690 | D018192 | lymphangioleiomyomatosis | exact |
| P49821 | 203450 | D038261 | alexander disease | exact |
| P49821 | 256000 | D007888 | leigh disease | exact |
| P49821 | 252010 | D028361 | mitochondrial diseases | 3.48654516268259 |
| P49915 | - | D007950 | leukemia, myelocytic, acute | 4.54951692806327 |
| P49918 | 130650 | D001506 | beckwith-wiedemann syndrome | exact |
| P49918 | - | D009369 | neoplasms | -1.64725631865348 |
| P50336 | 176200 | D011164 / D046350 | porphyrias / porphyria, variegate | -2.13339327034745 / exact |
| P50402 | 310300 | D020389 | muscular dystrophy, emery-dreifuss | exact |
| P50443 | 226900 | D010009 | osteochondrodysplasias | 5.34971508454136 |
| P50461 | 607482 | D002311 | cardiomyopathy, dilated | 1.92947071162984 |
| P50539 | 176807 | D011471 | prostatic neoplasms | exact |
| P50542 | 202370 | D018901 | peroxisomal disorders | exact |
| P50542 | 214100 | D015211 | zellweger syndrome | exact |
| P50542 | 266510 | D052919 | refsum disease, infantile | exact |
| P50549 | 133450 | D012512 | sarcoma, ewing's | exact |
| P50570 | 160150 | D020914 | myopathies, structural, congenital | 2.22167916865264 |
| P50570 | 606482 | D002607 | charcot-marie-tooth disease | 0.810355800912453 |
| P50747 | 253270 | D028922 | holocarboxylase synthetase deficiency | exact |
| P50897 | 256730 | D009472 | neuronal ceroid-lipofuscinoses | exact |
| P50897 | - | D009472 | neuronal ceroid-lipofuscinoses | 6.42119412750834 |
| P50993 | 602481 | D020325 | migraine with aura | 6.53759305543464 |
| P51149 | 600882 | D002607 / D015417 | charcot-marie-tooth disease / hereditary motor and sensory neuropathies | 3.52283314101445 / 5.0714225064032 |
| P51159 | 607624 | D016116 | piebaldism | 4.09061901009908 |
| P51168 | 264350 | D011546 | pseudohypoaldosteronism | exact |
| P51460 | 219050 | D003456 | cryptorchidism | exact |
| P51508 | 300498 | D038901 | mental retardation, x-linked | 4.39664496494468 |
| P51508 | - | D008607 | mental retardation | 0.189704038823044 |
| P51570 | 230200 | D005693 | galactosemias | 8.75740531078439 |
| P51575 | 600845 | D006470 | hemorrhage | 2.58119566437845 |
| P51587 | 600185 114480 | D001943 | breast neoplasms | exact |
| P51587 | 605724 227650 | D005199 | fanconi anemia | exact |
| P51608 | 105830 | D017204 | angelman syndrome | exact |
| P51608 | 300055 | D038901 | mental retardation, x-linked | -2.1764271588347 |
| P51608 | 300279 | D038901 | mental retardation, x-linked | 2.41609215145576 |
| P51608 | 300458 | D038901 | mental retardation, x-linked | 4.62601324261053 |
| P51608 | 312750 | D015518 | rett syndrome | exact |
| P51648 | 270200 | D016111 | sjogren-larsson syndrome | exact |
| P51654 | 312870 | D053632 | x-linked combined immunodeficiency diseases | -2.08128852608049 |
| P51688 | 252900 | D009084 | mucopolysaccharidosis iii | 4.86621311001913 |
| P51690 | 302950 | D002806 | chondrodysplasia punctata | 2.30626959071507 |
| P51692 | 245590 | D046150 | laron syndrome | 4.60009940047089 |
| P51787 | 192500 | D029597 | romano-ward syndrome | exact |
| P51787 | 220400 | D029593 | jervell-lange nielsen syndrome | exact |
| P51787 | 607554 | D001281 | atrial fibrillation | exact |
| P51788 | 607628 | D004830 | epilepsy, tonic-clonic | -0.186055390327741 |
| P51788 | 607682 | D004832 | epilepsy, absence | 5.06555191555434 |
| P51788 | 607631 | D004832 | epilepsy, absence | exact |
| P51795 | 300554 | D053098 | hypophosphatemic rickets, x-linked dominant | 7.14254603346765 |
| P51795 | 300009 | D053040 | nephrolithiasis | 2.80126756508455 |
| P51795 | 310468 | D053040 | nephrolithiasis | 2.95978221282182 |
| P51798 | 259700 | D010022 | osteopetrosis | 4.0658825110142 |
| P51798 | 166600 | D010022 | osteopetrosis | 5.38016857784297 |
| P51801 | 607364 | D001477 | bartter syndrome | 2.30429328198195 |
| P51810 | 300500 | D016117 | albinism, ocular | 1.84862726091306 |
| P51811 | 314850 | D020822 | choreatic disorders | -2.11971534000141 |
| P51812 | 303600 | D038921 | coffin-lowry syndrome | exact |
| P51814 | 314995 | D038901 | mental retardation, x-linked | 3.83517398053316 |
| P51814 | - | D008607 | mental retardation | -2.13048865370961 |
| P51816 | 309548 | D005600 | fragile x syndrome | 9.27612440527424 |
| P51817 | - | D012735 | sexual dysfunction, physiological | -2.34162926833566 |
| P51825 | - | D015447 | leukemia, lymphocytic, acute | 3.51758699420083 |
| P51843 | 300200 | D000224 | addison disease | 0.544800291026387 |
| P51854 | - | D020915 | korsakoff syndrome | 0.103370064474327 |
| P51861 | - | D020362 | paraneoplastic cerebellar degeneration | exact |
| P52701 | 600678 | D003123 | colorectal neoplasms, hereditary nonpolyposis | 4.38495531168387 |
| P52701 | 608089 | D016889 | endometrial neoplasms | exact |
| P52788 | 309583 | D038901 | mental retardation, x-linked | -0.410903267699446 |
| P52926 | - | D008067 | lipoma | -1.29248125036058 |
| P52926 | - | D007889 | leiomyoma | 1.09293327265567 |
| P52945 | 125853 | D003924 | diabetes mellitus, type 2 | exact |
| P52945 | 606392 606391 | D003924 | diabetes mellitus, type 2 | exact |
| P52948 | - | D007950 | leukemia, myelocytic, acute | 1.23080265610631 |
| P52948 | - | D015454 | leukemia, lymphocytic, acute, l1 | 1.25618615935482 |
| P52948 | - | D009190 | myelodysplastic syndromes | -1.94047015761774 |
| P52952 | 108900 | D006344 | heart septal defects, atrial | exact |
| P52952 | 187500 | D013771 | tetralogy of fallot | exact |
| P53420 | 203780 | D009394 | nephritis, hereditary | exact |
| P53420 | 141200 | D006417 | hematuria | -1.10441240597293 |
| P53634 | 245000 | D010214 | papillon-lefevre disease | exact |
| P53634 | 170650 | D010520 | periodontitis, juvenile | exact |
| P53673 | - | D002386 | cataract | -1.05521199484682 |
| P53675 | - | D004062 | digeorge syndrome | 3.89246449853428 |
| P53779 | 606369 | D020264 | lead poisoning, nervous system, childhood | 4.8391397403976 |
| P54098 | 157640 | D017246 | ophthalmoplegia, chronic progressive external | 5.12582906657871 |
| P54098 | 258450 | D017246 | ophthalmoplegia, chronic progressive external | 3.40127648784145 |
| P54098 | 607459 | D020754 | spinocerebellar ataxias | -1.08808755926671 |
| P54098 | 203700 | D002549 | diffuse cerebral sclerosis of schilder | exact |
| P54098 | 603041 | D007418 | intestinal pseudo-obstruction | -0.226811791615573 |
| P54132 | 210900 | D001816 | bloom syndrome | exact |
| P54252 | 109150 | D017827 | machado-joseph disease | exact |
| P54253 | 164400 | D020754 | spinocerebellar ataxias | exact |
| P54259 | 125370 | D020191 | myoclonic epilepsies, progressive | exact |
| P54277 | 600258 | D003123 | colorectal neoplasms, hereditary nonpolyposis | 4.57951151284273 |
| P54278 | 600259 | D003123 | colorectal neoplasms, hereditary nonpolyposis | 4.40892794044614 |
| P54278 | 276300 | D016543 | central nervous system neoplasms | -1.50685036263698 |
| P54278 | 608623 | D019080 | cafe-au-lait spots | 0.902475267967624 |
| P54577 | 608323 | D002607 | charcot-marie-tooth disease | 0.6543925768228 |
| P54802 | 252920 | D009084 | mucopolysaccharidosis iii | 3.69787580687739 |
| P54803 | 245200 | D007965 | leukodystrophy, globoid cell | exact |
| P54840 | 240600 | D005953 / D006008 | glycogen storage disease type i / glycogen storage disease | 2.50044887635656 / 3.72308198843234 |
| P54845 | 162080 | D012174 | retinitis pigmentosa | 2.37842132684244 |
| P55000 | 248300 | D007645 | keratoderma, palmoplantar | exact |
| P55017 | 263800 | D053579 | gitelman syndrome | exact |
| P55072 | 167320 | D000690 | amyotrophic lateral sclerosis | 4.80911856975267 |
| P55084 | 609015 | D011488 | protein deficiency | 0.0504004844431911 |
| P55157 | 200100 | D000012 | abetalipoproteinemia | exact |
| P55196 | - | D015447 | leukemia, lymphocytic, acute | 3.51758699420083 |
| P55197 | - | D015447 | leukemia, lymphocytic, acute | 3.51758699420083 |
| P55197 | - | D016403 | lymphoma, large-cell, diffuse | exact |
| P55198 | - | D015447 | leukemia, lymphocytic, acute | 3.51758699420083 |
| P55199 | - | D015447 | leukemia, lymphocytic, acute | 3.51758699420083 |
| P55268 | 609049 | D009404 | nephrotic syndrome | -1.44340601618626 |
| P55268 | 609049 | D009404 | nephrotic syndrome | 3.63980756393754 |
| P55809 | 245050 | D016883 | diabetic ketoacidosis | 3.62449086490779 |
| P55895 | 601457 | D016511 | severe combined immunodeficiency | 0.266668263748314 |
| P55895 | 603554 | D016511 | severe combined immunodeficiency | 3.43786019655994 |
| P55916 | 601665 | D009765 | obesity | exact |
| P56277 | - | D015458 | leukemia, t-cell | 3.22376941472451 |
| P56278 | - | D015458 | leukemia, t-cell | 2.32752878954422 |
| P56279 | - | D015461 | leukemia, t-cell, chronic | exact |
| P56377 | 300630 | D038901 | mental retardation, x-linked | 4.09955959951063 |
| P56539 | 607801 | D049288 | muscular dystrophies, limb-girdle | 3.23836324541289 |
| P56539 | 192600 | D024741 | cardiomyopathy, hypertrophic, familial | exact |
| P56589 | 214100 | D015211 | zellweger syndrome | exact |
| P56693 | 277580 | D014849 | waardenburg's syndrome | 2.47230745042781 |
| P56715 | 180100 | D012174 | retinitis pigmentosa | 4.85244291556671 |
| P56730 | 249500 | D008607 | mental retardation | -1.11663713475205 |
| P56846 | - | D015458 | leukemia, t-cell | -2.49515714737337 |
| P56847 | - | D015458 | leukemia, t-cell | -2.49515714737337 |
| P57082 | 147891 | D009261 | nail-patella syndrome | -0.491262519327753 |
| P57679 | 225500 | D004613 | ellis-van creveld syndrome | exact |
| P58012 | 110100 | D004370 | duane retraction syndrome | 4.65900947987718 |
| P58012 | 608996 | D016649 | ovarian failure, premature | 5.85251187651967 |
| P58304 | 610093 | D000853 | anophthalmos | -2.24269570156454 |
| P58340 | - | D009190 | myelodysplastic syndromes | exact |
| P58418 | 276902 | D052245 | usher syndromes | exact |
| P59103 | - | D012559 | schizophrenia | exact |
| P60201 | 312080 | D020371 | pelizaeus-merzbacher disease | exact |
| P60201 | 312920 | D010264 / D015419 | paraplegia / spastic paraplegia, hereditary | 1.6853312629306 / 4.49225696825352 |
| P60484 | 158350 | D006223 | hamartoma syndrome, multiple | exact |
| P60484 | 158350 | D006223 | hamartoma syndrome, multiple | exact |
| P60484 | 275355 | D002294 | carcinoma, squamous cell | 1.29152651980015 |
| P60484 | 608089 | D016889 | endometrial neoplasms | exact |
| P60484 | 176920 | D016715 | proteus syndrome | exact |
| P60484 | 137800 | D009837 / D005909 | oligodendroglioma / glioblastoma | exact / exact |
| P60484 | 176807 | D011471 | prostatic neoplasms | exact |
| P60484 | 605309 | D001321 | autistic disorder | -2.1319535785852 |
| P60568 | - | D015459 | leukemia, t-cell, acute | 6.11107035394932 |
| P61457 | 264070 | D010661 | phenylketonurias | -1.3684827970831 |
| P61626 | 105200 | D000686 / D028226 | amyloidosis / amyloidosis, familial | -0.939852883141143 / 3.57457139122741 |
| P61769 | 241600 | D011504 | protein-losing enteropathies | 9.9149024470324 |
| P61916 | 607625 | D052536 | niemann-pick disease, type a | 3.07902532474099 |
| P62070 | - | D010049 | ovarian diseases | -1.46526348104697 |
| P62304 | - | D008180 | lupus erythematosus, systemic | 2.34614781326417 |
| P62324 | - | D015451 | leukemia, b-cell, chronic | 6.06538328112731 |
| P62633 | 602668 | D009223 / D020967 | myotonic dystrophy / myotonic disorders | 4.37521896176149 / exact |
| P62847 | 610629 | D029503 | anemia, diamond-blackfan | 7.43776767698283 |
| P63092 | 103580 | D011556 | pseudopseudohypoparathyroidism | exact |
| P63092 | 103580 | D011547 / D011556 | pseudohypoparathyroidism / pseudopseudohypoparathyroidism | -2.12728869883478 / exact |
| P63092 | 174800 | D005359 | fibrous dysplasia, polyostotic | exact |
| P63092 | 102200 | D049912 / D000172 | growth hormone-secreting pituitary adenoma / acromegaly | 5.18853781841886 / exact |
| P63092 | 166350 | D009999 | ossification, heterotopic | 5.63325251855396 |
| P63092 | 219080 | D003480 | cushing syndrome | -1.87245777468612 |
| P63092 | 603233 | D011547 | pseudohypoparathyroidism | -1.79395536550144 |
| P63252 | 170390 | D050030 | andersen syndrome | exact |
| P68032 | 115200 | D002311 | cardiomyopathy, dilated | exact |
| P68032 | 192600 | D024741 | cardiomyopathy, hypertrophic, familial | exact |
| P68133 | 161800 | D017696 | myopathies, nemaline | 4.58287002991493 |
| P68133 | 102610 | D020512 | myopathy, central core | -2.36764381056662 |
| P68133 | 255310 | D020914 | myopathies, structural, congenital | 6.58399353259738 |
| P68871 | 141900 | D017086 | beta-thalassemia | exact |
| P68871 | 603903 | D000755 | anemia, sickle cell | exact |
| P68871 | 603902 | D017086 / D000742 | beta-thalassemia / anemia, dyserythropoietic, congenital | -2.28603916711621 / -1.97659954245064 |
| P69905 | 141800 | D017085 | alpha-thalassemia | exact |
| P69905 | 236750 | D015160 | hydrops fetalis | 5.54846010764246 |
| P78314 | 118400 | D002636 | cherubism | exact |
| P78332 | - | D002289 | carcinoma, non-small-cell lung | 2.24284003207922 |
| P78363 | 248200 | D008268 | macular degeneration | 3.73428050436983 |
| P78363 | 248200 | D008268 | macular degeneration | 3.73428050436983 |
| P78363 | 153800 | D008268 | macular degeneration | 6.46472276679181 |
| P78363 | 604116 | D012174 | retinitis pigmentosa | 5.91898098281027 |
| P78363 | 601718 | D012174 | retinitis pigmentosa | 3.16816593006434 |
| P78380 | 608557 | D009203 | myocardial infarction | exact |
| P78380 | 104300 | D000544 | alzheimer disease | exact |
| P78424 | 601583 | D009396 | wilms tumor | 4.09885723610175 |
| P78504 | 118450 | D016738 | alagille syndrome | exact |
| P78504 | 187500 | D013771 | tetralogy of fallot | exact |
| P78509 | - | D012559 | schizophrenia | exact |
| P78509 | - | D001321 | autistic disorder | exact |
| P78562 | 307800 | D053098 | hypophosphatemic rickets, x-linked dominant | exact |
| P80365 | 218030 | D043204 | mineralocorticoid excess syndrome, apparent | 12.1990641554197 |
| P80748 | - | D015432 | glomerulonephritis, membranoproliferative | 5.6343869493719 |
| P81172 | 602390 | D006432 | hemochromatosis | 1.8331313014115 |
| P82251 | 220100 | D003555 | cystinuria | exact |
| P82279 | 600105 | D012174 | retinitis pigmentosa | 3.71673810733109 |
| P84996 | 139320 | D018312 | sex cord-gonadal stromal tumors | -1.5727562070459 |
| P84996 | 219080 | D003480 | cushing syndrome | -1.87245777468612 |
| P84996 | 603233 | D011547 | pseudohypoparathyroidism | -1.79395536550144 |
| P85173 | - | D050197 | atherosclerosis | exact |
| P98160 | 255800 | D010009 | osteochondrodysplasias | exact |
| P98161 | 173900 | D016891 / D007690 | polycystic kidney, autosomal dominant / polycystic kidney diseases | exact / exact |
| P98170 | 300635 | D008232 | lymphoproliferative disorders | 5.65156810513465 |
| P98172 | 304110 | D004413 | dysostoses | -1.44376263537079 |
| P98174 | 305400 | D001289 | attention deficit disorder with hyperactivity | 3.44602100349225 |
| P98174 | - | D038901 | mental retardation, x-linked | 1.61699861109505 |
| P98177 | - | D015447 | leukemia, lymphocytic, acute | 3.51758699420083 |
| P98194 | 169600 | D016506 | pemphigus, benign familial | exact |
| Q00005 | 604326 | D020754 | spinocerebellar ataxias | 2.99563263643882 |
| Q00597 | 227650 | D005199 | fanconi anemia | exact |
| Q00653 | - | D008228 | lymphoma, non-hodgkin | -0.934567581409349 |
| Q00653 | - | D015458 | leukemia, t-cell | 1.46923412330156 |
| Q00LT1 | 610599 | D012174 | retinitis pigmentosa | 2.51676715993538 |
| Q01196 | - | D007950 | leukemia, myelocytic, acute | 4.31348280429288 |
| Q01196 | - | D009190 | myelodysplastic syndromes | -0.24425265708104 |
| Q01196 | - | D015464 | leukemia, myeloid, chronic | exact |
| Q01196 | - | D015454 | leukemia, lymphocytic, acute, l1 | exact |
| Q01196 | 601399 | D007950 | leukemia, myelocytic, acute | -1.00221539907645 |
| Q01453 | 118220 | D002607 / D015417 | charcot-marie-tooth disease / hereditary motor and sensory neuropathies | 3.38023145923698 / 5.05538843666641 |
| Q01453 | 145900 | D015417 | hereditary motor and sensory neuropathies | exact |
| Q01453 | 162500 | D011115 | polyneuropathies | 2.98805767565552 |
| Q01453 | 118300 | D002607 | charcot-marie-tooth disease | 5.35738990895768 |
| Q01453 | 139393 | D020275 | guillain-barre syndrome | exact |
| Q01484 | 600919 | D008133 / D012804 | long qt syndrome / sick sinus syndrome | 0.0266881202422546 / 3.97360902702387 |
| Q01534 | - | D018238 | gonadoblastoma | exact |
| Q01543 | 133450 | D012512 | sarcoma, ewing's | exact |
| Q01831 | 278720 | D014983 | xeroderma pigmentosum | 5.24887593225418 |
| Q01844 | 133450 | D012512 | sarcoma, ewing's | exact |
| Q01844 | - | D018227 | sarcoma, clear cell | exact |
| Q01844 | - | D018228 | sarcoma, small cell | 3.77337865485017 |
| Q01955 | - | D019867 | anti-glomerular basement membrane disease | exact |
| Q01955 | 203780 | D009394 | nephritis, hereditary | exact |
| Q01955 | 141200 | D006417 | hematuria | -1.10441240597293 |
| Q01968 | 309000 | D009800 | oculocerebrorenal syndrome | exact |
| Q01974 | 268310 | D009394 | nephritis, hereditary | -1.85286469841654 |
| Q02078 | 608320 | D003324 | coronary arteriosclerosis | 0.901122714152753 |
| Q02223 | - | D015459 | leukemia, t-cell, acute | 6.11107035394932 |
| Q02318 | 213700 | D019294 | xanthomatosis, cerebrotendinous | exact |
| Q02388 | - | D016107 | epidermolysis bullosa acquisita | exact |
| Q02388 | 131750 226600 | D016108 | epidermolysis bullosa dystrophica | exact |
| Q02388 | 131705 | D016108 | epidermolysis bullosa dystrophica | 4.4935061330659 |
| Q02388 | 131850 | D016108 / D004820 | epidermolysis bullosa dystrophica / epidermolysis bullosa | 2.87112715429007 / 2.39124604278065 |
| Q02388 | 132000 | D016108 | epidermolysis bullosa dystrophica | 2.90152777297908 |
| Q02388 | 604129 | D004820 | epidermolysis bullosa | 1.86292520920693 |
| Q02388 | 607600 | D016108 / D016110 | epidermolysis bullosa dystrophica / epidermolysis bullosa simplex | 1.40076262537993 / 4.39534566650934 |
| Q02413 | 148700 | D007645 | keratoderma, palmoplantar | 0.136446522658431 |
| Q02487 | 610476 | D019571 | arrhythmogenic right ventricular dysplasia | 6.78394946231695 |
| Q02643 | 262400 | D004393 | dwarfism, pituitary | 4.58606284218636 |
| Q02809 | 225400 | D004535 | ehlers-danlos syndrome | 2.23369530706455 |
| Q02846 | 601777 | D012174 | retinitis pigmentosa | 5.49094533750969 |
| Q02952 | - | D009157 | myasthenia gravis | exact |
| Q02962 | 120330 | D009901 | optic nerve diseases | 0.441287886032907 |
| Q03001 | - | D010391 | pemphigoid, bullous | exact |
| Q03111 | - | D015447 | leukemia, lymphocytic, acute | 3.51758699420083 |
| Q03112 | - | D015464 | leukemia, myeloid, chronic | exact |
| Q03164 | - | D015447 | leukemia, lymphocytic, acute | 3.51758699420083 |
| Q03164 | - | D015467 | leukemia, neutrophilic, chronic | exact |
| Q03252 | 608709 | D052496 | lipodystrophy, familial partial | 1.23753755797683 |
| Q03393 | 261640 | D010661 | phenylketonurias | exact |
| Q03395 | 268000 | D012174 | retinitis pigmentosa | exact |
| Q03426 | 260920 | D010505 | familial mediterranean fever | -1.84097743627205 |
| Q03431 | 166000 | D004687 / D018216 | enchondromatosis / osteochondromatosis | exact / exact |
| Q03468 | 133540 | D003057 | cockayne syndrome | exact |
| Q04446 | 232500 | D005953 / D006011 | glycogen storage disease type i / glycogen storage disease type iv | 3.11117646907697 / exact |
| Q04446 | 236750 | D015160 | hydrops fetalis | exact |
| Q04656 | 309400 | D007706 | menkes kinky hair syndrome | exact |
| Q04656 | 304150 | D009261 / D003483 | nail-patella syndrome / cutis laxa | -1.69324239749024 / 2.27970733722688 |
| Q04671 | 203200 | D016115 | albinism, oculocutaneous | 6.05932858013615 |
| Q04671 | - | D012878 | skin neoplasms | 2.040117573801 |
| Q04695 | 167210 | D053549 | pachyonychia congenita | exact |
| Q04695 | 184500 | D004814 | epidermal cyst | 4.64638235447678 |
| Q04721 | 610205 | D016738 | alagille syndrome | 3.94050603461355 |
| Q04771 | 135100 | D009221 | myositis ossificans | exact |
| Q04844 | 254200 | D020720 / D009157 | myasthenia gravis, autoimmune, experimental / myasthenia gravis | 4.69539730248757 / exact |
| Q04844 | 601462 254200 | D020294 | myasthenic syndromes, congenital | exact |
| Q04844 | 608930 | D020294 | myasthenic syndromes, congenital | 0.00627000693277147 |
| Q04844 | 608931 | D020294 | myasthenic syndromes, congenital | 2.43039544390362 |
| Q05066 | 306100 | D006059 / D006061 | gonadal dysgenesis / gonadal dysgenesis, 46,xy | -1.37393276562746 / exact |
| Q05066 | - | D014424 | turner syndrome | exact |
| Q05066 | 235600 | D050090 | hermaphroditism, true | exact |
| Q05084 | - | D003922 | diabetes mellitus, type 1 | exact |
| Q05086 | 105830 | D017204 | angelman syndrome | exact |
| Q05209 | - | D003110 | colonic neoplasms | exact |
| Q05516 | - | D015473 | leukemia, promyelocytic, acute | exact |
| Q05823 | 176807 601518 | D011471 | prostatic neoplasms | exact |
| Q06124 | 151100 | D044542 | leopard syndrome | exact |
| Q06124 | 163950 | D009634 | noonan syndrome | exact |
| Q06124 | 607785 | D015479 | leukemia, myelomonocytic, acute | 0.960874583246403 |
| Q06136 | - | D008224 | lymphoma, follicular | exact |
| Q06141 | - | D050500 | pancreatitis, chronic | exact |
| Q06187 | 300300 | D000361 | agammaglobulinemia | -1.27908393782798 |
| Q06187 | 307200 | D004393 | dwarfism, pituitary | 0.644431157832311 |
| Q06455 | - | D007950 | leukemia, myelocytic, acute | 4.54951692806327 |
| Q06495 | 182309 | D017674 / D010024 | hypophosphatemia / osteoporosis | exact / -0.427074566768273 |
| Q06609 | 114480 | D001943 | breast neoplasms | exact |
| Q06710 | 218700 | D050033 | thyroid dysgenesis | exact |
| Q06787 | 300624 | D005600 | fragile x syndrome | exact |
| Q06787 | 300623 300624 | D005600 | fragile x syndrome | exact |
| Q07001 | 601462 254200 | D020294 | myasthenic syndromes, congenital | exact |
| Q07001 | 608930 | D020294 | myasthenic syndromes, congenital | 0.00627000693277147 |
| Q07699 | 604233 | D003294 | seizures, febrile | 1.46855467728936 |
| Q07837 | 220100 | D003555 | cystinuria | exact |
| Q07889 | 135300 | D005351 | fibromatosis, gingival | 6.01000265145136 |
| Q07889 | 610733 | D009634 | noonan syndrome | 3.09537858124936 |
| Q08499 | 606799 | D020521 | cerebrovascular accident | -1.02969478242549 |
| Q09013 | 160900 | D009223 | myotonic dystrophy | exact |
| Q09428 | 256450 | D046768 | nesidioblastosis | exact |
| Q09428 | 606176 | D003920 | diabetes mellitus | -0.531976883236202 |
| Q09428 | 610374 | D003924 | diabetes mellitus, type 2 | 1.03853974784324 |
| Q09428 | - | D003924 | diabetes mellitus, type 2 | exact |
| Q09470 | 160120 | D020385 | myokymia | exact |
| Q09472 | - | D009369 | neoplasms | -1.96519726625707 |
| Q09472 | - | D007950 | leukemia, myelocytic, acute | 4.54951692806327 |
| Q10469 | 212066 | D018981 | carbohydrate-deficient glycoprotein syndrome | 4.64418988289749 |
| Q10567 | - | D008579 | meningioma | -2.14270110943112 |
| Q10571 | - | D007950 | leukemia, myelocytic, acute | 4.54951692806327 |
| Q10589 | - | D001172 | arthritis, rheumatoid | -1.12685772521912 |
| Q12768 | 603563 | D015419 | spastic paraplegia, hereditary | 3.53776696753425 |
| Q12778 | 268220 | D012208 / D018232 | rhabdomyosarcoma / rhabdomyosarcoma, alveolar | 2.44132152468092 / exact |
| Q12791 | 609446 | D020822 | choreatic disorders | 0.932693714342235 |
| Q12809 | 152427 | D008133 | long qt syndrome | 2.12489750223832 |
| Q12824 | 609322 | D018335 | rhabdoid tumor | exact |
| Q12840 | 604187 | D010264 / D015419 | paraplegia / spastic paraplegia, hereditary | 1.01563858393155 / 3.58374047591397 |
| Q12866 | 268000 | D012174 | retinitis pigmentosa | exact |
| Q12887 | 220110 | D030401 | cytochrome-c oxidase deficiency | exact |
| Q12887 | 256000 | D007888 | leigh disease | exact |
| Q12913 | - | D003110 | colonic neoplasms | -0.369189443187838 |
| Q13015 | - | D015447 | leukemia, lymphocytic, acute | 3.51758699420083 |
| Q13049 | 254110 | D049288 | muscular dystrophies, limb-girdle | 2.85137522959833 |
| Q13075 | 253300 | D014897 | spinal muscular atrophies of childhood | exact |
| Q13117 | - | D009845 | oligospermia | -2.26169437542798 |
| Q13144 | 603896 | D002493 | central nervous system diseases | -2.27852450745258 |
| Q13144 | 603896 | D002493 | central nervous system diseases | -2.27852450745258 |
| Q13144 | 603896 | D002493 | central nervous system diseases | -2.27852450745258 |
| Q13214 | - | D002289 | carcinoma, non-small-cell lung | 2.24284003207922 |
| Q13216 | 216400 | D003057 | cockayne syndrome | exact |
| Q13231 | - | D005776 | gaucher disease | 3.28016102632218 |
| Q13253 | 186500 | D013580 | synostosis | -2.15864887437553 |
| Q13286 | 204200 | D009472 | neuronal ceroid-lipofuscinoses | exact |
| Q13315 | 208900 | D001260 | ataxia telangiectasia | exact |
| Q13315 | - | D015459 | leukemia, t-cell, acute | 2.53870431006288 |
| Q13315 | - | D016393 | lymphoma, b-cell | -0.381327753749312 |
| Q13315 | - | D015451 | leukemia, b-cell, chronic | exact |
| Q13316 | 241520 | D053098 | hypophosphatemic rickets, x-linked dominant | 2.55686927508985 |
| Q13326 | 253700 | D049288 | muscular dystrophies, limb-girdle | 3.018041896265 |
| Q13342 | - | D008105 | liver cirrhosis, biliary | exact |
| Q13402 | 276900 | D052245 | usher syndromes | exact |
| Q13421 | - | D010051 | ovarian neoplasms | 1.44866849063435 |
| Q13428 | 154500 | D008342 | mandibulofacial dysostosis | exact |
| Q13459 | 609753 212750 | D002446 | celiac disease | exact |
| Q13461 | 610256 | D001035 | aphakia | -0.915867640059924 |
| Q13465 | - | D007950 | leukemia, myelocytic, acute | 1.23080265610631 |
| Q13485 | 260350 | D021441 | carcinoma, pancreatic ductal | 3.77617948817023 |
| Q13485 | 174900 | D011125 / D044483 | adenomatous polyposis coli / intestinal polyposis | -0.187508902524831 / 4.44180253794663 |
| Q13485 | 175050 187300 | D013683 | telangiectasia, hereditary hemorrhagic | 3.50676973610655 |
| Q13488 | 259700 | D010022 | osteopetrosis | 4.0658825110142 |
| Q13492 | - | D016403 | lymphoma, large-cell, diffuse | exact |
| Q13495 | 300633 | D007021 | hypospadias | -1.12258884339424 |
| Q13496 | 310400 | D020914 | myopathies, structural, congenital | exact |
| Q13501 | 602080 | D010001 | osteitis deformans | exact |
| Q13515 | 604219 | D002386 | cataract | -1.79112355822367 |
| Q13516 | - | D015459 | leukemia, t-cell, acute | 6.11107035394932 |
| Q13562 | 606394 606391 | D003924 | diabetes mellitus, type 2 | exact |
| Q13563 | 173900 | D016891 / D007690 | polycystic kidney, autosomal dominant / polycystic kidney diseases | exact / exact |
| Q13608 | 214100 | D015211 | zellweger syndrome | exact |
| Q13614 | 601382 | D002607 | charcot-marie-tooth disease | 2.98151188877386 |
| Q13620 | 300354 | D038901 | mental retardation, x-linked | 4.26546537565821 |
| Q13621 | 601678 | D001477 | bartter syndrome | 1.09109097000802 |
| Q13625 | - | D001943 | breast neoplasms | exact |
| Q13627 | - | D004314 | down syndrome | -0.232572461395472 |
| Q13635 | 109400 | D001478 | basal cell nevus syndrome | exact |
| Q13635 | 605462 | D002280 | carcinoma, basal cell | 4.8579950187559 |
| Q13635 | 610828 | D016142 | holoprosencephaly | 0.664474261282568 |
| Q13683 | - | D020914 | myopathies, structural, congenital | -1.12812083301581 |
| Q13698 | 170400 | D020514 | hypokalemic periodic paralysis | exact |
| Q13698 | 601887 | D008305 | malignant hyperthermia | 1.1239893736656 |
| Q13702 | 608931 | D020294 | myasthenic syndromes, congenital | 2.43039544390362 |
| Q13751 | 226700 | D016109 | epidermolysis bullosa, junctional | exact |
| Q13751 | 226650 | D016110 | epidermolysis bullosa simplex | -0.386916265556435 |
| Q13753 | 226700 | D016109 | epidermolysis bullosa, junctional | exact |
| Q13772 | 188550 | D002291 | carcinoma, papillary | 3.4424308069019 |
| Q13835 | 604536 | D004476 | ectodermal dysplasia | -1.77779123994553 |
| Q13873 | 178600 | D006976 | hypertension, pulmonary | 4.00510903830556 |
| Q13873 | 265450 | D011668 | pulmonary veno-occlusive disease | exact |
| Q13936 | 601005 | D008133 | long qt syndrome | 1.06725591404278 |
| Q13950 | 119600 | D002973 | cleidocranial dysplasia | exact |
| Q14003 | 605259 | D020754 | spinocerebellar ataxias | 2.44706045917207 |
| Q14031 | 308940 | D009394 | nephritis, hereditary | -1.53913569738039 |
| Q14050 | 600969 | D010009 | osteochondrodysplasias | 5.60559044313624 |
| Q14055 | 600204 | D010009 | osteochondrodysplasias | 5.74770655665609 |
| Q14055 | 603932 | D007405 | intervertebral disk displacement | 2.11655500100749 |
| Q14126 | 610193 | D019571 | arrhythmogenic right ventricular dysplasia | 6.7861949134016 |
| Q14129 | - | D004062 | digeorge syndrome | 2.25845877406531 |
| Q14145 | - | D002289 | carcinoma, non-small-cell lung | 5.22076260020167 |
| Q14191 | 277700 | D014898 | werner syndrome | exact |
| Q14202 | - | D038901 | mental retardation, x-linked | 3.83517398053316 |
| Q14203 | 607641 | D016472 | motor neuron disease | 6.07975283527454 |
| Q14232 | 603896 | D002493 | central nervous system diseases | -2.27852450745258 |
| Q14376 | 230350 | D005693 | galactosemias | 10.5903196584115 |
| Q14435 | 610233 | D006958 | hyperostosis, cortical, congenital | 0.386936330273464 |
| Q14524 | 113900 | D006327 / D002037 | heart block / bundle-branch block | -1.7713847325591 / exact |
| Q14524 | 603830 | D008133 | long qt syndrome | 1.98278138871847 |
| Q14524 | 601144 | D053840 | brugada syndrome | exact |
| Q14524 | 608567 | D012804 | sick sinus syndrome | 5.45996998611835 |
| Q14524 | 603829 | D014693 | ventricular fibrillation | 4.40581299680596 |
| Q14524 | 272120 | D013398 | sudden infant death | exact |
| Q14524 | 108770 | D001281 | atrial fibrillation | 1.57496928172322 |
| Q14524 | 601154 | D002311 | cardiomyopathy, dilated | 2.26280404496318 |
| Q14626 | - | D011471 | prostatic neoplasms | -1.96919269448012 |
| Q14654 | 601820 | D044903 | persistent hyperinsulinemia hypoglycemia of infancy | exact |
| Q14654 | 606176 | D003920 | diabetes mellitus | -0.531976883236202 |
| Q14654 | 610582 | D003922 / D003924 | diabetes mellitus, type 1 / diabetes mellitus, type 2 | -1.86603654054404 / 1.97473866551974 |
| Q14654 | - | D003924 | diabetes mellitus, type 2 | exact |
| Q14683 | 300590 | D003635 | de lange syndrome | 5.16453743115213 |
| Q14739 | 169400 | D010381 | pelger-huet anomaly | exact |
| Q14896 | 115197 192600 | D024741 | cardiomyopathy, hypertrophic, familial | exact |
| Q15022 | - | D036821 | endometrial stromal tumors | exact |
| Q15049 | 604004 | D015140 | dementia, vascular | -0.212547779991966 |
| Q15049 | - | D002389 | catatonia | 1.12196279144304 |
| Q15051 | 609254 266900 | D015792 | retinal dysplasia | -0.776911957260617 |
| Q15052 | 300436 | D038901 | mental retardation, x-linked | 4.43517398053316 |
| Q15056 | 194050 | D018980 | williams syndrome | exact |
| Q15067 | 264470 | D000326 | adrenoleukodystrophy | -2.06464150847248 |
| Q15075 | - | D008178 | lupus erythematosus, cutaneous | -0.871020112291849 |
| Q15078 | - | D000544 | alzheimer disease | exact |
| Q15116 | 605218 | D008180 | lupus erythematosus, systemic | 4.04146295398168 |
| Q15125 | 302960 | D002806 | chondrodysplasia punctata | exact |
| Q15149 | 226670 | D016110 / D049288 | epidermolysis bullosa simplex / muscular dystrophies, limb-girdle | 3.23613150845635 / 0.900150318690812 |
| Q15149 | 131950 131900 131800 | D016110 / D016108 | epidermolysis bullosa simplex / epidermolysis bullosa dystrophica | 6.64710248323284 / exact |
| Q15154 | 188550 | D002291 | carcinoma, papillary | 3.4424308069019 |
| Q15198 | 114500 | D015179 | colorectal neoplasms | exact |
| Q15223 | 225060 | D004476 | ectodermal dysplasia | 3.25766486678518 |
| Q15233 | - | D002292 | carcinoma, renal cell | exact |
| Q15306 | 254500 | D009101 | multiple myeloma | exact |
| Q15334 | - | D015179 | colorectal neoplasms | -1.31318216930819 |
| Q15465 | 120200 | D003103 | coloboma | 1.3122454324539 |
| Q15465 | 142945 | D016142 | holoprosencephaly | 1.88199075636097 |
| Q15466 | 601665 | D009765 | obesity | exact |
| Q15468 | - | D015459 | leukemia, t-cell, acute | exact |
| Q15526 | 256000 | D007888 | leigh disease | exact |
| Q15532 | - | D013584 | sarcoma, synovial | exact |
| Q15582 | 121820 | D053559 / D003317 | corneal dystrophy, juvenile epithelial of meesmann / corneal dystrophies, hereditary | -1.61570377334201 / -1.43046871670349 |
| Q15582 | 121900 | D003317 | corneal dystrophies, hereditary | 6.4995338869732 |
| Q15582 | 608470 | D003317 | corneal dystrophies, hereditary | 3.14402525812279 |
| Q15582 | 607541 | D003317 | corneal dystrophies, hereditary | 3.34960645743677 |
| Q15583 | 142946 | D016142 | holoprosencephaly | 1.37024003917122 |
| Q15596 | - | D007950 | leukemia, myelocytic, acute | 4.54951692806327 |
| Q15643 | - | D007950 | leukemia, myelocytic, acute | exact |
| Q15669 | - | D008228 | lymphoma, non-hodgkin | 2.26753058843704 |
| Q15672 | 101400 218600 | D000168 | acrocephalosyndactylia | exact |
| Q156A1 | 608768 | D020754 | spinocerebellar ataxias | 3.05611492688467 |
| Q15738 | 308050 | D016113 | ichthyosiform erythroderma, congenital | -0.0224662189625183 |
| Q15788 | - | D012208 | rhabdomyosarcoma | exact |
| Q15796 | - | D015179 | colorectal neoplasms | -0.378828469294248 |
| Q15811 | - | D004314 | down syndrome | exact |
| Q15822 | 610353 | D017034 | epilepsy, frontal lobe | 1.01345703987632 |
| Q15831 | 175200 | D010580 | peutz-jeghers syndrome | exact |
| Q15831 | 273300 | D013733 / D018239 | testicular diseases / seminoma | -0.951256292962171 / exact |
| Q15858 | 133020 | D004916 | erythromelalgia | 3.86904612981025 |
| Q15858 | 243000 | D000699 | pain insensitivity, congenital | 3.69959631062562 |
| Q15858 | 167400 | D009437 | neuralgia | -2.02353623517325 |
| Q16204 | 188550 | D002291 | carcinoma, papillary | 3.4424308069019 |
| Q16222 | - | D007248 | infertility, male | -0.157573568530402 |
| Q16281 | 216900 | D003117 | color vision defects | 3.26003648498857 |
| Q16384 | - | D013584 | sarcoma, synovial | exact |
| Q16385 | - | D013584 | sarcoma, synovial | exact |
| Q16394 | 133700 | D005097 | exostoses, multiple hereditary | exact |
| Q16394 | 215300 | D002813 | chondrosarcoma | exact |
| Q16518 | 180069 | D012174 | retinitis pigmentosa | 3.32552207816942 |
| Q16534 | - | D015452 | leukemia, pre-b-cell | 2.9366553525661 |
| Q16559 | - | D015459 | leukemia, t-cell, acute | exact |
| Q16585 | 604286 | D049288 | muscular dystrophies, limb-girdle | 3.11553564638519 |
| Q16586 | 608099 | D020388 / D049288 | muscular dystrophy, duchenne / muscular dystrophies, limb-girdle | -1.77261278437214 / 3.15260104994126 |
| Q16586 | 608099 | D049288 | muscular dystrophies, limb-girdle | 3.15260104994126 |
| Q16595 | 229300 | D005621 | friedreich ataxia | exact |
| Q16610 | 247100 | D008065 | lipoid proteinosis of urbach and wiethe | exact |
| Q16633 | - | D015448 | leukemia, b-cell | 4.22801841567191 |
| Q16635 | 302060 300069 | D002311 | cardiomyopathy, dilated | 2.26280404496318 |
| Q16637 | 253300 253400 253550 | D009134 / D014897 | muscular atrophy, spinal / spinal muscular atrophies of childhood | exact / exact |
| Q16658 | - | D001943 | breast neoplasms | -1.28899707315699 |
| Q16667 | 114550 | D006528 | carcinoma, hepatocellular | exact |
| Q16671 | 261550 | D011545 | pseudohermaphroditism | 3.83771255551425 |
| Q16678 | 231300 | D006871 | hydrophthalmos | exact |
| Q16787 | 226700 | D016109 | epidermolysis bullosa, junctional | exact |
| Q16821 | 125853 | D003924 | diabetes mellitus, type 2 | exact |
| Q16821 | - | D007333 | insulin resistance | exact |
| Q16849 | - | D003922 | diabetes mellitus, type 1 | exact |
| Q18PE1 | 254300 | D020294 | myasthenic syndromes, congenital | 1.26794313582203 |
| Q29963 | 177900 | D011565 | psoriasis | 3.15329377437644 |
| Q2M3X9 | 300573 | D038901 | mental retardation, x-linked | 3.83517398053316 |
| Q30201 | 235200 | D006432 | hemochromatosis | exact |
| Q30201 | 176200 | D011164 / D046350 | porphyrias / porphyria, variegate | -2.13339327034745 / exact |
| Q32P28 | 610915 | D010013 | osteogenesis imperfecta | 1.66033231543642 |
| Q3SYG4 | 601583 | D009396 | wilms tumor | 4.09885723610175 |
| Q3SYG4 | 209900 | D020788 | bardet-biedl syndrome | exact |
| Q3T906 | 252500 | D009081 | mucolipidoses | exact |
| Q3T906 | 252600 | D009081 | mucolipidoses | exact |
| Q495M9 | 606943 | D052245 | usher syndromes | 0.286605098772388 |
| Q49AH7 | - | D004062 | digeorge syndrome | 2.25845877406531 |
| Q49MI3 | 608380 | D012174 | retinitis pigmentosa | 2.37842132684244 |
| Q4V328 | - | D012713 | serum sickness | -1.64045761331286 |
| Q4VC05 | - | D008228 | lymphoma, non-hodgkin | 2.88880845735543 |
| Q53GD3 | 256550 | D009081 | mucolipidoses | exact |
| Q53GG5 | - | D020391 | muscular dystrophy, facioscapulohumeral | -2.06257065151667 |
| Q58EX7 | 117210 | D020754 | spinocerebellar ataxias | 2.59310082996937 |
| Q58F21 | - | D007248 | infertility, male | exact |
| Q5I7T1 | 152427 | D008133 | long qt syndrome | 2.12489750223832 |
| Q5IJ48 | 268000 | D012174 | retinitis pigmentosa | exact |
| Q5JTC6 | - | D009396 | wilms tumor | exact |
| Q5JVL4 | 254770 606904 | D020190 | myoclonic epilepsy, juvenile | exact |
| Q5JWF2 | 139320 | D018312 | sex cord-gonadal stromal tumors | -1.5727562070459 |
| Q5JWF2 | 219080 | D003480 | cushing syndrome | -1.87245777468612 |
| Q5JWF2 | 603233 | D011547 | pseudohypoparathyroidism | -1.79395536550144 |
| Q5M775 | - | D015479 | leukemia, myelomonocytic, acute | 0.960874583246403 |
| Q5QGS0 | - | D008607 | mental retardation | -1.56583902611344 |
| Q5S007 | 607060 168600 | D010300 | parkinson disease | exact |
| Q5SW96 | 603813 143890 | D006938 | hyperlipoproteinemia type ii | exact |
| Q5T4F4 | 610244 | D010264 / D015419 | paraplegia / spastic paraplegia, hereditary | -0.15668620620242 / 2.64588064380679 |
| Q5TBB1 | 610181 225750 | D004660 | encephalitis | -2.14884027432034 |
| Q5VV43 | 600202 | D004410 | dyslexia | exact |
| Q5VWK5 | 266600 | D003424 | crohn disease | exact |
| Q5VWK5 | 191390 | D003093 | colitis, ulcerative | exact |
| Q5VWQ8 | - | D007950 | leukemia, myelocytic, acute | 1.22401558400151 |
| Q5VXU1 | - | D008223 | lymphoma | exact |
| Q68CP4 | 252930 | D009084 | mucopolysaccharidosis iii | 3.33396161733487 |
| Q693B1 | - | D008527 | medulloblastoma | exact |
| Q695T7 | 234500 | D006250 | hartnup disease | exact |
| Q6FHJ7 | - | D010018 | osteomalacia | -1.03959282117654 |
| Q6IEE7 | 162200 | D009456 | neurofibromatosis 1 | exact |
| Q6IFS5 | 201300 | D009477 / D013595 | hereditary sensory and autonomic neuropathies / syringomyelia | 4.85120411781606 / exact |
| Q6KC79 | 122470 | D003635 | de lange syndrome | exact |
| Q6P1J9 | 145000 | D006961 / D010282 | hyperparathyroidism / parathyroid neoplasms | -2.23962904623455 / 4.81795303089275 |
| Q6P1J9 | 145001 | D006961 | hyperparathyroidism | 2.39155368790546 |
| Q6P1J9 | 608266 | D010282 | parathyroid neoplasms | exact |
| Q6P1N0 | 608443 | D008607 | mental retardation | -1.29373588466284 |
| Q6P2Q9 | 600059 | D012174 | retinitis pigmentosa | 3.16816593006434 |
| Q6Q788 | 145750 | D006953 | hyperlipoproteinemia type iv | exact |
| Q6QNY0 | 203300 | D022861 | hermanski-pudlak syndrome | exact |
| Q6RI45 | - | D015451 | leukemia, b-cell, chronic | 6.06085856639078 |
| Q6U949 | - | D009396 | wilms tumor | 1.8384039982602 |
| Q6UN15 | 607685 | D017681 | hypereosinophilic syndrome | exact |
| Q6VVB1 | 254780 | D020191 / D020192 | myoclonic epilepsies, progressive / lafora disease | 2.76694641728193 / exact |
| Q6W2J9 | 300166 | D006343 | heart septal defects | -2.11537533775557 |
| Q6X4W1 | 146110 | D007006 | hypogonadism | exact |
| Q6XZB0 | 145750 | D006953 | hyperlipoproteinemia type iv | exact |
| Q6ZUT3 | 310700 | D020417 | nystagmus, congenital | 0.48652655327403 |
| Q6ZVD7 | 609404 | D011225 | pre-eclampsia | 4.74379047598929 |
| Q6ZVN8 | 602390 | D006432 | hemochromatosis | 1.8331313014115 |
| Q70J99 | 608898 | D051359 | lymphohistiocytosis, hemophagocytic | 6.47582153993093 |
| Q75V66 | 166260 | D010013 | osteogenesis imperfecta | -2.13521241767359 |
| Q76LX8 | 274150 235400 | D011697 | purpura, thrombotic thrombocytopenic | 7.9060445543531 |
| Q7KZN9 | 220110 | D030401 | cytochrome-c oxidase deficiency | exact |
| Q7KZN9 | 256000 | D007888 | leigh disease | exact |
| Q7LGC8 | 608637 | D010009 | osteochondrodysplasias | -0.00877266611709127 |
| Q7RTP0 | 600363 | D010264 / D015419 | paraplegia / spastic paraplegia, hereditary | 1.11517950411017 / 3.66337321205686 |
| Q7RTS3 | 609069 | D003920 | diabetes mellitus | -2.46629804361048 |
| Q7Z2E3 | 208920 | D013132 | spinocerebellar degenerations | 4.45148738238108 |
| Q7Z333 | 606002 | D020754 | spinocerebellar ataxias | 0.968849510081722 |
| Q7Z333 | 602433 | D000690 | amyotrophic lateral sclerosis | 3.71206456853763 |
| Q7Z403 | 226400 | D004819 | epidermodysplasia verruciformis | exact |
| Q7Z412 | 214100 | D015211 | zellweger syndrome | exact |
| Q7Z412 | 202370 | D018901 | peroxisomal disorders | exact |
| Q7Z412 | 266510 | D052919 | refsum disease, infantile | exact |
| Q7Z4S6 | 135700 | D009886 | ophthalmoplegia | 1.74300610887031 |
| Q86SG3 | - | D009845 | oligospermia | -2.26169437542798 |
| Q86SJ6 | 607903 | D007039 | hypotrichosis | -2.46435853644303 |
| Q86SJ6 | - | D010392 | pemphigus | exact |
| Q86U42 | 164300 | D039141 | muscular dystrophy, oculopharyngeal | exact |
| Q86UK0 | 242500 | D017490 | ichthyosis, lamellar | exact |
| Q86UK0 | 601277 | D017490 | ichthyosis, lamellar | 5.58013042842802 |
| Q86UK5 | 225500 | D004613 | ellis-van creveld syndrome | exact |
| Q86UR5 | - | D012174 | retinitis pigmentosa | 2.89334148436024 |
| Q86V81 | - | D008180 | lupus erythematosus, systemic | 3.17037896497453 |
| Q86VZ6 | - | D036821 | endometrial stromal tumors | exact |
| Q86WC4 | 259700 | D010022 | osteopetrosis | 4.0658825110142 |
| Q86WG3 | 601238 | D002524 | cerebellar ataxia | -0.8167805000405 |
| Q86WG5 | 604563 | D002607 | charcot-marie-tooth disease | 3.07900563889405 |
| Q86XK2 | 193200 | D014820 | vitiligo | exact |
| Q86YC2 | - | D001943 | breast neoplasms | 3.06534051536488 |
| Q86YC2 | 610832 227650 | D005199 | fanconi anemia | exact |
| Q86YV9 | 203300 | D022861 | hermanski-pudlak syndrome | exact |
| Q8IU68 | 226400 | D004819 | epidermodysplasia verruciformis | exact |
| Q8IUD2 | 188550 | D002291 | carcinoma, papillary | 3.4424308069019 |
| Q8IUG5 | - | D008175 | lung neoplasms | exact |
| Q8IVB4 | 143465 | D001289 | attention deficit disorder with hyperactivity | exact |
| Q8IVL0 | - | D009182 | mycosis fungoides | exact |
| Q8IVL0 | - | D012751 | sezary syndrome | exact |
| Q8IWA5 | - | D034381 | hearing loss | 3.21026356677112 |
| Q8IWS0 | 301900 | D008607 | mental retardation | -2.16158751833381 |
| Q8IWY9 | 224120 | D000742 | anemia, dyserythropoietic, congenital | exact |
| Q8IWZ6 | 209900 | D020788 | bardet-biedl syndrome | exact |
| Q8IX94 | - | D016410 | lymphoma, t-cell, cutaneous | 4.88597773623416 |
| Q8IX95 | - | D016410 | lymphoma, t-cell, cutaneous | 4.88597773623416 |
| Q8IXF0 | - | D012559 | schizophrenia | 0.61723262681851 |
| Q8IYD8 | 227650 | D005199 | fanconi anemia | exact |
| Q8IZD9 | 143465 | D001289 | attention deficit disorder with hyperactivity | exact |
| Q8IZP0 | - | D015447 | leukemia, lymphocytic, acute | 3.51758699420083 |
| Q8IZP1 | - | D011471 | prostatic neoplasms | 4.26391978850027 |
| Q8IZU3 | 270960 | D053713 | azoospermia | exact |
| Q8N0W4 | 300495 | D038901 | mental retardation, x-linked | exact |
| Q8N0W4 | 300497 | D020817 | asperger syndrome | -1.1765619731952 |
| Q8N0X7 | 275900 | D010264 / D015419 | paraplegia / spastic paraplegia, hereditary | 3.08392511809605 / 3.21459246584587 |
| Q8N130 | 241530 | D053098 | hypophosphatemic rickets, x-linked dominant | 0.838904329685735 |
| Q8N183 | 252010 | D028361 | mitochondrial diseases | 3.48654516268259 |
| Q8N196 | 610896 | D019280 | branchio-oto-renal syndrome | 4.05705060502442 |
| Q8N302 | - | D007715 | klippel-trenaunay-weber syndrome | 7.77941269850475 |
| Q8N3I7 | 209900 | D020788 | bardet-biedl syndrome | exact |
| Q8N427 | 610852 | D007619 | kartagener syndrome | 5.82758150137435 |
| Q8N4T0 | - | D004370 | duane retraction syndrome | 6.24790843179037 |
| Q8N5I3 | - | D015451 | leukemia, b-cell, chronic | exact |
| Q8N5M1 | 604273 | D028361 | mitochondrial diseases | -1.20977369857287 |
| Q8N608 | - | D001249 | asthma | exact |
| Q8N653 | - | D004062 | digeorge syndrome | 2.25845877406531 |
| Q8N6F8 | 194050 | D018980 | williams syndrome | exact |
| Q8NB91 | 300514 | D005199 | fanconi anemia | -1.73144661652537 |
| Q8NB91 | 227650 | D005199 | fanconi anemia | exact |
| Q8NBK3 | 272200 | D052517 | multiple sulfatase deficiency disease | exact |
| Q8NBP7 | 603776 | D006938 | hyperlipoproteinemia type ii | 5.03966593040012 |
| Q8NBS3 | 217700 | D003317 | corneal dystrophies, hereditary | 1.33293092886006 |
| Q8NCE2 | - | D020914 | myopathies, structural, congenital | 2.21449690175873 |
| Q8NDY4 | - | D009190 | myelodysplastic syndromes | exact |
| Q8NEA6 | 610199 | D003409 | congenital hypothyroidism | -1.99917680765262 |
| Q8NEU8 | 606232 | D002872 | chromosome deletion | -2.00122470656248 |
| Q8NF91 | 610743 | D020754 | spinocerebellar ataxias | -1.33048039866911 |
| Q8NFG4 | - | D002292 | carcinoma, renal cell | exact |
| Q8NFJ6 | 244200 | D017436 / D007006 | kallmann syndrome / hypogonadism | 0.929969557495484 / 3.83124598277809 |
| Q8NFJ9 | 209900 | D020788 | bardet-biedl syndrome | exact |
| Q8NFU7 | - | D015447 | leukemia, lymphocytic, acute | 3.51758699420083 |
| Q8NG31 | - | D007950 | leukemia, myelocytic, acute | exact |
| Q8NI22 | 227300 | D006467 | hemophilia a | 3.0791740643296 |
| Q8NI36 | 609887 | D005902 | glaucoma, open-angle | 3.07612588890091 |
| Q8TA86 | 180104 | D012174 | retinitis pigmentosa | 3.59304005676734 |
| Q8TAM1 | 209900 | D020788 | bardet-biedl syndrome | exact |
| Q8TAM2 | 209900 | D020788 | bardet-biedl syndrome | exact |
| Q8TAX7 | 600807 | D001249 | asthma | exact |
| Q8TB36 | 214400 | D002607 | charcot-marie-tooth disease | 3.07900563889405 |
| Q8TB36 | 607706 | D002607 / D014826 | charcot-marie-tooth disease / vocal cord paralysis | -2.08853491178467 / 1.01974134486748 |
| Q8TB36 | 607831 | D002607 | charcot-marie-tooth disease | 3.11607104245012 |
| Q8TB36 | 608340 | D002607 | charcot-marie-tooth disease | 1.3483257236331 |
| Q8TBA6 | 188550 | D002291 | carcinoma, papillary | 3.4424308069019 |
| Q8TCJ0 | - | D038901 | mental retardation, x-linked | exact |
| Q8TCZ9 | 263200 | D017044 | polycystic kidney, autosomal recessive | exact |
| Q8TDM0 | - | D001943 | breast neoplasms | exact |
| Q8TDN4 | - | D003110 | colonic neoplasms | 2.51416080381216 |
| Q8TDP1 | 610329 225750 | D004660 | encephalitis | -2.14884027432034 |
| Q8TDQ0 | 180300 | D001172 | arthritis, rheumatoid | exact |
| Q8TDY2 | - | D001943 | breast neoplasms | 1.13822061085666 |
| Q8TE73 | 608644 | D007619 | kartagener syndrome | 6.25561714667493 |
| Q8TF17 | 601596 | D002607 | charcot-marie-tooth disease | 2.98151188877386 |
| Q8TF63 | - | D003863 | depression | 0.539863596235366 |
| Q8WTS1 | 275630 242100 | D017490 | ichthyosis, lamellar | 8.18206058101934 |
| Q8WU17 | 144700 | D002292 | carcinoma, renal cell | exact |
| Q8WUF5 | - | D001943 | breast neoplasms | exact |
| Q8WUJ3 | - | D034381 | hearing loss | -0.164217181749801 |
| Q8WVV4 | 300604 | D016649 | ovarian failure, premature | 4.88411037554556 |
| Q8WW38 | 187500 | D013771 | tetralogy of fallot | exact |
| Q8WW38 | 610187 | D006548 | hernia, diaphragmatic | 5.48275268655063 |
| Q8WWY3 | 600138 | D012174 | retinitis pigmentosa | 3.85010049326872 |
| Q8WWZ3 | 224900 | D053360 / D004476 | ectodermal dysplasia, hypohidrotic, autosomal recessive / ectodermal dysplasia | exact / exact |
| Q8WX94 | 231090 | D006828 | hydatidiform mole | exact |
| Q8WXD0 | 219050 | D003456 | cryptorchidism | exact |
| Q8WXF7 | 182600 | D010264 / D015419 | paraplegia / spastic paraplegia, hereditary | 1.54321514941075 / 4.00580172829732 |
| Q8WXG9 | 605472 | D052245 | usher syndromes | 0.123585924627466 |
| Q8WXG9 | 604352 | D003294 | seizures, febrile | 1.33450577464654 |
| Q8WXK8 | - | D010391 | pemphigoid, bullous | exact |
| Q8WXU2 | 127700 | D004410 | dyslexia | exact |
| Q8WYQ5 | - | D004062 | digeorge syndrome | 2.25845877406531 |
| Q8WZ42 | 603689 | D012131 | respiratory insufficiency | -1.50549827094863 |
| Q8WZ42 | 188840 192600 | D024741 | cardiomyopathy, hypertrophic, familial | exact |
| Q8WZ42 | 604145 115200 | D002311 | cardiomyopathy, dilated | exact |
| Q8WZ42 | 600334 | D049310 | distal myopathies | 3.98287511285361 |
| Q8WZ42 | 608807 | D049288 | muscular dystrophies, limb-girdle | 3.18470856293166 |
| Q8WZ55 | 602522 | D001477 | bartter syndrome | 0.613842174024659 |
| Q8WZA1 | 253280 253800 236670 | D050336 / D009136 | mulibrey nanism / muscular dystrophies | -0.530914354979772 / 0.133575041251881 |
| Q8WZA1 | 236670 253800 | D009136 | muscular dystrophies | 0.133575041251881 |
| Q92466 | 278740 | D014983 | xeroderma pigmentosum | 4.72985709753709 |
| Q92481 | 169100 | D004374 | ductus arteriosus, patent | -2.10787879920968 |
| Q92560 | - | D002289 | carcinoma, non-small-cell lung | 2.24284003207922 |
| Q92560 | - | D008527 | medulloblastoma | 3.5851960742088 |
| Q92570 | 133450 | D012512 | sarcoma, ewing's | exact |
| Q92574 | 191100 | D014402 | tuberous sclerosis | exact |
| Q92597 | 601455 | D002607 | charcot-marie-tooth disease | 3.12626259907342 |
| Q92629 | 601287 | D049288 | muscular dystrophies, limb-girdle | 3.11553564638519 |
| Q92629 | 606685 | D002311 | cardiomyopathy, dilated | 1.92947071162984 |
| Q92685 | 601110 | D018981 | carbohydrate-deficient glycoprotein syndrome | 4.29857160186699 |
| Q92698 | - | D009369 | neoplasms | -1.64725631865348 |
| Q92733 | - | D002292 | carcinoma, renal cell | exact |
| Q92734 | 188550 | D002291 | carcinoma, papillary | 3.4424308069019 |
| Q92736 | 600996 | D019571 | arrhythmogenic right ventricular dysplasia | 7.32194905660084 |
| Q92736 | 604772 | D017180 | tachycardia, ventricular | -2.14086407579149 |
| Q92736 | 192605 | D017180 | tachycardia, ventricular | 4.4694170314583 |
| Q92743 | 610149 | D008268 | macular degeneration | -2.01086762301126 |
| Q92765 | 165720 | D010003 | osteoarthritis | exact |
| Q92793 | - | D007950 | leukemia, myelocytic, acute | 4.54951692806327 |
| Q92793 | 180849 | D012415 | rubinstein-taybi syndrome | exact |
| Q92794 | - | D007950 | leukemia, myelocytic, acute | 4.54951692806327 |
| Q92796 | 300189 | D038901 | mental retardation, x-linked | 4.35216648067739 |
| Q92834 | 300389 | D012174 | retinitis pigmentosa | 4.55727833238206 |
| Q92834 | 300029 | D012174 | retinitis pigmentosa | 3.27856789955613 |
| Q92834 | 304020 | D012174 | retinitis pigmentosa | 1.1026802685791 |
| Q92838 | 305100 | D004476 / D053358 | ectodermal dysplasia / ectodermal dysplasia 1, anhidrotic | exact / exact |
| Q92838 | 300606 | D000848 | anodontia | exact |
| Q92887 | 237500 | D007566 | jaundice, chronic idiopathic | exact |
| Q92889 | 278760 | D014983 | xeroderma pigmentosum | 4.42339658427318 |
| Q92902 | 203300 | D022861 | hermanski-pudlak syndrome | exact |
| Q92904 | - | D007246 | infertility | exact |
| Q92915 | 609307 | D020754 | spinocerebellar ataxias | 1.65731585595017 |
| Q92932 | - | D003922 | diabetes mellitus, type 1 | exact |
| Q92949 | 607154 | D006255 | rhinitis, allergic, seasonal | 5.80847188873594 |
| Q92954 | 208250 | D012700 | serositis | -1.88848262658073 |
| Q92968 | 202370 | D018901 | peroxisomal disorders | exact |
| Q92990 | 138000 | D005918 | glomus tumor | 4.39331851700972 |
| Q93015 | - | D002289 | carcinoma, non-small-cell lung | 2.24284003207922 |
| Q93052 | - | D008067 | lipoma | -1.29248125036058 |
| Q93052 | - | D007948 | leukemia, monocytic, acute | exact |
| Q93063 | 133701 | D005097 | exostoses, multiple hereditary | 3.15475049001231 |
| Q93063 | 601224 | D004062 | digeorge syndrome | -0.794376850539633 |
| Q93099 | 203500 | D000474 | alkaptonuria | exact |
| Q93100 | 261750 | D005953 / D006008 | glycogen storage disease type i / glycogen storage disease | 1.49157005248487 / 2.71198345859273 |
| Q969F8 | 146110 | D007006 | hypogonadism | exact |
| Q969F9 | 203300 | D022861 | hermanski-pudlak syndrome | exact |
| Q969G2 | - | D015447 | leukemia, lymphocytic, acute | exact |
| Q969Q4 | 151400 | D015462 | leukemia, lymphocytic, chronic | exact |
| Q969V6 | - | D007947 | leukemia, megakaryocytic, acute | exact |
| Q96A29 | 266265 | D018370 | leukocyte-adhesion deficiency syndrome | 2.37354981001675 |
| Q96AD5 | - | D003924 | diabetes mellitus, type 2 | 3.264874480286 |
| Q96CV9 | 137760 | D005902 | glaucoma, open-angle | 6.78305931894896 |
| Q96CV9 | 606657 | D006850 | hydrocephalus, normal pressure | -0.728154609203746 |
| Q96DA6 | 610198 | D002311 | cardiomyopathy, dilated | 4.30818294095852 |
| Q96DF8 | - | D004062 | digeorge syndrome | 2.25845877406531 |
| Q96DH6 | - | D015464 | leukemia, myeloid, chronic | 2.02691617471708 |
| Q96DT5 | 270100 | D012857 | situs inversus | 3.64605308479807 |
| Q96DT5 | 242650 | D007619 | kartagener syndrome | exact |
| Q96EP1 | - | D002289 | carcinoma, non-small-cell lung | 1.92837967019121 |
| Q96EV8 | 203300 | D022861 | hermanski-pudlak syndrome | exact |
| Q96EW2 | 144700 | D002292 | carcinoma, renal cell | exact |
| Q96F07 | - | D009103 | multiple sclerosis | exact |
| Q96G97 | 269700 | D052497 | lipodystrophy, congenital generalized | exact |
| Q96G97 | 270685 | D010264 | paraplegia | 3.21946687125026 |
| Q96G97 | 600794 | D015419 / D009134 | spastic paraplegia, hereditary / muscular atrophy, spinal | 2.25675020099564 / 3.48717337285744 |
| Q96GD4 | - | D005149 | facial expression | -1.73992062803381 |
| Q96GG9 | - | D002294 | carcinoma, squamous cell | exact |
| Q96GX5 | 188000 | D013921 | thrombocytopenia | 1.94132152468092 |
| Q96HC4 | - | D012559 | schizophrenia | exact |
| Q96HD1 | 606217 600309 | D006345 / D004694 | heart septal defects, ventricular / endocardial cushion defects | 1.24846834848611 / exact |
| Q96I51 | 194050 | D018980 | williams syndrome | exact |
| Q96IS3 | 603075 | D008268 | macular degeneration | 6.54398009066045 |
| Q96IS3 | 610381 | D012174 | retinitis pigmentosa | 5.38859760347527 |
| Q96J92 | 145260 | D011546 | pseudohypoaldosteronism | exact |
| Q96JI7 | 604360 | D010264 / D015419 | paraplegia / spastic paraplegia, hereditary | 1.01283177007574 / 3.52933951490545 |
| Q96JP5 | - | D007950 | leukemia, myelocytic, acute | 1.9876069413948 |
| Q96K21 | - | D007950 | leukemia, myelocytic, acute | exact |
| Q96KN7 | 608194 | D012174 | retinitis pigmentosa | 5.19580227609924 |
| Q96KQ4 | - | D001943 | breast neoplasms | exact |
| Q96L73 | 117550 | D005877 | gigantism | 2.60472668281447 |
| Q96L73 | 130650 | D001506 | beckwith-wiedemann syndrome | exact |
| Q96L73 | - | D015454 | leukemia, lymphocytic, acute, l1 | 1.25618615935482 |
| Q96L73 | - | D009190 | myelodysplastic syndromes | -1.55135652462549 |
| Q96MS0 | 607313 | D017246 | ophthalmoplegia, chronic progressive external | 0.346108463457285 |
| Q96MV8 | 300577 | D038901 | mental retardation, x-linked | 3.83517398053316 |
| Q96P20 | 120100 | D006967 | hypersensitivity | -0.809064682328177 |
| Q96PF2 | - | D004062 | digeorge syndrome | 2.25845877406531 |
| Q96PU8 | - | D012559 | schizophrenia | exact |
| Q96PU8 | - | D009369 | neoplasms | 0.559185677482371 |
| Q96PX8 | 137580 | D005879 | tourette syndrome | exact |
| Q96PZ7 | - | D002294 | carcinoma, squamous cell | 0.658463370162202 |
| Q96Q42 | 205100 | D000690 | amyotrophic lateral sclerosis | 4.03045774622941 |
| Q96Q42 | 606353 | D016472 | motor neuron disease | 5.46924006172507 |
| Q96QS3 | 308350 | D013036 | spasms, infantile | 0.884962179277794 |
| Q96QS3 | 300432 | D038901 | mental retardation, x-linked | 0.501541359207017 |
| Q96QS3 | 309510 | D038901 | mental retardation, x-linked | 4.26546537565821 |
| Q96QS3 | 300419 | D038901 | mental retardation, x-linked | 4.23517398053316 |
| Q96QU1 | 602083 | D052245 | usher syndromes | 0.714228444473642 |
| Q96RD9 | 113970 | D002051 | burkitt lymphoma | exact |
| Q96RK4 | 209900 | D020788 | bardet-biedl syndrome | exact |
| Q96RL7 | 200150 | D020822 | choreatic disorders | exact |
| Q96RP9 | 609060 | D028361 | mitochondrial diseases | 3.07382005515849 |
| Q96RR1 | 609286 | D017246 | ophthalmoplegia, chronic progressive external | 1.26485485827485 |
| Q96RR1 | 607459 | D020754 | spinocerebellar ataxias | -1.08808755926671 |
| Q96RU3 | - | D015447 | leukemia, lymphocytic, acute | 3.51758699420083 |
| Q96RW7 | 603075 | D008268 | macular degeneration | 6.54398009066045 |
| Q96SD1 | 602450 | D016511 | severe combined immunodeficiency | 4.93026678429906 |
| Q96SD1 | 602450 | D016511 | severe combined immunodeficiency | 4.93026678429906 |
| Q96SD1 | 602450 | D016511 | severe combined immunodeficiency | 4.93026678429906 |
| Q96SD1 | 603554 | D016511 | severe combined immunodeficiency | 3.43786019655994 |
| Q96SL1 | 144700 | D002292 | carcinoma, renal cell | exact |
| Q96SW2 | 607417 | D008607 | mental retardation | -1.18004299384696 |
| Q96T37 | - | D007947 | leukemia, megakaryocytic, acute | exact |
| Q96T52 | 137580 | D005879 | tourette syndrome | exact |
| Q99217 | 301200 | D000567 | amelogenesis imperfecta | -0.0460310306957211 |
| Q99250 | 604233 | D003294 | seizures, febrile | 1.46855467728936 |
| Q99259 | 603513 | D002547 | cerebral palsy | 4.09050607619764 |
| Q99424 | - | D015211 | zellweger syndrome | exact |
| Q99453 | 209880 | D020919 | sleep disorders, intrinsic | 2.37837563432222 |
| Q99453 | 256700 | D009447 | neuroblastoma | exact |
| Q99456 | 122100 | D053559 | corneal dystrophy, juvenile epithelial of meesmann | exact |
| Q99459 | - | D021782 | multicystic dysplastic kidney | exact |
| Q99497 | 606324 168600 | D020734 / D010300 | parkinsonian disorders / parkinson disease | -0.758355127214238 / exact |
| Q99502 | 113650 | D019280 | branchio-oto-renal syndrome | 4.12045646411932 |
| Q99519 | 256550 | D009081 | mucolipidoses | exact |
| Q99608 | - | D011218 | prader-willi syndrome | exact |
| Q99643 | 605373 | D010236 / D010235 | paraganglioma, extra-adrenal / paraganglioma | 0.646551438783247 / 1.81484021050518 |
| Q99698 | 214500 | D002609 | chediak-higashi syndrome | exact |
| Q99700 | 183090 | D020754 | spinocerebellar ataxias | exact |
| Q99707 | 601634 | D009436 | neural tube defects | 0.874749199640761 |
| Q99714 | 300220 | D038901 | mental retardation, x-linked | 2.3181813168017 |
| Q99728 | - | D014594 | uterine neoplasms | -2.3616003273765 |
| Q99732 | 601098 | D002607 | charcot-marie-tooth disease | 3.20183323792175 |
| Q99732 | - | D010145 | paget's disease, extramammary | 4.34241243281564 |
| Q99748 | 142623 | D006627 | hirschsprung disease | exact |
| Q99758 | 267450 | D012128 / D006819 | respiratory distress syndrome, adult / hyaline membrane disease | 7.25537239146634 / exact |
| Q99801 | - | D011471 | prostatic neoplasms | exact |
| Q99835 | - | D002280 | carcinoma, basal cell | exact |
| Q99958 | 153200 | D008209 / D008538 | lymphedema / meige syndrome | -1.73986043864402 / -1.08449741891993 |
| Q99958 | 153000 | D008209 | lymphedema | -0.331482506361215 |
| Q99958 | 153400 | D008209 | lymphedema | -1.46299970927811 |
| Q99959 | 609040 | D019571 | arrhythmogenic right ventricular dysplasia | 6.62971320041613 |
| Q99972 | 137750 | D005902 | glaucoma, open-angle | 4.43609515758697 |
| Q9BPY8 | - | D002294 | carcinoma, squamous cell | -2.3946983566892 |
| Q9BQ52 | 176807 | D011471 | prostatic neoplasms | exact |
| Q9BQB4 | 269500 239100 | D010009 | osteochondrodysplasias | exact |
| Q9BQE9 | 194050 | D018980 | williams syndrome | exact |
| Q9BS16 | - | D015447 | leukemia, lymphocytic, acute | 3.51758699420083 |
| Q9BSQ5 | 603284 | D020786 | hemangioma, cavernous, central nervous system | -2.42285501548729 |
| Q9BTC0 | - | D009369 | neoplasms | -1.50110551365768 |
| Q9BUI4 | - | D012595 | scleroderma, systemic | 0.636484582357539 |
| Q9BUP3 | - | D006528 | carcinoma, hepatocellular | exact |
| Q9BWF3 | - | D004314 | down syndrome | -0.232572461395472 |
| Q9BX63 | 114480 | D001943 | breast neoplasms | exact |
| Q9BX63 | 609054 227650 | D005199 | fanconi anemia | exact |
| Q9BX84 | 602014 | D013746 | tetany | -1.08496250072116 |
| Q9BXC9 | 209900 | D020788 | bardet-biedl syndrome | exact |
| Q9BXJ0 | 605670 | D012162 | retinal degeneration | -0.0842418735878758 |
| Q9BXJ7 | 261100 | D000749 | anemia, megaloblastic | 5.17616006721624 |
| Q9BXM0 | 145900 | D015417 | hereditary motor and sensory neuropathies | exact |
| Q9BXM7 | 605909 168600 | D020734 / D010300 | parkinsonian disorders / parkinson disease | -0.655085068792169 / exact |
| Q9BXW9 | 227650 | D005199 | fanconi anemia | exact |
| Q9BY14 | - | D045743 | scleroderma, diffuse | 4.85049941375812 |
| Q9BY27 | - | D004062 | digeorge syndrome | 2.25845877406531 |
| Q9BY76 | - | D001169 | arthritis, experimental | exact |
| Q9BY76 | - | D002292 | carcinoma, renal cell | 2.55155847924951 |
| Q9BYB0 | 606232 | D002872 | chromosome deletion | -2.00122470656248 |
| Q9BYB0 | - | D001321 | autistic disorder | -2.00772389325244 |
| Q9BYB4 | - | D004062 | digeorge syndrome | 2.25845877406531 |
| Q9BYH1 | - | D002289 | carcinoma, non-small-cell lung | 7.09463761436384 |
| Q9BYJ1 | 242100 | D016113 / D017490 | ichthyosiform erythroderma, congenital / ichthyosis, lamellar | 2.34951558019456 / 8.18206058101934 |
| Q9BYX4 | 610155 | D003922 | diabetes mellitus, type 1 | 6.19757989249959 |
| Q9BZ11 | - | D001249 | asthma | exact |
| Q9BZ23 | 234200 | D006211 | hallervorden-spatz syndrome | exact |
| Q9BZ95 | - | D002289 | carcinoma, non-small-cell lung | exact |
| Q9BZ95 | - | D015454 | leukemia, lymphocytic, acute, l1 | 1.25618615935482 |
| Q9BZE9 | 606243 | D018234 | sarcoma, alveolar soft part | exact |
| Q9BZE9 | - | D002292 | carcinoma, renal cell | exact |
| Q9BZH6 | - | D005909 | glioblastoma | 2.54661873943849 |
| Q9BZS1 | 304790 | D053632 / D000744 | x-linked combined immunodeficiency diseases / anemia, hemolytic, autoimmune | 1.26336777633125 / 1.03214111819537 |
| Q9BZV2 | 607483 | D001480 | basal ganglia diseases | 0.540047706779302 |
| Q9C000 | 193200 | D014820 | vitiligo | exact |
| Q9C000 | 606579 | D008180 | lupus erythematosus, systemic | 2.21483739843615 |
| Q9GZR3 | 217095 | D004310 | double outlet right ventricle | exact |
| Q9GZR5 | 600110 | D020734 | parkinsonian disorders | -1.65041580179053 |
| Q9GZR5 | 600110 | D020734 | parkinsonian disorders | -1.65041580179053 |
| Q9GZT9 | 609820 | D011086 | polycythemia | -0.913437191735226 |
| Q9GZU1 | 252650 | D009081 | mucolipidoses | exact |
| Q9GZU5 | 310500 | D009755 | night blindness | exact |
| Q9GZV9 | 193100 | D053098 | hypophosphatemic rickets, x-linked dominant | 5.25502342161413 |
| Q9GZX3 | 217800 | D003317 | corneal dystrophies, hereditary | exact |
| Q9GZX7 | 605258 | D053306 | hyper-igm immunodeficiency syndrome | exact |
| Q9GZY6 | 194050 | D018980 | williams syndrome | exact |
| Q9H015 | 266600 | D003424 | crohn disease | exact |
| Q9H015 | - | D001172 | arthritis, rheumatoid | exact |
| Q9H0F7 | 209900 | D020788 | bardet-biedl syndrome | exact |
| Q9H0P0 | 266120 | D000743 | anemia, hemolytic | -2.27583148474641 |
| Q9H0Q3 | 603342 181500 | D012559 | schizophrenia | exact |
| Q9H0U9 | 608800 | D013398 | sudden infant death | 2.4125193901307 |
| Q9H161 | 601224 | D004062 | digeorge syndrome | -0.794376850539633 |
| Q9H173 | 248800 | D013132 | spinocerebellar degenerations | exact |
| Q9H1C4 | 610551 | D020803 | encephalitis, herpes simplex | exact |
| Q9H1R3 | 192600 | D024741 | cardiomyopathy, hypertrophic, familial | exact |
| Q9H228 | - | D001327 | autoimmune diseases | exact |
| Q9H244 | 609821 | D006470 | hemorrhage | 2.58119566437845 |
| Q9H251 | 601067 | D052245 | usher syndromes | 0.596857123648337 |
| Q9H2C0 | 256850 | D020269 | alcoholic neuropathy | -1.1108434630049 |
| Q9H2F3 | 607765 | D002780 | cholestasis, intrahepatic | -1.53528103225992 |
| Q9H3D4 | 103285 | D012202 | reye syndrome | 0.730468603073378 |
| Q9H3D4 | - | D006258 | head and neck neoplasms | -2.0324593464203 |
| Q9H3S1 | 610282 | D012174 | retinitis pigmentosa | 2.62407652489784 |
| Q9H3S1 | 610283 | D012174 | retinitis pigmentosa | 5.39140441733108 |
| Q9H4A3 | 145260 | D011546 | pseudohypoaldosteronism | exact |
| Q9H6D5 | 194050 | D018980 | williams syndrome | exact |
| Q9H6K4 | 258501 | D009896 | optic atrophy | 3.47233530195228 |
| Q9H6K4 | 165300 | D029241 | optic atrophy, autosomal dominant | 4.98937046409333 |
| Q9H6U6 | - | D001943 | breast neoplasms | exact |
| Q9H6U8 | - | D001714 | bipolar disorder | -0.411311139900679 |
| Q9H902 | 610250 | D010264 / D015419 | paraplegia / spastic paraplegia, hereditary | 0.0128317700757403 / 2.78149502482932 |
| Q9H936 | 609304 | D004831 | epilepsies, myoclonic | 3.66200885458577 |
| Q9H9Q4 | - | D009100 | multiple carboxylase deficiency | -2.3524441575785 |
| Q9H9S5 | 606612 | D009136 | muscular dystrophies | -1.15247805056448 |
| Q9H9S5 | 607155 | D049288 | muscular dystrophies, limb-girdle | 3.07169657874622 |
| Q9H9S5 | 253280 | D050336 | mulibrey nanism | -0.530914354979772 |
| Q9H9S5 | 236670 | D015792 | retinal dysplasia | -2.2899137831277 |
| Q9HB58 | 235550 | D006504 | hepatic veno-occlusive disease | 6.775059897568 |
| Q9HB96 | 227650 | D005199 | fanconi anemia | exact |
| Q9HBE1 | - | D018228 | sarcoma, small cell | 3.77337865485017 |
| Q9HBG4 | 602722 | D000141 | acidosis, renal tubular | 5.77874618920461 |
| Q9HC23 | 610628 | D017436 | kallmann syndrome | 3.15685343829517 |
| Q9HC29 | 266600 | D003424 | crohn disease | exact |
| Q9HC29 | 191390 | D003093 | colitis, ulcerative | exact |
| Q9HC29 | 609464 | D012507 | sarcoidosis | -1.49078871183516 |
| Q9HC96 | 601283 125853 | D003924 | diabetes mellitus, type 2 | exact |
| Q9HCF4 | - | D017728 | lymphoma, large-cell, ki-1 | 6.19721679327503 |
| Q9HCJ1 | 118600 | D002805 | chondrocalcinosis | 10.7907486004689 |
| Q9HCK4 | 610878 | D014718 | vesico-ureteral reflux | 6.66357576839945 |
| Q9HD26 | - | D005909 | glioblastoma | -1.72971580931865 |
| Q9NNX9 | - | D038901 | mental retardation, x-linked | 2.07508829048354 |
| Q9NP59 | 606069 | D006432 | hemochromatosis | -0.775692340799024 |
| Q9NP70 | - | D000564 | ameloblastoma | 3.97027915418696 |
| Q9NP71 | 194050 | D018980 | williams syndrome | exact |
| Q9NP85 | 600995 | D009404 | nephrotic syndrome | 2.99506870361932 |
| Q9NPC2 | - | D001943 | breast neoplasms | exact |
| Q9NPF2 | - | D015451 | leukemia, b-cell, chronic | exact |
| Q9NPI8 | 227650 | D005199 | fanconi anemia | exact |
| Q9NPJ1 | 209900 | D020788 | bardet-biedl syndrome | exact |
| Q9NQ11 | 606693 | D010300 | parkinson disease | 1.90112596308536 |
| Q9NQG7 | 203300 | D022861 | hermanski-pudlak syndrome | exact |
| Q9NQW8 | 262300 | D003117 | color vision defects | 2.97580425794886 |
| Q9NQZ3 | - | D009845 | oligospermia | -2.26169437542798 |
| Q9NR30 | - | D020252 | gastric antral vascular ectasia | 3.38916407342301 |
| Q9NR50 | 603896 | D002493 | central nervous system diseases | -2.27852450745258 |
| Q9NR90 | - | D009845 | oligospermia | -2.26169437542798 |
| Q9NRA2 | 604369 | D029461 | sialic acid storage disease | exact |
| Q9NRA2 | 269920 | D029461 | sialic acid storage disease | exact |
| Q9NRA2 | 236750 | D015160 | hydrops fetalis | exact |
| Q9NRD8 | 607200 | D003409 | congenital hypothyroidism | exact |
| Q9NRG9 | 231550 | D000309 | adrenal insufficiency | 0.631517202916899 |
| Q9NRI5 | 604906 181500 | D012559 | schizophrenia | exact |
| Q9NRI5 | 181500 | D011618 / D012559 | psychotic disorders / schizophrenia | exact / exact |
| Q9NRM1 | 104500 | D000567 | amelogenesis imperfecta | -1.21381573109469 |
| Q9NRM1 | 608563 | D003744 | dental enamel hypoplasia | -0.625625085132296 |
| Q9NS00 | - | D005922 | glomerulonephritis, iga | exact |
| Q9NS82 | - | D003555 | cystinuria | exact |
| Q9NS86 | - | D005909 | glioblastoma | 1.45344529780426 |
| Q9NSC2 | 113650 | D019280 | branchio-oto-renal syndrome | 4.12045646411932 |
| Q9NSU2 | 225750 | D004660 | encephalitis | -2.14884027432034 |
| Q9NSU2 | 610448 | D002647 | chilblains | 2.06050770048068 |
| Q9NTI5 | - | D004938 | esophageal neoplasms | 4.52160320400427 |
| Q9NUW8 | 607250 | D020754 | spinocerebellar ataxias | 0.0197591875976308 |
| Q9NVA2 | - | D015467 | leukemia, neutrophilic, chronic | exact |
| Q9NVI1 | 227650 | D005199 | fanconi anemia | exact |
| Q9NVK5 | - | D009196 | myeloproliferative disorders | 0.609720507513883 |
| Q9NW38 | 227650 | D005199 | fanconi anemia | exact |
| Q9NWW5 | 601780 | D009472 | neuronal ceroid-lipofuscinoses | 6.24903384080263 |
| Q9NXB0 | 249000 | D008467 | meckel diverticulum | -1.75501587783746 |
| Q9NXR8 | 275355 | D002294 | carcinoma, squamous cell | 1.29152651980015 |
| Q9NYJ7 | 277300 | D004413 | dysostoses | -2.10472668281448 |
| Q9NZ94 | 300494 | D020817 | asperger syndrome | -1.1131561141003 |
| Q9NZC2 | 221770 | D000544 | alzheimer disease | 1.14052344313828 |
| Q9NZC7 | 133239 | D002294 / D004938 | carcinoma, squamous cell / esophageal neoplasms | 4.49953163982265 / exact |
| Q9NZD8 | 248900 | D015419 | spastic paraplegia, hereditary | 3.13821833877714 |
| Q9NZJ5 | 226980 | D010009 | osteochondrodysplasias | -1.18961581110783 |
| Q9NZN1 | 300143 | D038901 | mental retardation, x-linked | 4.64405280440485 |
| Q9NZN5 | - | D015447 | leukemia, lymphocytic, acute | 3.51758699420083 |
| Q9NZQ3 | - | D016609 | neoplasms, second primary | 1.31626229432038 |
| Q9NZR4 | 122000 | D003317 | corneal dystrophies, hereditary | 0.527397943934729 |
| Q9NZR4 | 148300 | D007640 | keratoconus | exact |
| Q9NZW4 | 125490 | D003811 | dentinogenesis imperfecta | 9.71616870183507 |
| Q9NZW4 | 605594 | D003811 | dentinogenesis imperfecta | -0.907835620266889 |
| Q9P0J1 | 608782 | D015325 | pyruvate dehydrogenase complex deficiency disease | 0.420729808730402 |
| Q9P212 | 610725 | D009404 | nephrotic syndrome | 0.830332213372915 |
| Q9P2F6 | - | D015451 | leukemia, b-cell, chronic | exact |
| Q9P2R6 | - | D009447 | neuroblastoma | -2.15633008436479 |
| Q9UBB4 | 603516 | D020754 | spinocerebellar ataxias | 3.13273744085086 |
| Q9UBC1 | 180300 | D001172 | arthritis, rheumatoid | exact |
| Q9UBC3 | 242860 | D017074 | common variable immunodeficiency | 1.93794814144264 |
| Q9UBF9 | 159000 | D049288 | muscular dystrophies, limb-girdle | 3.41676146672812 |
| Q9UBF9 | 182920 | D017696 | myopathies, nemaline | -2.35782901029293 |
| Q9UBK8 | 601634 | D009436 | neural tube defects | 0.874749199640761 |
| Q9UBM7 | 270400 | D019082 | smith-lemli-opitz syndrome | exact |
| Q9UBP0 | 182601 | D010264 / D015419 | paraplegia / spastic paraplegia, hereditary | 1.28733979081587 / 3.80110144142142 |
| Q9UBP5 | - | D006345 | heart septal defects, ventricular | 3.70758825707248 |
| Q9UBQ7 | 260000 | D006960 | hyperoxaluria, primary | 2.07572826101744 |
| Q9UBR4 | 262600 | D007018 | hypopituitarism | exact |
| Q9UBV7 | 130070 | D004535 | ehlers-danlos syndrome | 3.8547745246372 |
| Q9UBW7 | - | D015460 | leukemia-lymphoma, t-cell, acute, htlv-i-associated | -2.17019389346424 |
| Q9UBX0 | 182230 | D004393 | dwarfism, pituitary | -1.90929585797411 |
| Q9UBX0 | 262600 | D004393 / D007018 | dwarfism, pituitary / hypopituitarism | 4.2148154535033 / exact |
| Q9UBX2 | - | D020391 | muscular dystrophy, facioscapulohumeral | exact |
| Q9UBX5 | 123700 | D003483 | cutis laxa | 2.74566027365228 |
| Q9UBX5 | 219100 | D003483 | cutis laxa | 2.81085466105711 |
| Q9UBX5 | 608895 | D008268 | macular degeneration | -1.52386102497989 |
| Q9UBY8 | 600143 | D009472 | neuronal ceroid-lipofuscinoses | 6.09202603514937 |
| Q9UBY8 | 610003 | D008607 | mental retardation | 0.0854244857268229 |
| Q9UDR5 | 238700 | D020167 | hyperlysinemias | exact |
| Q9UDT6 | 194050 | D018980 | williams syndrome | exact |
| Q9UEF7 | - | D007676 | kidney failure, chronic | 4.37595217463345 |
| Q9UET6 | 309549 | D038901 | mental retardation, x-linked | 4.88093771863234 |
| Q9UGJ0 | 194200 | D014927 | wolff-parkinson-white syndrome | exact |
| Q9UGJ0 | 600858 | D014927 | wolff-parkinson-white syndrome | 2.00385776758295 |
| Q9UGJ0 | 261740 | D006008 | glycogen storage disease | 4.48911457278727 |
| Q9UGM3 | 137800 | D005910 / D005909 | glioma / glioblastoma | 2.08930536465427 / exact |
| Q9UGP8 | 174050 | D016891 / D008107 | polycystic kidney, autosomal dominant / liver diseases | 2.67044314557056 / 1.37554529844664 |
| Q9UHB7 | - | D015447 | leukemia, lymphocytic, acute | exact |
| Q9UHD8 | - | D007950 | leukemia, myelocytic, acute | -1.3367082899624 |
| Q9UHD8 | 162100 | D020968 | brachial plexus neuritis | 6.5498560399636 |
| Q9UHF7 | 190350 | D015826 | langer-giedion syndrome | 6.38525921201286 |
| Q9UHF7 | 150230 | D015826 | langer-giedion syndrome | exact |
| Q9UHF7 | 190351 | D015826 | langer-giedion syndrome | 6.19963551767133 |
| Q9UHN1 | 610131 | D017246 | ophthalmoplegia, chronic progressive external | 1.09427128587827 |
| Q9UI10 | 603896 | D002493 | central nervous system diseases | -2.27852450745258 |
| Q9UI10 | 603896 | D002493 | central nervous system diseases | -2.27852450745258 |
| Q9UI46 | 242650 | D007619 | kartagener syndrome | exact |
| Q9UI46 | 244400 | D007619 | kartagener syndrome | exact |
| Q9UIF7 | 608456 | D011125 | adenomatous polyposis coli | -1.96657310586661 |
| Q9UIF7 | 137215 | D013274 | stomach neoplasms | exact |
| Q9UIG0 | 194050 | D018980 | williams syndrome | exact |
| Q9UIJ5 | - | D006528 | carcinoma, hepatocellular | 0.836104351731148 |
| Q9UIR0 | 181000 | D012507 | sarcoidosis | exact |
| Q9UJ55 | - | D011218 | prader-willi syndrome | exact |
| Q9UJJ9 | 252605 | D009081 | mucolipidoses | 0.791522011071164 |
| Q9UJQ4 | 607323 | D004370 | duane retraction syndrome | exact |
| Q9UJS0 | 603471 | D020159 | citrullinemia | -0.44403768710241 |
| Q9UJS0 | 605814 | D002780 | cholestasis, intrahepatic | 5.09827977732101 |
| Q9UJW2 | - | D009395 | nephritis, interstitial | 2.16837420942593 |
| Q9UJY1 | 158590 | D002607 | charcot-marie-tooth disease | 2.26222869335076 |
| Q9UJY1 | 608673 | D002607 | charcot-marie-tooth disease | 0.889395988497072 |
| Q9UK53 | 275355 | D002294 | carcinoma, squamous cell | 1.29152651980015 |
| Q9UKJ5 | - | D007950 | leukemia, myelocytic, acute | 1.23080265610631 |
| Q9UKM9 | - | D020031 | epstein-barr virus infections | -0.0187852977652317 |
| Q9UKU0 | - | D009190 | myelodysplastic syndromes | 0.692357449466273 |
| Q9UKU0 | - | D007950 | leukemia, myelocytic, acute | 4.14556035978551 |
| Q9UKU0 | - | D015472 | leukemia, eosinophilic, acute | exact |
| Q9UKX2 | 605637 | D018979 | myositis, inclusion body | 0.755934027398273 |
| Q9UKY4 | 236670 | D015792 | retinal dysplasia | -2.2899137831277 |
| Q9UL17 | 208550 | D009298 | nasal polyps | 4.19301016542201 |
| Q9UL17 | 208550 | D009298 | nasal polyps | 4.19301016542201 |
| Q9UL49 | - | D014355 | chagas disease | exact |
| Q9ULC8 | - | D012559 | schizophrenia | exact |
| Q9ULL8 | 300434 | D038901 | mental retardation, x-linked | -0.882041958124988 |
| Q9ULL8 | - | D038901 | mental retardation, x-linked | exact |
| Q9ULL8 | - | D038901 | mental retardation, x-linked | exact |
| Q9ULV1 | 133780 | D012178 | retinopathy of prematurity | exact |
| Q9ULV5 | 116800 | D002386 | cataract | -1.55521199484682 |
| Q9UM07 | 180300 | D001172 | arthritis, rheumatoid | exact |
| Q9UM47 | 125310 | D046589 | cadasil | exact |
| Q9UM54 | 192600 | D024741 | cardiomyopathy, hypertrophic, familial | exact |
| Q9UM73 | - | D008228 | lymphoma, non-hodgkin | 5.72497016853913 |
| Q9UM73 | - | D017728 | lymphoma, large-cell, ki-1 | 6.19721679327503 |
| Q9UMD9 | 226650 | D016110 | epidermolysis bullosa simplex | -0.386916265556435 |
| Q9UMN6 | - | D021441 | carcinoma, pancreatic ductal | 3.77617948817023 |
| Q9UMQ3 | - | D019465 | craniofacial abnormalities | -0.254496436368341 |
| Q9UMX1 | 155255 | D008527 | medulloblastoma | exact |
| Q9UMX9 | 606574 | D016115 | albinism, oculocutaneous | 2.03766167728099 |
| Q9UN76 | 300306 | D009765 | obesity | -1.84998995380116 |
| Q9UNA1 | 607785 | D015479 | leukemia, myelomonocytic, acute | 0.960874583246403 |
| Q9UNE0 | 129490 224900 | D053360 / D053359 | ectodermal dysplasia, hypohidrotic, autosomal recessive / ectodermal dysplasia 3, anhidrotic | 6.27509058574399 / exact |
| Q9UNE7 | - | D015462 | leukemia, lymphocytic, chronic | -1.34273065643788 |
| Q9UP52 | 604250 | D006432 | hemochromatosis | -0.725403986000023 |
| Q9UPN9 | 188550 | D002291 | carcinoma, papillary | 3.4424308069019 |
| Q9UPP1 | 300263 | D038901 | mental retardation, x-linked | 4.33463829220468 |
| Q9UPW6 | - | D002972 | cleft palate | 3.95567266456643 |
| Q9UPZ3 | 203300 | D022861 | hermanski-pudlak syndrome | exact |
| Q9UQ90 | 607259 | D010264 / D015419 | paraplegia / spastic paraplegia, hereditary | 0.934456901871545 / 3.4666396203421 |
| Q9UQE7 | 610759 | D003635 | de lange syndrome | 5.05084454033625 |
| Q9UQF0 | - | D009103 | multiple sclerosis | 0.142805670830561 |
| Q9UQF0 | - | D009103 | multiple sclerosis | exact |
| Q9UQF2 | 125853 | D003924 | diabetes mellitus, type 2 | exact |
| Q9UQN3 | 600795 | D003704 | dementia | 1.31333471849041 |
| Q9Y210 | 603965 | D005923 | glomerulosclerosis, focal segmental | 7.31188130462033 |
| Q9Y215 | 603034 | D020294 | myasthenic syndromes, congenital | 2.18039544390362 |
| Q9Y219 | - | D052245 | usher syndromes | 0.704067948349306 |
| Q9Y223 | 269921 | D029461 | sialic acid storage disease | exact |
| Q9Y223 | 600737 | D018979 | myositis, inclusion body | 0.869626918214156 |
| Q9Y223 | 605820 | D049310 | distal myopathies | 1.48358860208508 |
| Q9Y238 | 211980 | D008175 | lung neoplasms | exact |
| Q9Y238 | 133239 | D004938 | esophageal neoplasms | exact |
| Q9Y238 | - | D007680 | kidney neoplasms | exact |
| Q9Y253 | 278750 | D014983 | xeroderma pigmentosum | 2.00420959920947 |
| Q9Y281 | 610687 | D017696 | myopathies, nemaline | 3.7181132822658 |
| Q9Y287 | 176500 | D003704 / D016657 | dementia / cerebral amyloid angiopathy | 1.24911969250961 / 2.37342390560954 |
| Q9Y287 | 117300 | D003704 | dementia | 0.841655552064125 |
| Q9Y2I7 | 121850 | D003317 | corneal dystrophies, hereditary | -1.37980101888847 |
| Q9Y2Q5 | 610798 | D053632 | x-linked combined immunodeficiency diseases | 0.227667363377122 |
| Q9Y2T1 | 114500 | D015179 | colorectal neoplasms | exact |
| Q9Y2U8 | 166700 | D010023 | osteopoikilosis | 1.70299617983792 |
| Q9Y3A5 | 260400 | D010188 | exocrine pancreatic insufficiency | -2.0578032040196 |
| Q9Y3D3 | 610498 | D028361 | mitochondrial diseases | 3.01041419606359 |
| Q9Y3Q4 | 163800 | D012804 / D001281 | sick sinus syndrome / atrial fibrillation | 2.81818420835108 / 2.91392068053024 |
| Q9Y458 | 303400 | D002972 | cleft palate | 2.0463658463787 |
| Q9Y4J8 | 606617 | D006330 | heart defects, congenital | -1.9880223579252 |
| Q9Y4P3 | 194050 | D018980 | williams syndrome | exact |
| Q9Y5K2 | 204700 | D000567 | amelogenesis imperfecta | -1.57692150179535 |
| Q9Y5K6 | 607832 | D005923 | glomerulosclerosis, focal segmental | 7.16976519110048 |
| Q9Y5Y5 | 214100 | D015211 | zellweger syndrome | exact |
| Q9Y606 | 600462 | D000756 | anemia, sideroblastic | 0.847304161731253 |
| Q9Y620 | - | D009369 | neoplasms | -1.64725631865348 |
| Q9Y653 | 606854 | D002524 | cerebellar ataxia | -2.35192641318002 |
| Q9Y672 | 603147 | D018981 | carbohydrate-deficient glycoprotein syndrome | 4.21336279944396 |
| Q9Y6A1 | 236670 | D015792 | retinal dysplasia | -2.2899137831277 |
| Q9Y6A1 | 609308 | D049288 | muscular dystrophies, limb-girdle | 3.15260104994126 |
| Q9Y6B6 | 246700 | D012153 | retention (psychology) | -2.36797193961328 |
| Q9Y6B6 | 607689 | D000795 | fabry disease | 3.46699540869876 |
| Q9Y6B6 | 607692 | D013132 | spinocerebellar degenerations | 0.0933498990680643 |
| Q9Y6C5 | 155255 | D008527 | medulloblastoma | exact |
| Q9Y6C5 | 605462 | D002280 | carcinoma, basal cell | 4.8579950187559 |
| Q9Y6D9 | - | D009369 | neoplasms | exact |
| Q9Y6H5 | 168600 | D010300 | parkinson disease | exact |
| Q9Y6H6 | 170400 | D020514 | hypokalemic periodic paralysis | exact |
| Q9Y6H6 | 188580 | D020514 / D010245 | hypokalemic periodic paralysis / paralyses, familial periodic | 5.20584352151651 / -0.511706850077947 |
| Q9Y6J6 | 603796 | D008133 | long qt syndrome | 1.55474574341788 |
| Q9Y6K9 | 300291 | D004476 | ectodermal dysplasia | 5.61644612240979 |
| Q9Y6K9 | 300301 | D004476 | ectodermal dysplasia | -0.709557335125627 |
| Q9Y6K9 | 300584 | D004476 | ectodermal dysplasia | 2.29463549024452 |
| Q9Y6K9 | 308300 | D007184 | incontinentia pigmenti | exact |
| Q9Y6M1 | - | D006528 | carcinoma, hepatocellular | exact |
| Q9Y6N7 | - | D001943 | breast neoplasms | 2.84568615464324 |
| Q9Y6N9 | 276904 | D052245 | usher syndromes | 0.346857123648337 |
| Q9Y6Q6 | 602080 | D010001 | osteitis deformans | exact |
| Q9Y6R1 | 604278 | D000141 | acidosis, renal tubular | exact |
| Q9Y6Y1 | - | D009837 | oligodendroglioma | 6.97708578619142 |
