# Supplementary material for: Mapping proteins to disease terminologies: from UniProt to MeSH
Source: BMC Bioinformatics. 2008 Apr 29;9(Suppl 5):S3. doi: 10.1186/1471-2105-9-S5-S3 (PMC2367626; doi:10.1186/1471-2105-9-S5-S3)
Supplement: Additional file 3 — This file contains the regular expressions used to extract disease names from the UniProtKB/Swiss-Prot disease comment lines (pdf format). [file 1471-2105-9-S5-S3-S3.pdf]

Regular Expressions used to extract the disease names from the Swiss-Prot disease comment lines

**(1) Starter expressions**	**(2) Specific stop words**	**(3) Termination term**
Cause(s) of /a	susceptibility to	also known as
involved in	development of	but
(can) contribute(s) to	genetic predisposition for	which
associated/association with	developing	an
correlated with	pathogenesis of	due to
responsible for	subset of	in condition(s) such as
contributor to	various types of	.
result(s)/resulting in	some form of	[MIM:
lead(s) to	increased risk of	
induce(s)		
defective in		
individual(s) with		
patient(s) with/suffering from		
reduce(s)		
influence(s)		
deleted in		
down-regulated in		
found in		
implicated in		
predispose(s) to		
favor		
antigen of		
antigen for		
thought to be an		
role in		
could impart		
mediate(s)		
candidate (gene)		

(1) Expressions used to extract the part of the string containing the disease name. (2) Terms removed from the string extracted. (3) Expressions indicating the end of the disease name.
